# Engineering Enantiocomplementary Protoglobins for Stereoconvergent Construction of *N*‑Alkylated α‑Aminoketones

**DOI:** 10.1021/jacs.5c17989

**Published:** 2026-05-01

**Authors:** Zi-Yang Qin, Zi-Qi Li, Chi Zhang, Jan L. Heise, Runze Mao, Sophia J. Wu, K. N. Houk, William A. Goddard III, Frances H. Arnold

**Affiliations:** † Division of Chemistry and Chemical Engineering, California Institute of Technology, Pasadena, California 91125, United States; ‡ Materials and Process Simulation Center, 6469 California Institute of Technology, Pasadena, California 91125, United States; ¶ Department of Chemistry and Biochemistry, 8783 University of California, Los Angeles, Los Angeles, California 90095, United States

## Abstract

The synthesis of enantiopure compounds from a mixture of *E/Z* alkenes represents a notable challenge in synthetic chemistry. While enzymes excel in achieving unparalleled selectivity, their inherent specificity often confines activity to a single stereoisomeric substrate, consequently restricting the overall efficiency of such transformations. Here, we demonstrate that protoglobin-derived hemoproteins can catalyze stereoconvergent intermolecular amination using simple *N*-alkyl hydroxylamines as nitrene precursors, a transformation which remains elusive in synthetic chemistry. These engineered enzymes process *E/Z* mixtures of silyl enol ethers, enabling the precise incorporation of *N*-alkyl amino moieties (−NHAlkyl) into diverse molecular structures (up to 79% yield and 95% ee). Two complementary protoglobin variants were engineered using directed evolution to enable enantiodivergent synthesis of both enantiomers of α-aminoketones. This enzymatic platform achieves *stereoconvergent* and *enantiodivergent* transformations, facilitating the conversion of simple chemicals into an array of valuable pharmaceutical compounds featuring aminoketone functionalities.

## Graphical abstract

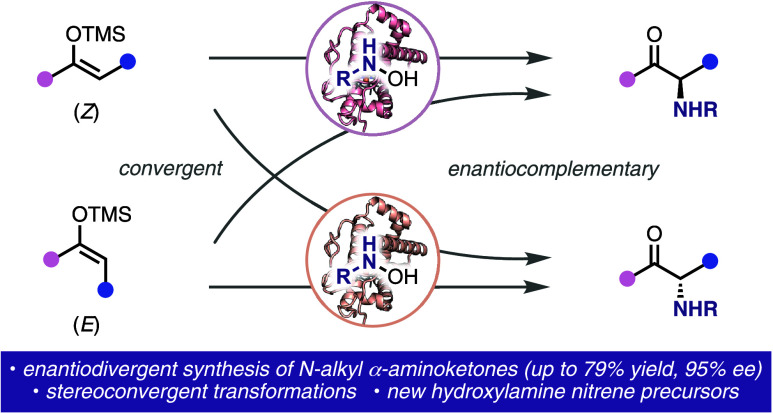

Alkenes are valuable feedstocks in chemical synthesis, serving as versatile building blocks to construct molecular complexity.^1,2^ Their inherent reactivity enables a wide array of transformations, allowing access to diverse functional groups and facilitating the conversion of planar, two-dimensional frameworks into three-dimensional architectures.^2^ The stereochemistry of alkenes and its effect on reaction outcomes have been extensively investigated to elucidate mechanistic pathways, optimize synthetic strategies, and expand the scope of stereoselective transformations.^3,4^ Both *E*- and *Z*-alkenes are essential components of many natural products, such as fatty acids, polyketides, and terpenoids.^5,6^ Nature’s fine-tuned catalytic machinery not only orchestrates the biosynthesis of a specific alkene isomer but also specifically functionalizes one alkene isomer to regulate biological processes such as metabolism, membrane dynamics, and natural product biosynthesis (Scheme 1A).^5,7–10^ On the other hand, the limited accessibility of stereochemically pure *E*- and *Z*-alkenes and their intrinsic differences in reactivity and selectivity pose significant challenges to state-of-the-art methodologies for enantioselective alkene functionalization. In this context, stereoconvergent strategies, where both *E*- and *Z*-alkene isomers are transformed into a single enantiomerically pure product, offer a powerful solution, emulating Nature’s precision while obviating the need for stereochemically pure starting materials.^11^

**Scheme 1:**
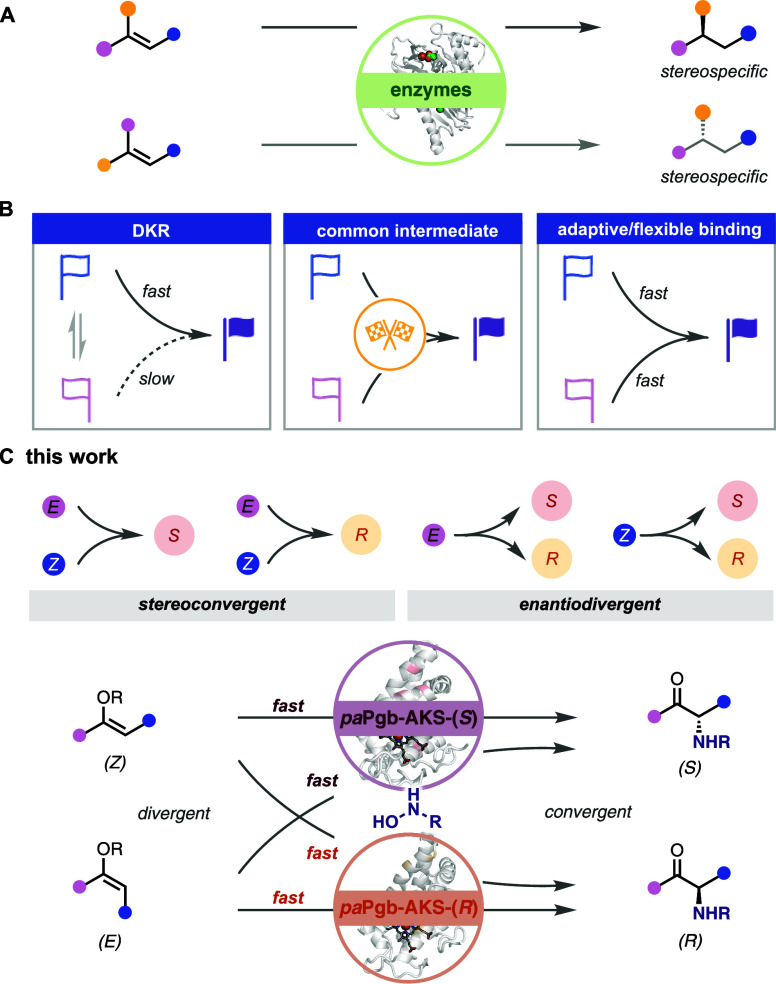
Stereoconvergent Construction of *N*-Alkylated Aminoketones Using Hydroxylamines

Three distinct strategies enable stereoconvergence in biocatalysis (Scheme 1B). Dynamic kinetic resolution (DKR) exploits rapid in situ interconversion of stereoisomers to yield enantiopure products,^12–14^ while a second approach funnels substrates through a common intermediate.^15–23^ In both cases, convergence depends on auxiliary chemical systems to unify isomers into a single substrate or achiral intermediate. In contrast, a third, yet underexplored strategy achieves enantioconvergence solely via enzyme engineering, particularly active-site modification. Here, a single catalyst recognizes both isomers through distinct binding modes and channels them to the same enantiomeric product, enabled by a flexible binding environment without external auxiliaries.^16,24^ However, this strategy is constrained by intrinsic enzyme specificity dictated by structural complementarity, which tightly links substrate and product selectivity and limits simultaneous processing of multiple isomers.

In 2023, our group demonstrated P411-catalyzed stereoconvergent alkylation of (*E*/*Z*)-trisubstituted silyl enol ether *via* carbene transfer.^25^ These findings stimulated interest in two key dimensions: (1) extending stereoconvergent biocatalysis to other enzyme scaffolds beyond cytochrome P411 and (2) applying a stereoconvergent strategy to other bond-forming reactions, such as C–N bond formation. Here, we explore this possibility by engineering thermostable protoglobins to catalyze enantioselective C–N bond formation *via* intermolecular alkyl nitrene transfer (Scheme 1C). Silyl enol ethers, featuring electron-rich double bonds, react with electrophilic heme-nitrenoids to afford α-aminoketones, which are valuable motifs in pharmaceuticals and synthetic intermediates.

To probe our reaction design, we initiated our investigation with trimethylsilyl enol ether **1a** (Figure 1). Though hydroxylamine has been established as an effective nitrene precursor for incorporating a free – NH_2_ group by protoglobin-catalyzed C–H amination and aminohydroxylation reactions,^26^ *N*-alkylated hydroxylamine derivatives remain unexplored. This is primarily due to their inherent instability, reduced reactivity, and propensity to undergo an unproductive 1,2-hydride shift, leading irreversibly to undesired imines.^27^ *N*-alkyl moieties are prevalent in bioactive compounds compared to free amines,^28,29^ as they enhance metabolic stability, mitigate off-target effects, and modulate key physicochemical properties, such as lipophilicity and basicity.^30–34^ Conventional synthetic strategies for *N*-alkyl installation, such as reductive alkylation of amines and transition-metal-catalyzed alkylation with alcohols, often involve harsh reducing conditions, poor selectivity control, or toxic reagents and typically require the preinstallation of amino functionalities.^35–41^ To address these challenges, we screened a series of *N*-alkyl hydroxylamine derivatives bearing different leaving groups,^42^ aiming to enable the direct incorporation of *N*-alkyl moieties *via* enzyme-catalyzed nitrene transfer (see Supporting Information, Section I).

**Figure 1:**
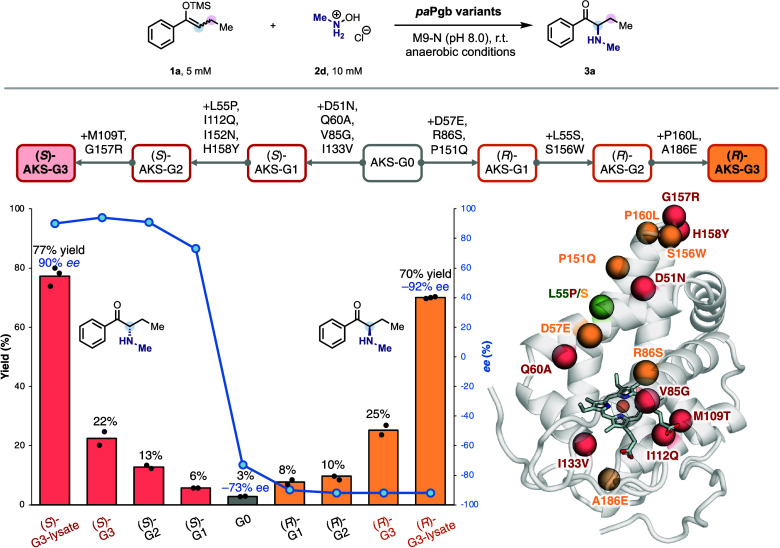
**Directed evolution for intermolecular amination of silyl enol ethers.** (A) Evolutionary trajectory of **AKS-(** *S* **)-G3-5332** and **AKS-(** *R* **)-G3-5335** for the synthesis of *N*-methyl aminoketone **3a**. Both variants were derived from the common ancestor **AKS-G0-5329** through divergent evolutionary trajectories. Indicated mutations are relative to **AKS-G0-5329**. (B) The mutated residues for both **AKS-(** *S* **)-G3-5332** (pink) and **AKS-(** *R* **)-G3-5335** (light yellow) are highlighted in the AlphaFold3-generated protoglobin structure.

To our delight, commercially available *N*-methylhydroxylamine (HONHMe·HCl, **2d**) emerged as the most effective nitrene precursor. Among the identified hits, a ***P*** *yrobaculum* ***a*** *rsenaticum* protoglobin (*pa* **Pgb**) variant bearing five mutations from wild-type *pa* **Pgb** exhibited the highest catalytic efficiency, affording the desired product in 3% yield and – 73% enantiomeric excess (ee) (Figure 1). This variant was designated as *pa* **Pgb**-**A**mino**K**etone **S**ynthase (**AKS**)-**G0-5329**. Given the stability of **2d** in aqueous solutions and the formation of water as the sole byproduct, we leveraged **2d** as the nitrene precursor for the directed evolution campaign.

We employed a combination of site-saturation mutagenesis (SSM), random mutagenesis (RM), and staggered extension process (StEP) recombination to comprehensively explore the functional space of *pa* **Pgb-AKS**. Screening identified *pa* **Pgb-AKS-(** *R* **)-G1-5333**, which catalyzed the desired reactions with excellent ee and a 3-fold yield boost. Moreover, a variant generated from *pa* **Pgb-AKS-G0-5329** bearing four additional mutations (D51N, Q60A, V85G, and I133V) inverted enantioselectivity, affording the opposite enantiomer of the desired product in 6% yield and 60% ee. Subsequent explorations revealed that the single **V85G** mutation was responsible for this inversion (Table S4). We propose that reduced steric repulsion between the ethyl group of **1a** and the mutated residue shifts the substrate binding, thereby reversing selectivity. Recognizing the potential,^43^ we pursued divergent evolution to generate two variants capable of selectively producing either enantiomer of the desired α-aminoketone. Two additional rounds of directed evolution provided *pa* **Pgb-AKS-(** *S* **)-G3-5332** (L55P, M109T, I112Q, I152N, G157R, H158Y) and *pa* **Pgb-AKS-(** *R* **)-G3-5335** (L55S, S156W, P160L, A186E), both of which delivered >20% yield with excellent enantioselectivity.

Among different reaction formats using whole-cell, lysate, and purified protein conditions, lysate-based reactions were found to be the most efficient and consistent, affording a 2-fold improvement in yield over whole-cell conditions and excellent ee within two hours (see Supporting Information, Section II), with unreacted starting material comprising the remainder of the mass balance. Further investigation revealed that the incomplete conversion was due to rapid depletion of the *N*-alkyl hydroxylamine (see Supporting Information, Section II), likely due to a competing nitrene reduction pathway.^44,45^ Under lysate conditions, 2.0 equiv. of the nitrene precursor were fully consumed within one hour. To overcome this, an additional 2.0 equiv. of *N*-methyl hydroxylamine (**2d**) were added after one hour, enhancing the final yields to 77% for *pa* **Pgb-AKS-(** *S* **)-G3-5332** and 70% for *pa* **Pgb-AKS-(** *R* **)-G3-5335**, without compromising ee.

To further investigate the influence of substrate modifications on activity and selectivity, variations in aromatic functionalities, alkyl nitrene precursors, and silyl leaving groups were systematically explored (Figure 2). We first examined substrates with both shorter and longer carbon chains. The results revealed that extending the carbon chain (**1c**) preserved high enantioselectivity, whereas shortening it (**1d**) led to stereochemical erosion, with both engineered enzymes affording products with <50% ee. Although *pa* **Pgb-AKS-(** *S* **)-G3-5332** and *pa* **Pgb-AKS-(** *R* **)-G3-5335** were engineered for the template transformation, both demonstrated substantial compatibility with diverse substitutions on the aromatic ring. An array of positional substitutions, 4-F (**1e**), 4-Me (**1f**), 4-OMe (**1g**), 4-Cl (**1h**), 3-Cl (**1j**), 2-Me (**1k**), proved compatible with the enzymatic platform, furnishing products in modest to good yields with moderate to excellent enantioselectivity. Other aromatic substrates including naphthalene **(1l)** and thiophene **(1m)** were also converted into their corresponding *N*-methylated ketones with enantioselectivity up to 94% ee.

**Figure 2:**
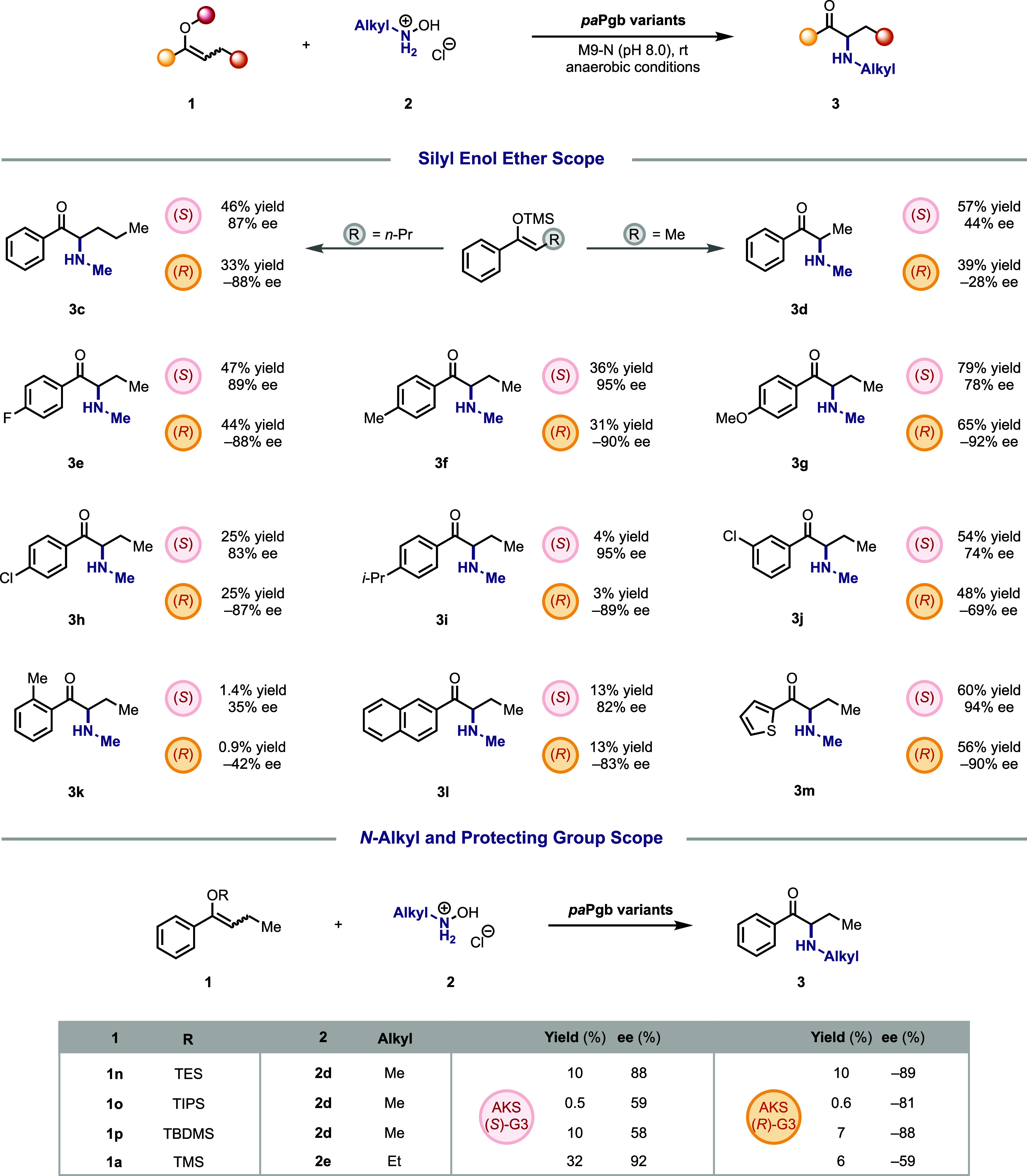
**Substrate scope studies of stereoconvergent amination reactions.** The experiments were performed at analytical scale using the cell lysate of *E. coli* (OD_600_ = 30) that expressed the *pa* **Pgb-AKS-(** *S* **)-G3-5332** and *pa* **Pgb-AKS-(** *R* **)-G3-5335** enzymes with 5 mM substrate (**1a–1p**) and 20 mM **2d** or **2e** in M9-N buffer (pH = 8.0) under anaerobic conditions. Yields were quantified by LC-MS based on the calibration curves of the corresponding reference products. Enantioselectivities were measured by LC-MS on a chiral phase.

Next, we examined a series of silyl protecting groups and alkyl nitrene precursors. Multiple silyl protecting groups were tolerated in the system, including bulky groups such as TIPS (**1o**) and TBDMS (**1p**), which still enabled detectable product formation, though affording diminished ee with **(** *S* **)-G3**-5332. While *N*-ethyl hydroxylamine (**2e**) was successfully incorporated to generate *N*-ethyl aminoketone (**3q**), bulkier nitrene precursors such as *N*-isopropyl (**2f**) and *N*-tert-butyl (**2g**) hydroxylamines failed to react. It is well established from both literature^46–48^ and industrial biocatalytic process development^49–51^ that directed evolution can reliably amplify low target activities to synthetically useful levels.

To elucidate how the engineered protoglobins achieve the transformation of a stereoisomeric mixture of (*E*)- and (*Z*)-**1a** into an enantiopure product, we performed a series of mechanistic investigations. Stereopure isomers of **1a**, (*Z*)-**1a** (99% stereopurity) and (*E*)-**1a** (99% stereopurity) were isolated and subjected individually to enzymatic reactions (Figure 3). Using *pa* **Pgb-AKS-(** *S* **)-G3-5332**, both (*Z*)-**1a** and (*E*)-**1a** were efficiently converted into the same enantiomer of the product in high yields (77% and 72%, respectively) and excellent enantioselectivity (90% ee for both). Similarly, *pa* **Pgb-AKS-(** *R* **)-G3-5335** afforded the opposite enantiomer in good yields (up to 70%) and excellent enantioselectivity. Kinetic experiments with (*E*)-**1a** and (*Z*)-**1a** confirmed no interconversion of the isomers. Both substrates exhibited comparable reaction rates, with v_Z_/v_E_ = 0.68 for **(** *S* **)-G3-5332** and 1.17 for **(** *R* **)-G3-5335** (Figure 3 and Scheme S1). These findings strongly indicate that stereometric isomers converge to the same enantiomeric product. Based on prior knowledge^24,25^ and molecular docking analyses (see Supporting Information, Section X), we hypothesize that this stereoconvergence arises from distinct binding modes adopted by the (*E*)-**1a** and (*Z*)-**1a** isomers within the active site of the engineered protoglobins. Structural studies to define how the active site accommodates both isomers to afford a single enantiopure product are challenging without a high-resolution crystal structure of the engineered enzyme, which we have not been able to obtain to date.

**Figure 3:**
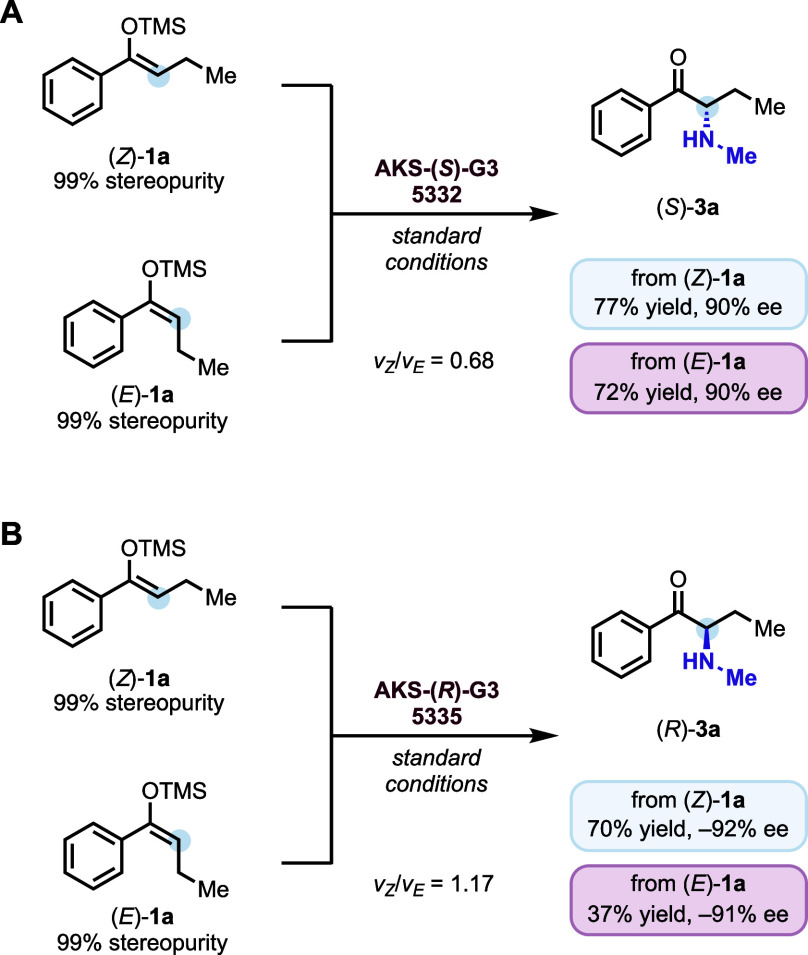
**Mechanistic studies of stereoconvergent biocatalytic amination.** Nitrene transfer reactions catalyzed by **AKS-(** *S* **)-G3-5332** and **AKS-(** *R* **)-G3-5335** using (*Z*)-**1a** and (*E*)-**1a**.

The mechanism by which the enzymes mediate this unprecedented transformation is of particular interest, particularly regarding (1) the pathway of C–N bond formation from the novel *N*-alkylated nitrenoid intermediate and (2) how *N*-alkyl substitution alters the electronic structure relative to the well-studied unsubstituted heme–nitrenoid system.^52,53^ To address these questions, density functional theory (DFT) calculations were performed using a truncated model system (Int1, see Figure S3 and the Supporting Information for details). Exploration of the reaction landscape indicates that C–N bond formation proceeds through a radical pathway from an iron–nitrenoid active species (Int2) analogous to previously reported systems,^54–56^ involving direct coupling between the nitrenoid and the substrate to form an α-silyl ether radical intermediate (Int3), followed by deprotection and intramolecular electron transfer to afford the *N*-alkylated intermediate (Int4), followed by protonation to release the α-aminoketone product and Int1. This mechanism is consistent with the radical clock experiment, which affords the ring-retaining product **3b** as the sole product (Figure S2). Hirshfeld spin population analysis (Figure 4) reveals that *N*-alkyl substitution shifts spin density from Fe to the nitrene *N* atom.^57–60^ Whereas the unsubstituted system features greater spin localization on Fe, the *N*-alkylated nitrenoid exhibits stronger radical character at the nitrene *N* atom, with a similar trend observed in the C–N bond-forming transition state. Together, these results identify direct radical C–N coupling as the operative pathway and show that *N*-alkyl substitution modulates the electronic structure of the nitrenoid intermediate. These findings highlight substitution as a useful handle for tuning spin distribution and potentially influencing reactivity in nitrene-transfer systems.

**Figure 4:**
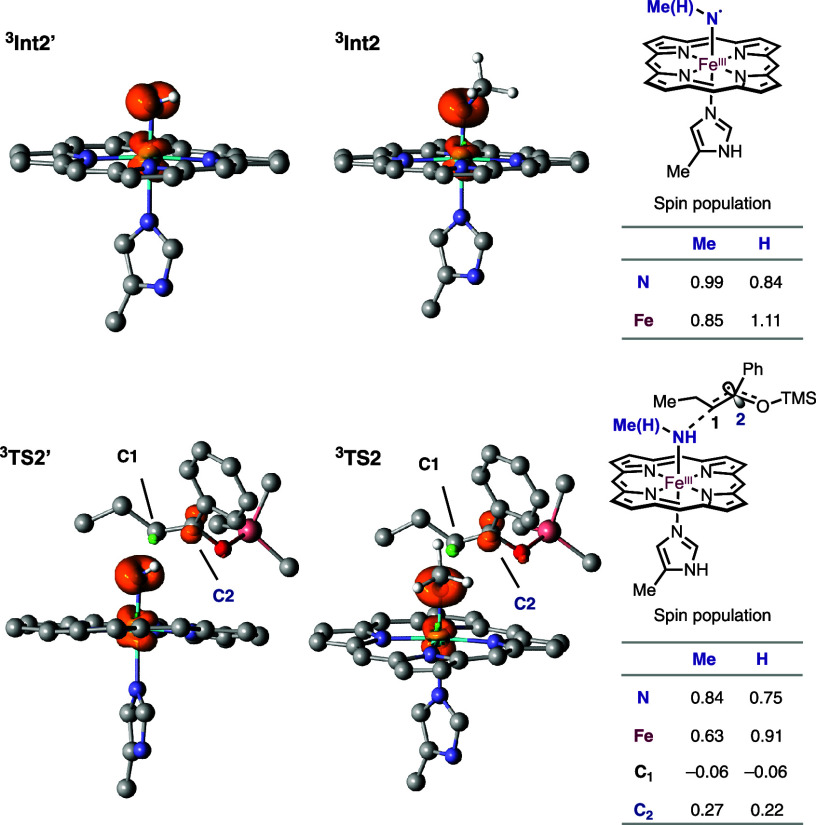
**Computational investigation of mechanisms.** DFT calculations were carried out at (U)­B3LYP-D3­(BJ)/DEF2TZVP/CPCM­(Et_2_O)//(U)­B3LYP-D3­(BJ)/6–31G*-SDD­(Fe)/CPCM­(Et_2_O) level of theory. Hirshfeld spin density and population for Int2, TS2, and corresponding unsubstituted nitrene species Int2′ and TS2′. The isovalue is 0.02 for all cases. Nonessential hydrogen atoms are omitted for clarity.

In summary, we have established a protoglobin platform, exemplified by the engineered variants *pa* **Pgb-AKS-(** *S* **)-G3-5332** and *pa* **Pgb-AKS-(** *R* **)-G3-5335**, capable of catalyzing unprecedented stereoconvergent, intermolecular alkylamination of isomeric silyl enol ethers to furnish valuable α-aminoketones with high activity and selectivity. This biocatalytic system harnesses readily accessible and environmentally benign *N*-alkyl hydroxylamines as nitrene precursors, which has remained elusive in both small-molecule and enzymatic catalysis. Notably, this strategy diverges from the conventional stereospecific paradigm of biocatalytic alkene functionalization by enabling stereoconvergent access to enantiopure products from alkene mixtures. Furthermore, enantiodivergent variants were readily accessed through a few mutations, enabling selective synthesis of either enantiomer. We anticipate these findings will expand the enzymatic repertoire of engineered protoglobins and offer a versatile platform for stereoconvergent enzymatic transformations, paving the way for new biocatalytic strategies and molecular scaffolds.

## Supplementary Material





## Notes

MestReNova files of substrates and products (ZIP)
Materials and methods, experimental procedures, copies of NMR spectra (PDF)
