# Supplementary material for: Engineering Enantiocomplementary Protoglobins for Stereoconvergent Construction of N‑Alkylated α‑Aminoketones
Source: J Am Chem Soc. 2026 May 1;148(18):18555–61. doi: 10.1021/jacs.5c17989 (PMC13185117; doi:10.1021/jacs.5c17989)
Supplement: Supplementary file 2 [file ja5c17989_si_002.pdf]

Supporting Information

Engineering Enantiocomplementary Protoglobins for Stereoconvergent Construction of *N*-Alkylated α-Aminoketones

Zi-Yang Qin,[†,§] Zi-Qi Li,[†,§] Chi Zhang,[‡,§] Jan L. Heise,[§,#] Runze Mao,[§,||], Sophia J. Wu[§], K. N. Houk,[¶] William A. Goddard III,[‡] Frances H. Arnold*[,§]

[§]Division of Chemistry and Chemical Engineering, California Institute of Technology, Pasadena, California 91125, USA.

[‡]Materials and Process Simulation Center, California Institute of Technology, Pasadena, California 91125, USA.

[¶]Department of Chemsitry and Biochemistry, University of California, Los Angeles, Los Angeles, California 90095, USA.

[#]Current address: Institute of Theoretical and Computational Chemistry, Heinrich Heine University Düsseldorf, Düsseldorf, 40225, Germany.

[||]Current address: Tsinghua Shenzhen International Graduate School, Shenzhen, Guangdong, 518055, China.

[†]These authors contributed equally.

*To whom correspondence should be addressed. E-mail: frances@cheme.caltech.edu

Table of Contents

Engineering Convergent Protoglobins for Enantiodivergent Construction of α-Aminoketones ... 1
I. General Procedures ... 2
II. Directed Evolution of *pa*Pgb-AKS-(*S*)-5332 and *pa*Pgb-AKS-(*R*)-5335 for Intermolecular, Stereoconvergent Amination ... 6
III. Supporting Experimental Figures and Miscellaneous Experiments ... 8
IV. Preparation and Characterization of the Silyl Enol Ether Substrates ... 11
V. Preparation and Characterization of α-Amino Ketone Standard Products ... 15
VI. Analytic-Scale Enzymatic Reactions and Calibration Curves for Products ... 20
VII. Preparative-Scale Enzymatic Synthesis ... 38
VIII. Chiral Polar Protic HPLC Traces ... 40
IX. Sequence Information ... 75
X. Computational Studies ... 79
XI. NMR Spectra ... 98
XII. References ... 114

I. General Procedures

(A) General

Unless otherwise noted, all chemicals and reagents were obtained from commercial suppliers (Sigma-Aldrich, VWR, TCI America, Fischer Scientific, Alfa Aesar, Acros, and Combi Blocks) and used without further purification. Erlenmeyer flasks used for cell-culture and protein expression were purchased from VWR and autoclaved before use. Glassware used for analytical reaction set-up and HPLC analysis were purchased from Agilent and used as received. All solvents were purchased from MilliporeSigma, VWR, Thermofisher and used as received. Silica gel chromatography was carried out using Biotage® Sfär chromatography column. ^{1}H and ^{13}C NMR spectra were recorded on a Bruker Prodigy 400 MHz instrument (400 MHz for ^{1}H and 101 MHz for ^{13}C NMR, and 376 MHz for ^{19}F NMR). Chemical shifts (δ) are reported in ppm downfield from tetramethylsilane, using the solvent resonance as the internal standard (^{1}H NMR: δ = 7.26, ^{13}C NMR: δ = 77.16 for $CDCl_3$; ^{1}H NMR: δ 4.79 for D_2O). Data for ^{1}H NMR are reported as follows: chemical shift (δ ppm), multiplicity (s = singlet, d = doublet, t = triplet, q = quartet, p = pentet, sext = sextet, m = multiplet, dd = doublet of doublets, dt = doublet of triplets, ddd = doublet of doublet of doublets), coupling constant (Hz), integration. Sonication was performed using a Qsonica Q500 sonicator. High-resolution mass spectra were obtained at the California Institute of Technology Mass Spectral Facility. Samples were analyzed by field ionization (FI) using a JEOL AccuTOF GC-Alpha (JMS-T2000GC) mass spectrometer interfaced with an Agilent 8890 GC system. Ions detected by FI are radical cations.

Chemical reactions were monitored using thin layer chromatography (Merck 60 silica gel plates) and a UV lamp for visualization. Analytical reverse-phase high-performance liquid chromatography-mass spectroscopy (HPLC-MS) was carried out using an Agilent 1260 series instrument and an Agilent C18 column (InfinityLab Poroshell 120 EC-C18, 4.6 x 50 mm, 2.7 µm; Part Number: 699975-902T) with water and acetonitrile, both containing 0.1% acetic acid, as the mobile phase. Analytical chiral polar protic HPLC was conducted using an Agilent 1260 series instrument with ethanol and water (pH 4.0, with 20 mM ammonium formate). Enantiomers were separated using an Angilent Poroshell 120 Chiral-V column, 2.7 µm, 2.1 × 150 mm. Separation of stereoisomer (*E*)- and (*Z*)-**1a** was conducted using a JASCO SF-2000 integrated analytical supercritical fluid chromatography (SFC) system with supercritical CO_2 and 5% of 25% isopropanol in hexanes as the mobile phase on a Chiralpak AD-H preparative column.

Escherichia coli cells were grown using Luria-Bertani medium or Terrific Broth with 100 µg/mL ampicillin (LB_{amp} or TB_{amp}). Primer sequences are available upon request. Phusion polymerase and *Taq* ligase were purchased from New England Biolabs (NEB, Ipswich, MA). M9-N minimal media (abbreviated as M9-N buffer, pH = 8.0) were used as buffering systems for whole cells unless otherwise specified. M9-N buffer was used without a carbon source; it contains 47.7 mM Na_2HPO_4, 22.0 mM KH_2PO_4, 8.6 mM NaCl, 2.0 mM $MgSO_4$, and 0.1 mM $CaCl_2$. All substrates and standard products were synthesized as described in **Section IV** and **V**.

(B) Cloning, Mutagenesis, and Expression of Enzymes

The genes encoding all enzymes described in this study were cloned using Gibson assembly[1] into vector pET22b(+) (Novagen) between restriction sites *Nde*I and *Xho*I in frame with a *C*-terminal 6×His-tag. Site-saturation mutagenesis was performed using the "22c-trick" as degenerative codons.[2] The PCR products were digested with *Dpn*I, gel purified, and ligated using Gibson Mix™. Without further purification after the Gibson step, 1 µL of the Gibson product was used to transform 25 µL of electrocompetent *E. coli* BL21 E. cloni® (Lucigen) cells.

Table S1. Primers used in site-saturation mutagenesis

Primers	Sequences (5' → 3')
Universal Forward (UniF)	CCA ACT TAC TTC TGA CAA CGA TCG GAG GAC CGA AGG AGC TAA CCG CTT TTT TGC
Universal Reverse (UniR)	CGA TCG TTG TCA GAA GTA AGT TGG CCG CAG TGT TAT CAC TCA TGG TTA TGG CAG

Random mutations were introduced using error-prone PCR with the addition of 300–600 µM $MnCl_2$ to a Taq PCR method as previously reported using the primers described below[3]:

Table S2. Primers used in error-prone PCR

Primers	Sequences (5' → 3')
005	GAA ATA ATT TTG TTT AAC TTT AAG AAG GAG ATA TAC ATA TG
006	GCC GGA TCT CAG TGG TGG TGG TGG TGG TGC TCG AG
007	CAT ATG TAT ATC TCC TTC TTA AAG TTA AAC AAA ATT ATT TC
008	CTC GAG CAC CAC CAC CAC CAC CAC TGA GAT CCG GC

Primers 005 and 006 were used to amplify the region coding for the enzyme using Taq polymerase and the parent DNA template. Mutations within the PCR fragment were introduced by varying the concentration of $MnCl_2$ during PCR amplification. The backbone (i.e., pET22b(+) vector) fragment was amplified following the SSM protocol using Primers 007 and 008. The excess plasmid DNA templates were digested with *Dpn*I, and the amplified fragments were purified by gel electrophoresis (1–2% agarose gel). Both amplified fragments were then assembled into a circular plasmid using the Gibson assembly.[2] Libraries generated with 300, 400, 500, and 600 µM $MnCl_2$ were test screened (one 96-well plate each) to determine their diversity, activity, and selectivity.

Staggered extension process (StEP) PCR was conducted by pooling plasmids of the variants to be recombined in equimolar fashion and using this mixture as the template DNA.[3-4] This method recombines multiple hits obtained from SSM and error-prone PCR libraries with high efficiency by performing special PCR cycles, where the elongation step is disrupted earlier by denaturation. The procedure has been designed to always require two PCRs: one to recombine the sequences as efficiently as possible and another to amplify the recombination product from the first PCR to create a useful amount of product. Primers 005 and 006 were used to amplify the region coding for the enzyme using Taq polymerase and the parent DNA template. After examination by gel electrophoresis (1–2% agarose gel), the successful recombination fragment with the lowest reaction temperature was chosen for the construction of the library. The amplified fragments were then assembled into a circular plasmid using the Gibson assembly procedure.

E. coli (E. cloni BL21(DE3)) cells carrying plasmids encoding the appropriate protoglobin variant were grown overnight for 14–16 hours in 5-mL Luria-Bertani medium supplemented with 0.1 mg/mL ampicillin (LB_{amp}). The preculture (1 mL) was used to inoculate 50 mL of TerrificBroth medium supplemented with 0.1 mg/mL ampicillin (TB_{amp}) in a 125-mL Erlenmeyer flask. This culture was incubated at 37 ºC and 250 rpm for 2–2.5 hours until an optical density at 600 nm (OD_{600}) of 0.8–1.0 was reached. The culture was then cooled on ice for 45 min and induced with 0.5 mM IPTG and 1.0 mM ALA (final concentrations). Expression was conducted at 22 ºC and 180 rpm for 20–22 hours. Subsequently, the *E. coli* cells were pelleted by centrifugation (4,000 g, 8 min, and 4 ºC). Media were removed, and the pellets were resuspended to an OD_{600} of 38 in M9-N minimal medium with the pH adjusted to 8.0. The cell suspension was used to determine protein concentration after lysis by sonication.

(C) Hemochrome Assay for the Determination of Hemoprotein Concentration

Protein concentration in the cells was determined using the hemochrome assay in cell lysate.[3] Lysate was obtained by sonication using a Qsonica Q500 sonicator (6 minutes total sonication time, 1 second on, 2 seconds off, 35% amplitude, on wet ice). The cell debris was removed by centrifugation (14,000 g, 10 minutes, and 4 °C). To a cuvette, 500 μL of the lysate and 500 μL of solution I [0.2 M NaOH, 40% (v/v) pyridine, 0.5 mM $K_3Fe(CN)_6$] were added. The UV-Vis spectrum (380–620 nm) of the oxidized state Fe(III) was recorded immediately. Sodium dithionite (10 μL of 0.5 M solution in water) was added, and the UV-Vis spectrum of the reduced state Fe(II) was recorded immediately. The protein concentration was calculated using the extinction coefficient and dilution factor (2× dilution in volume): ε_[$557_{reduced} - 540_{oxidized}$] = 23.98 $mM^{-1}cm^{-1}$. The hemochrome assay detects total heme level, which is a good approximation of over-expressed heme enzyme.

(D) Analytic Reaction Setup and Product Quantification

All biocatalytic reactions were set up in an anaerobic chamber (oxygen level: <60 ppm). Harvested cells were resuspended with M9-N buffer (pH 8.0, containing 25 mM D-glucose) to OD_{600} = 38, and 340 μL of resuspended cells were aliquoted to 2-mL screw cap vials. Unless otherwise specified, 20 μL of the 500 mM glucose solution were added to make the total volume to 360 μL. The resulting mixtures were then added the hydrocarbon substrate **1** (20 μL, 0.1 M stock in ethanol) and the nitrene precursor **2d** (20 μL, 0.2 M stock in M9-N buffer) in a sequential

manner. Unless otherwise noted, the reactions were then shaken at room temperature for overnight at 900 rpm.

After the reaction was completed and the vials removed from the shaker, an internal standard (800 μL ethanol containing 5 mM 1,2,3-trimethoxybenzene) was added. The mixture was transferred to a 2.0-mL Eppendorf tube and then subjected to vortexing (20 s × 2) and centrifugation (14,000 × g, 10 min, 4 °C). A sample of the supernatant (0.2 mL) was transferred to a vial with an insert for reverse-phase HPLC-MS analysis. Products were identified and quantified based on the corresponding reference compounds (**Section V**).

To further determine the enantiomeric excess (*ee*), the supernatants of these parallel analytical reactions were combined and transferred to a 2-dram vial for reverse-phase HPLC-MS analysis.

(E) Reaction Screening in 96-Well Plate in Whole-Cell Format

Single colonies from LB_{amp} agar plates were picked using sterile toothpicks and shaken in deep-well 96-well plates containing LB_{amp} (500 μL/well) at 37 ºC, 80% humidity, and 220 rpm overnight. Subsequently, TB_{amp} (950 μL/well) in a deep-well plate was inoculated with an aliquot (50 μL/well) of these overnight cultures and allowed to shake for 2.5 hours at 37 ºC, 80% humidity, and 220 rpm. The plates were then cooled on ice for 30 minutes, and the cultures were induced with 0.5 mM isopropyl *β*-D-1-thiogalactopyranoside (IPTG) and 1.0 mM 5-aminolevulinic acid (ALA) (final concentrations). Expression was then conducted at 20 ºC, and 220 rpm for 20–22 hours.

II. Directed Evolution of *pa*Pgb-AKS-(*S*)-5332 and *pa*Pgb-AKS-(*R*)-5335 for Intermolecular, Stereoconvergent Amination

(A) Discovery of Initial Activity

The Arnold lab collection of protoglobins was screened in 96-well-plate whole-cell reactions for product formation (see **Experimental Methods**). The collection consisted of nearly 200 distinct variants accumulated from prior directed evolution campaigns. One variant from a previous nitrene transferring lineage showed the highest product formation as assayed by LCMS. No product was detected in control reactions with free heme.

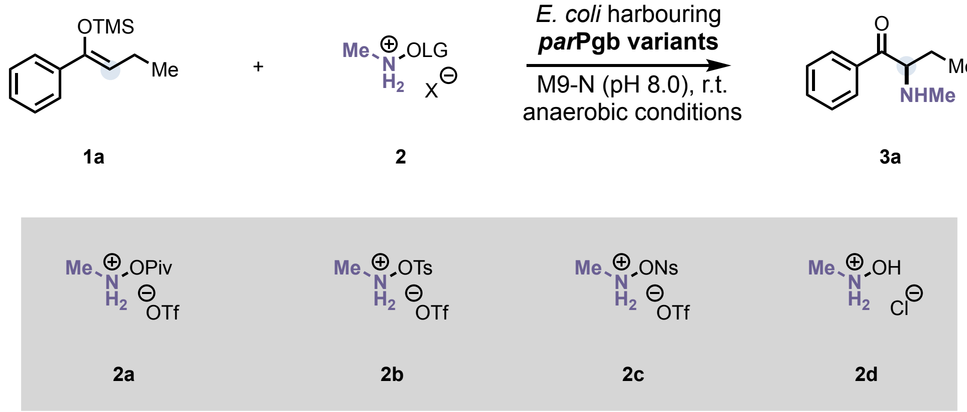

(B) Directed Evolution of AminoKetone Synthase (AKS)

Table S3. Detailed information of the evolutionary lineage for **A**mino**K**etone **S**ynthase (**AKS**).

***pa*Pgb-AKS** variant	**Yield (%)**	***ee***	**EC (μM)**
pa*Pgb-B5** (pa*Pgb-AKS-5329**)	2.8 ± 0.02	–73%	4.4
pa*Pgb-AKS-5329**- D51N Q60A V85G I133V (pa*Pgb-AKS-5330**)	5.7 ± 0.01	60%	6.3
pa*Pgb-AKS-5330**- L55P I112Q I152N H158Y (pa*Pgb-AKS-5331**)	13 ± 0.8	91%	3.4
pa*Pgb-AKS-5331**- M109T G157R (pa*Pgb-AKS-5332**)	22 ± 3.2	94%	7.9
pa*Pgb-AKS-5329**- D57E R86S P151Q (pa*Pgb-AKS-5333**)	7.7 ± 1.1	–89%	4.1
pa*Pgb-AKS-5333**- L55S S156W (pa*Pgb-AKS-5334**)	10 ± 0.2	–92%	7.1
pa*Pgb-AKS-5334**- P160L A186E (pa*Pgb-AKS-5335**)	25 ± 2.3	–92%	9.2

Note: enzyme concentration (EC) was determined using hemochrome assay in **Section I**, **C**.

Table S4. Investigation of enantioselectivity inversion for ***pa*Pgb-AKS**.

***pa*Pgb-AKS** variant	**Yield (%)**	***ee***

***pa*Pgb-B5 (*pa*Pgb-AKS-5329)**	2.8 ± 0.02	–73%
pa*Pgb-AKS-5329**- D51N Q60A V85G I133V (pa*Pgb-AKS-5330**)	5.8 ± 0.01	60%
***pa*Pgb-AKS-5329**- D51N	3.2 ± 0.22	–64%
***pa*Pgb-AKS-5329**- Q60A	6.0 ± 0.14	–72%
***pa*Pgb-AKS-5329**- V85G	4.8 ± 0.05	49%
***pa*Pgb-AKS-5329**- I133V	4.5 ± 0.05	–72%

III. Supporting Experimental Figures and Miscellaneous Experiments

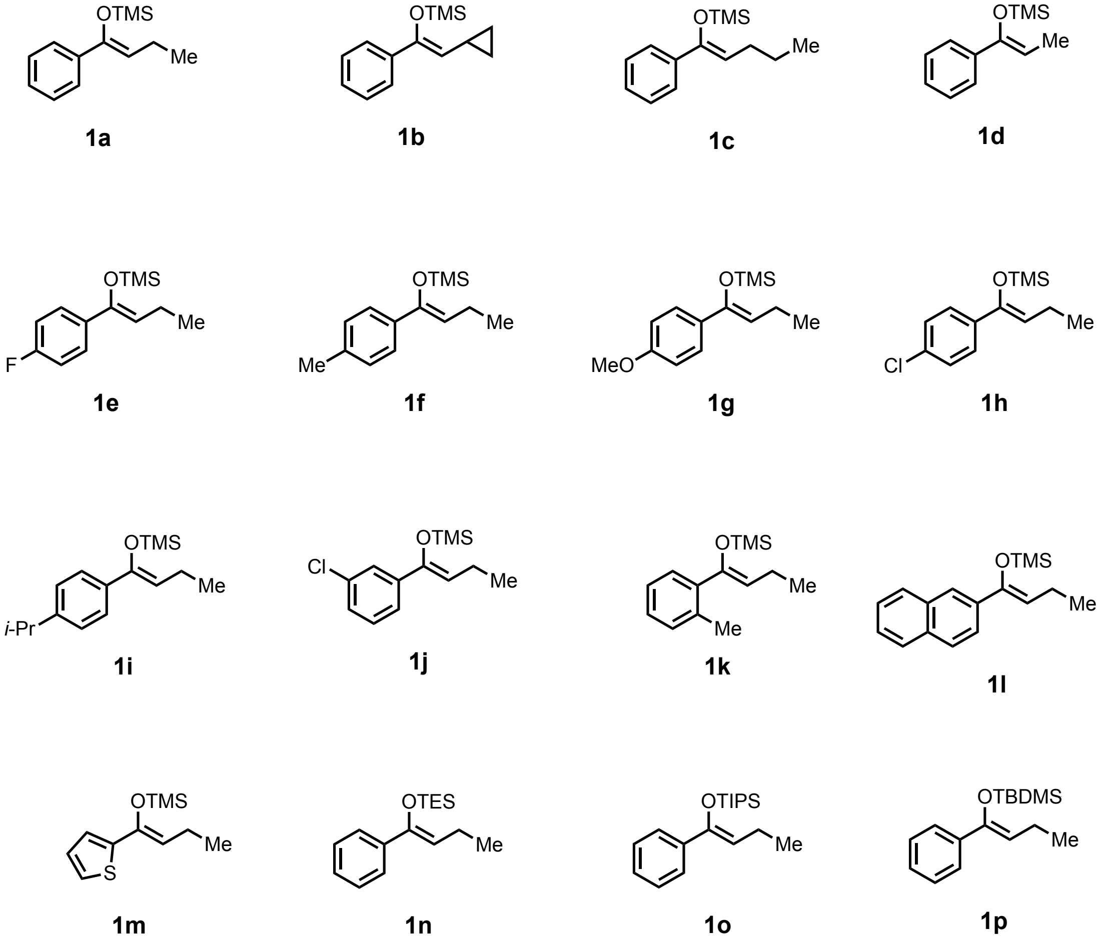

Figure S1. Summary of silyl enol ether substrates (**1a**–**1p**).

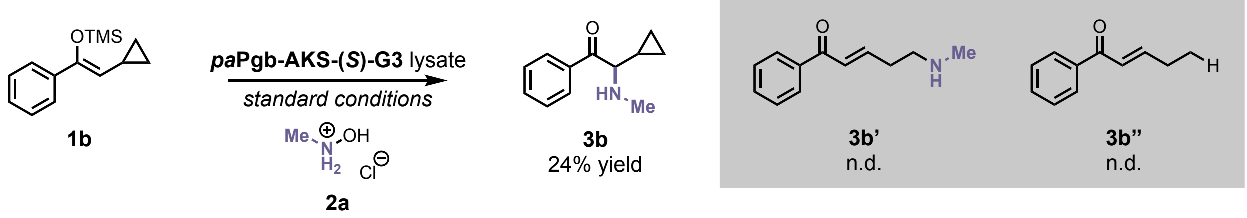

Figure S2. Radical clock experiments using ***pa*Pgb-AKS-(S)-G3-5332** with **1b**.

Table S5. Assessment of whole-cell, lysate, and purified protein reaction formats for variant ***pa*****Pgb-AKS-(*****S*****)-G3-5332**.

pa**Pgb-AKS-(*****S*****)-G3-5332** reaction formats	**Yield (%)**	***ee***
Whole cells	22 ± 3.2	94%
lysate	59 ± 1.8	92%
purified protein	34 ± 2.2	92%
lysate with additional 2.0 equiv. of *N*-methylhydroxylamine (**2d**)	77 ± 4.3	90%

Table S6. Assessment of whole-cell, lysate, and purified protein reaction formats for variant ***pa*****Pgb-AKS-(*****R*****)-G3-5335**.

pa**Pgb-AKS-(*****R*****)-G3-5335** reaction formats	**Yield (%)**	***ee***
Whole cells	25 ± 2.3	–92%
lysate	41 ± 0.6	–93%
purified protein	47 ± 1.9	–93%
lysate format with additional 2.0 equiv. of *N*-methylhydroxylamine (**2d**)	70 ± 0.2	–92%

Scheme S1. Kinetic studies using enantiopure (*Z*)-**1a** and (*E*)-**1a** with ***pa*****Pgb-AKS-(*****S*****)-G3-5332** and ***pa*****Pgb-AKS-(*****R*****)-G3-5335**.

The kinetic experiments were conducted with lower catalyst loading (OD_{600} = 10 instead of OD_{600} = 30) to reduce the overall reaction rate. The results confirmed that there is no interconversion of the isomers. Moreover, the results showed that the reaction rates for both **(*****Z*****)-1a** and **(*****E*****)-1a** substrate are similar. For ***pa*****Pgb-AKS-(*****S*****)-G3-5332**, $v_Z/v_E = 0.68$; For ***pa*****Pgb-AKS-(*****R*****)-G3-5335**, $v_Z/v_E = 1.17$. The small differences in reaction rate contributed to the overall observed stereoconvergence.

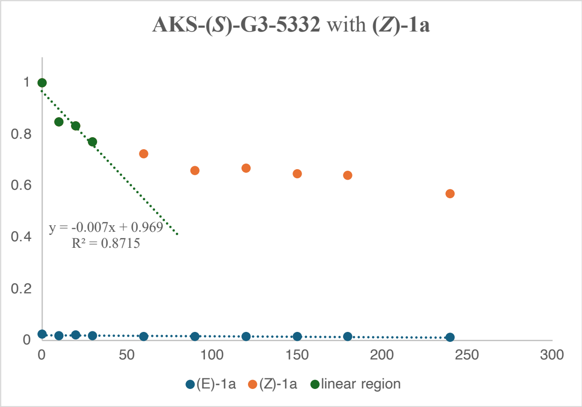
AKS-(S)-G3-5332 with (Z)-1a
y = -0.007x + 0.969
R² = 0.8715
(E)-1a
(Z)-1a
linear region

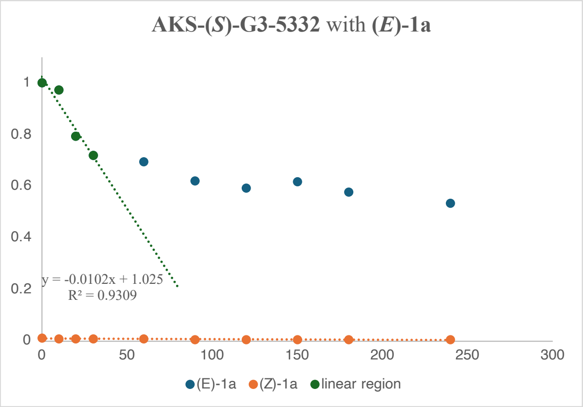
AKS-(S)-G3-5332 with (E)-1a
y = -0.0102x + 1.025
R² = 0.9309
(E)-1a
(Z)-1a
linear region

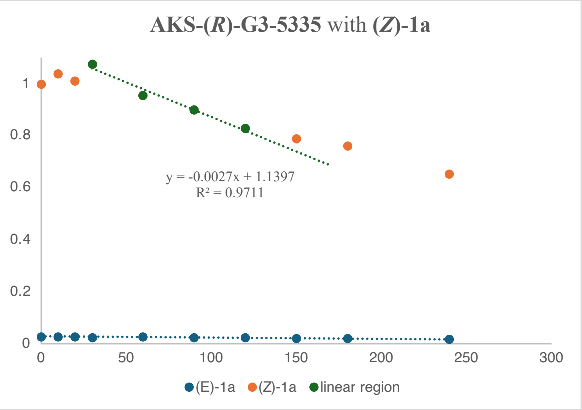
AKS-(R)-G3-5335 with (Z)-1a
y = -0.0027x + 1.1397
R² = 0.9711
(E)-1a
(Z)-1a
linear region

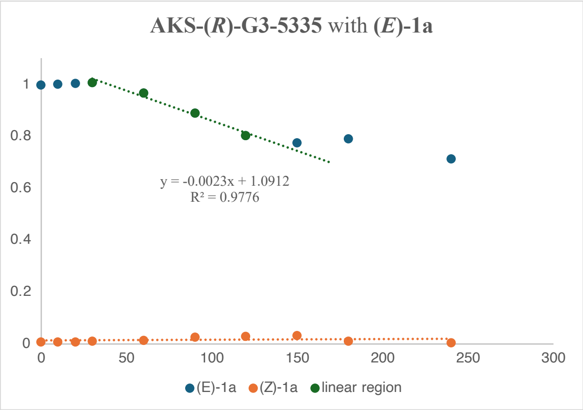
AKS-(R)-G3-5335 with (E)-1a
y = -0.0023x + 1.0912
R² = 0.9776
(E)-1a
(Z)-1a
linear region

IV. Preparation and Characterization of the Silyl Enol Ether Substrates

General procedure A:

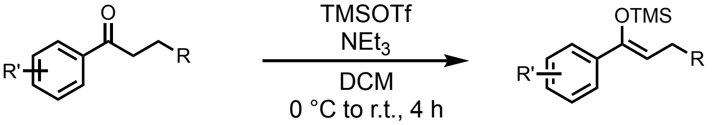

To an oven-dried 40-mL vial were added the corresponding ketone (10.0 mmol, 1.0 equiv.) and anhydrous DCM (20 mL, 0.5 M). The resulting mixture was cooled in an ice bath. Triethylamine (15.0 mmol, 1.5 equiv., 2.1 mL) and TMSOTf (11.0 mmol, 1.1 equiv., 2.0 mL) were added sequentially. The mixture was stirred for four hours at this temperature and poured into water/DCM in an extraction funnel. The product was extracted by DCM (20 mL × 3), and the combined organic layer was dried over Na_2SO_4 and concentrated to afford a transparent to yellow oil. Flash column chromatography (5% ethyl acetate in hexanes as eluent) was performed to afford pure product in 60% to 90% yield.

General procedure B:

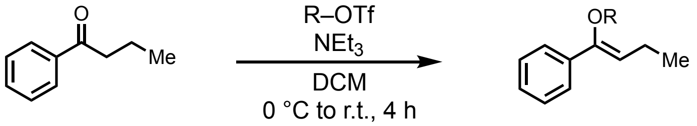

To an oven-dried 40-mL vial were added butyrophenone (10.0 mmol, 1.0 equiv.) and anhydrous DCM (20 mL, 0.5 M). The resulting mixture was cooled in an ice bath. Triethylamine (15.0 mmol, 1.5 equiv., 2.1 mL) and R–OTf (11.0 mmol, 1.1 equiv.) were added sequentially. The mixture was stirred for four hours at this temperature and poured into water/DCM in an extraction funnel. The product was extracted by DCM (20 mL × 3), and the combined organic layer was dried over Na_2SO_4 and concentrated to afford a transparent to yellow oil. Flash column chromatography (5% ethyl acetate in hexanes as eluent) was performed to afford pure product in 20% to 50% yield.

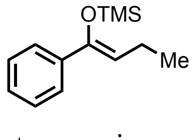

Trimethyl((1-phenylbut-1-en-1-yl)oxy)silane (1a): The reaction was carried out following General Procedure A using 1-phenylbutan-1-one (10.0 mmol, 1.0 equiv), triethylamine (15.0 mmol, 1.5 equiv, 2.1 mL), and TMSOTf (11.0 mmol, 1.1 equiv, 2.0 mL). **1a** was obtained as a colorless liquid (93:7 *er*). Analytical data were in agreement with literature values[1]. **^{1}H NMR** (400 MHz, Acetone) δ 7.5–7.51 (m, 2H), 7.39–7.33 (m, 2H), 7.31–7.26 (m, 1H), 5.33 (t, J = 7.1 Hz, 1H), 2.25 (p, J = 7.4 Hz, 2H), 1.07 (t, J = 7.5 Hz, 3H), 0.16 (s, 9H).

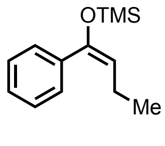

(*E*)-trimethyl((1-phenylbut-1-en-1-yl)oxy)silane ((*E*)-1a): The reaction was carried out following a known procedure with slight modifications. To a solution of trimethyl((1-phenylbut-1-en-1-yl)oxy)silane (5.0 mmol, 1.0 equiv) in acetonitrile (5.0 mL) was added Ir(ppy)$_3$ (0.05 mmol, 1 mol%), and

diisopropylethylamine (1 drop). The reaction mixture was degassed by sparging nitrogen gas for 10 min and then left under positive nitrogen pressure by removing the exit needle. The vial was placed in a light bath (Blue LEDs were purchased from Kessil Co., Ltd. (40 W max., product No. A160WE)) for 16 hours. The crude product was purified by normal phase chromatography. The Z/E ratio of **1a** (1:1.6) was determined by ^{1}H NMR. Separation of stereoisomer (*Z*)- and (*E*)-**1a** was conducted using a JASCO SF-2000 integrated analytical supercritical fluid chromatography (SFC) system with supercritical CO_2 and 5% of 25% isopropanol in hexanes as the mobile phase on a Chiralpak AD-H preparative column. Analytical data were in agreement with literature values[1]. **^{1}H NMR** (400 MHz, $CDCl_3$) δ 7.39–7.19 (m, 8H), 5.02 (t, J = 7.6 Hz, 1H), 2.11 (p, J = 7.5 Hz, 2H), 0.99 (t, J = 7.4 Hz, 3H), 0.13 (s, 9H).

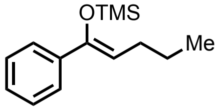

Trimethyl((1-phenylpent-1-en-1-yl)oxy)silane (1c): The reaction was carried out following General Procedure A using 1-phenylpentan-1-one (10.0 mmol, 1.0 equiv), triethylamine (15.0 mmol, 1.5 equiv, 2.1 mL), and TMSOTf (11.0 mmol, 1.1 equiv, 2.0 mL). **1i** was obtained as a colorless liquid (96:4 *er*). Analytical data were in agreement with literature values[1]. ^{1}H NMR (400 MHz, $CDCl_3$) δ 7.51–7.40 (m, 2H), 7.37–7.20 (m, 4H), 5.26 (t, J = 7.2 Hz, 1H), 2.18 (td, J = 7.8, 6.5 Hz, 2H), 1.53–1.39 (m, 2H), 0.97 (t, J = 7.4 Hz, 3H), 0.14 (s, 9H).

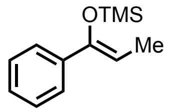

Trimethyl((1-phenylprop-1-en-1-yl)oxy)silane (1d): The reaction was carried out following General Procedure A using propiophenone (10.0 mmol, 1.0 equiv), triethylamine (15.0 mmol, 1.5 equiv, 2.1 mL), and TMSOTf (11.0 mmol, 1.1 equiv, 2.0 mL). **1j** was obtained as a colorless liquid (98:2 *er*). Analytical data were in agreement with literature values[1]. ^{1}H NMR (400 MHz, $CDCl_3$) δ 7.49–7.40 (m, 2H), 7.34– .20 (m, 3H), 5.38–5.26 (m, 1H), 1.74 (d, J = 7.0 Hz, 3H), 0.14 (s, 9H).

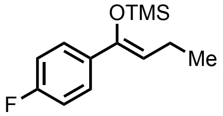

((1-(4-Fluorophenyl)but-1-en-1-yl)oxy)trimethylsilane (1e): The reaction was carried out following General Procedure A using 1-(4-fluorophenyl)butan-1-one (10.0 mmol, 1.0 equiv), triethylamine (15.0 mmol, 1.5 equiv, 2.1 mL), and TMSOTf (11.0 mmol, 1.1 equiv, 2.0 mL). **1b** was obtained as a colorless liquid (73:27 *er*). Analytical data were in agreement with literature values[1]. **^{1}H NMR** (400 MHz, Acetone) δ 7.62–7.53 (m, 2H), 7.31 (t, J = 8.8 Hz, 1H), 7.12 (t, J = 8.9 Hz, 1H), 5.29 (t, J = 7.1 Hz, 1H), 2.29–2.19 (m, 2H), 1.06 (t, J = 7.5 Hz, 3H), 0.16 (s, 9H).

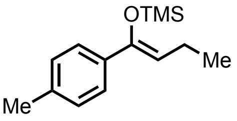

Trimethyl((1-(*p*-tolyl)but-1-en-1-yl)oxy)silane (1f): The reaction was carried out following General Procedure A using 1-(*p*-tolyl)butan-1-one (10.0 mmol, 1.0 equiv), triethylamine (15.0 mmol, 1.5 equiv, 2.1 mL), and TMSOTf (11.0 mmol, 1.1 equiv, 2.0 mL). **1c** was obtained as a colorless liquid (99:1 *er*). Analytical data were in agreement with literature values[1]. **^{1}H NMR** (400 MHz, $CDCl_3$) δ 7.36 (d, J = 8.0 Hz, 2H), 7.10 (d, J = 8.0 Hz, 2H), 5.19 (t, J = 7.1 Hz, 1H), 2.33 (s, 3H), 2.20 (p, J = 7.4 Hz, 2H), 1.03 (t, J = 7.5 Hz, 3H), 0.13 (s, 9H).

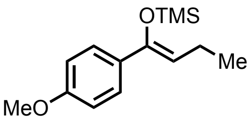

((1-(4-Methoxyphenyl)but-1-en-1-yl)oxy)trimethylsilane (1g): The reaction was carried out following General Procedure A using 1-(4-methoxyphenyl)butan-1-one (10.0 mmol, 1.0 equiv), triethylamine (15.0 mmol, 1.5 equiv, 2.1 mL), and TMSOTf (11.0 mmol, 1.1 equiv, 2.0 mL). **1d** was obtained as a colorless liquid (96:4 *er*). Analytical data were in agreement with literature

values[1]. **^{1}H NMR** (400 MHz, Acetone) δ 7.44 (d, *J* = 8.8 Hz, 2H), 6.91 (d, *J* = 8.8 Hz, 2H), 5.18 (t, *J* = 7.1 Hz, 1H), 3.82 (s, 3H), 2.22 (p, *J* = 7.4 Hz, 2H), 1.04 (t, *J* = 7.5 Hz, 2H), 0.15 (s, 9H).

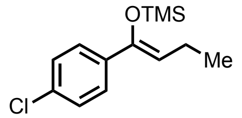

((1-(4-Chlorophenyl)but-1-en-1-yl)oxy)trimethylsilane (1h): The reaction was carried out following General Procedure A using 1-(4-chlorophenyl)butan-1-one (10.0 mmol, 1.0 equiv), triethylamine (15.0 mmol, 1.5 equiv, 2.1 mL), and TMSOTf (11.0 mmol, 1.1 equiv, 2.0 mL). **1e** was obtained as a colorless liquid (95:5 *er*). Analytical data were in agreement with literature values[1]. **^{1}H NMR** (400 MHz, $CDCl_3$) δ 7.41–7.37 (m, 2H), 7.28–7.23 (m, 3H), 5.22 (t, *J* = 7.1 Hz, 1H), 2.20 (p, *J* = 7.5 Hz, 2H), 1.03 (t, *J* = 7.5 Hz, 3H), 0.13 (s, 8H).

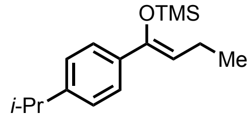

((1-(4-Isopropylphenyl)but-1-en-1-yl)oxy)trimethylsilane (1i): The reaction was carried out following General Procedure A using 1-(4-isopropylphenyl)butan-1-one (10.0 mmol, 1.0 equiv), triethylamine (15.0 mmol, 1.5 equiv, 2.1 mL), and TMSOTf (11.0 mmol, 1.1 equiv, 2.0 mL). **1f** was obtained as a colorless liquid (96:4 *er*). Analytical data were in agreement with literature values[1]. **^{1}H NMR** (400 MHz, Acetone) δ 7.45 (d, *J* = 8.3 Hz, 2H), 7.23 (d, *J* = 8.3 Hz, 2H), 5.28 (t, *J* = 7.1 Hz, 1H), 2.97–2.86 (m, 1H), 2.23 (p, *J* = 7.5 Hz, 2H), 1.26 (d, *J* = 6.9 Hz, 6H), 1.05 (t, *J* = 7.5 Hz, 3H), 0.16 (s, 9H).

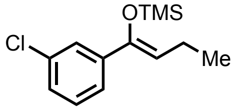

((1-(3-Chlorophenyl)but-1-en-1-yl)oxy)trimethylsilane (1j): The reaction was carried out following General Procedure A using 1-(3-chlorophenyl)butan-1-one (10.0 mmol, 1.0 equiv), triethylamine (15.0 mmol, 1.5 equiv, 2.1 mL), and TMSOTf (11.0 mmol, 1.1 equiv, 2.0 mL). **1g** was obtained as a colorless liquid (96:4 *er*). Analytical data were in agreement with literature values[1]. **^{1}H NMR** (400 MHz, Acetone) δ 7.56–7.47 (m, 2H), 7.39 (t, *J* = 7.8 Hz, 1H), 7.35–7.30 (m, 1H), 5.44 (t, *J* = 7.2 Hz, 1H), 2.26 (p, *J* = 7.5 Hz, 2H), 1.07 (t, *J* = 7.5 Hz, 3H), 0.19 (s, 6H).

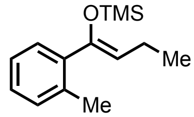

Trimethyl((1-(*o*-tolyl)but-1-en-1-yl)oxy)silane (1k): The reaction was carried out following General Procedure A using 1-(*o*-tolyl)butan-1-one (10.0 mmol, 1.0 equiv), triethylamine (15.0 mmol, 1.5 equiv, 2.1 mL), and TMSOTf (11.0 mmol, 1.1 equiv, 2.0 mL). **1h** was obtained as a colorless liquid with a Z/E ratio of 34:66. Analytical data were in agreement with literature values[1]. **1h:** ^{1}H NMR (400 MHz, Acetone) δ 7.30–7.07 (m, 4H), 5.07 (t, *J* = 7.7 Hz, 1H), 2.34 (s, 3H), 1.81 (p, *J* = 7.5 Hz, 2H), 0.93 (t, *J* = 7.4 Hz, 3H), 0.12 (s, 9H). **(*E*)-1h:** ^{1}H NMR (400 MHz, Acetone) δ 7.32–7.11 (m, 4H), 4.80 (t, *J* = 7.1 Hz, 1H), 2.38 (s, 3H), 2.29–2.19 (m, 2H), 1.06 (t, *J* = 7.5 Hz, 3H), 0.02 (s, 9H).

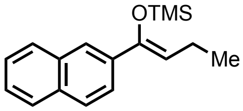

Trimethyl((1-(naphthalen-2-yl)but-1-en-1-yl)oxy)silane (1l): The reaction was carried out following General Procedure A using 1-(naphthalen-2-yl)butan-1-one (10.0 mmol, 1.0 equiv), triethylamine (15.0 mmol, 1.5 equiv, 2.1 mL), and TMSOTf (11.0 mmol, 1.1 equiv, 2.0 mL). **1k** was obtained as a colorless liquid (99:1 *er*). Analytical data were in agreement with literature values[1]. ^{1}H NMR (400 MHz, Acetone) δ 8.04–7.99 (m, 1H), 7.97–7.85 (m, 3H), 7.76–7.69 (m, 1H), 7.59–7.47 (m, 2H), 5.53 (t, *J* = 7.1 Hz, 1H), 2.32 (p, *J* = 7.5 Hz, 2H), 1.11 (t, *J* = 7.5 Hz, 3H), 0.21 (s, 8H).

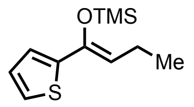

Trimethyl((1-(thiophen-2-yl)but-1-en-1-yl)oxy)silane (1m): The reaction was carried out following General Procedure A using 1-(thiophen-2-yl)butan-1-one (10.0 mmol, 1.0 equiv), triethylamine (15.0 mmol, 1.5 equiv, 2.1 mL), and TMSOTf (11.0 mmol, 1.1 equiv, 2.0 mL). **1l** was obtained as a colorless liquid (87:13 *er*). Analytical data were in agreement with literature values[1]. ^{1}H NMR (400 MHz, $CDCl_3$) δ 6.90 (dd, J = 5.1, 1.2 Hz, 1H), 6.83–6.80 (m, 1H), 6.73–6.70 (m, 1H), 5.02 (t, J = 7.2 Hz, 1H), 1.97 (p, J = 7.5 Hz, 2H), 0.82 (t, J = 7.6 Hz, 3H), 0.00 (s, 9H).

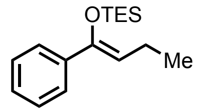

Triethyl((1-phenylbut-1-en-1-yl)oxy)silane (1n): The reaction was carried out following General Procedure B using 1-phenylbutan-1-one (10.0 mmol, 1.0 equiv), triethylamine (15.0 mmol, 1.5 equiv, 2.1 mL), and TESCl (11.0 mmol, 1.1 equiv). **1m** was obtained as a colorless liquid (94:6 *er*). Analytical data were in agreement with literature values[1]. ^{1}H NMR (400 MHz, $CDCl_3$) δ 7.48–7.42 (m, 2H), 7.33–7.21 (m, 3H), 5.12 (t, J = 7.1 Hz, 1H), 2.24 (p, J = 7.5 Hz, 2H), 1.04 (t, J = 7.5 Hz, 3H), 0.93 (t, J = 7.9 Hz, 9H), 0.61 (q, J = 8.1 Hz, 6H).

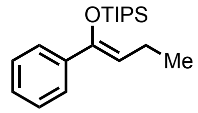

Triisopropyl((1-phenylbut-1-en-1-yl)oxy)silane (1o): The reaction was carried out following General Procedure B using 1-phenylbutan-1-one (10.0 mmol, 1.0 equiv), triethylamine (15.0 mmol, 1.5 equiv, 2.1 mL), and TIPSCl (11.0 mmol, 1.1 equiv). **1n** was obtained as a colorless liquid (86:14 *er*). Analytical data were in agreement with literature values[1]. ^{1}H NMR (400 MHz, $CDCl_3$) δ 7.49–7.40 (m, 2H), 7.31–7.25 (m, 3H), 4.95 (t, J = 7.0 Hz, 1H), 2.25 (p, J = 7.5 Hz, 2H), 1.08–0.97 (m, 24H).

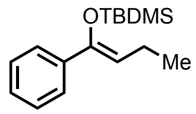

***tert*-Butyldimethyl((1-phenylbut-1-en-1-yl)oxy)silane (1p):** The reaction was carried out following General Procedure B using 1-phenylbutan-1-one (10.0 mmol, 1.0 equiv), triethylamine (15.0 mmol, 1.5 equiv, 2.1 mL), and TBDMSCl (11.0 mmol, 1.1 equiv). **1o** was obtained as a colorless liquid (95:5 *er*). Analytical data were in agreement with literature values[1]. ^{1}H NMR (400 MHz, $CDCl_3$) δ 7.49–7.42 (m, 2H), 7.32–7.23 (m, 3H), 5.09 (t, J = 7.1 Hz, 1H), 2.22 (p, J = 7.5 Hz, 2H), 1.03 (t, J = 7.3 Hz, 3H), 0.98 (s, 9H), 0.92 (s, 6H).

V. Preparation and Characterization of α-Amino Ketone Standard Products

General procedure C:

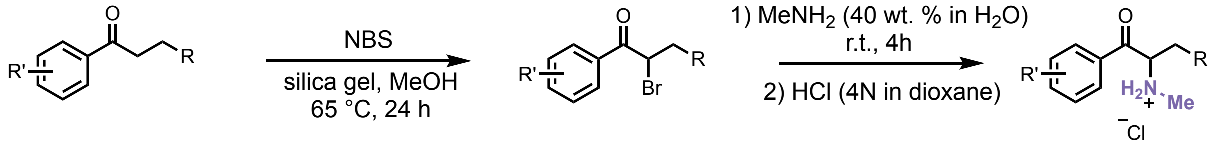

Bromination:

Corresponding ketone (10.0 mmol, 1.0 equiv.) and 28-200 mesh silica gel (1.0 equiv. to mass) were dissolved in methanol (50 mL). *N*-Bromosuccunimide was added to the reaction mixture in six batches and stirred for 24 h at 65 °C. The reaction mixture was filtered and concentrated in vacuum. The mixture was extracted with ethyl acetate (30 mL × 3). The organic layer was washed with saturated $NaHCO_3$ (40 mL × 2) and then washed with brine. Flash column chromatography (5% ethyl acetate in hexanes as eluent) was performed to afford pure product in 20% to 80% yield.

Amination:

To a 2-dram vial containing α-bromo ketones (0.5 mmol) was added methylamine (0.5 mL, 40% aqueous solution). The reaction mixture was stirred at room temperature for 4 h. After this time, the reaction was quenched with brine. The aqueous solution was extracted with ethyl acetate (2 mL ×3). The combined organic layers were dried over Na_2SO_4. The organic solvent was removed under reduced pressure, and the residue was subjected to thin-layer chromatography on preparative thin-layer chromatography (PTLC, 1000 micron) with DCM/MeOH (95:5) as the eluent to afford the α-methylamino ketones. Subsequently, the solution of α-methylamino ketones in diethyl ether was added HCl (2.0 equiv, 4N in dioxane) to afford the precipitate of α-methylamino ketone HCl salts as desired products. The supernatant was removed. The product was triturated in hexane and dried under vacuum.

Synthesis of (*S*)-3j:

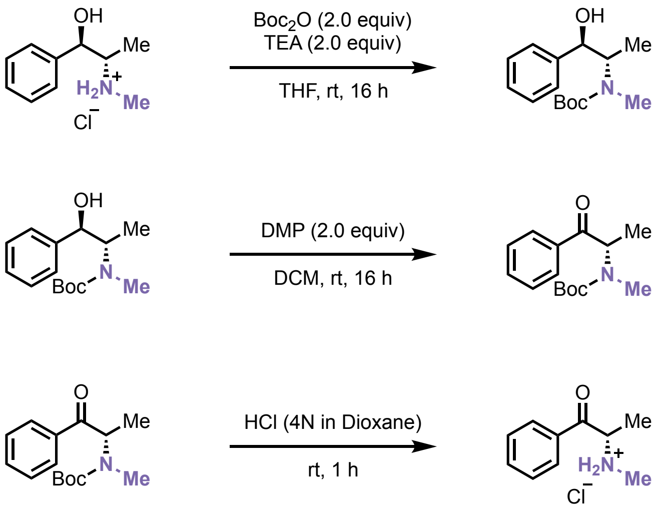

Protection:

To a 100-mL round-bottom-flask containing (1*R*,2*S*)-2-(methylamino)-1-phenylpropan-1-ol (10 mmol) and triethylamine (20 mmol) at 0 °C was added di-*tert*-butyl decarbonate (20 mmol) portion wise. The reaction mixture was gradually warmed to room temperature and stirred for 16 h. After this time, the reaction was quenched with brine. The aqueous solution was extracted with ethyl acetate (50 mL ×3). The combined organic layers were dried over Na_2SO_4. The organic solvent was removed under reduced pressure, and the residue was subjected to flash column chromatography to afford the Boc protected amino alcohol product.

Oxidation:

Subsequently, a solution of the Boc-protected amino alcohol in DCM (0.2 M) was added portion wise to Dess–Martin periodinane (20 mmol). The reaction mixture was stirred at room temperature for 16 h. After this time, the reaction was quenched with brine. The aqueous solution was extracted with DCM (50 mL ×3). The combined organic layers were dried over Na_2SO_4. The organic solvent was removed under reduced pressure, and the residue was subjected to flash column chromatography to afford the Boc protected amino ketone product.

Deprotection:

To a 20-mL scintillation vial containing the Boc protected amino ketone (0.2 g) was added 5 mL HCL solution (4N in Dioxane). The reaction was stirred at room temperature until the full consumption of starting material monitored by TLC. The organic solvent was removed under constant air flow to afford **(*S*)-3j** as a white solid.

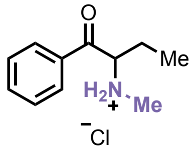

2-(Methylamino)-1-phenylbutan-1-one hydrochloride (3a): The reaction was carried out following General Procedure **C**, using **1a** (5 mM), and *N*-methyl hydroxylamine hydrochloride (20 mM). **3a** was obtained in 77% yield, 90% ee using **AKS-(S)-G3**, and 70% yield, –92% ee using **AKS-(R)-G3**. **^{1}H NMR** (400 MHz, D_2O) δ 7.95 (dd, *J* = 8.5, 1.3 Hz, 2H), 7.76–7.63 (m, 1H), 7.54 (dd, *J* = 8.3, 7.4 Hz, 2H), 5.10 (t, *J* = 5.1 Hz, 1H), 2.10–1.92 (m, 2H), 1.26 (t, *J* = 7.3 Hz, 3H). **^{13}C NMR** (101 MHz, D_2O) δ 197.16, 135.47, 133.02, 129.24, 128.81, 64.40, 31.56, 23.03, 7.27. **HRMS** (ESI-TOF) Calcd for $C_{11}H_{16}NO^+$ [M+H] 178.1226, found 178.1226.

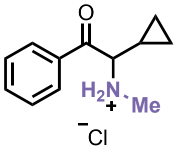

2-Cyclopropyl-2-(methylamino)-1-phenylethan-1-one hydrochloride (3b): The reaction was carried out following General Procedure **C**, using **1b** (5 mM), and *N*-methyl hydroxylamine hydrochloride (20 mM). **3b** was obtained in 24% yield, using **AKS-(S)-G3**. **^{1}H NMR** (400 MHz, D_2O) δ 8.00–7.94 (m, 2H), 7.74 (t, J = 7.4 Hz, 1H), 7.59 (t, J = 7.6 Hz, 2H), 4.57 (d, J = 9.7 Hz, 1H), 2.62 (d, J = 1.2 Hz, 3H), 0.99–0.88 (m, 1H), 0.78–0.68 (m, 2H), 0.66–0.56 (m, 1H), 0.56–0.42 (m, 1H), 0.42–0.27 (m, 1H). **^{13}C NMR** (101 MHz, D_2O) δ 196.76, 135.37, 134.10, 129.17, 129.05, 66.31, 31.27, 11.24, 5.28, 3.86. **HRMS** (ESI-TOF) Calcd for $C_{12}H_{16}NO^+$ [M+H] 190.1226, found 190.1226.

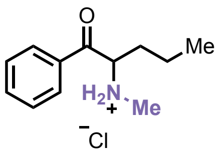

2-(Methylamino)-1-phenylpentan-1-one hydrochloride (3c): The reaction was carried out following General Procedure **C**, using **1c** (5 mM), and *N*-methyl hydroxylamine hydrochloride (20 mM). **3c** was obtained in 29% yield, 87% ee using **AKS-(S)-G3**, and 39% yield, –88% ee using **AKS-(R)-G3**. **^{1}H NMR** (400 MHz, D_2O) ^{1}H NMR (400 MHz, D_2O) δ 8.07–7.97 (m, 2H), 7.84–7.74 (m, 1H), 7.66–7.56 (m, 2H), 5.15 (t, *J* = 5.4 Hz, 1H), 2.78 (s, 3H), 2.15–1.88 (m, 2H), 1.42–1.14 (m, 2H), 0.83 (t, *J* = 7.3 Hz, 3H). **^{13}C NMR** (101 MHz, D_2O) δ 197.29, 135.46, 133.07, 129.25, 128.81, 63.69, 31.78, 31.69, 16.98, 12.83. **HRMS** (ESI-TOF) Calcd for $C_{12}H_{18}NO^+$ [M+H] 192.1383, found 192.1383.

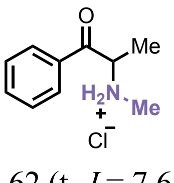

2-(Methylamino)-1-phenylpropan-1-one hydrochloride (3d): The reaction was carried out following General Procedure **C**, using **1d** (5 mM), and *N*-methyl hydroxylamine hydrochloride (20 mM). **3d** was obtained in 57% yield, 48% ee using **AKS-(S)-G3**, and 39% yield, –26% ee using **AKS-(R)-G3**. **^{1}H NMR** (400 MHz, D_2O) ^{1}H NMR (400 MHz, D_2O) δ 8.39–7.96 (m, 2H), 7.87–7.70 (m, 1H), 7.62 (t, *J* = 7.6 Hz, 2H), 5.11 (q, *J* = 7.3 Hz, 1H), 2.81 (s, 2H), 1.88–1.47 (m, 3H). **^{13}C NMR** (101 MHz, DMSO) δ 196.37, 134.73, 132.90, 129.21, 128.82, 58.24, 30.66, 26.88, 15.40. **HRMS** (ESI-TOF) Calcd for $C_{10}H_{14}NO^+$ [M+H] 164.1070, found 164.1070.

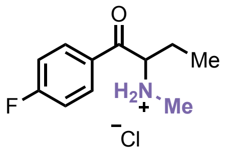

1-(4-Fluorophenyl)-2-(methylamino)butan-1-one hydrochloride (3e): The reaction was carried out following General Procedure **C**, using **1e** (5 mM), and *N*-methyl hydroxylamine hydrochloride (20 mM). **3e** was obtained in 47% yield, 89% ee using **AKS-(S)-G3**, and 44% yield, –88% ee using **AKS-(R)-G3**. **^{1}H NMR** (400 MHz, D_2O) δ 8.11 (dd, *J* = 9.0, 5.3 Hz, 1H), 7.35 (t, *J* = 8.8 Hz, 1H), 5.13 (t, *J* = 5.1 Hz, 1H), 2.78 (s, 3H), 2.12 (dpd, *J* = 23.2, 7.8, 5.2 Hz, 2H), 0.87 (t, *J* = 7.6 Hz, 3H). **^{13}C NMR** (101 MHz, D_2O) δ 195.50, 166.79 (d, $J_{C–F}$ = 256.0 Hz), 131.95 (d, $J_{C–F}$ = 10.2 Hz), 129.57 (d, $J_{C–F}$ = 2.9 Hz), 116.41 (d, $J_{C–F}$ = 22.5 Hz), 64.29, 31.57, 23.06, 7.27. **^{19}F NMR** (376 MHz, D_2O) δ –102.06. **HRMS** (ESI-TOF) Calcd for $C_{11}H_{15}FNO^+$ [M+H] 196.1132, found 196.1132.

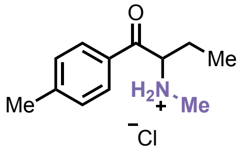

2-(Methylamino)-1-(*p*-tolyl)butan-1-one hydrochloride (3f): The reaction was carried out following General Procedure **C**, using **1f** (5 mM), and *N*-methyl hydroxylamine hydrochloride (20 mM). **3f** was obtained in 35% yield, 95% ee using **AKS-(S)-G3**, and 30% yield, –86% ee using **AKS-(R)-G3**. **^{1}H NMR** (400 MHz, D_2O) δ 7.94 (d, *J* = 8.3 Hz, 2H), 7.46 (d, *J* = 8.4 Hz, 2H), 5.09 (t, *J* = 5.2 Hz, 1H), 2.76 (s, 3H), 2.21–2.01 (m, 2H), 0.86 (t, *J* = 7.6 Hz, 3H). **^{13}C NMR** (101 MHz, D_2O) δ 196.62, 147.51, 130.45, 129.86, 128.98, 64.23, 31.56, 23.22, 20.93, 7.31. **HRMS** (ESI-TOF) Calcd for $C_{12}H_{18}NO^+$ [M+H] 192.1383, found 192.1383.

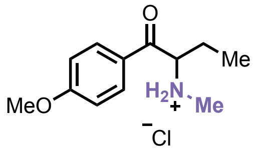

1-(4-Methoxyphenyl)-2-(methylamino)butan-1-one hydrochloride (3g): The reaction was carried out following General Procedure **1D**, using **1g** (5 mM), and *N*-methyl hydroxylamine hydrochloride (20 mM). **3g** was obtained in 79% yield, 79% ee using **AKS-(S)-G3**, and 69% yield, –92% ee using **AKS-(R)-G3**. **^{1}H NMR** (400 MHz, D_2O) δ 8.04 (d, *J* = 9.0 Hz, 2H), 7.15 (d, *J* = 9.0 Hz, 2H), 5.08 (t, *J* = 5.2 Hz, 1H), 3.94 (s, 3H), 2.75 (s, 3H), 2.19–1.98 (m, 2H), 0.86 (t, *J* = 7.6 Hz, 3H). **^{13}C NMR** (101 MHz, D_2O) δ 195.22, 164.98, 131.59, 126.07, 114.57,

63.97, 55.72, 31.57, 23.43, 7.35. **HRMS** (ESI-TOF) Calcd for $C_{12}H_{18}NO_2^+$ [M+H] 208.1332, found 208.1319.

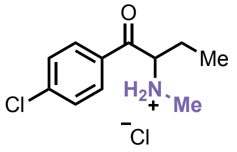

1-(4-Chlorophenyl)-2-(methylamino)butan-1-one hydrochloride (3h): The reaction was carried out following General Procedure **C**, using **1h** (5 mM), and *N*-methyl hydroxylamine hydrochloride (20 mM). **3h** was obtained in 24% yield, 83% ee using **AKS-(S)-G3**, and 25% yield, –87% ee using **AKS-(R)-G3**. ^{1}**H NMR** (400 MHz, D_2O) δ 7.59 (d, *J* = 8.5 Hz, 2H), 7.40 (d, *J* = 8.5 Hz, 2H), 5.12 (t, *J* = 5.2 Hz, 1H), 2.60 (s, 3H), 2.12 (dqd, *J* = 22.9, 7.8, 3.8 Hz, 2H), 0.86 (t, *J* = 7.6 Hz, 3H). ^{1}**H NMR** (400 MHz, D_2O) δ 8.00 (d, *J* = 8.7 Hz, 2H), 7.63 (d, *J* = 8.7 Hz, 2H), 5.12 (t, *J* = 5.1 Hz, 1H), 2.77 (s, 3H), 2.26–1.97 (m, 2H), 0.85 (t, *J* = 7.6 Hz, 3H). ^{13}**C NMR** (101 MHz, D_2O) δ 195.95, 141.34, 131.48, 130.35, 129.44, 64.35, 31.57, 22.99, 7.28. **HRMS** (ESI-TOF) Calcd for $C_{11}H_{15}ClNO^+$ [M+H] 212.0837, found 212.0837.

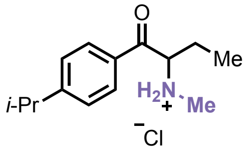

1-(4-Isopropylphenyl)-2-(methylamino)butan-1-one hydrochloride (3i): The reaction was carried out following General Procedure **C**, using **1i** (5 mM), and *N*-methyl hydroxylamine hydrochloride (20 mM). **3i** was obtained in 12% yield, 95% ee using **AKS-(S)-G3**, and 4.1% yield, –87% ee using **AKS-(R)-G3**. ^{1}**H NMR** (400 MHz, D_2O) δ 7.90 (d, *J* = 8.2 Hz, 2H), 7.46 (d, *J* = 8.2 Hz, 2H), 5.04 (t, *J* = 5.2 Hz, 1H), 2.96 (p, *J* = 7.0 Hz, 1H), 2.70 (s, 3H), 2.13–1.93 (m, 2H), 1.19 (d, *J* = 6.9 Hz, 6H), 0.78 (t, *J* = 7.6 Hz, 3H). ^{13}**C NMR** (101 MHz, D_2O) δ 196.63, 158.05, 130.83, 129.19, 127.44, 64.26, 33.92, 31.56, 23.21, 22.73, 22.68, 7.31. **HRMS** (ESI-TOF) Calcd for $C_{14}H_{22}NO^+$ [M+H] 220.1696, found 220.1696.

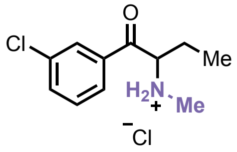

1-(3-Chlorophenyl)-2-(methylamino)butan-1-one hydrochloride (3j): The reaction was carried out following General Procedure **C**, using **1j** (5 mM), and *N*-methyl hydroxylamine hydrochloride (20 mM). **3j** was obtained in 54% yield, 74% ee using **AKS-(S)-G3**, and 48% yield, –70% ee using **AKS-(R)-G3**. ^{1}**H NMR** (400 MHz, D_2O) δ 8.04 (t, *J* = 1.9 Hz, 1H), 7.93 (ddd, *J* = 7.9, 1.8, 1.0 Hz, 1H), 7.77 (ddd, *J* = 8.1, 2.2, 1.0 Hz, 1H), 7.58 (t, *J* = 8.0 Hz, 1H), 5.11 (t, *J* = 5.1 Hz, 1H), 2.77 (s, 3H), 2.21–1.96 (m, 2H), 0.85 (t, *J* = 7.6 Hz, 3H). ^{13}**C NMR** (101 MHz, D_2O) δ 195.96, 135.04, 134.84, 134.60, 130.72, 128.51, 127.16, 66.53, 31.57, 22.84, 7.22. **HRMS** (ESI-TOF) Calcd for $C_{11}H_{15}ClNO^+$ [M+H] 212.0837, found 212.0837.

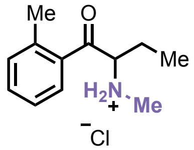

2-(Methylamino)-1-(*o*-tolyl)butan-1-one hydrochloride (3k): The reaction was carried out following General Procedure **C**, using **1k** (5 mM), and *N*-methyl hydroxylamine hydrochloride (20 mM). **3k** was obtained in 1.3% yield, 36% ee using **AKS-(S)-G3**, and 0.9% yield, –46% ee using **AKS-(R)-G3**. ^{1}**H NMR** (400 MHz, D_2O) δ 7.79 (d, *J* = 7.8 Hz, 1H), 7.64–7.55 (m, 1H), 7.48–7.38 (m, 2H), 5.04 (t, *J* = 5.3 Hz, 1H), 2.81 (s, 3H), 2.49 (s, 3H), 2.12–1.88 (m, 2H), 0.83 (t, *J* = 7.6 Hz, 3H). ^{13}**C NMR** (101 MHz, D_2O) δ 199.99, 139.69, 133.65, 133.12, 132.46, 129.38, 126.20, 65.77, 31.46, 22.41, 20.19, 7.49. **HRMS** (ESI-TOF) Calcd for $C_{12}H_{18}NO^+$ [M+H] 192.1383, found 192.1383.

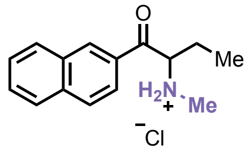

2-(Methylamino)-1-(naphthalen-2-yl)butan-1-one hydrochloride (3l): The reaction was carried out following General Procedure **C**, using **1l** (5 mM), and *N*-methyl hydroxylamine hydrochloride (20 mM). **3l** was obtained in 13% yield, 90% ee using **AKS-(S)-G3**, and 13% yield, –84% ee using **AKS-(R)-G3**. **^{1}H NMR** (400 MHz, D_2O) δ 8.67 (s, 1H), 8.15–7.99 (m, 4H), 7.80–7.64 (m, 2H), 5.30 (t, *J* = 5.2 Hz, 1H), 2.82 (s, 3H), 2.31–2.11 (m, 2H), 0.89 (t, *J* = 7.6 Hz, 3H). **^{13}C NMR** (101 MHz, D_2O) δ 196.93, 136.15, 132.01, 131.77, 130.50, 129.97, 129.84, 129.20, 127.85, 127.57, 123.24, 64.41, 31.63, 23.33, 7.37. **HRMS** (ESI-TOF) Calcd for $C_{15}H_{18}NO^+$ [M+H] 228.1383, found 228.1383.

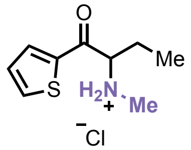

2-(Methylamino)-1-(thiophen-2-yl)butan-1-one hydrochloride (3m): The reaction was carried out following General Procedure **C**, using **1m** (5 mM), and *N*-methyl hydroxylamine hydrochloride (20 mM). **3m** was obtained in 60% yield, 94% ee using **AKS-(S)-G3**, and 56% yield, –90% ee using **AKS-(R)-G3**. **^{1}H NMR** (400 MHz, D_2O) δ 8.05–7.97 (m, 2H), 7.25 (dd, *J* = 4.9, 4.0 Hz, 1H), 4.87 (t, *J* = 5.5 Hz, 1H), 2.67 (s, 3H), 2.21–2.00 (m, 2H), 0.84 (t, *J* = 7.6 Hz, 3H). **^{13}C NMR** (101 MHz, D_2O) δ 189.16, 139.44, 138.49, 136.25, 129.44, 64.62, 31.52, 24.09, 7.63. **HRMS** (ESI-TOF) Calcd for $C_9H_{14}NOS^+$ [M+H] 184.0791, found 184.0791.

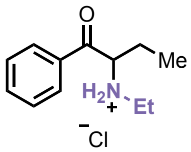

2-(Ethylamino)-1-phenylbutan-1-one hydrochloride (3q): The reaction was carried out following General Procedure **1D**, using **1a** (5 mM), and *N*-ethyl hydroxylamine hydrochloride (20 mM). **3q** was obtained in 32% yield, 92% ee using **AKS-(S)-G3**, and 6% yield, –59% ee using **AKS-(R)-G3**. **^{1}H NMR** (400 MHz, D_2O) δ 7.96 (d, *J* = 7.2 Hz, 1H), 7.71 (ddt, *J* = 7.9, 7.1, 1.3 Hz, 1H), 7.59–7.52 (m, 2H), 5.10 (t, *J* = 5.2 Hz, 1H), 3.16–2.99 (m, 2H), 2.10–1.96 (m, 2H), 1.26 (t, *J* = 7.3 Hz, 3H), 0.78 (t, *J* = 7.6 Hz, 3H). **^{13}C NMR** (101 MHz, D_2O) δ 197.22, 135.48, 133.06, 129.25, 128.81, 62.78, 42.14, 23.31, 10.62, 7.42. **HRMS** (ESI-TOF) Calcd for $C_{12}H_{18}NO^+$ [M+H] 192.1383, found 192.1383.

VI. Analytic-Scale Enzymatic Reactions and Calibration Curves for Products

All enzymatic reactions for stereoconvergent, intermolecular amination reactions on analytical scale were conducted following the general procedure described in **Section I, General Methods D**. Reactions for every substrate were set up in triplicate. Product formation was quantified by LC-MS based on the calibration curve of the corresponding racemic standard compound (**Section V**). All TTNs for different products were calculated as the concentration of products divided by the concentration of hemoproteins measured by the hemochrome assay (**Section I, General Methods C**).

Calibration curves of synthesized reference compounds were conducted for the determination of yield and TTN. For each substrate, at least five different concentrations of product (0.125, 0.25, 0.5, 1, 1.5, 2, 2.5, 3, 4, and 5 mM) were prepared in 400 µL M9-N (pH = 8.0) buffer. Internal standard solution (5 mM of 1,2,3-trimethylbenzene) was prepared in acetonitrile. The mixtures of standard product and 800-µL internal standard acetonitrile solution were vortexed and centrifuged to pellet down all the debris. The supernatant was analyzed by HPLC based on UV absorbance at 254 or 210 nm. The calibration curves depict the ratio of concentration in mM (x-axis) against product area to internal standard area (y-axis). Notes: Pdt = product area, IS = internal standard area, [Pdt] = product concentration in reaction, [PC] = protein concentration in reaction, Avg. TTN = average total turnover number, SD TTN = standard deviation of TTN, Avg. Yield = average yield, SD Yield = standard deviation of yield.

2-(Methylamino)-1-phenylbutan-1-one (3a)

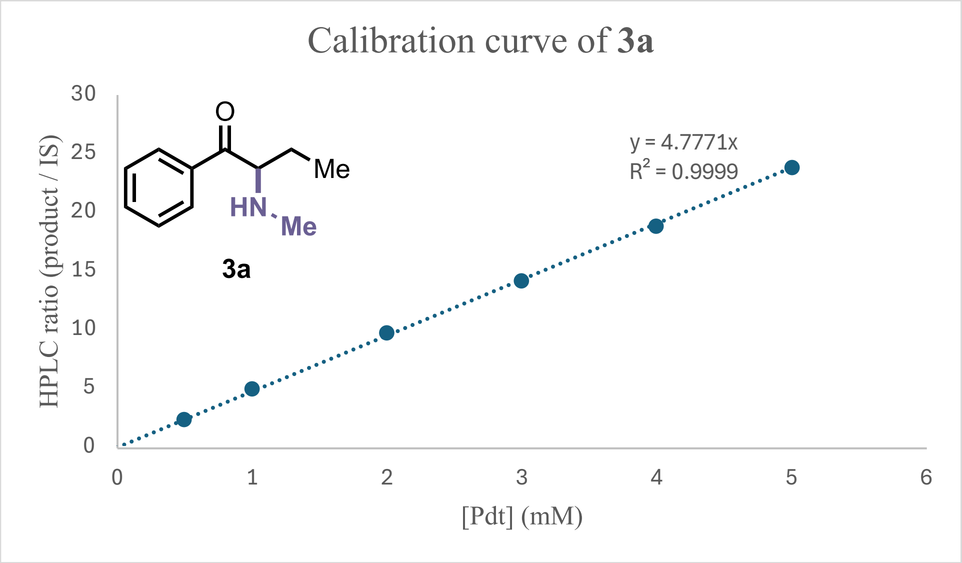

Data Analysis for Evolution Lineage:

Data analysis for ***pa*Pgb-AKS-G0-5329** catalyzed stereoconvergent amination of silyl enol ether **1a** (5 mM, OD_{600} = 30):

Entry	Pdt	IS	Pdt/IS	[Pdt]/mM	Yield [%]	Avg. Yield [%]	SD Yield [%]
1	50.6	967.3	0.665	0.139	2.8	3	0.02
2	40.9	945.9	0.672	0.141	2.8		

Data analysis for ***pa*****Pgb-AKS-(*****S*****)-G1-5330** catalyzed stereoconvergent amination of silyl enol ether **1a** (5 mM, $OD_{600} = 30$):

Entry	Pdt	IS	Pdt/IS	[Pdt]/mM	Yield [%]	Avg. Yield [%]	SD Yield [%]
1	141.9	104.5	1.36	0.284	5.7	6	0.01
2	144.9	106.4	1.36	0.285	5.7		

Data analysis for ***pa*****Pgb-AKS-(*****S*****)-G2-5331** catalyzed stereoconvergent amination of silyl enol ether **1a** (5 mM, $OD_{600} = 30$):

Entry	Pdt	IS	Pdt/IS	[Pdt]/mM	Yield [%]	Avg. Yield [%]	SD Yield [%]
1	333.6	105.1	3.17	0.664	13.3	13	0.8
2	307.4	105.3	2.92	0.611	12.2		

Data analysis for ***pa*****Pgb-AKS-(*****S*****)-G3-5332** catalyzed stereoconvergent amination of silyl enol ether **1a** (5 mM, $OD_{600} = 30$):

Entry	Pdt	IS	Pdt/IS	[Pdt]/mM	Yield [%]	Avg. Yield [%]	SD Yield [%]
1	503.4	104.9	4.80	1.01	20.1	22	3.2
2	624.2	105.9	5.89	1.23	24.7		

Data analysis for ***pa*****Pgb-AKS-(*****S*****)-G3-5332** **lysate** catalyzed stereoconvergent amination of silyl enol ether **1a** with 2.0 equiv. of *N*-methyl hydroxylamine **2d** (5 mM, $OD_{600} = 30$):

Entry	Pdt	IS	Pdt/IS	[Pdt]/mM	Yield [%]	Avg. Yield [%]	SD Yield [%]
1	1520.8	105.8	14.4	3.01	60.2	59	1.8
2	1467.7	106.6	13.8	2.88	57.6		

Data analysis for ***pa*****Pgb-AKS-(*****R*****)-G1-5333** catalyzed stereoconvergent amination of silyl enol ether **1a** (5 mM, $OD_{600} = 30$):

Entry	Pdt	IS	Pdt/IS	[Pdt]/mM	Yield [%]	Avg. Yield [%]	SD Yield [%]
1	169.6	102.7	1.65	0.346	6.9	8	1.1
2	211.5	104.8	2.02	0.422	8.4		

Data analysis for ***pa*Pgb-AKS-(*R*)-G2-5334** catalyzed stereoconvergent amination of silyl enol ether **1a** (5 mM, $OD_{600} = 30$):

Entry	Pdt	IS	Pdt/IS	[Pdt]/mM	Yield [%]	Avg. Yield [%]	SD Yield [%]
1	238.0	104.3	2.28	0.478	9.6	10	0.2
2	246.0	105.4	2.33	0.489	9.8		

Data analysis for ***pa*Pgb-AKS-(*R*)-G3-5335** catalyzed stereoconvergent amination of silyl enol ether **1a** (5 mM, $OD_{600} = 30$):

Entry	Pdt	IS	Pdt/IS	[Pdt]/mM	Yield [%]	Avg. Yield [%]	SD Yield [%]
1	593.2	105.4	5.63	1.18	23.6	25	2.3
2	677.9	105.8	6.41	1.34	26.8		

Data analysis for ***pa*Pgb-AKS-(*R*)-G3-5335** **lysate** catalyzed stereoconvergent amination of silyl enol ether **1a** with 2.0 equiv. of *N*-methyl hydroxylamine **2d** (5 mM, $OD_{600} = 30$):

Entry	Pdt	IS	Pdt/IS	[Pdt]/mM	Yield [%]	Avg. Yield [%]	SD Yield [%]
1	1085.8	107.2	10.1	2.12	42.4	42	0.3
2	1083.8	108.0	10.0	2.10	42.0		

Data Analysis for Substrate 1a, 1n, 1o, and 1p with standard conditions:

Data analysis for ***pa*Pgb-AKS-(*S*)-G3-5332** **lysate** catalyzed stereoconvergent amination of silyl enol ether **1a** with 2.0+2.0 equiv. of *N*-methyl hydroxylamine **2d** (5 mM, $OD_{600} = 30$):

Entry	Pdt	IS	Pdt/IS	[Pdt]/mM	Yield [%]	Avg. Yield [%]	SD Yield [%]
1	2044.1	116.0	17.6	3.69	73.8	77	3.2
2	2141.2	112.2	19.1	3.99	79.9		
3	2130.8	114.1	18.7	3.91	78.2		

Data analysis for ***pa*Pgb-AKS-(*S*)-G3-5332** lysate catalyzed stereoconvergent amination of silyl enol ether **1n** with 2.0+2.0 equiv. of *N*-methyl hydroxylamine **2d** (5 mM, OD_{600} = 30):

Entry	Pdt	IS	Pdt/IS	[Pdt]/mM	Yield [%]	Avg. Yield [%]	SD Yield [%]
1	263.7	106.2	2.48	0.520	10.4	10	0.06
2	265.6	106.3	2.50	0.523	10.5		
3	260.1	105.4	2.47	0.517	10.3		

Data analysis for ***pa*Pgb-AKS-(*S*)-G3-5332** lysate catalyzed stereoconvergent amination of silyl enol ether **1o** with 2.0+2.0 equiv. of *N*-methyl hydroxylamine **2d** (5 mM, OD_{600} = 30):

Entry	Pdt	IS	Pdt/IS	[Pdt]/mM	Yield [%]	Avg. Yield [%]	SD Yield [%]
1	12.2	105	0.116	0.0243	0.49	0.5	0.05
2	12.2	106.9	0.114	0.0239	0.48		
3	10.1	107	0.094	0.0198	0.40		

Data analysis for ***pa*Pgb-AKS-(*S*)-G3-5332** lysate catalyzed stereoconvergent amination of silyl enol ether **1p** with 2.0+2.0 equiv. of *N*-methyl hydroxylamine **2d** (5 mM, OD_{600} = 30):

Entry	Pdt	IS	Pdt/IS	[Pdt]/mM	Yield [%]	Avg. Yield [%]	SD Yield [%]
1	243.7	105.3	2.31	0.484	9.7	10	0.2
2	243.8	105.6	2.31	0.483	9.7		
3	237.4	105.7	2.25	0.470	9.4		

Data analysis for ***pa*Pgb-AKS-(*R*)-G3-5335** lysate catalyzed stereoconvergent amination of silyl enol ether **1a** with 2.0+2.0 equiv. of *N*-methyl hydroxylamine **2d** (5 mM, OD_{600} = 30):

Entry	Pdt	IS	Pdt/IS	[Pdt]/mM	Yield [%]	Avg. Yield [%]	SD Yield [%]
1	1874.7	112.6	16.6	3.49	69.7	70	0.3
2	1894.3	113.4	16.7	3.50	69.9		
3	1888.3	112.4	16.8	3.52	70.3		

Data analysis for ***pa*Pgb-AKS-(*R*)-G3-5335** lysate catalyzed stereoconvergent amination of silyl enol ether **1n** with 2.0+2.0 equiv. of *N*-methyl hydroxylamine **2d** (5 mM, OD_{600} = 30):

Entry	Pdt	IS	Pdt/IS	[Pdt]/mM	Yield [%]	Avg. Yield [%]	SD Yield [%]
1	258.3	106.5	2.43	0.508	10.2	10	0.4
2	244.5	106.2	2.30	0.482	9.6		
3	235.3	106.2	2.22	0.464	9.3		

Data analysis for ***pa*Pgb-AKS-(*R*)-G3-5335** lysate catalyzed stereoconvergent amination of silyl enol ether **1o** with 2.0+2.0 equiv. of *N*-methyl hydroxylamine **2d** (5 mM, $OD_{600} = 30$):

Entry	Pdt	IS	Pdt/IS	[Pdt]/mM	Yield [%]	Avg. Yield [%]	SD Yield [%]
1	13.9	106.0	0.131	0.0275	0.55	0.6	0.3
2	14.4	106.3	0.135	0.0284	0.57		
3	14.5	106.2	0.137	0.0286	0.57		

Data analysis for ***pa*Pgb-AKS-(*R*)-G3-5335** lysate catalyzed stereoconvergent amination of silyl enol ether **1p** with 2.0+2.0 equiv. of *N*-methyl hydroxylamine **2d** (5 mM, $OD_{600} = 30$):

Entry	Pdt	IS	Pdt/IS	[Pdt]/mM	Yield [%]	Avg. Yield [%]	SD Yield [%]
1	174.3	106.5	1.64	0.343	6.9	7	0.3
2	183.1	105.4	1.74	0.364	7.3		
3	182.6	106.0	1.72	0.361	7.2		

2-Cyclopropyl-2-(methylamino)-1-phenylethan-1-one (3b)

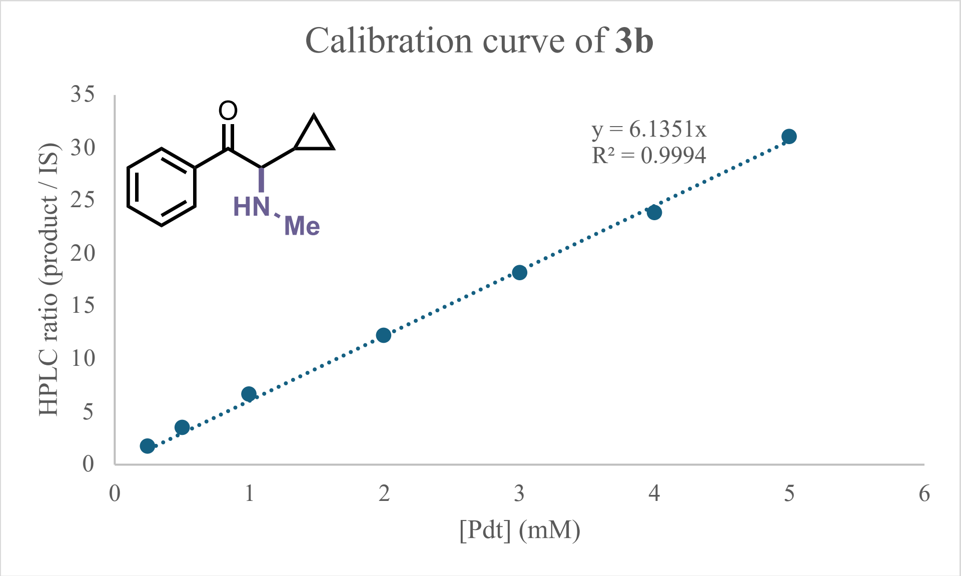

Data analysis for ***pa*Pgb-AKS-(*S*)-G3-5332** (M9-N, pH = **8.0**) catalyzed stereoconvergent amination reaction with **1b** (5.0 mM, OD_{600} = 30) under standard conditions:

Entry	Pdt	IS	Pdt/IS	[Pdt]/mM	Yield [%]	Avg. Yield [%]	SD Yield [%]
1	919.8	113.5	8.10	1.32	26.4	24	2.3
2	772.0	114.0	6.77	1.10	22.1		
3	872.3	125.9	6.93	1.13	22.6		

2-(Methylamino)-1-phenylpentan-1-one (3c)

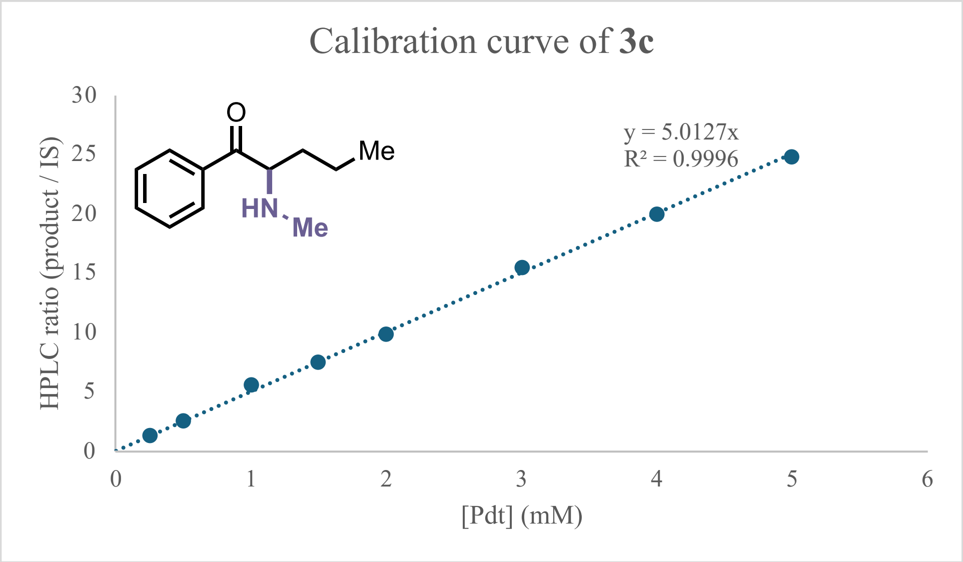

Data analysis for ***pa*****Pgb-AKS-(*S*)-G3-5332** (M9-N, pH = **8.0**) catalyzed stereoconvergent amination reaction with **1c** (5.0 mM, OD_{600} = 30) under standard conditions:

Entry	Pdt	IS	Pdt/IS	[Pdt]/mM	Yield [%]	Avg. Yield [%]	SD Yield [%]
1	1300.9	113.8	11.4	2.28	45.6	46	0.7
2	1284.7	109.2	11.8	2.35	46.9		
3	1266.2	109.0	11.6	2.32	46.3		

Data analysis for ***pa*****Pgb-AKS-(*R*)-G3-5335** (M9-N, pH = **8.0**) catalyzed stereoconvergent amination reaction with **1c** (5.0 mM, OD_{600} = 30) under standard conditions:

Entry	Pdt	IS	Pdt/IS	[Pdt]/mM	Yield [%]	Avg. Yield [%]	SD Yield [%]
1	910.8	109.9	8.29	1.65	33.1	33	0.8
2	901.2	108.6	8.30	1.66	33.1		
3	910.5	114.5	7.95	1.59	31.7		

2-(Methylamino)-1-phenylpropan-1-one (3d)

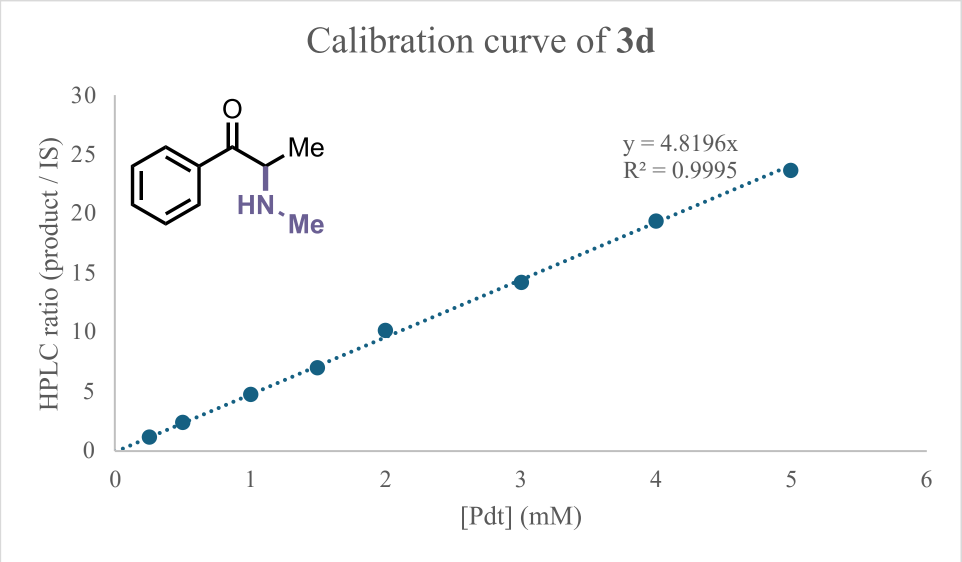

Data analysis for ***pa*****Pgb-AKS-(*****S*****)-G3-5332** (M9-N, pH = **8.0**) catalyzed stereoconvergent amination reaction with **1d** (5.0 mM, OD_{600} = 30) under standard conditions:

Entry	Pdt	IS	Pdt/IS	[Pdt]/mM	Yield [%]	Avg. Yield [%]	SD Yield [%]
1	1501.0	110.6	13.6	2.82	56.3	57	2.2
2	1495.4	111.5	13.4	2.78	55.7		
3	1568.3	108.9	14.4	2.99	59.8		

Data analysis for ***pa*****Pgb-AKS-(*****R*****)-G3-5335** (M9-N, pH = **8.0**) catalyzed stereoconvergent amination reaction with **1d** (5.0 mM, OD_{600} = 30) under standard conditions:

Entry	Pdt	IS	Pdt/IS	[Pdt]/mM	Yield [%]	Avg. Yield [%]	SD Yield [%]
1	1002.2	108.3	9.25	1.92	38.4	39	0.7
2	1022.4	106.5	9.60	1.99	39.8		
3	1028.6	108.1	9.52	1.97	39.5		

1-(4-Fluorophenyl)-2-(methylamino)butan-1-one (3e)

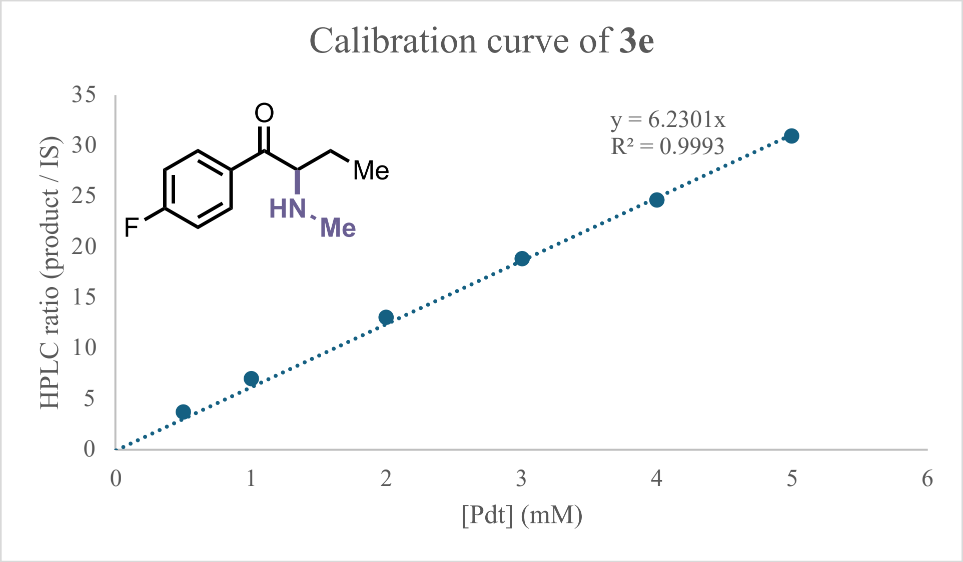

Data analysis for ***pa*Pgb-AKS-(*S*)-G3-5332** (M9-N, pH = **8.0**) catalyzed stereoconvergent amination reaction with **1e** (5.0 mM, OD_{600} = 30) under standard conditions:

Entry	Pdt	IS	Pdt/IS	[Pdt]/mM	Yield [%]	Avg. Yield [%]	SD Yield [%]
1	1525.1	105.5	14.5	2.32	46.4	47	1.1
2	1538.6	105.3	14.6	2.35	46.9		
3	1584.5	105.0	15.1	2.42	48.4		

Data analysis for ***pa*Pgb-AKS-(*R*)-G3-5335** (M9-N, pH = **8.0**) catalyzed stereoconvergent amination reaction with **1e** (5.0 mM, OD_{600} = 30) under standard conditions:

Entry	Pdt	IS	Pdt/IS	[Pdt]/mM	Yield [%]	Avg. Yield [%]	SD Yield [%]
1	1496.6	106.2	14.1	2.26	45.2	44	0.8
2	1474.8	107.2	13.8	2.21	44.2		
3	1462.0	107.2	13.6	2.19	43.8		

2-(Methylamino)-1-(*p*-tolyl)butan-1-one (3f)

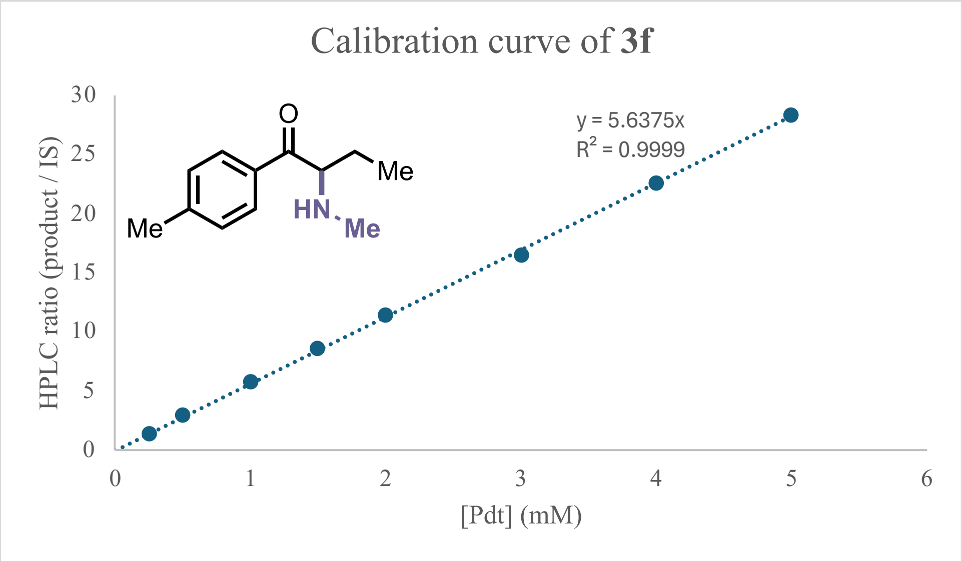

Data analysis for ***pa*Pgb-AKS-(*S*)-G3-5332** (M9-N, pH = **8.0**) catalyzed stereoconvergent amination reaction with **1f** (5.0 mM, OD_{600} = 30) under standard conditions:

Entry	Pdt	IS	Pdt/IS	[Pdt]/mM	Yield [%]	Avg. Yield [%]	SD Yield [%]
1	1244.1	121.0	10.3	1.82	36.5	36	0.8
2	1249.6	120.8	10.3	1.83	36.7		
3	1246.7	125.9	9.9	1.76	35.1		

Data analysis for ***pa*Pgb-AKS-(*R*)-G3-5335** (M9-N, pH = **8.0**) catalyzed stereoconvergent amination reaction with **1f** (5.0 mM, OD_{600} = 30) under standard conditions:

Entry	Pdt	IS	Pdt/IS	[Pdt]/mM	Yield [%]	Avg. Yield [%]	SD Yield [%]
1	994.4	113.3	8.78	1.56	31.1	31	0.3
2	1000.2	115.7	8.64	1.53	30.7		
3	992.6	112.8	8.80	1.56	31.2		

1-(4-Methoxyphenyl)-2-(methylamino)butan-1-one (3g)

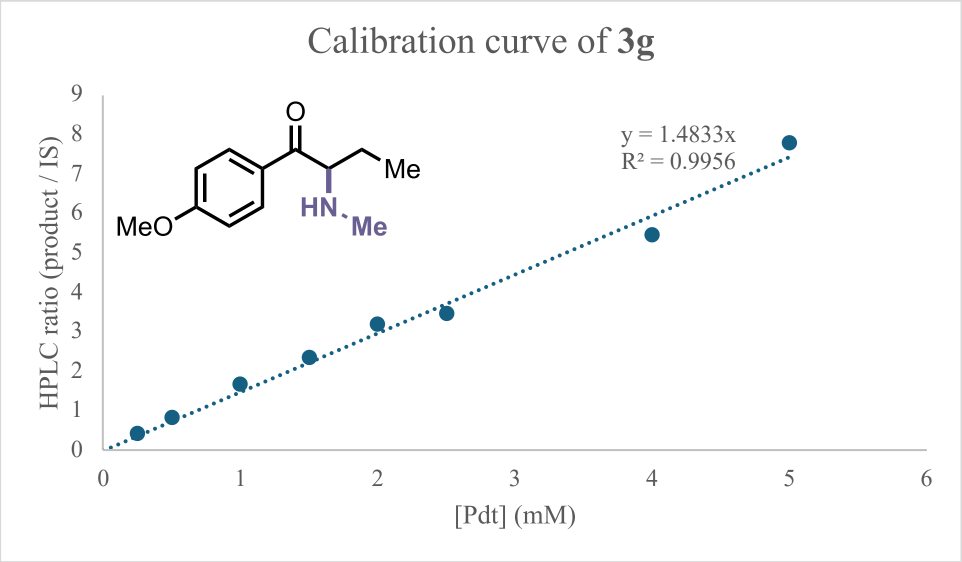

Data analysis for ***pa*Pgb-AKS-(*S*)-G3-5332** (M9-N, pH = **8.0**) catalyzed stereoconvergent amination reaction with **1g** (5.0 mM, OD_{600} = 30) under standard conditions:

Entry	Pdt	IS	Pdt/IS	[Pdt]/mM	Yield [%]	Avg. Yield [%]	SD Yield [%]
1	681.7	116.8	5.84	3.93	78.7	79	1.6
2	680.9	114.0	5.97	4.03	80.5		
3	668.7	116.7	5.73	3.86	77.3		

Data analysis for ***pa*Pgb-AKS-(*R*)-G3-5335** (M9-N, pH = **8.0**) catalyzed stereoconvergent amination reaction with **1g** (5.0 mM, OD_{600} = 30) under standard conditions:

Entry	Pdt	IS	Pdt/IS	[Pdt]/mM	Yield [%]	Avg. Yield [%]	SD Yield [%]
1	535.3	112.8	4.75	3.20	64.0	65	1.0
2	548.9	112.3	4.89	3.30	65.9		
3	549.8	114.6	4.80	3.23	64.7		

1-(4-Chlorophenyl)-2-(methylamino)butan-1-one (3h)

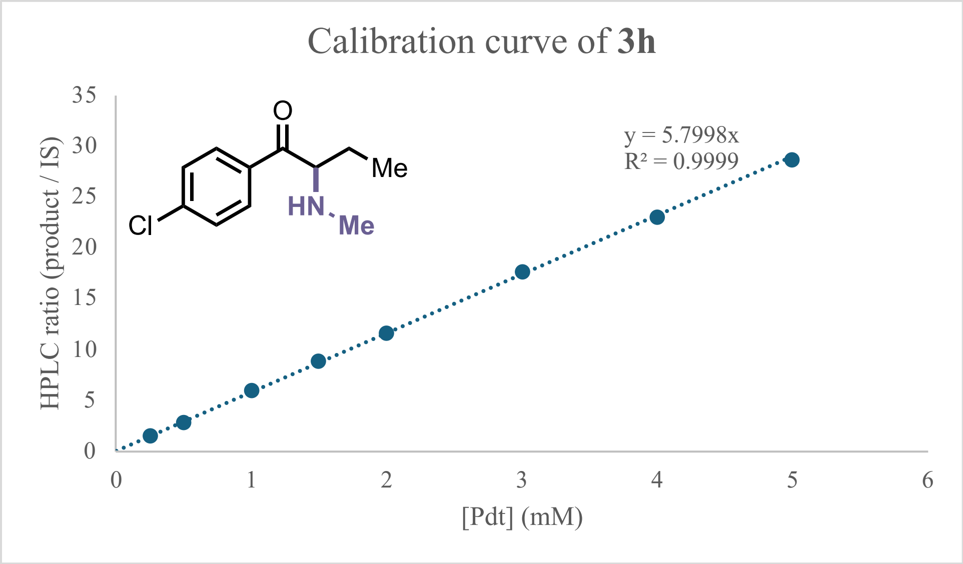

Data analysis for ***pa*Pgb-AKS-(*S*)-G3-5332** (M9-N, pH = **8.0**) catalyzed stereoconvergent amination reaction with **1h** (5.0 mM, OD_{600} = 30) under standard conditions:

Entry	Pdt	IS	Pdt/IS	[Pdt]/mM	Yield [%]	Avg. Yield [%]	SD Yield [%]
1	863.4	123.8	6.97	1.20	24.0	25	0.4
2	863.8	119.8	7.21	1.24	24.9		
3	860.6	120.7	7.13	1.23	24.6		

Data analysis for ***pa*Pgb-AKS-(*R*)-G3-5335** (M9-N, pH = **8.0**) catalyzed stereoconvergent amination reaction with **1h** (5.0 mM, OD_{600} = 30) under standard conditions:

Entry	Pdt	IS	Pdt/IS	[Pdt]/mM	Yield [%]	Avg. Yield [%]	SD Yield [%]
1	843.2	115.0	7.33	1.26	25.3	25	0.2
2	868.8	118.4	7.34	1.27	25.3		
3	862.3	118.9	7.25	1.25	25.0		

1-(4-Isopropylphenyl)-2-(methylamino)butan-1-one (3i)

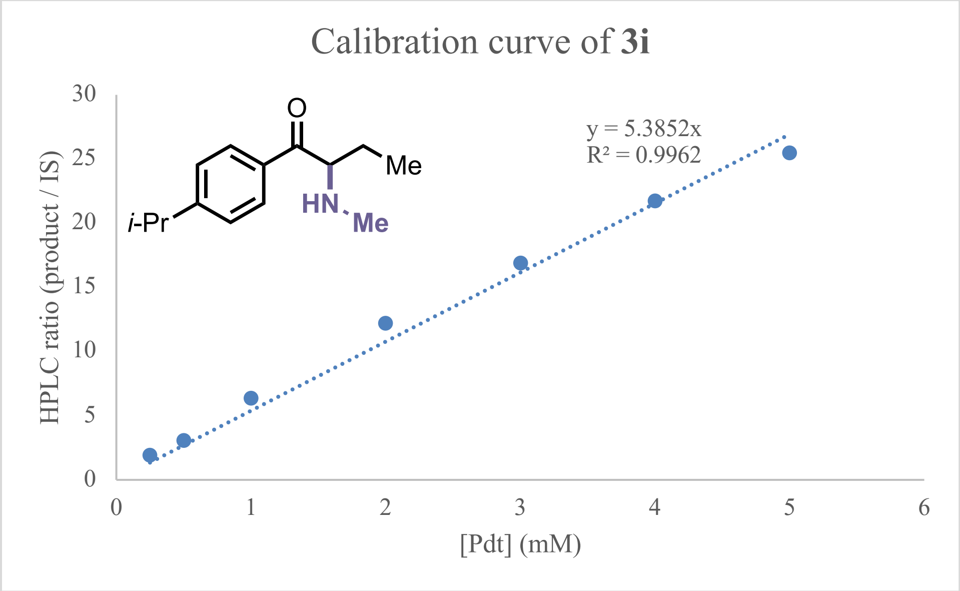

Data analysis for ***pa*****Pgb-AKS-(*****S*****)-G3-5332** (M9-N, pH = **8.0**) catalyzed stereoconvergent amination reaction with **1i** (5.0 mM, OD_{600} = 30) under standard conditions:

Entry	Pdt	IS	Pdt/IS	[Pdt]/mM	Yield [%]	Avg. Yield [%]	SD Yield [%]
1	85.2	93.7	0.909	0.169	3.38	4	0.1
2	92.9	95.3	0.975	0.181	3.62		
3	87.4	93.8	0.932	0.173	3.46		

Data analysis for ***pa*****Pgb-AKS-(*****R*****)-G3-5335** (M9-N, pH = **8.0**) catalyzed stereoconvergent amination reaction with **1i** (5.0 mM, OD_{600} = 30) under standard conditions:

Entry	Pdt	IS	Pdt/IS	[Pdt]/mM	Yield [%]	Avg. Yield [%]	SD Yield [%]
1	76.8	99.9	0.769	0.143	2.86	3	0.1
2	78.8	95.6	0.824	0.153	3.06		
3	75.7	95.1	0.796	0.148	2.96		

1-(3-Chlorophenyl)-2-(methylamino)butan-1-one (3j)

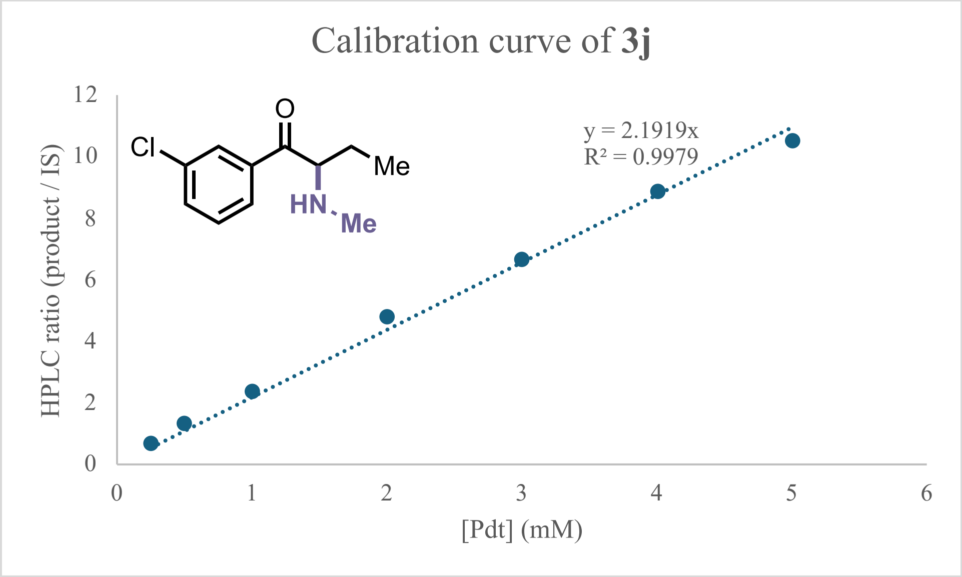

Data analysis for ***pa*Pgb-AKS-(*S*)-G3-5332** (M9-N, pH = **8.0**) catalyzed stereoconvergent amination reaction with **1j** (5.0 mM, OD_{600} = 30) under standard conditions:

Entry	Pdt	IS	Pdt/IS	[Pdt]/mM	Yield [%]	Avg. Yield [%]	SD Yield [%]
1	657.6	105.5	6.23	2.84	56.9	54	2.7
2	613.6	108.8	5.64	2.57	51.5		
3	634.3	107.6	5.89	2.69	53.8		

Data analysis for ***pa*Pgb-AKS-(*R*)-G3-5335** (M9-N, pH = **8.0**) catalyzed stereoconvergent amination reaction with **1j** (5.0 mM, OD_{600} = 30) under standard conditions:

Entry	Pdt	IS	Pdt/IS	[Pdt]/mM	Yield [%]	Avg. Yield [%]	SD Yield [%]
1	555.4	107.9	5.15	2.35	47.0	48	0.9
2	578.7	108.7	5.32	2.43	48.6		
3	560.8	108.9	5.15	2.35	47.0		

2-(Methylamino)-1-(*o*-tolyl)butan-1-one (3k)

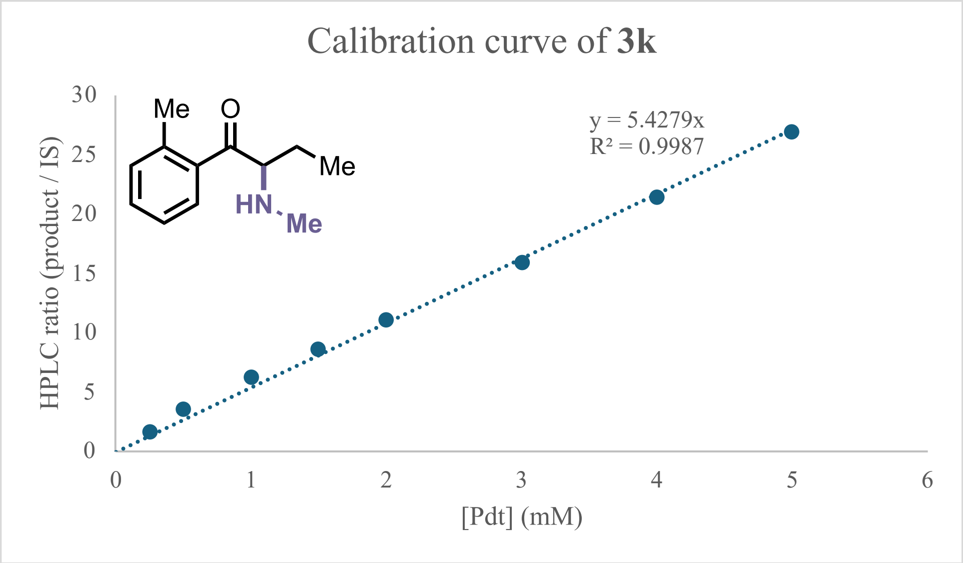

Data analysis for ***pa*Pgb-AKS-(*S*)-G3-5332** (M9-N, pH = **8.0**) catalyzed stereoconvergent amination reaction with **1k** (5.0 mM, OD_{600} = 30) under standard conditions:

Entry	Pdt	IS	Pdt/IS	[Pdt]/mM	Yield [%]	Avg. Yield [%]	SD Yield [%]
1	39.2	108.0	0.363	0.0669	1.34	1.4	0.05
2	41.1	107.9	0.381	0.0701	1.40		
3	38.7	108.4	0.357	0.0658	1.32		

Data analysis for ***pa*Pgb-AKS-(*R*)-G3-5335** (M9-N, pH = **8.0**) catalyzed stereoconvergent amination reaction with **1k** (5.0 mM, OD_{600} = 30) under standard conditions:

Entry	Pdt	IS	Pdt/IS	[Pdt]/mM	Yield [%]	Avg. Yield [%]	SD Yield [%]
1	27.5	113.0	0.243	0.0448	0.90	0.9	0.01
2	27.4	112.5	0.244	0.0449	0.90		
3	27.2	109.8	0.248	0.0456	0.91		

2-(Methylamino)-1-(naphthalen-2-yl)butan-1-one (3l)

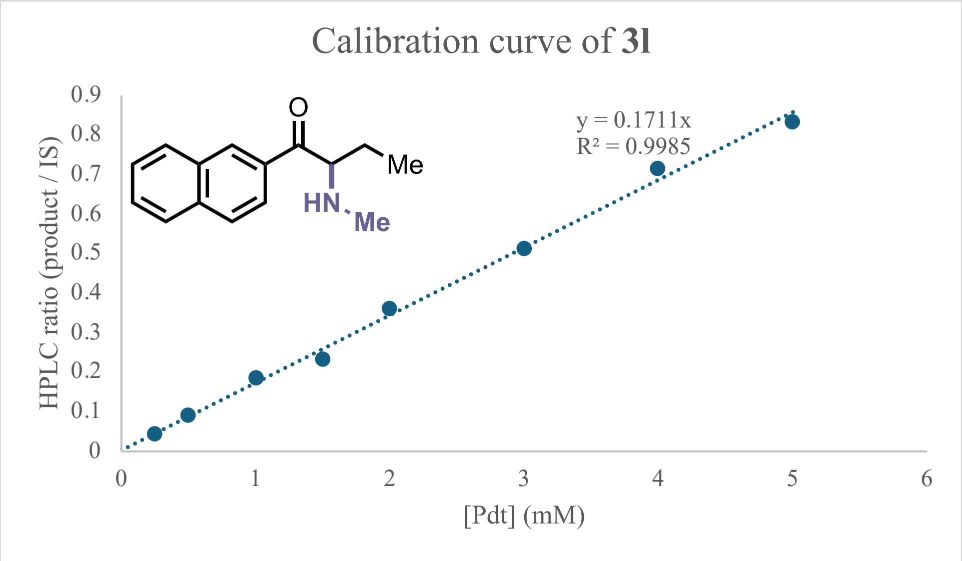

Data analysis for ***pa*Pgb-AKS-(S)-G3-5332** (M9-N, pH = **8.0**) catalyzed stereoconvergent amination reaction with **1l** (5.0 mM, OD_{600} = 30) under standard conditions:

Entry	Pdt	IS	Pdt/IS	[Pdt]/mM	Yield [%]	Avg. Yield [%]	SD Yield [%]
1	775.1	6641.3	0.117	0.682	13.6	13	0.3
2	763.8	6768.5	0.113	0.660	13.2		
3	763.6	6580.4	0.116	0.678	13.6		

Data analysis for ***pa*Pgb-AKS-(R)-G3-5335** (M9-N, pH = **8.0**) catalyzed stereoconvergent amination reaction with **1l** (5.0 mM, OD_{600} = 30) under standard conditions:

Entry	Pdt	IS	Pdt/IS	[Pdt]/mM	Yield [%]	Avg. Yield [%]	SD Yield [%]
1	689.1	6419.8	0.107	0.627	12.5	13	0.1
2	697.8	6420.6	0.109	0.635	12.7		
3	662.1	6378	0.104	0.607	12.1		

2-(Methylamino)-1-(thiophen-2-yl)butan-1-one (3m)

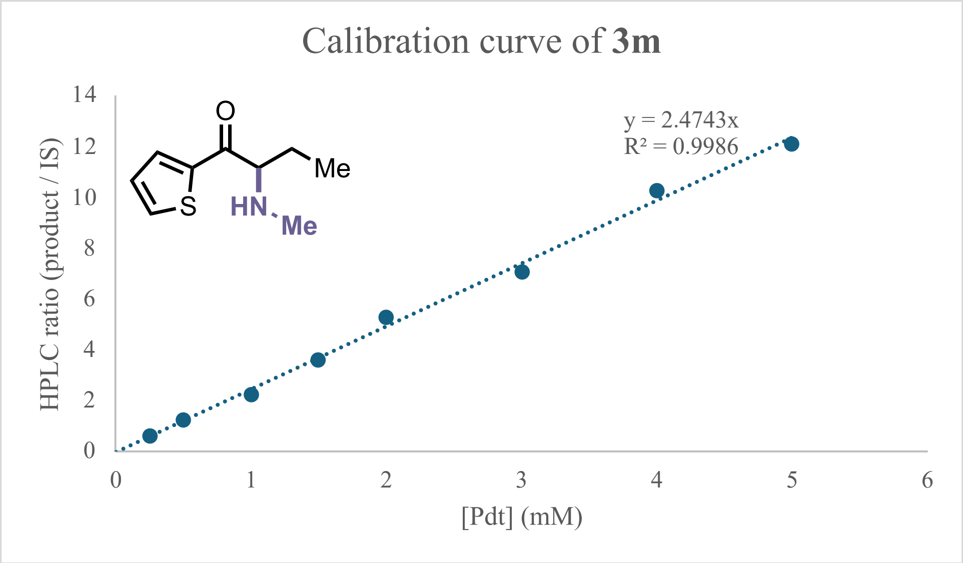

Data analysis for ***pa*Pgb-AKS-(*S*)-G3-5332** (M9-N, pH = **8.0**) catalyzed stereoconvergent amination reaction with **1m** (5.0 mM, OD_{600} = 30) under standard conditions:

Entry	Pdt	IS	Pdt/IS	[Pdt]/mM	Yield [%]	Avg. Yield [%]	SD Yield [%]
1	802.9	106.2	7.56	3.06	61.1	60	0.6
2	786.3	106.1	7.41	3.00	59.9		
3	792.8	106.5	7.44	3.01	60.2		

Data analysis for ***pa*Pgb-AKS-(*R*)-G3-5335** (M9-N, pH = **8.0**) catalyzed stereoconvergent amination reaction with **1m** (5.0 mM, OD_{600} = 30) under standard conditions:

Entry	Pdt	IS	Pdt/IS	[Pdt]/mM	Yield [%]	Avg. Yield [%]	SD Yield [%]
1	753.0	109.4	6.88	2.78	55.6	56	1.0
2	765.3	109.2	7.01	2.83	56.6		
3	744.3	110.0	6.77	2.73	54.7		

2-(Ethylamino)-1-phenylbutan-1-one (3q)

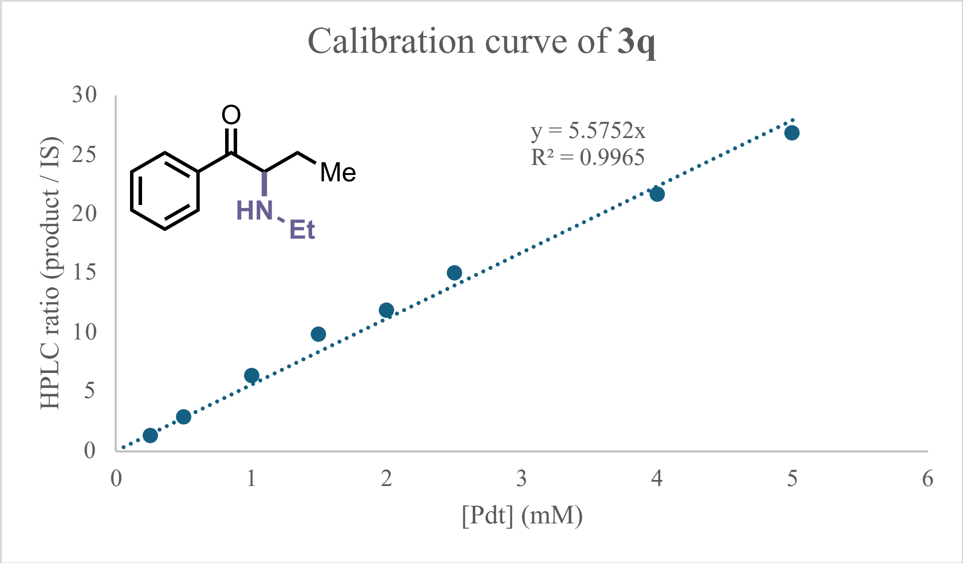

Data analysis for ***pa*****Pgb-AKS-(*****S*****)-G3-5332** (M9-N, pH = **8.0**) catalyzed stereoconvergent amination reaction with **1a** (5.0 mM, OD_{600} = 30) under standard conditions:

Entry	Pdt	IS	Pdt/IS	[Pdt]/mM	Yield [%]	Avg. Yield [%]	SD Yield [%]
1	961.9	107.3	8.96	1.61	32.2	32	0.3
2	958.2	108.0	8.87	1.59	31.8		
3	962.1	106.5	9.03	1.62	32.4		

Data analysis for ***pa*****Pgb-AKS-(*****R*****)-G3-5335** (M9-N, pH = **8.0**) catalyzed stereoconvergent amination reaction with **1a** (5.0 mM, OD_{600} = 30) under standard conditions:

Entry	Pdt	IS	Pdt/IS	[Pdt]/mM	Yield [%]	Avg. Yield [%]	SD Yield [%]
1	166.5	108.0	1.54	0.277	5.5	6	0.01
2	167.1	107.9	1.55	0.278	5.6		
3	166.3	107.9	1.54	0.276	5.5		

VII. Preparative-Scale Enzymatic Synthesis

General procedure for preparative-scale enzymatic intermolecular, stereoconvergent amination with 1a and 2d:

A suspension of *E. coli* expressing variant ***pa*Pgb-(*S*)-G3-5332** (OD_{600} = 30) in M9-N (pH = 8.0) was lysed following sonication methods in **Section I, General Procedure C**. In a 500-mL flask, 180 mL of the lysate, 10 mL trimethyl((1-phenylbut-1-en-1-yl)oxy)silane **1a** (300 mg, 1.36 mmol in EtOH), and 10 mL *N*-methylhydroxylamine **2d** (227 mg in M9-N, pH 8.0) were added sequentially under anaerobic conditions. The flask was capped and sealed with parafilm inside the anaerobic chamber. The mixture was shaken at 250 rpm in a shaker inside of an anaerobic chamber for 1 h followed by adding another 10 mL *N*-methylhydroxylamine **2d** (227 mg in M9-N, pH 8.0).

After 2 h, the reaction mixture was quenched by 400 mL acetonitrile and transferred to a centrifuge bucket to remove all the cell debris. The supernatant was collected in a 1-L flask. The mixture was concentrated under reduced pressure to remove majority of the acetonitrile. The remaining aqueous mixture was added 1M HCl to pH 2.0. The mixture was washed with ethyl acetate (200 mL × 4). The aqueous phase was added 1M NaOH solution to pH 10.0 and then extracted with ethyl acetate (100 mL × 3). The organic layer was combined and dried over Na_2SO_4. The combined organic solution was added 4N HCl in dioxane. The organic solvent was removed under reduced pressure to yield **3a** as a yellow solid.

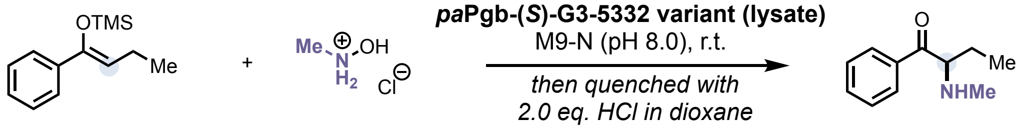

1a 300 mg, 1.36 mmol | **2d** | **3a** 210 mg, 72% yield

General procedure for preparative-scale enzymatic intermolecular, stereoconvergent amination with 1 and 2d:

A suspension of *E. coli* expressing variant ***pa*Pgb-(*S*)-G3-5332** (OD_{600} = 30) in M9-N (pH = 8.0) was lysed following sonication methods in **Section I, General Procedure C**. In a 500-mL flask, 140 mL of the lysate, 10 mL silyl enol ether **1** (0.8 mmol in EtOH), and 10 mL *N*-methylhydroxylamine **2d** (2.0 equiv. in M9-N, pH 8.0) were added sequentially under anaerobic conditions. The flask was capped and sealed with parafilm inside the anaerobic chamber. The mixture was shaken at 250 rpm in a shaker inside of an anaerobic chamber for 1 h followed by adding another 10 mL *N*-methylhydroxylamine **2d** (2.0 equiv. in M9-N, pH 8.0).

After 2 h, the reaction mixture was quenched by 400 mL acetonitrile and transferred to a centrifuge bucket to remove all the cell debris. The supernatant was collected in a 1-L flask. The mixture was concentrated under reduced pressure to remove majority of the acetonitrile. The remaining aqueous mixture was added 1M HCl to pH 2.0. The mixture was washed with ethyl acetate (200 mL × 4). The aqueous phase was added 1M NaOH solution to pH 10.0 and then extracted with ethyl acetate (100 mL × 3). The organic layer was combined and dried over Na_2SO_4.

The combined organic solution was added 4N HCl in dioxane. The organic solvent was removed under reduced pressure to yield **3** as a yellow solid.

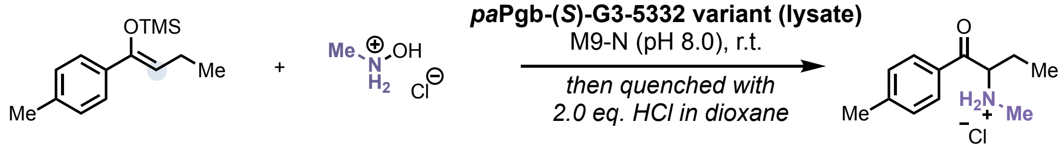

1f 188 mg, 0.8 mmol | **2d** | **3f** 50 mg, 28% yield

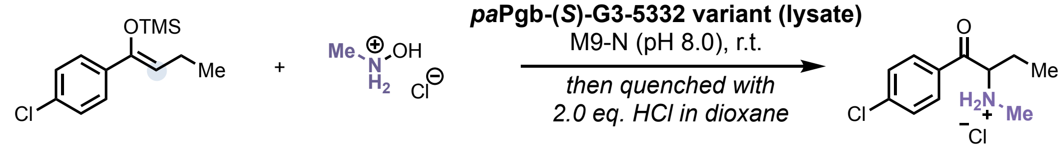

1h 204 mg, 0.8 mmol | **2d** | **3h** 81 mg, 41% yield

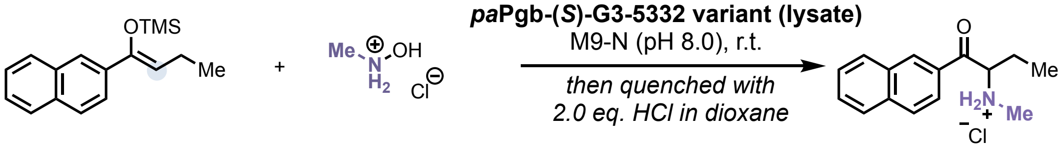

1l 216 mg, 0.8 mmol | **2d** | **3l** 62 mg, 29% yield

VIII. Chiral Polar Protic HPLC Traces

The absolute stereochemistry for enzymatic products **3j** generated using ***pa*Pgb-AKS-(*S*)-G3-5332** were assigned to be *S,* by comparing with a chemically synthesized optical pure standard product. All other products were assigned by analogy.

Determination of stereochemistry for all products:

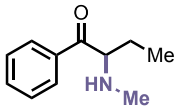

2-(methylamino)-1-phenylbutan-1-one (3a)
Chiral polar protic HPLC conditions: Angilent Poroshell 120 Chiral-V, 2.7 µm, 2.1 × 150 mm, 95:5 EtOH:water (20 mM ammonium formate, pH 4.0), 0.5 mL/min, 25 ºC, 254 nm

Racemic 3a:

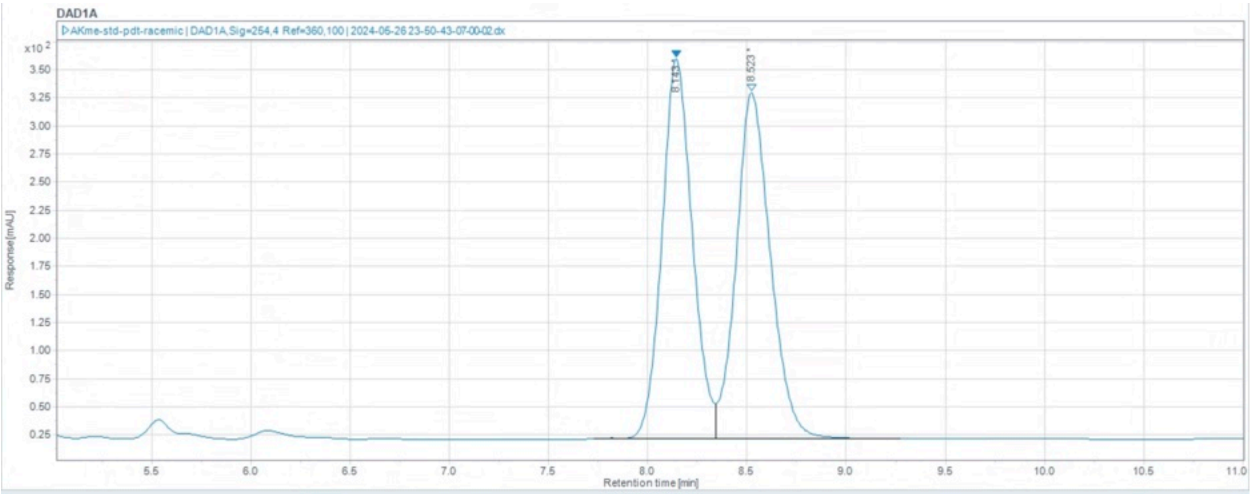

Injection Results

Peaks | Summary

#	Name	Signal description	RT (min)	Area (mAU·s)	Area%	Height (mAU)	Height%	Amount	Concentration	Start time (min)	End time (min)
1		DAD1A,Sig=254,4 Ref=360,100	8.143	3604.118	49.287	338.129	52.29			7.727	8.341
2		DAD1A,Sig=254,4 Ref=360,100	8.523	3708.366	50.713	308.509	47.71			8.341	9.274

Enzymatic preparation of 3a with *pa*Pgb-AKS-G0-5329: –73% *ee*

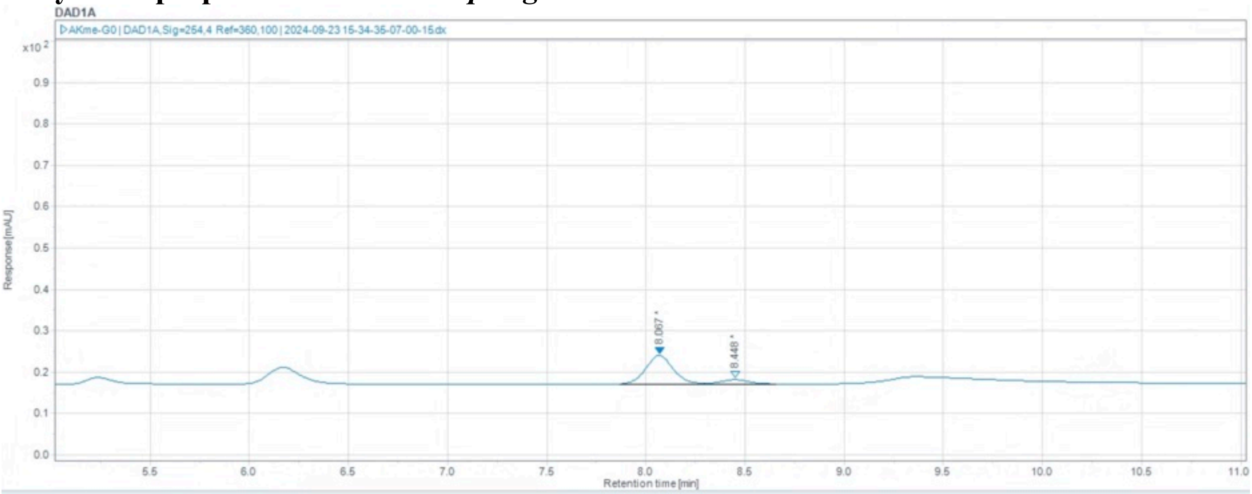

Injection Results

Peaks | Summary

#	Name	Signal description	RT (min)	Area (mAU·s)	Area%	Height (mAU)	Height%	Amount	Concentration	Start time (min)	End time (min)
1		DAD1A,Sig=254,4 Ref=360,100	8.067	65.898	86.631	6.901	86.84			7.869	8.294
2		DAD1A,Sig=254,4 Ref=360,100	8.448	10.169	13.369	1.046	13.16			8.294	8.654

Enzymatic preparation of 3a with *pa*Pgb-AKS-(S)-G1-5330: 60% *ee*

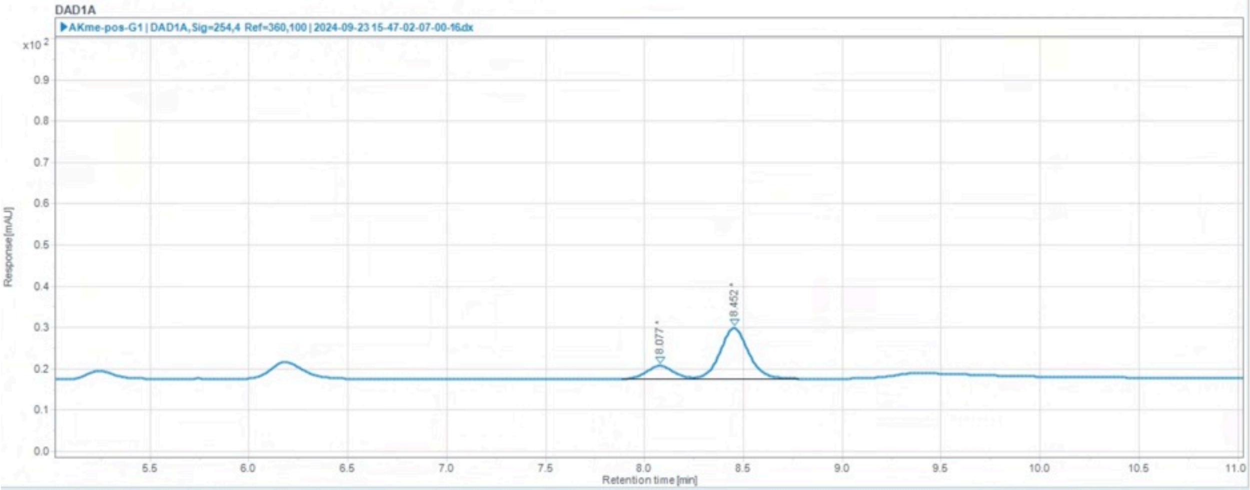

Injection Results

Peaks | Summary

#	Name	Signal description	RT (min)	Area (mAU·s)	Area%	Height (mAU)	Height%	Amount	Concentration	Start time (min)	End time (min)
1		DAD1A,Sig=254,4 Ref=360,100	8.077	30.412	19.848	3.220	20.74			7.881	8.251
2		DAD1A,Sig=254,4 Ref=360,100	8.452	122.814	80.152	12.305	79.26			8.251	8.781

Enzymatic preparation of 3a with *pa*Pgb-AKS-(S)-G2-5331: 91% *ee*

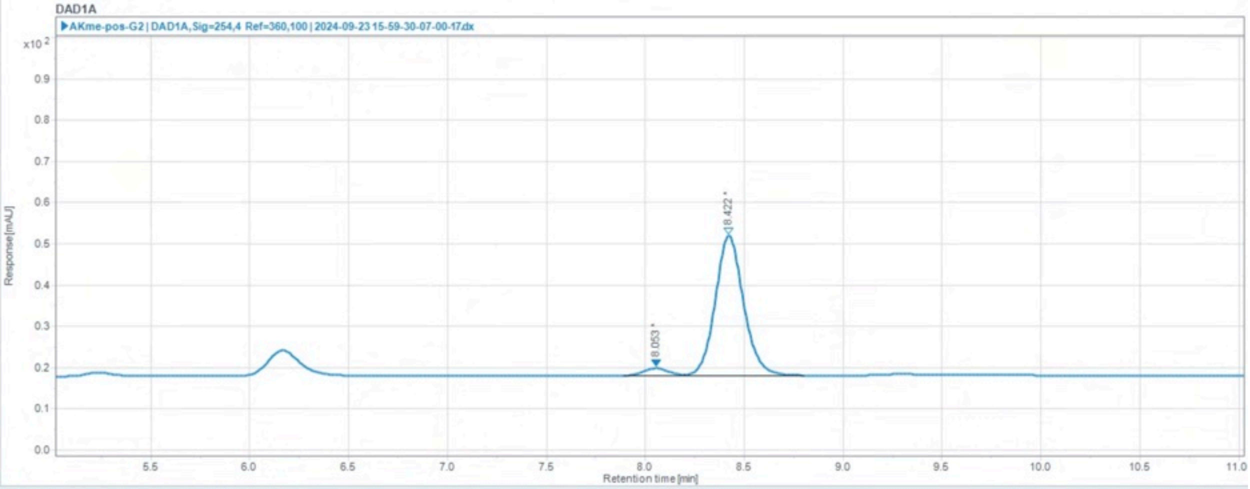

Injection Results

Peaks | Summary

#	Name	Signal description	RT (min)	Area (mAU·s)	Area%	Height (mAU)	Height%	Amount	Concentration	Start time (min)	End time (min)
1		DAD1A,Sig=254,4 Ref=360,100	8.053	16.285	4.592	1.822	5.09			7.890	8.199
2		DAD1A,Sig=254,4 Ref=360,100	8.422	338.318	95.408	33.945	94.91			8.199	8.798

Enzymatic preparation of 3a with *pa*Pgb-AKS-(S)-G3-5332: 94% *ee*

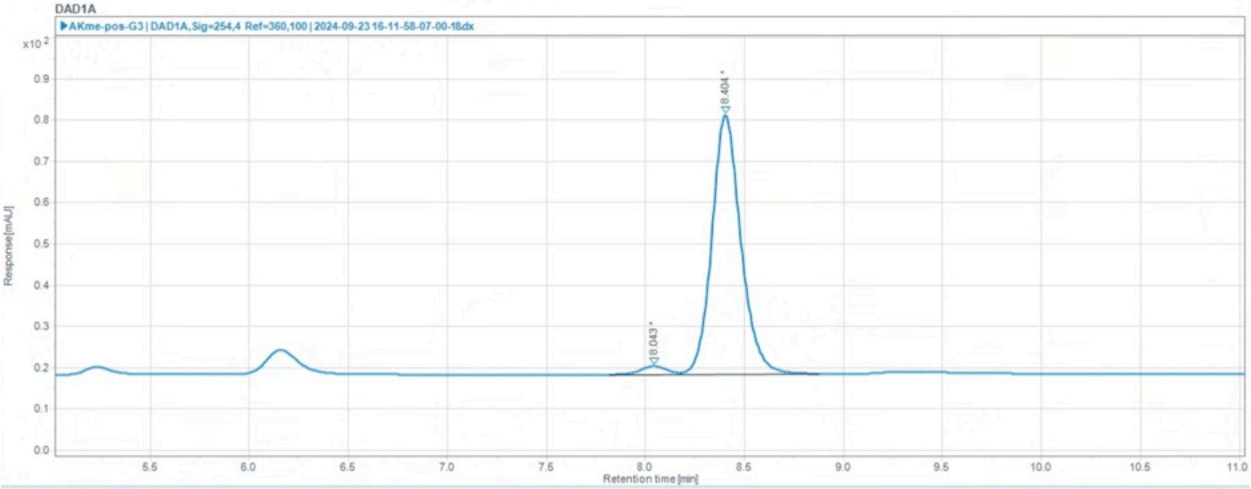

Injection Results

Peaks | Summary

#	Name	Signal description	RT (min)	Area (mAU·s)	Area%	Height (mAU)	Height%	Amount	Concentration	Start time (min)	End time (min)
1		DAD1A,Sig=254,4 Ref=360,100	8.043	18.273	2.785	1.991	3.08			7.818	8.177
2		DAD1A,Sig=254,4 Ref=360,100	8.404	637.916	97.215	62.696	96.92			8.177	8.877

Enzymatic preparation of 3a with *pa*Pgb-AKS-(R)-G1-5333: –89% *ee*

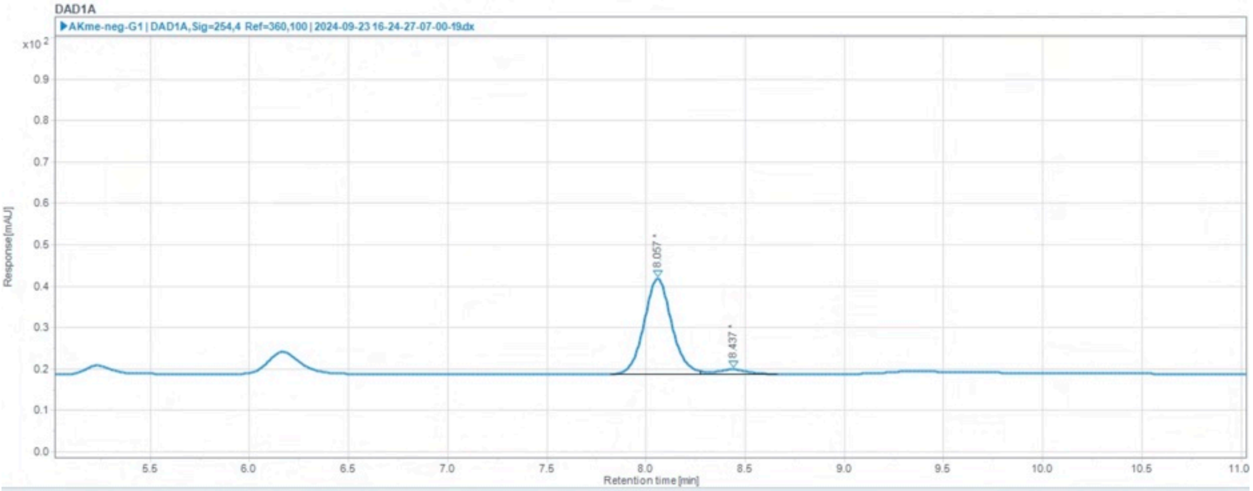

Injection Results

Peaks | Summary

#	Name	Signal description	RT (min)	Area (mAU·s)	Area%	Height (mAU)	Height%	Amount	Concentration	Start time (min)	End time (min)
1		DAD1A,Sig=254,4 Ref=360,100	8.057	222.951	94.695	23.166	95.43			7.823	8.275
2		DAD1A,Sig=254,4 Ref=360,100	8.437	12.491	5.305	1.109	4.57			8.275	8.663

Enzymatic preparation of 3a with *pa*Pgb-AKS-(R)-G2-5334: –92% *ee*

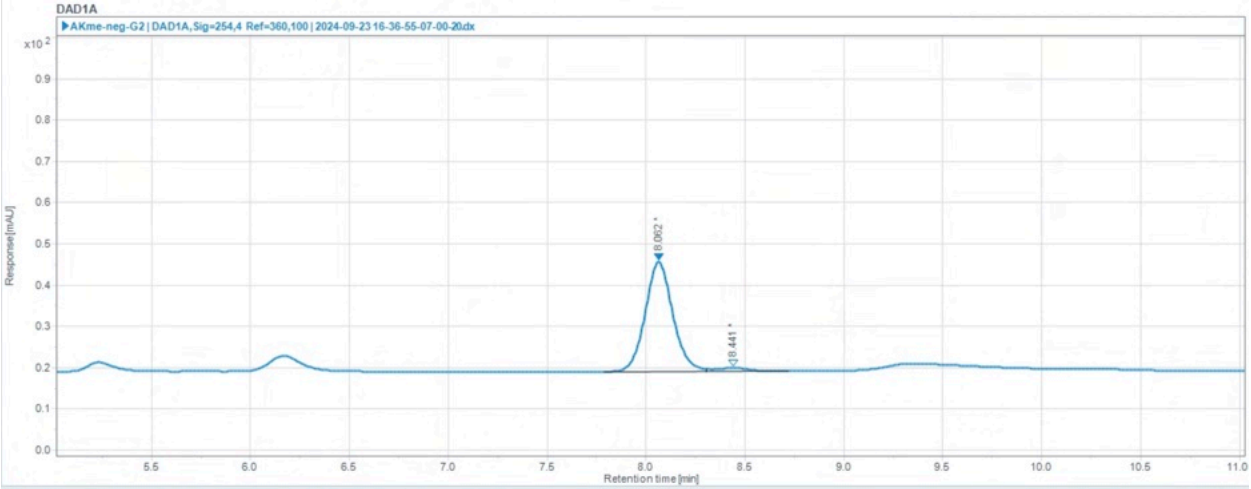

Injection Results

Peaks | Summary

#	Name	Signal description	RT (min)	Area (mAU·s)	Area%	Height (mAU)	Height%	Amount	Concentration	Start time (min)	End time (min)
1		DAD1A,Sig=254,4 Ref=360,100	8.062	257.597	96.353	26.549	96.88			7.789	8.306
2		DAD1A,Sig=254,4 Ref=360,100	8.441	9.750	3.647	0.856	3.12			8.306	8.720

Enzymatic preparation of 3a with *pa*Pgb-AKS-(R)-G3-5335: –92% *ee*

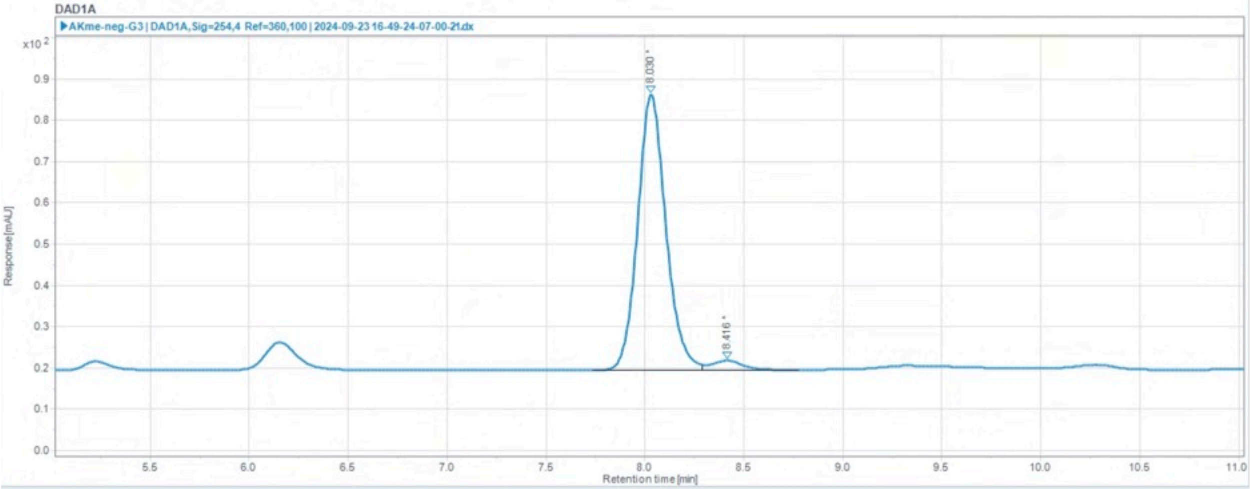

Injection Results

Peaks | Summary

#	Name	Signal description	RT (min)	Area (mAU·s)	Area%	Height (mAU)	Height%	Amount	Concentration	Start time (min)	End time (min)
1		DAD1A,Sig=254,4 Ref=360,100	8.030	660.485	96.191	66.867	96.62			7.740	8.290
2		DAD1A,Sig=254,4 Ref=360,100	8.416	26.157	3.809	2.338	3.38			8.290	8.776

Substrates with pure diastereomers:

Enzymatic preparation of 3a from (*Z*)-1a with *pa*Pgb-AKS-(S)-G3-5332: 90% *ee*

Chiral polar protic HPLC conditions: Angilent Poroshell 120 Chiral-V, 2.7 µm, 2.1 × 150 mm, 90:10 EtOH:water (20 mM ammonium formate, pH 4.0), 0.5 mL/min, 25 ºC, 254 nm

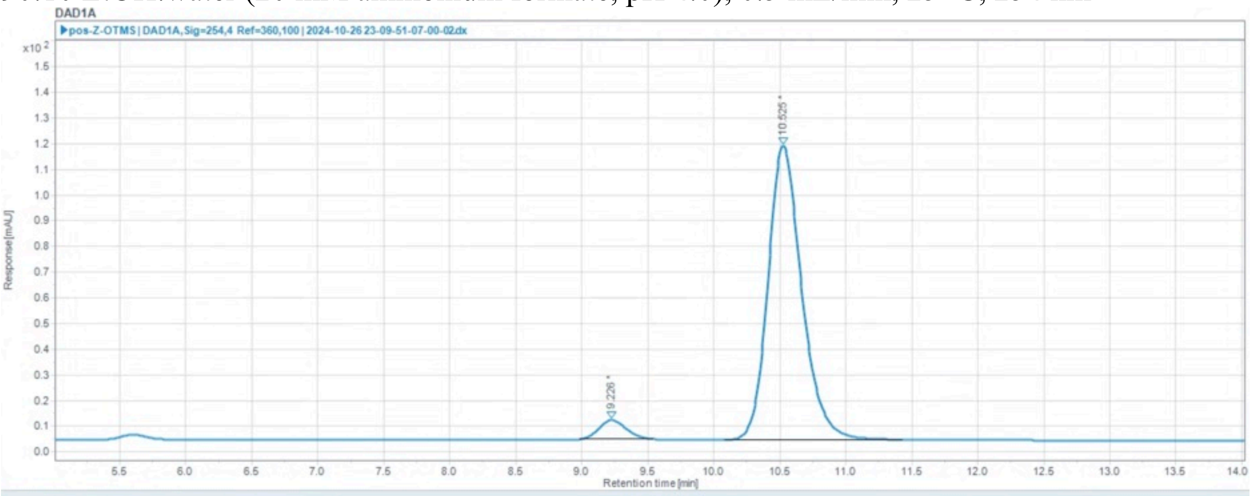

Injection Results

Peaks | Summary

#	Name	Signal description	RT (min)	Area (mAU·s)	Area%	Height (mAU)	Height%	Amount	Concentration	Start time (min)	End time (min)
1		DAD1A,Sig=254,4 Ref=360,100	9.226	105.580	4.929	7.410	6.07			8.987	9.536
2		DAD1A,Sig=254,4 Ref=360,100	10.525	2036.523	95.071	114.570	93.93			10.085	11.432

Enzymatic preparation of 3a from (*Z*)-1a with *pa*Pgb-AKS-(R)-G3-5335: –92% *ee*

Chiral polar protic HPLC conditions: Angilent Poroshell 120 Chiral-V, 2.7 µm, 2.1 × 150 mm, 90:10 EtOH:water (20 mM ammonium formate, pH 4.0), 0.5 mL/min, 25 ºC, 254 nm

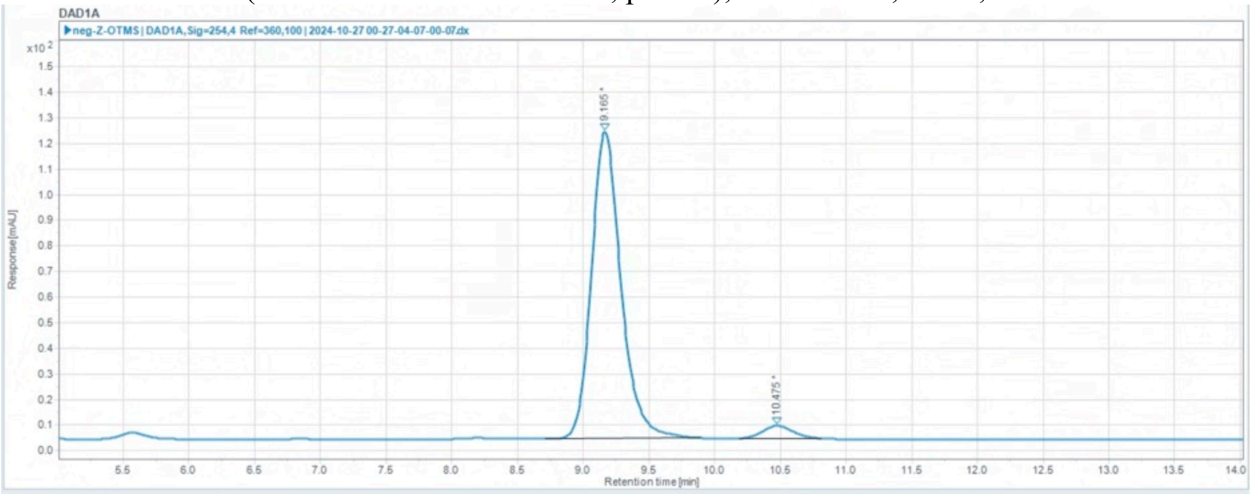

Injection Results

Peaks | Summary

#	Name	Signal description	RT (min)	Area (mAU·s)	Area%	Height (mAU)	Height%	Amount	Concentration	Start time (min)	End time (min)
1		DAD1A,Sig=254,4 Ref=360,100	9.165	1876.970	95.944	120.006	96.06			8.711	9.895
2		DAD1A,Sig=254,4 Ref=360,100	10.475	79.354	4.056	4.928	3.94			10.188	10.814

Enzymatic preparation of 3a from (*E*)-1a with *pa*Pgb-AKS-(S)-G3-5332: 90% *ee*

Chiral polar protic HPLC conditions: Angilent Poroshell 120 Chiral-V, 2.7 µm, 2.1 × 150 mm, 90:10 EtOH:water (20 mM ammonium formate, pH 4.0), 0.5 mL/min, 25 ºC, 254 nm

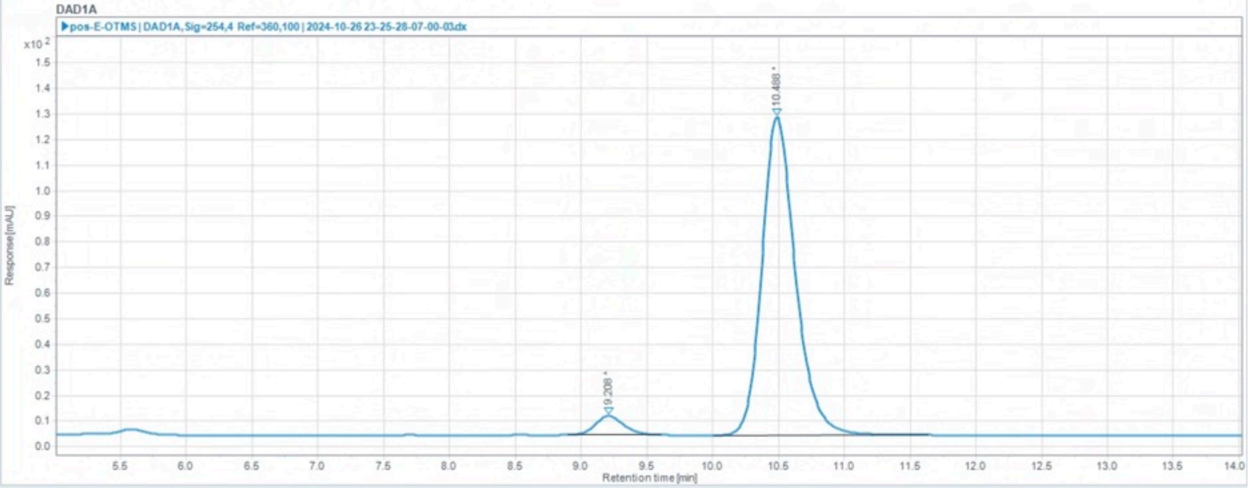

Injection Results

Peaks | Summary

#	Name	Signal description	RT (min)	Area (mAU·s)	Area%	Height (mAU)	Height%	Amount	Concentration	Start time (min)	End time (min)
1		DAD1A,Sig=254,4 Ref=360,100	9.208	111.476	4.810	7.421	5.63			8.902	9.610
2		DAD1A,Sig=254,4 Ref=360,100	10.488	2206.150	95.190	124.324	94.37			10.007	11.638

Enzymatic preparation of 3a from (*E*)-1a with *pa*Pgb-AKS-(R)-G3-5335: –91% *ee*

Chiral polar protic HPLC conditions: Angilent Poroshell 120 Chiral-V, 2.7 µm, 2.1 × 150 mm, 90:10 EtOH:water (20 mM ammonium formate, pH 4.0), 0.5 mL/min, 25 ºC, 254 nm

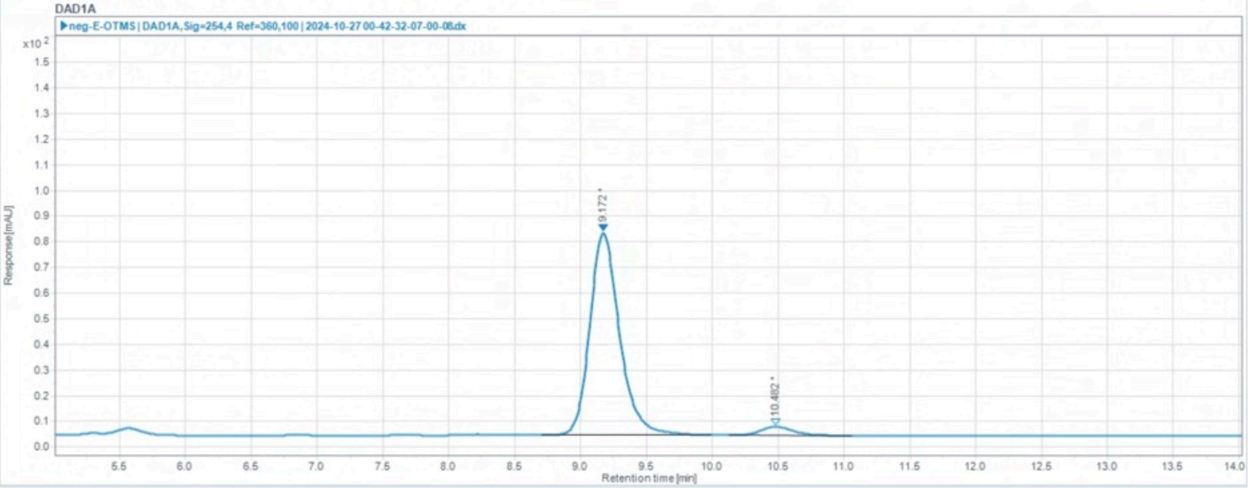

Injection Results

Peaks | Summary

#	Name	Signal description	RT (min)	Area (mAU·s)	Area%	Height (mAU)	Height%	Amount	Concentration	Start time (min)	End time (min)
1		DAD1A,Sig=254,4 Ref=360,100	9.172	1235.314	95.663	78.774	95.92			8.705	9.991
2		DAD1A,Sig=254,4 Ref=360,100	10.482	55.998	4.337	3.350	4.08			10.128	11.058

<u>Substrates with different silyl groups:</u>

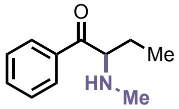

2-(methylamino)-1-phenylbutan-1-one (3a)

Chiral polar protic HPLC conditions: Angilent Poroshell 120 Chiral-V, 2.7 µm, 2.1 × 150 mm, 95:5 EtOH:water (20 mM ammonium formate, pH 4.0), 0.5 mL/min, 25 ºC, 254 nm

Enzymatic preparation of 3a from 1n with *pa*Pgb-AKS-(S)-G3-5332: 88% *ee*

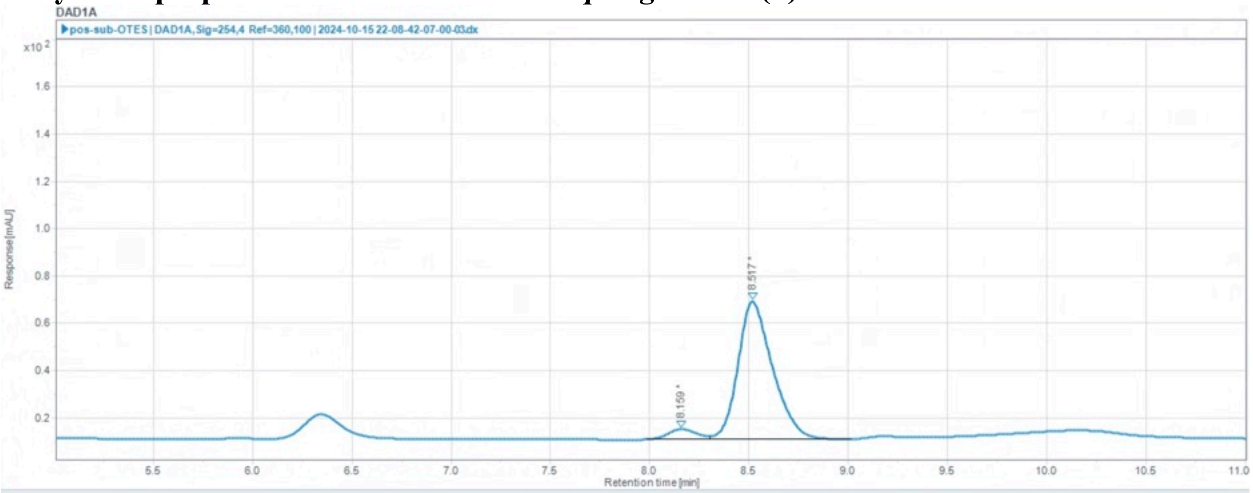

Injection Results

Peaks | Summary

#	Name	Signal description	RT (min)	Area (mAU·s)	Area%	Height (mAU)	Height%	Amount	Concentration	Start time (min)	End time (min)
1		DAD1A,Sig=254,4 Ref=360,100	8.159	43.502	5.803	4.366	6.97			7.984	8.303
2		DAD1A,Sig=254,4 Ref=360,100	8.517	706.205	94.197	58.272	93.03			8.303	9.015

Enzymatic preparation of 3a from 1n with *pa*Pgb-AKS-(R)-G3-5335: –89% *ee*

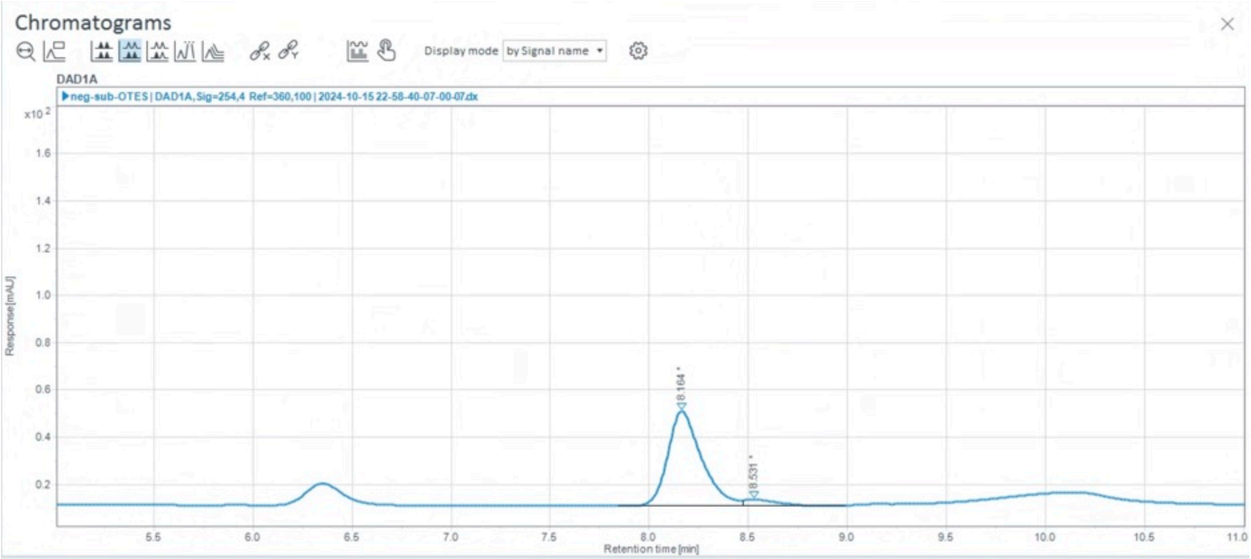

Injection Results

Peaks | Summary

#	Name	Signal description	RT (min)	Area (mAU·s)	Area%	Height (mAU)	Height%	Amount	Concentration	Start time (min)	End time (min)
1		DAD1A,Sig=254,4 Ref=360,100	8.164	475.889	94.518	40.102	93.98			7.842	8.475
2		DAD1A,Sig=254,4 Ref=360,100	8.531	27.599	5.482	2.568	6.02			8.475	8.990

Enzymatic preparation of 3a from 1o with *pa*Pgb-AKS-(S)-G3-5332: 59% *ee*

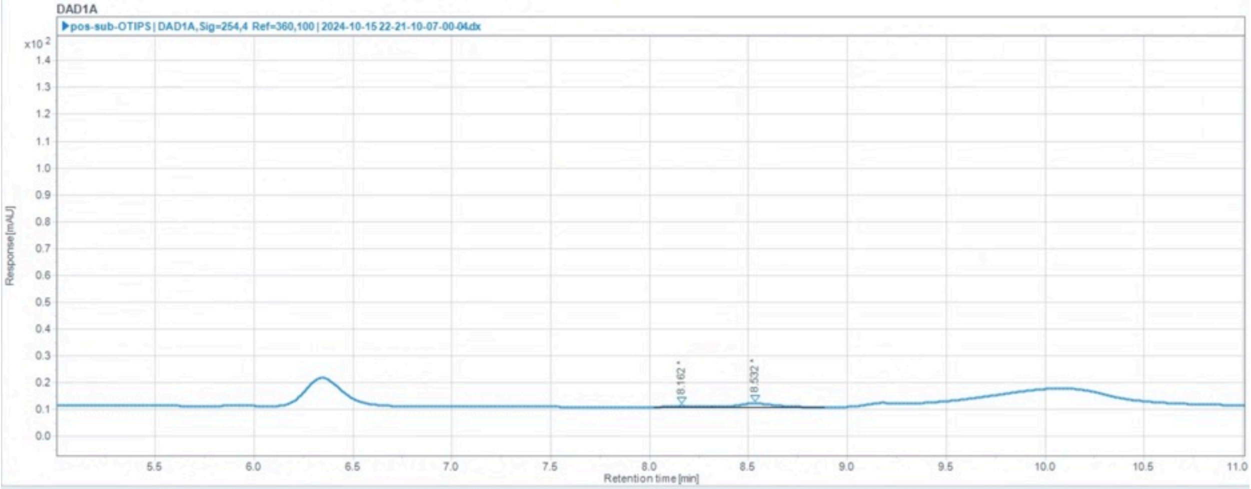

Injection Results

Peaks | Summary

#	Name	Signal description	RT (min)	Area (mAU·s)	Area%	Height (mAU)	Height%	Amount	Concentration	Start time (min)	End time (min)
1		DAD1A,Sig=254,4 Ref=360,100	8.162	4.470	20.678	0.466	25.14			8.023	8.317
2		DAD1A,Sig=254,4 Ref=360,100	8.532	17.149	79.322	1.387	74.86			8.317	8.884

Enzymatic preparation of 3a from 1o with *pa*Pgb-AKS-(R)-G3-5335: –81% *ee*

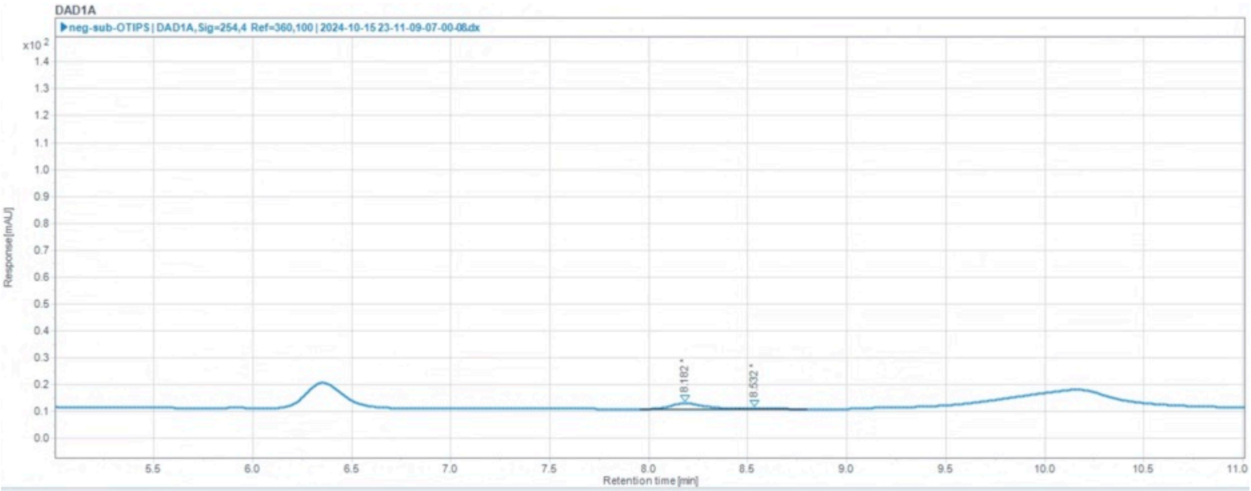

Injection Results

Peaks | Summary

#	Name	Signal description	RT (min)	Area (mAU·s)	Area%	Height (mAU)	Height%	Amount	Concentration	Start time (min)	End time (min)
1		DAD1A,Sig=254,4 Ref=360,100	8.182	25.910	90.687	2.176	90.72			7.957	8.458
2		DAD1A,Sig=254,4 Ref=360,100	8.532	2.661	9.313	0.223	9.28			8.458	8.800

Enzymatic preparation of 3a from 1p with *pa*Pgb-AKS-(S)-G3-5332: 58% *ee*

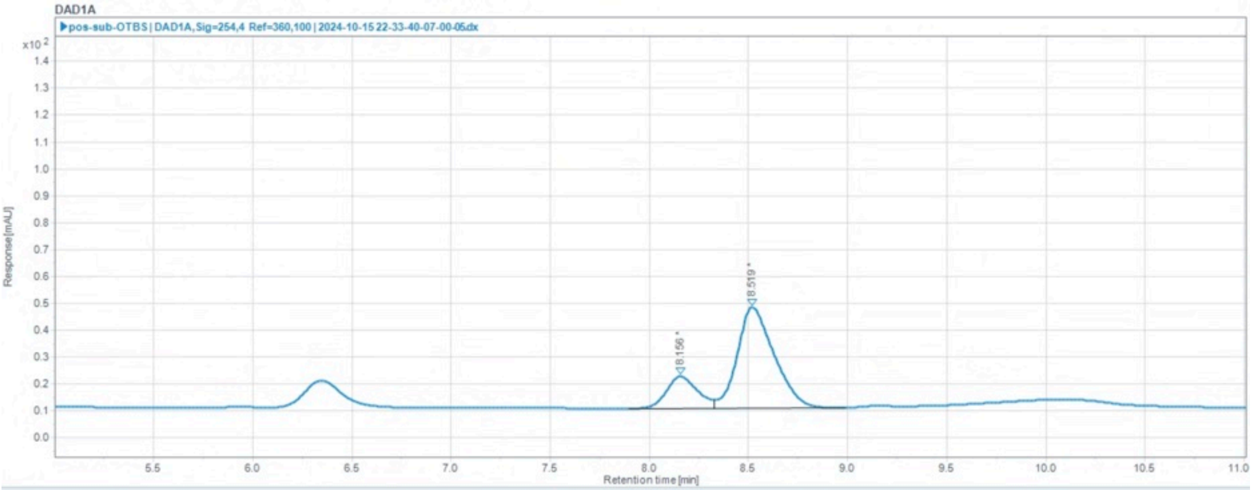

Injection Results

Peaks | Summary

#	Name	Signal description	RT (min)	Area (mAU·s)	Area%	Height (mAU)	Height%	Amount	Concentration	Start time (min)	End time (min)
1		DAD1A,Sig=254,4 Ref=360,100	8.156	129.746	21.078	11.927	24.09			7.898	8.328
2		DAD1A,Sig=254,4 Ref=360,100	8.519	485.800	78.922	37.585	75.91			8.328	8.990

Enzymatic preparation of 3a from 1p with *pa*Pgb-AKS-(R)-G3-5335: –88% *ee*

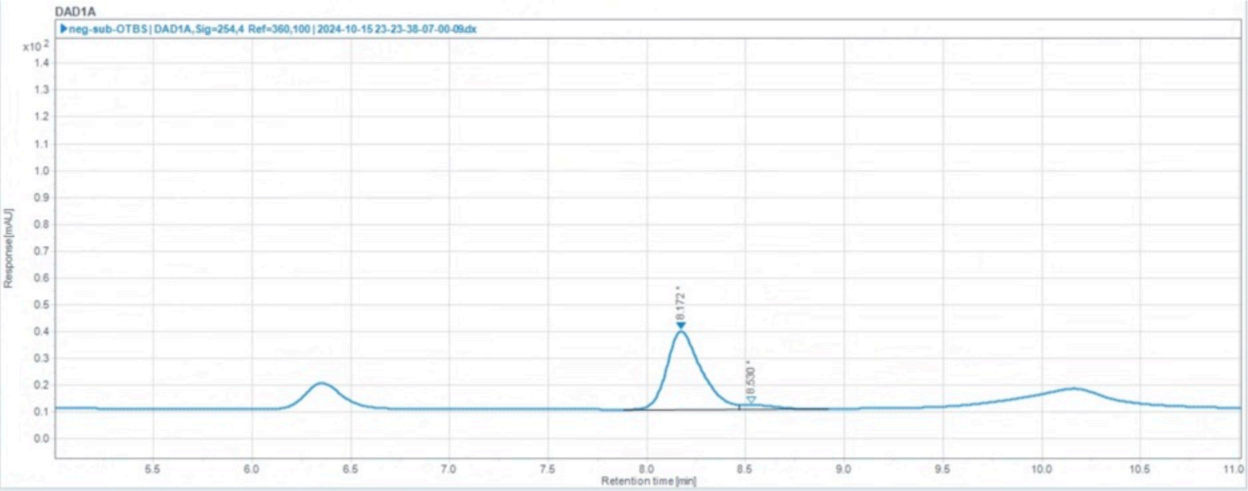

Injection Results

Peaks Summary

#	Name	Signal description	RT (min)	Area (mAU·s)	Area%	Height (mAU)	Height%	Amount	Concentration	Start time (min)	End time (min)
1		DAD1A,Sig=254,4 Ref=360,100	8.172	352.954	94.225	29.322	94.30			7.880	8.466
2		DAD1A,Sig=254,4 Ref=360,100	8.530	21.632	5.775	1.773	5.70			8.466	8.918

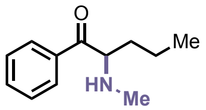

2-(methylamino)-1-phenylpentan-1-one (3c)
Chiral polar protic HPLC conditions: Angilent Poroshell 120 Chiral-V, 2.7 µm, 2.1 × 150 mm, 95:5 EtOH:water (20 mM ammonium formate, pH 4.0), 0.5 mL/min, 25 ºC, 254 nm

Racemic 3c:

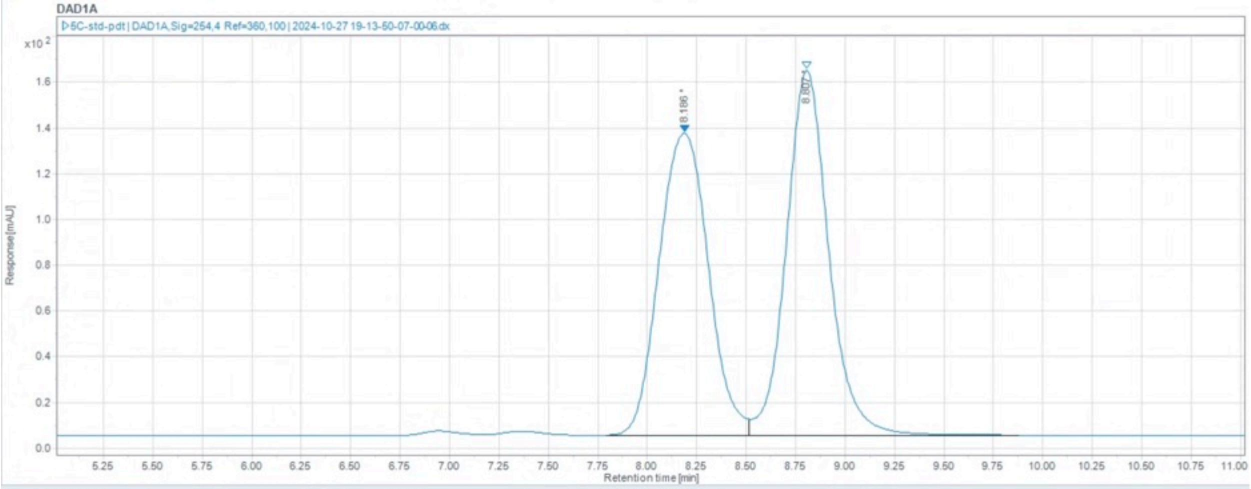

Injection Results

Peaks | Summary

#	Name	Signal description	RT (min)	Area (mAU·s)	Area%	Height (mAU)	Height%	Amount	Concentration	Start time (min)	End time (min)
1		DAD1A,Sig=254,4 Ref=360,100	8.186	2290.843	49.137	132.065	45.30			7.790	8.516
2		DAD1A,Sig=254,4 Ref=360,100	8.807	2371.309	50.863	159.469	54.70			8.516	9.880

Enzymatic preparation of 3c with *pa*Pgb-AKS-(S)-G3-5332: 87% *ee*

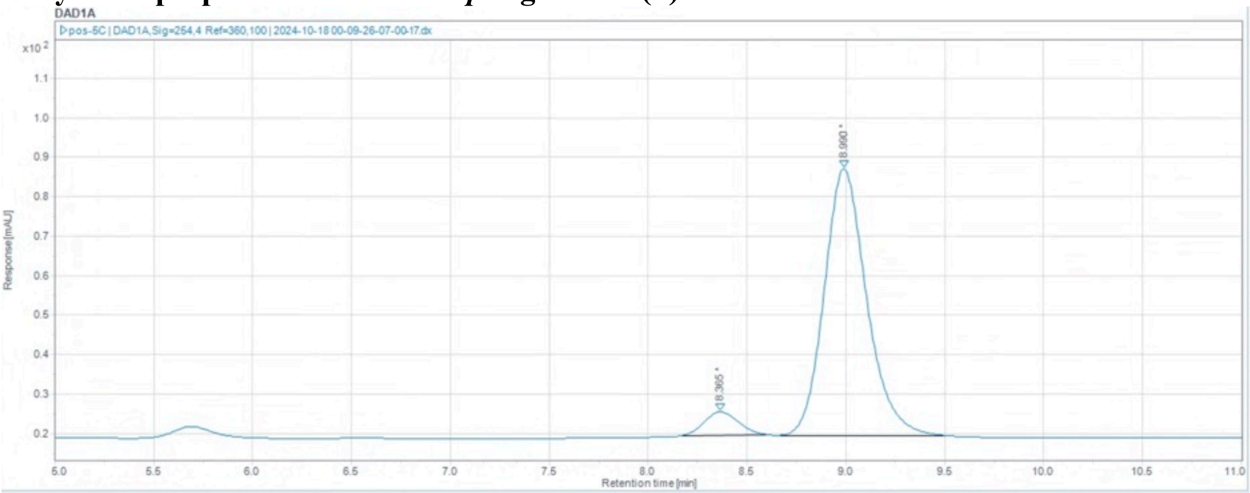

Injection Results

Peaks | Summary

#	Name	Signal description	RT (min)	Area (mAU·s)	Area%	Height (mAU)	Height%	Amount	Concentration	Start time (min)	End time (min)
1		DAD1A,Sig=254,4 Ref=360,100	8.365	69.368	6.421	5.833	7.94			8.173	8.592
2		DAD1A,Sig=254,4 Ref=360,100	8.990	1010.984	93.579	67.613	92.06			8.668	9.498

Enzymatic preparation of 3c with *pa*Pgb-AKS-(R)-G3-5335: –88% *ee*

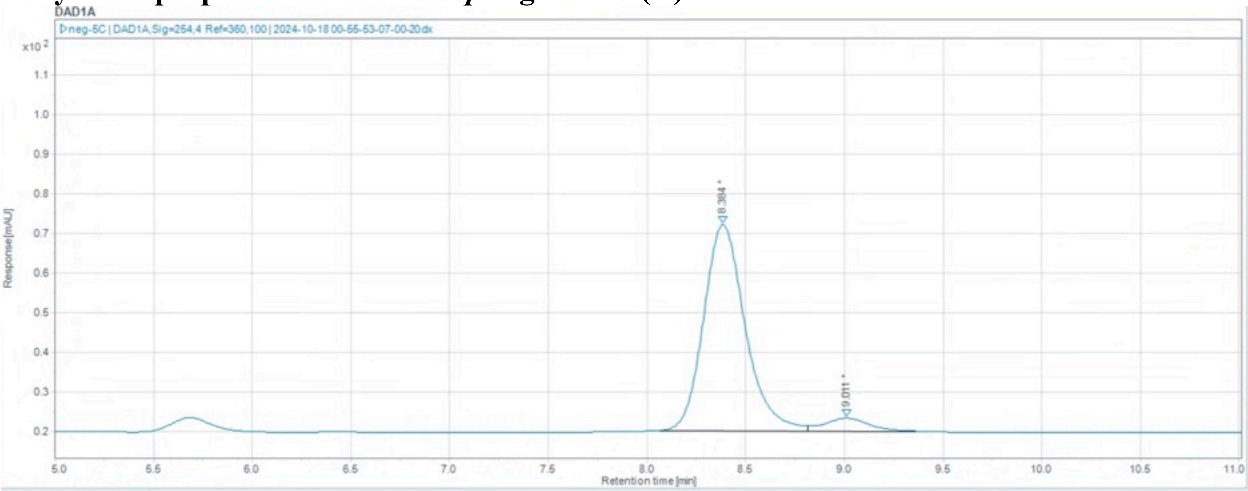

Injection Results

Peaks Summary

#	Name	Signal description	RT (min)	Area (mAU·s)	Area%	Height (mAU)	Height%	Amount	Concentration	Start time (min)	End time (min)
1		DAD1A,Sig=254,4 Ref=360,100	8.384	762.375	93.833	52.015	94.14			8.066	8.815
2		DAD1A,Sig=254,4 Ref=360,100	9.011	50.109	6.167	3.237	5.86			8.815	9.364

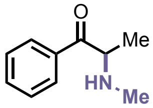

2-(methylamino)-1-phenylpropan-1-one (3d)

Chiral polar protic HPLC conditions: Angilent Poroshell 120 Chiral-V, 2.7 µm, 2.1 × 150 mm, 95:5 EtOH:water (20 mM ammonium formate, pH 4.0), 0.5 mL/min, 25 ºC, 254 nm

Racemic 3d:

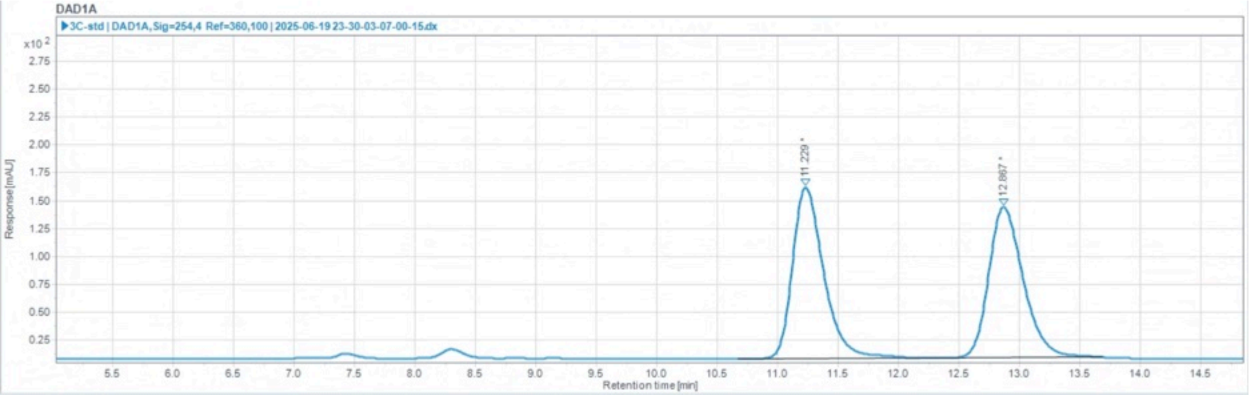

Injection Results

Peaks | Summary

#	Name	Signal description	RT (min)	Area (mAU·s)	Area%	Height (mAU)	Height%	Amount	Concentration	Start time (min)	End time (min)
1		DAD1A,Sig=254,4 Ref=360,100	11.229	2755.518	50.620	153.514	53.23			10.669	12.284
2		DAD1A,Sig=254,4 Ref=360,100	12.867	2687.969	49.380	134.891	46.77			12.284	13.685

Enzymatic preparation of 3d with *pa*Pgb-AKS-(*S*)-G3-5332: 44% *ee*

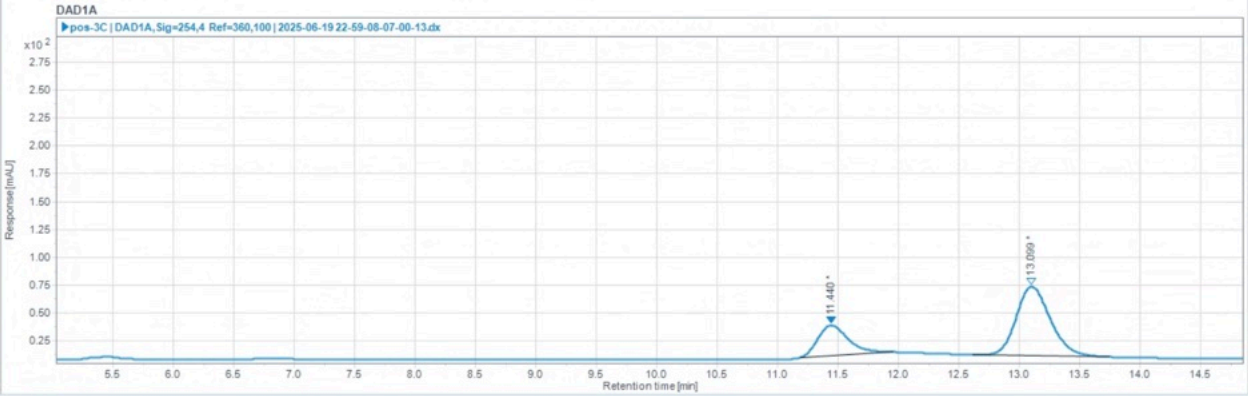

Injection Results

Peaks | Summary

#	Name	Signal description	RT (min)	Area (mAU·s)	Area%	Height (mAU)	Height%	Amount	Concentration	Start time (min)	End time (min)
1		DAD1A,Sig=254,4 Ref=360,100	11.440	480.141	28.194	27.203	30.50			11.187	11.954
2		DAD1A,Sig=254,4 Ref=360,100	13.099	1222.863	71.806	61.990	69.50			12.609	13.741

Enzymatic preparation of 3d with *pa*Pgb-AKS-(*R*)-G3-5335: –28% *ee*

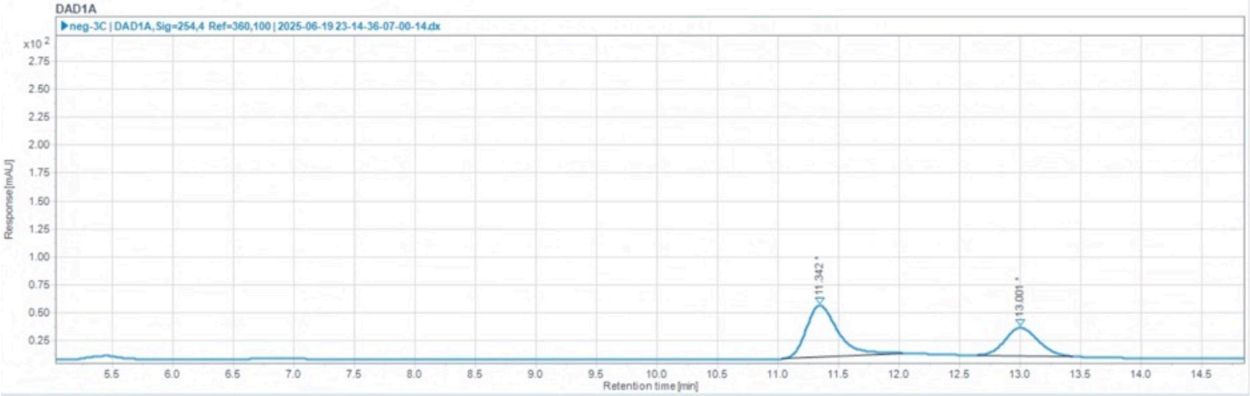

Injection Results

Peaks Summary

#	Name	Signal description	RT (min)	Area (mAU·s)	Area%	Height (mAU)	Height%	Amount	Concentration	Start time (min)	End time (min)
1		DAD1A,Sig=254,4 Ref=360,100	11.342	836.027	63.873	46.274	65.00			11.029	12.022
2		DAD1A,Sig=254,4 Ref=360,100	13.001	472.871	36.127	24.915	35.00			12.643	13.433

Chemical synthesis of (*S*)-3d

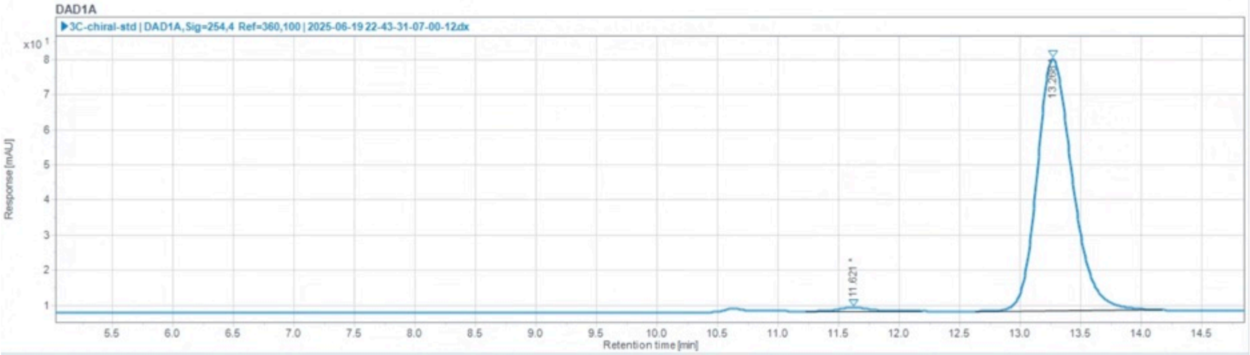

Injection Results

Peaks Summary

#	Name	Signal description	RT (min)	Area (mAU·s)	Area%	Height (mAU)	Height%	Amount	Concentration	Start time (min)	End time (min)
1		DAD1A,Sig=254,4 Ref=360,100	11.621	18.155	1.224	1.029	1.41			11.229	12.180
2		DAD1A,Sig=254,4 Ref=360,100	13.268	1465.490	98.776	71.829	98.59			12.631	14.170

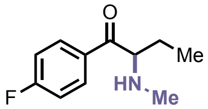

1-(4-Fluorophenyl)-2-(methylamino)butan-1-one (3e)
Chiral polar protic HPLC conditions: Angilent Poroshell 120 Chiral-V, 2.7 µm, 2.1 × 150 mm, 95:5 EtOH:water (20 mM ammonium formate, pH 4.0), 0.5 mL/min, 25 ºC, 254 nm

Racemic 3e:

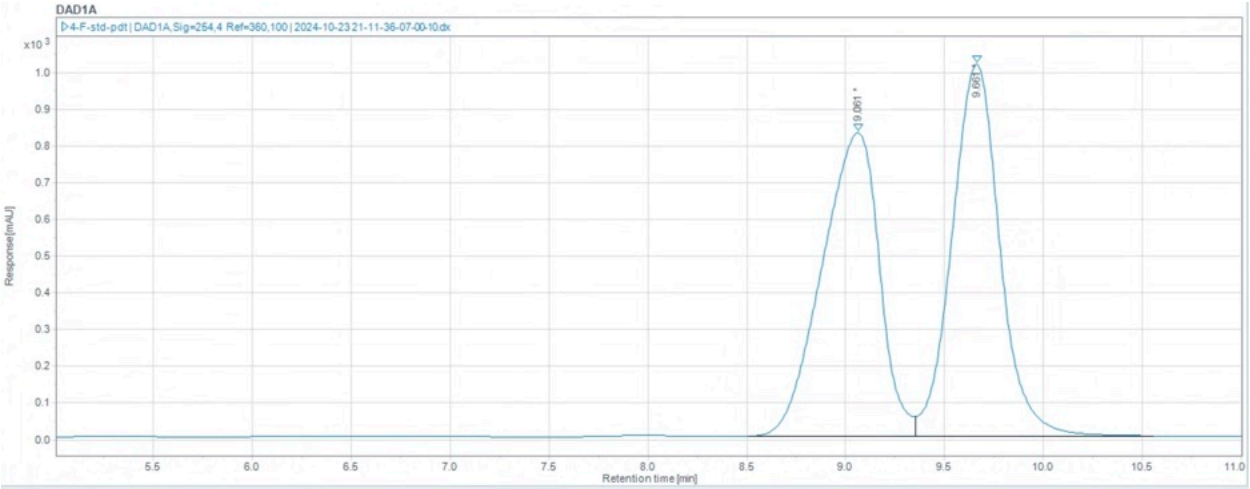

Injection Results

Peaks | Summary

#	Name	Signal description	RT (min)	Area (mAU·s)	Area%	Height (mAU)	Height%	Amount	Concentration	Start time (min)	End time (min)
1		DAD1A,Sig=254,4 Ref=360,100	9.061	16639.459	49.441	826.756	44.93			8.506	9.351
2		DAD1A,Sig=254,4 Ref=360,100	9.661	17015.497	50.559	1013.214	55.07			9.351	10.556

Enzymatic preparation of 3e with *pa*Pgb-AKS-(S)-G3-5332: 89% *ee*

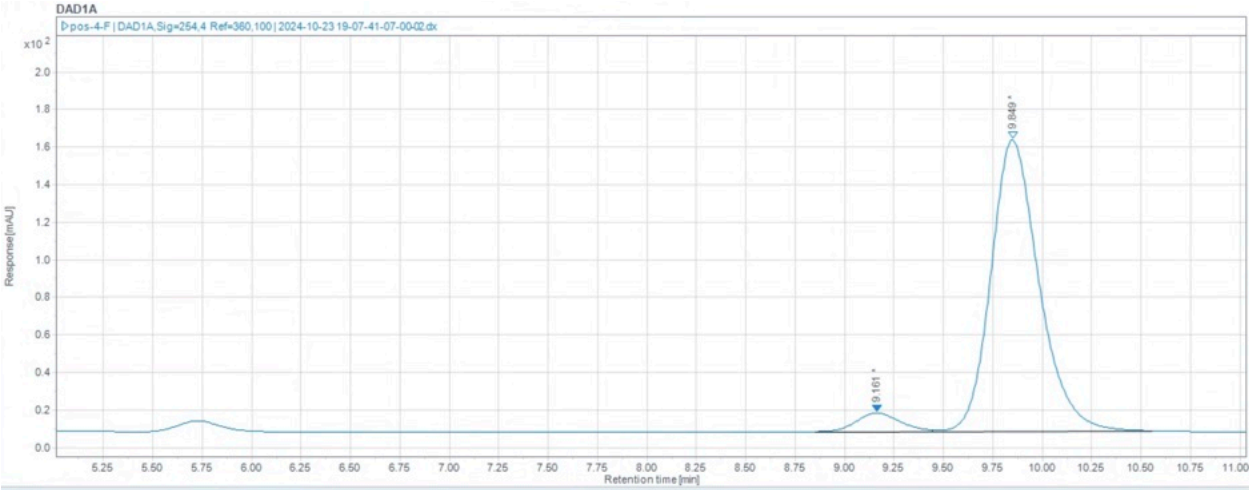

Injection Results

Peaks | Summary

#	Name	Signal description	RT (min)	Area (mAU·s)	Area%	Height (mAU)	Height%	Amount	Concentration	Start time (min)	End time (min)
1		DAD1A,Sig=254,4 Ref=360,100	9.161	150.468	5.424	9.897	6.00			8.852	9.442
2		DAD1A,Sig=254,4 Ref=360,100	9.849	2623.587	94.576	155.125	94.00			9.442	10.556

Enzymatic preparation of 3e with *pa*Pgb-AKS-(R)-G3-5335: –88% *ee*

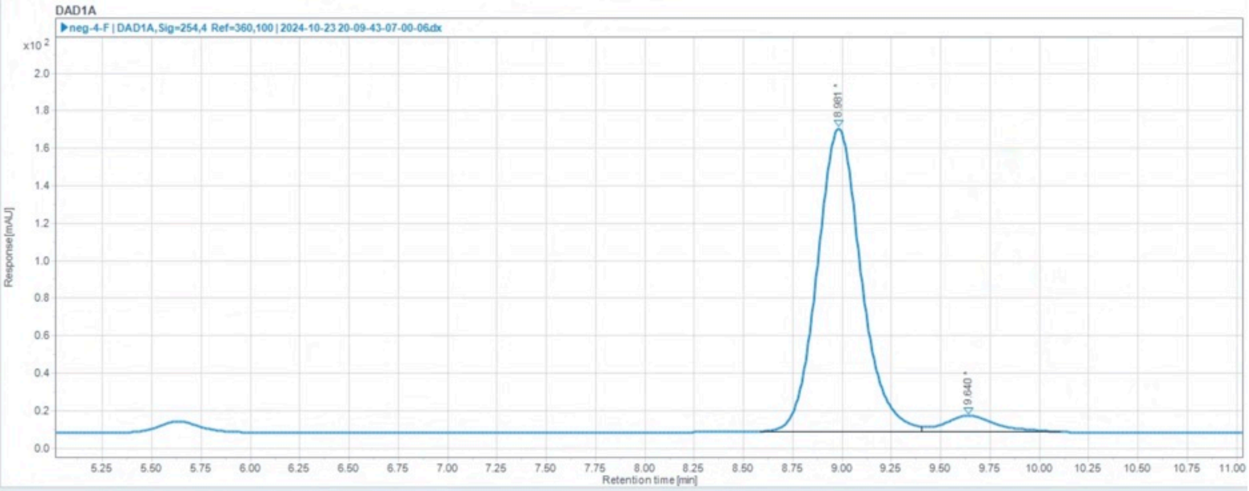

Injection Results

Peaks | Summary

#	Name	Signal description	RT (min)	Area (mAU·s)	Area%	Height (mAU)	Height%	Amount	Concentration	Start time (min)	End time (min)
1		DAD1A,Sig=254,4 Ref=360,100	8.981	2503.620	94.180	161.656	94.92			8.585	9.406
2		DAD1A,Sig=254,4 Ref=360,100	9.640	154.716	5.820	8.660	5.08			9.406	10.105

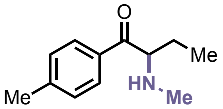

2-(Methylamino)-1-(*p*-tolyl)butan-1-one (3f)
Chiral polar protic HPLC conditions: Angilent Poroshell 120 Chiral-V, 2.7 µm, 2.1 × 150 mm, 95:5 EtOH:water (20 mM ammonium formate, pH 4.0), 0.2 mL/min, 25 ºC, 210 nm

Racemic 3f:

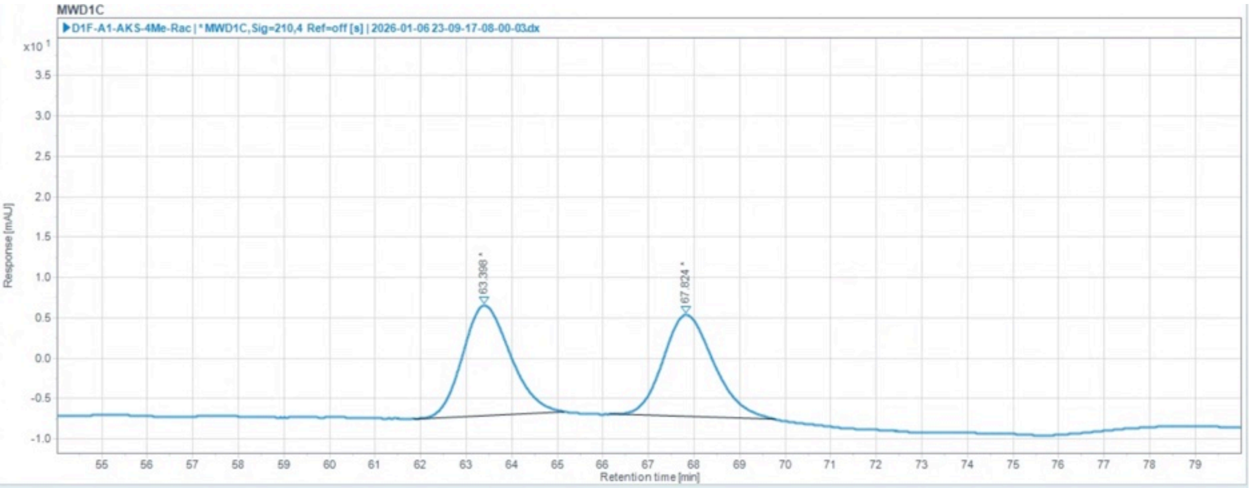

Injection Results

Peaks | Summary

#	Name	Signal description	RT (min)	Area (mAU·s)	Area%	Height (mAU)	Height%	Amou	Concentrati	Start time (	End t
1		* MWD1C,Sig=210,4 Ref=off [s]	63.398	985.924	50.186	13.577	51.88			61.866	5.146
2		* MWD1C,Sig=210,4 Ref=off [s]	67.824	978.623	49.814	12.595	48.12			66.153	9.781

Enzymatic preparation of 3f with *pa*Pgb-AKS-(S)-G3-5332: 95% *ee*

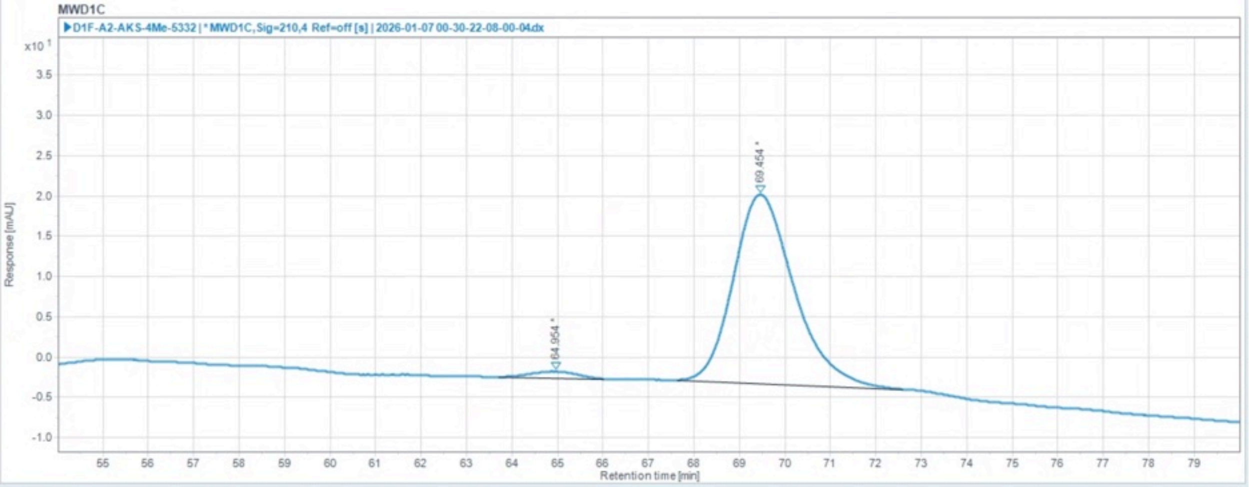

Injection Results

Peaks | Summary

#	Name	Signal description	RT (min)	Area (mAU·s)	Area%	Height (mAU)	Height%	Amou	Concentrati	Start time (	End t
1		* MWD1C,Sig=210,4 Ref=off [s]	64.954	56.348	2.530	0.811	3.35			63.696	6.012
2		* MWD1C,Sig=210,4 Ref=off [s]	69.454	2170.819	97.470	23.404	96.65			67.626	2.593

Enzymatic preparation of 3f with *pa*Pgb-AKS-(R)-G3-5335: –90% *ee*

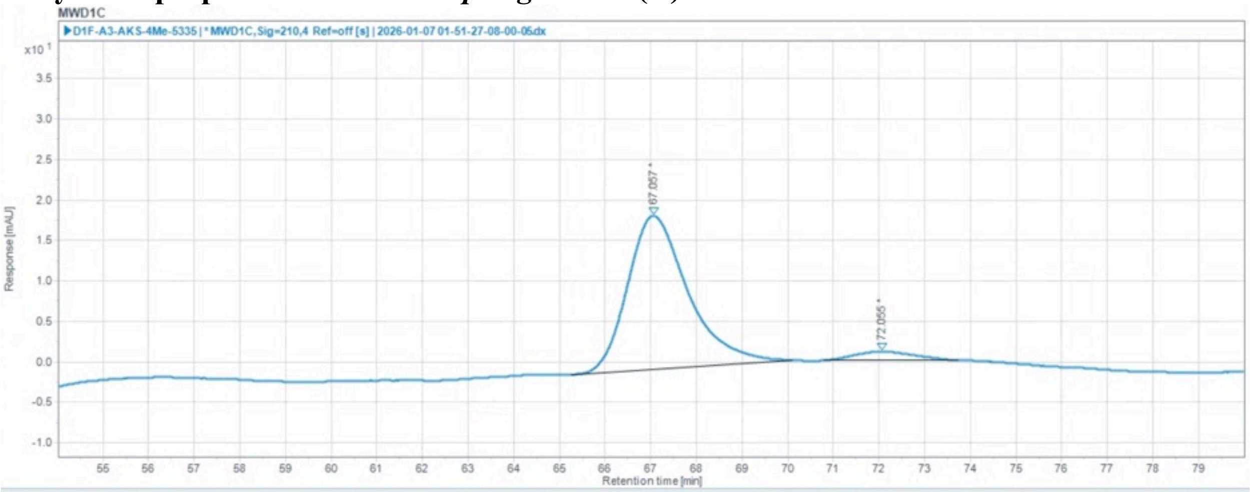

Injection Results

Peaks Summary

#	Name	Signal description	RT (min)	Area (mAU·s)	Area%	Height (mAU)	Height%	Amou	Concentrati	Start time (	End t
1		* MWD1C,Sig=210,4 Ref=off [s]	67.057	1741.051	94.801	18.952	94.63			65.264	0.096
2		* MWD1C,Sig=210,4 Ref=off [s]	72.055	95.477	5.199	1.076	5.37			70.779	3.721

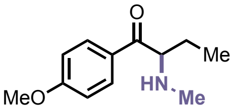

1-(4-Methoxyphenyl)-2-(methylamino)butan-1-one (3g)
Chiral polar protic HPLC conditions: Angilent Poroshell 120 Chiral-V, 2.7 µm, 2.1 × 150 mm, 95:5 EtOH:water (20 mM ammonium formate, pH 4.0), 0.5 mL/min, 25 ºC, 254 nm

Racemic 3g:

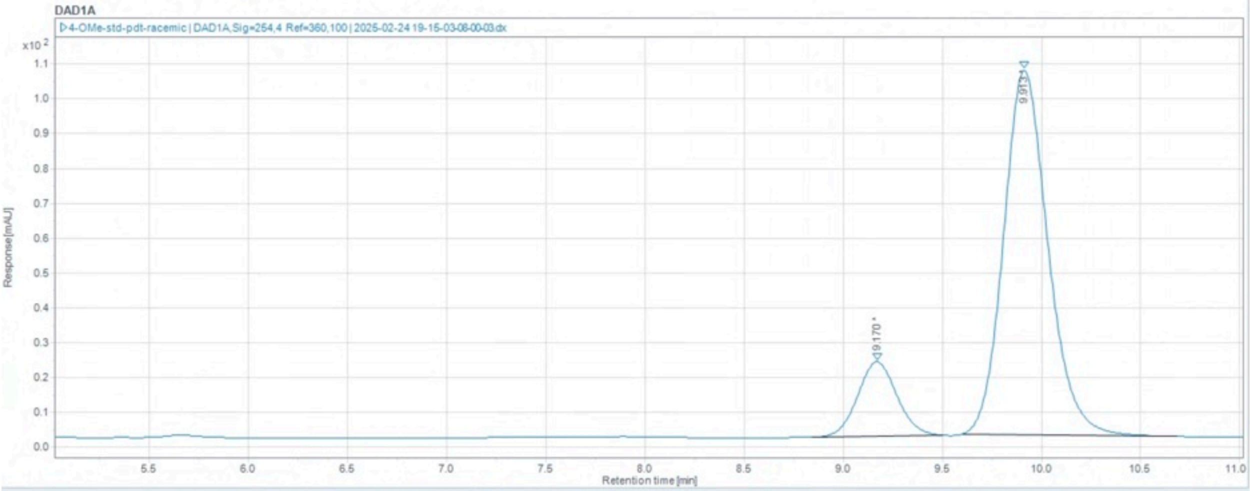

Injection Results

Peaks | Summary

#	Name	Signal description	RT (min)	Area (mAU·s)	Area%	Height (mAU)	Height%	Amount	Concentration	Start time (min)	End time (min)
1		DAD1A,Sig=254,4 Ref=360,100	9.170	295.370	15.400	21.433	17.01			8.842	9.501
2		DAD1A,Sig=254,4 Ref=360,100	9.913	1622.616	84.600	104.540	82.99			9.601	10.682

Enzymatic preparation of 3g with *pa*Pgb-AKS-(S)-G3-5332: 78% *ee*

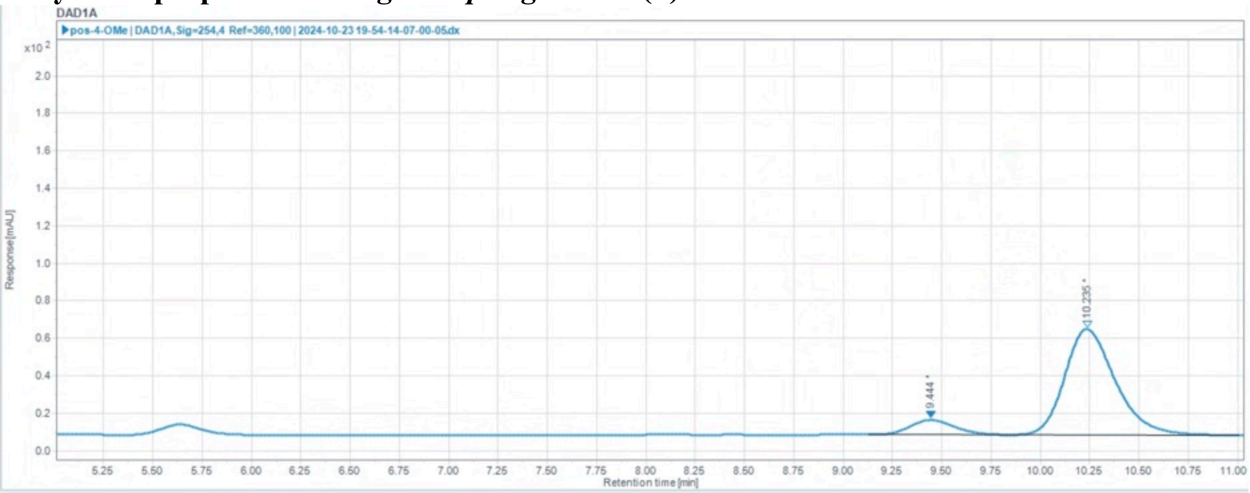

Injection Results

Peaks | Summary

#	Name	Signal description	RT (min)	Area (mAU·s)	Area%	Height (mAU)	Height%	Amount	Concentration	Start time (min)	End time (min)
1		DAD1A,Sig=254,4 Ref=360,100	9.444	123.398	10.870	7.944	12.31			9.128	9.801
2		DAD1A,Sig=254,4 Ref=360,100	10.235	1011.800	89.130	56.611	87.69			9.801	11.009

Enzymatic preparation of 3g with *pa*Pgb-AKS-(R)-G3-5335: –92% *ee*

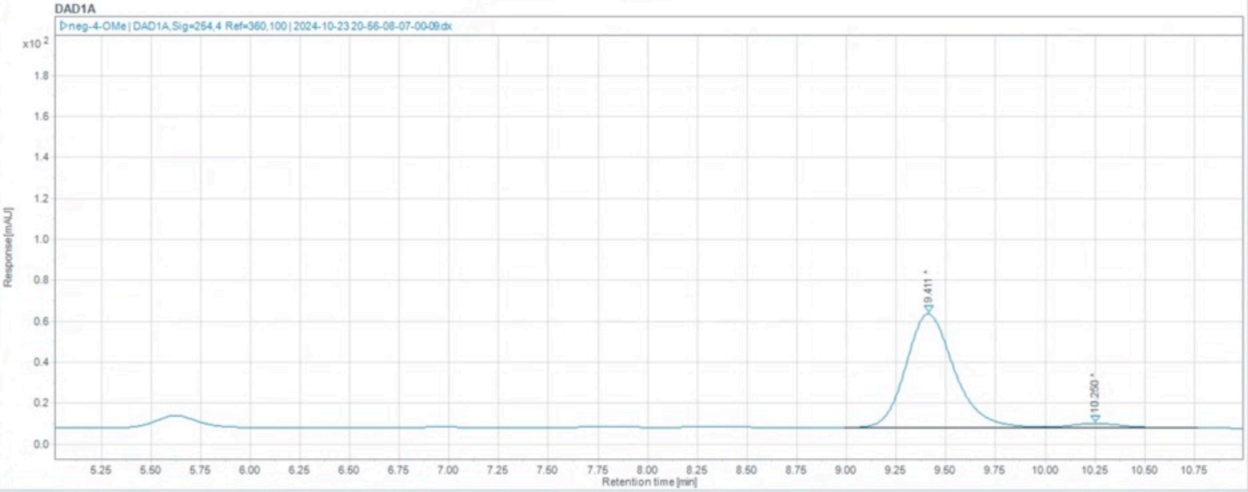

Injection Results

Peaks Summary

#	Name	Signal description	RT (min)	Area (mAU·s)	Area%	Height (mAU)	Height%	Amount	Concentration	Start time (min)	End time (min)
1		DAD1A,Sig=254,4 Ref=360,100	9.411	938.338	95.916	55.596	96.24			8.990	9.997
2		DAD1A,Sig=254,4 Ref=360,100	10.250	39.958	4.084	2.174	3.76			9.997	10.764

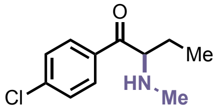

1-(4-Chlorophenyl)-2-(methylamino)butan-1-one (3h)
Chiral polar protic HPLC conditions: Angilent Poroshell 120 Chiral-V, 2.7 µm, 2.1 × 150 mm, 95:5 EtOH:water (20 mM ammonium formate, pH 4.0), 0.5 mL/min, 25 ºC, 254 nm

Racemic 3h:

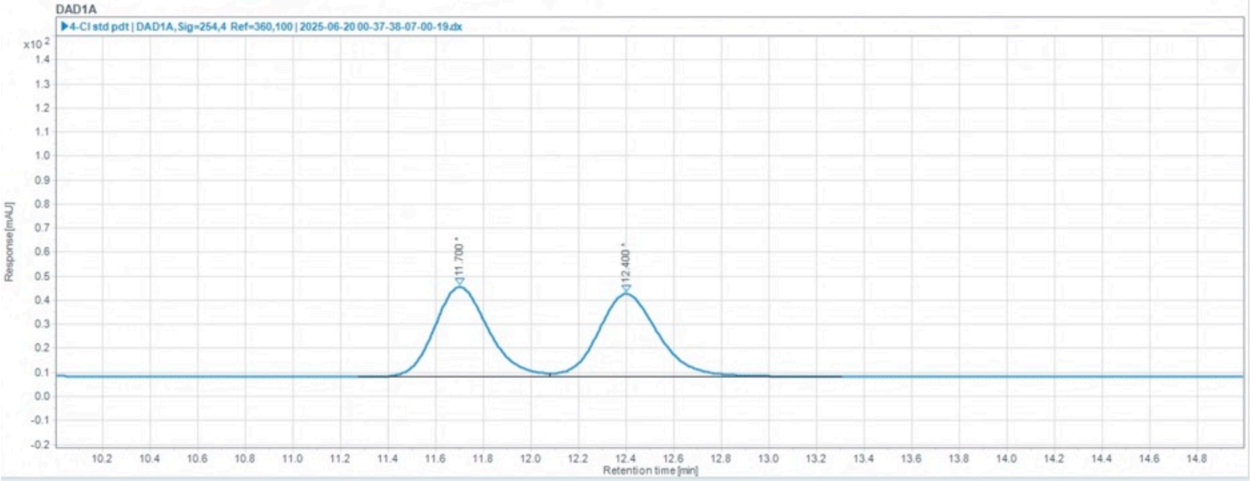

Injection Results

Peaks | Summary

#	Name	Signal description	RT (min)	Area (mAU·s)	Area%	Height (mAU)	Height%	Amount	Concentration	Start time (min)	End time (min)
1		DAD1A,Sig=254,4 Ref=360,100	11.700	592.917	49.374	37.429	52.01			11.275	12.079
2		DAD1A,Sig=254,4 Ref=360,100	12.400	607.963	50.626	34.543	47.99			12.079	13.305

Enzymatic preparation of 3h with *pa*Pgb-AKS-(S)-G3-5332: 83% *ee*

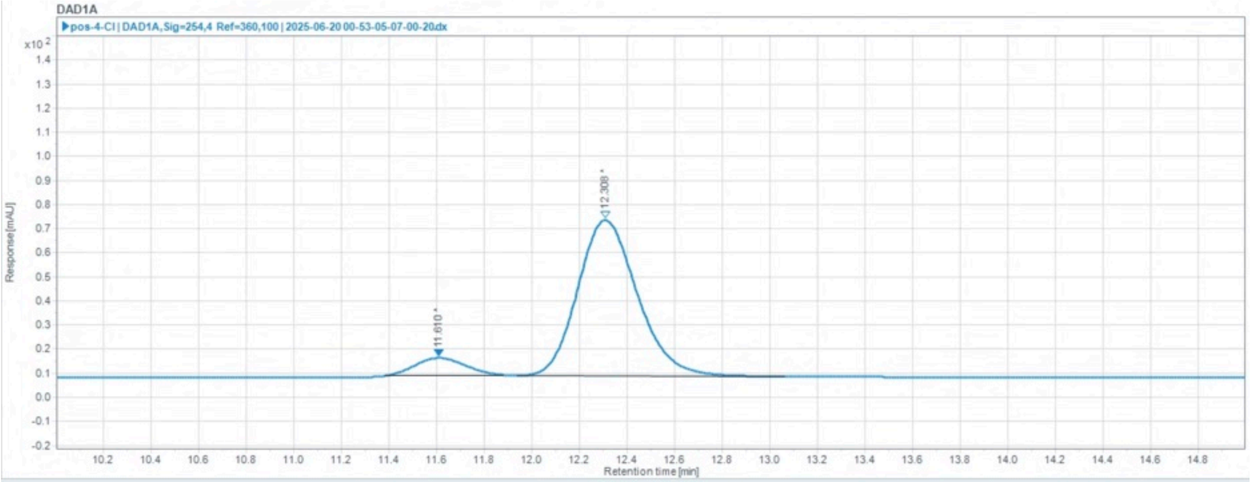

Injection Results

Peaks | Summary

#	Name	Signal description	RT (min)	Area (mAU·s)	Area%	Height (mAU)	Height%	Amount	Concentration	Start time (min)	End time (min)
1		DAD1A,Sig=254,4 Ref=360,100	11.610	106.142	8.563	7.254	10.07			11.385	11.880
2		DAD1A,Sig=254,4 Ref=360,100	12.308	1133.339	91.437	64.799	89.93			11.937	13.064

Enzymatic preparation of 3h with *pa*Pgb-AKS-(R)-G3-5335: –87% *ee*

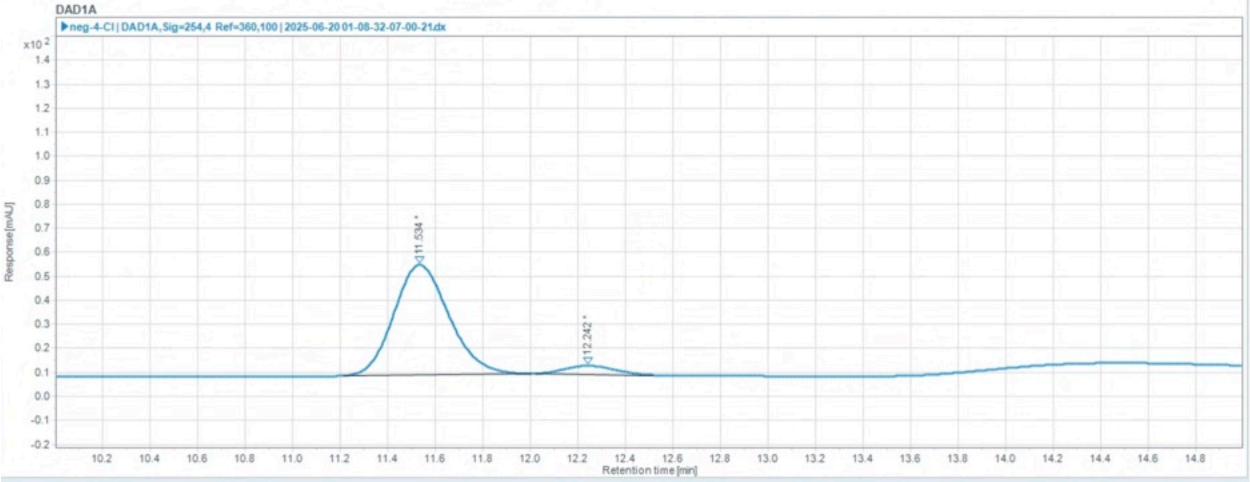

Injection Results

Peaks | Summary

#	Name	Signal description	RT (min)	Area (mAU·s)	Area%	Height (mAU)	Height%	Amount	Concentration	Start time (min)	End time (min)
1		DAD1A,Sig=254,4 Ref=360,100	11.534	740.111	93.448	45.803	92.89			11.213	12.004
2		DAD1A,Sig=254,4 Ref=360,100	12.242	51.890	6.552	3.507	7.11			12.023	12.519

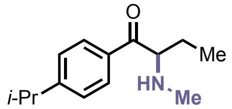

1-(4-Isopropylphenyl)-2-(methylamino)butan-1-one (3i)
Chiral polar protic HPLC conditions: 2 × Angilent Poroshell 120 Chiral-V, 2.7 µm, 2.1 × 150 mm, 95:5 EtOH:water (20 mM ammonium formate, pH 4.0), 0.5 mL/min, 25 ºC, 254 nm

Racemic 3i:

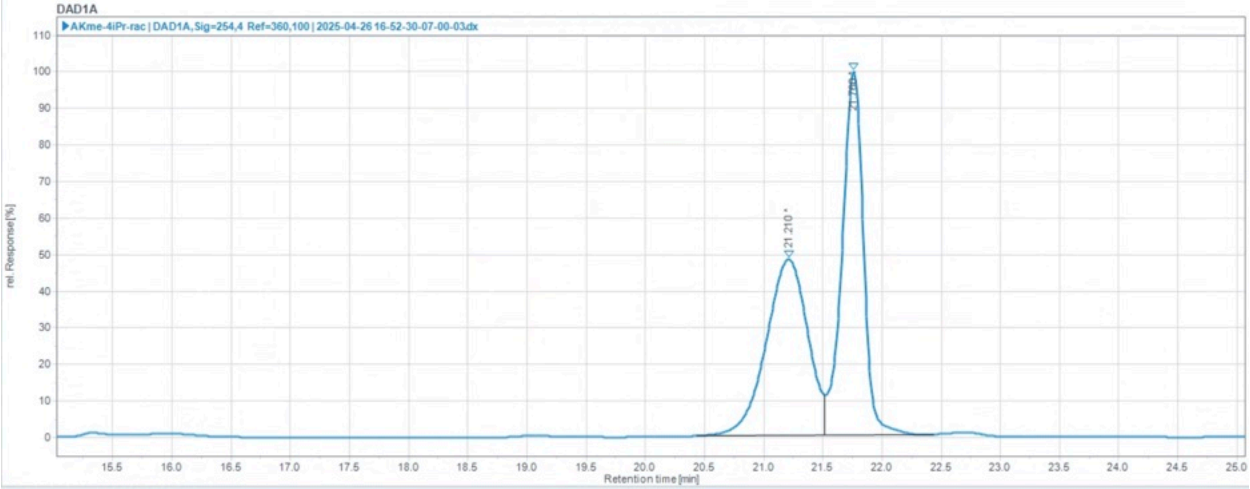

Injection Results

Peaks | Summary

#	Name	Signal description	RT (min)	Area (mAU·s)	Area%	Height (mAU)	Height%	Amount	Concentration	Start time (min)	End time (min)
1		DAD1A,Sig=254,4 Ref=360,100	21.210	3651.390	48.462	149.607	32.63			20.436	21.512
2		DAD1A,Sig=254,4 Ref=360,100	21.760	3883.114	51.538	308.843	67.37			21.512	22.438

Enzymatic preparation of 3i with *pa*Pgb-AKS-(S)-G3-5332: 95% *ee*

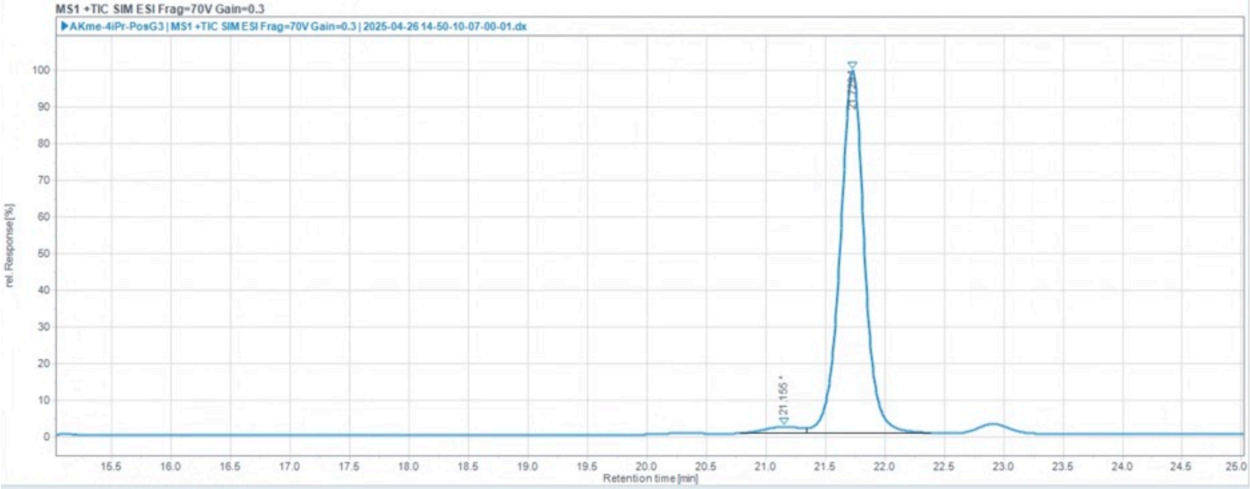

Injection Results

Peaks | Summary

#	Name	Signal description	RT (min)	Area (Counts·s)	Area%	Height (Counts	Height%	Amount	Concentration	Start time (min)	End time (min)
1		MS1 +TIC SIM ESI Frag=70V Gain=0.3	21.155	288408.858	2.552	14035.162	1.78			20.788	21.342
2		MS1 +TIC SIM ESI Frag=70V Gain=0.3	21.729	11013712.959	97.448	774335.745	98.22			21.342	22.386

Enzymatic preparation of 3i with *pa*Pgb-AKS-(R)-G3-5335: –89% *ee*

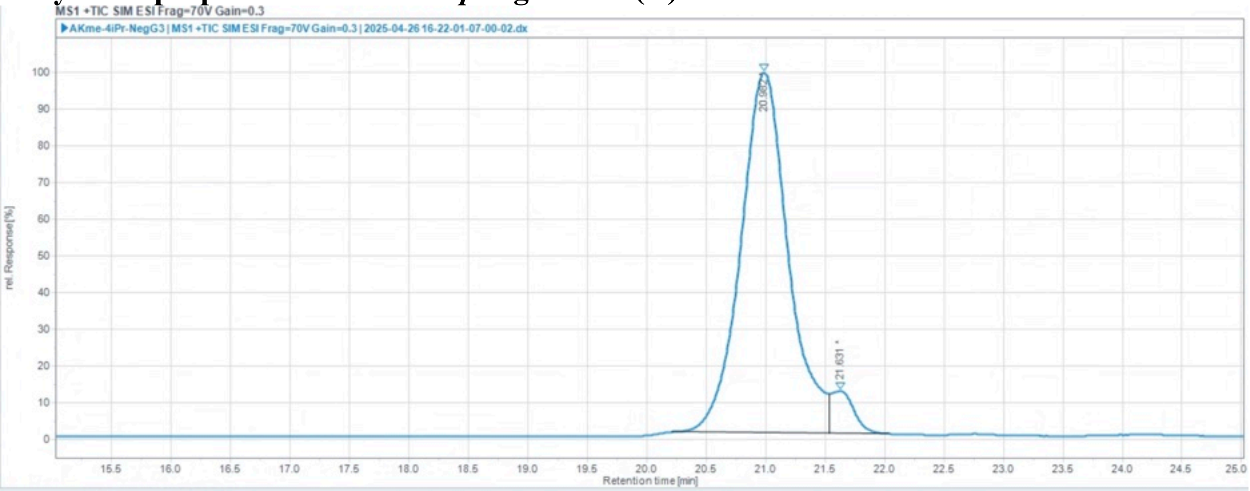

Injection Results

Peaks | Summary

#	Name	Signal description	RT (min)	Area (Counts·s)	Area%	Height (Counts)	Height%	Amount	Concentration	Start time (min)	End time (min)
1		MS1 +TIC SIM ESI Frag=70V Gain=0.3	20.982	11343836.368	94.511	408751.195	89.43			20.214	21.531
2		MS1 +TIC SIM ESI Frag=70V Gain=0.3	21.631	658842.405	5.489	48328.236	10.57			21.531	22.040

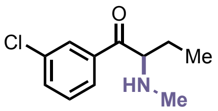

1-(3-Chlorophenyl)-2-(methylamino)butan-1-one (3j)
Chiral polar protic HPLC conditions: Angilent Poroshell 120 Chiral-V, 2.7 µm, 2.1 × 150 mm, 95:5 EtOH:water (20 mM ammonium formate, pH 4.0), 0.4 mL/min, 25 ºC, 254 nm

Racemic 3j:

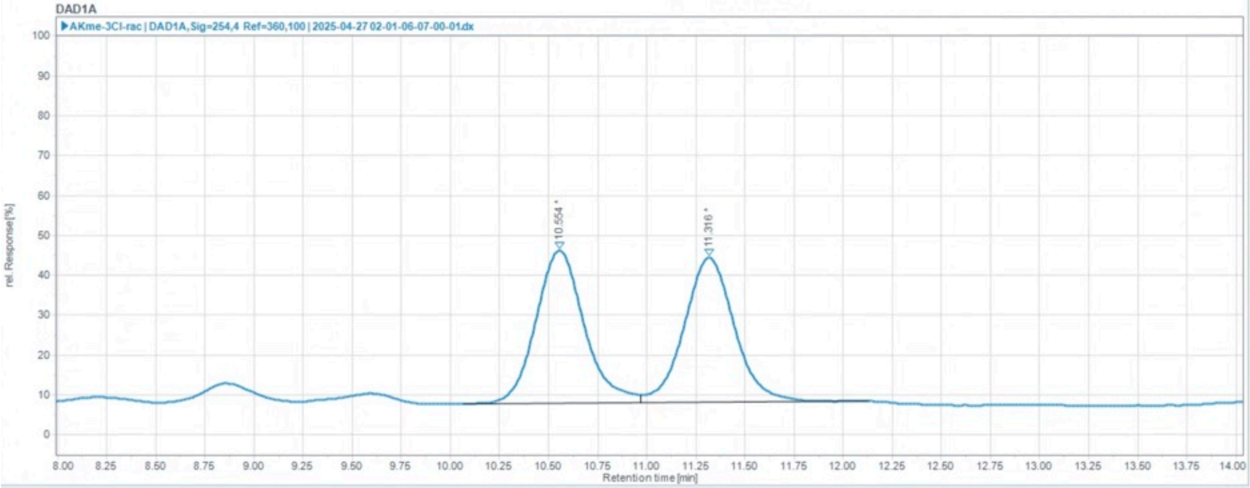

Injection Results

Peaks | Summary

#	Name	Signal description	RT (min)	Area (mAU·s)	Area%	Height (mAU)	Height%	Amount	Concentration	Start time (min)	End time (min)
1		DAD1A,Sig=254,4 Ref=360,100	10.554	115.133	50.506	6.714	51.33			10.067	10.968
2		DAD1A,Sig=254,4 Ref=360,100	11.316	112.824	49.494	6.366	48.67			10.968	12.132

Enzymatic preparation of 3j with *pa*Pgb-AKS-(S)-G3-5332: 74% *ee*

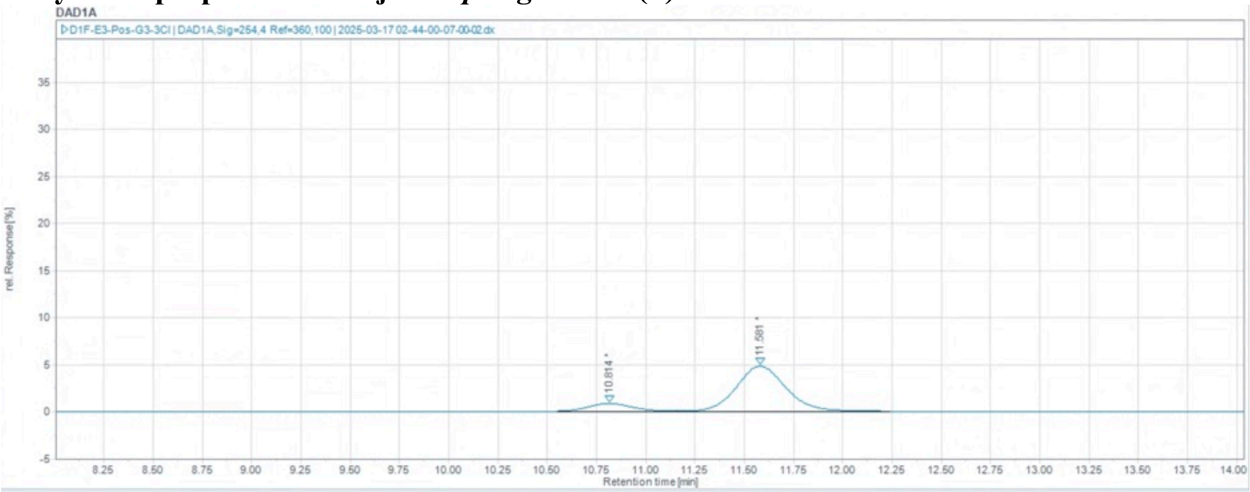

Injection Results

Peaks | Summary

#	Name	Signal description	RT (min)	Area (mAU·s)	Area%	Height (mAU)	Height%	Amount	Concentration	Start time (min)	End time (min)
1		DAD1A,Sig=254,4 Ref=360,100	10.814	82.602	13.076	5.323	14.54			10.550	11.159
2		DAD1A,Sig=254,4 Ref=360,100	11.581	549.107	86.924	31.280	85.46			11.159	12.241

Enzymatic preparation of 3j with *pa*Pgb-AKS-(R)-G3-5335: –69% *ee*

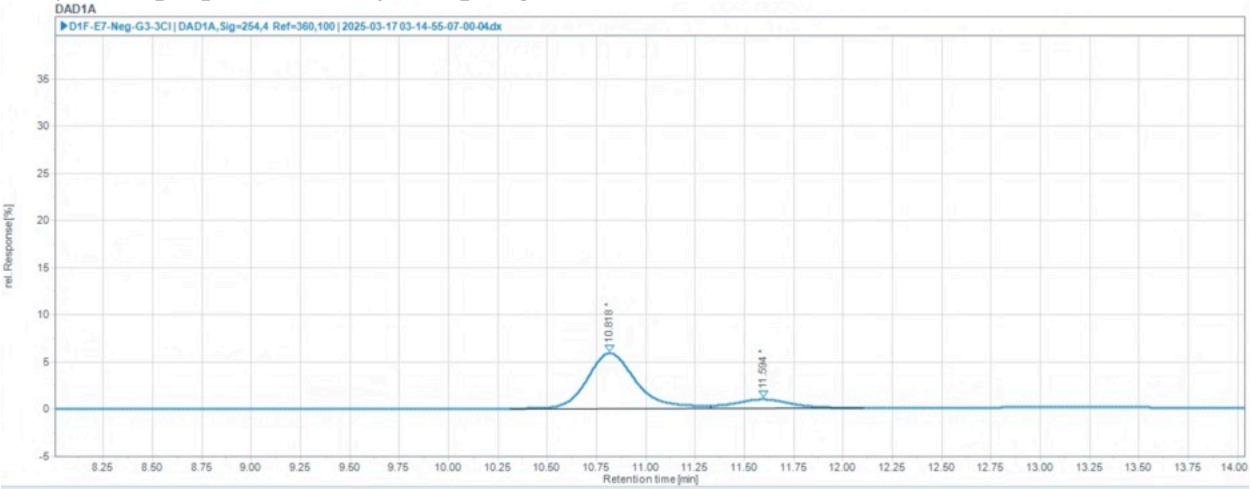

Injection Results

Peaks | Summary

#	Name	Signal description	RT (min)	Area (mAU·s)	Area%	Height (mAU)	Height%	Amount	Concentration	Start time (min)	End time (min)
1		DAD1A,Sig=254,4 Ref=360,100	10.818	610.578	84.638	35.506	86.38			10.314	11.332
2		DAD1A,Sig=254,4 Ref=360,100	11.594	110.817	15.362	5.599	13.62			11.332	12.105

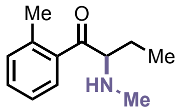

2-(Methylamino)-1-(*o*-tolyl)butan-1-one (3k)
Chiral polar protic HPLC conditions: Angilent Poroshell 120 Chiral-V, 2.7 µm, 2.1 × 150 mm, 95:5 EtOH:water (20 mM ammonium formate, pH 4.0), 0.5 mL/min, 25 ºC, ion count (single ion channel)

Racemic 3k:

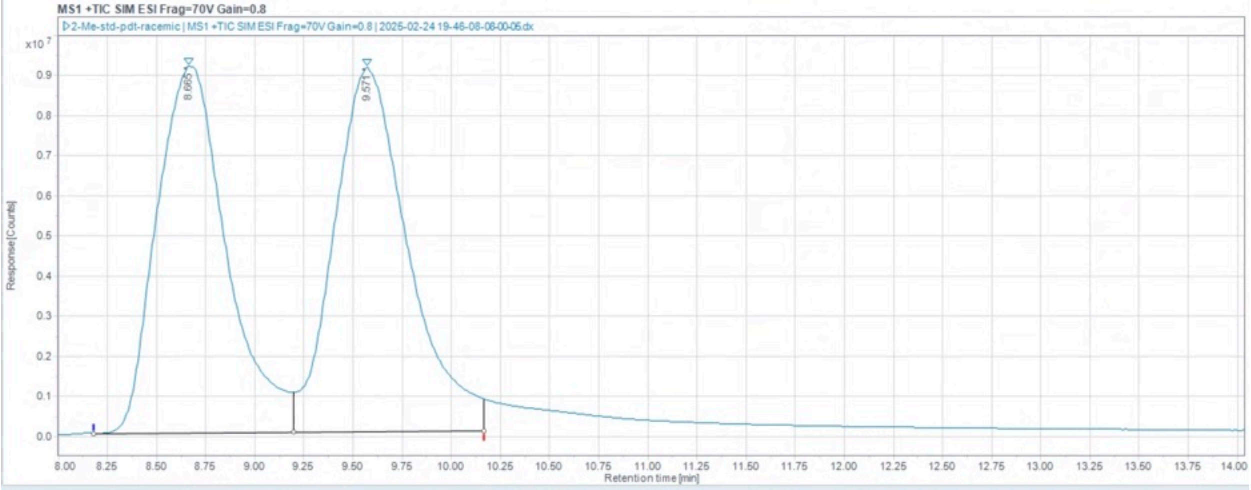

Injection Results

Peaks | Summary

#	Name	Signal description	RT (min)	Area (Counts·s)	Area%	Height (Counts)	Height%	Amount	Concentration	Start time (min)	End time (min)
1		MS1 +TIC SIM ESI Frag=70V Gain=0.8	8.665	225378221.258	48.646	9137725.366	50.17			8.181	9.197
2		MS1 +TIC SIM ESI Frag=70V Gain=0.8	9.571	237921041.828	51.354	9076664.463	49.83			9.197	10.165

Enzymatic preparation of 3k with *pa*Pgb-AKS-(S)-G3-5332: 35% *ee*

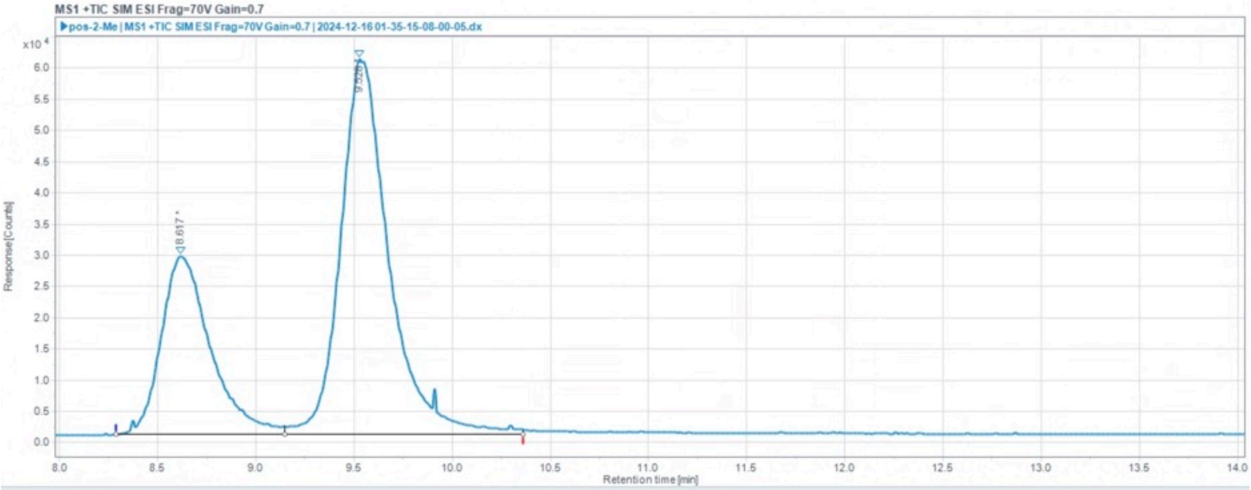

Injection Results

Peaks | Summary

#	Name	Signal description	RT (min)	Area (Counts·s)	Area%	Height (Counts)	Height%	Amount	Concentration	Start time (min)	End time (min)
1		MS1 +TIC SIM ESI Frag=70V Gain=0.7	8.617	500853.372	32.633	28621.780	32.21			8.286	9.147
2		MS1 +TIC SIM ESI Frag=70V Gain=0.7	9.528	1033931.439	67.367	60228.661	67.79			9.147	10.362

Enzymatic preparation of 3k with *pa*Pgb-AKS-(R)-G3-5335: –42% *ee*

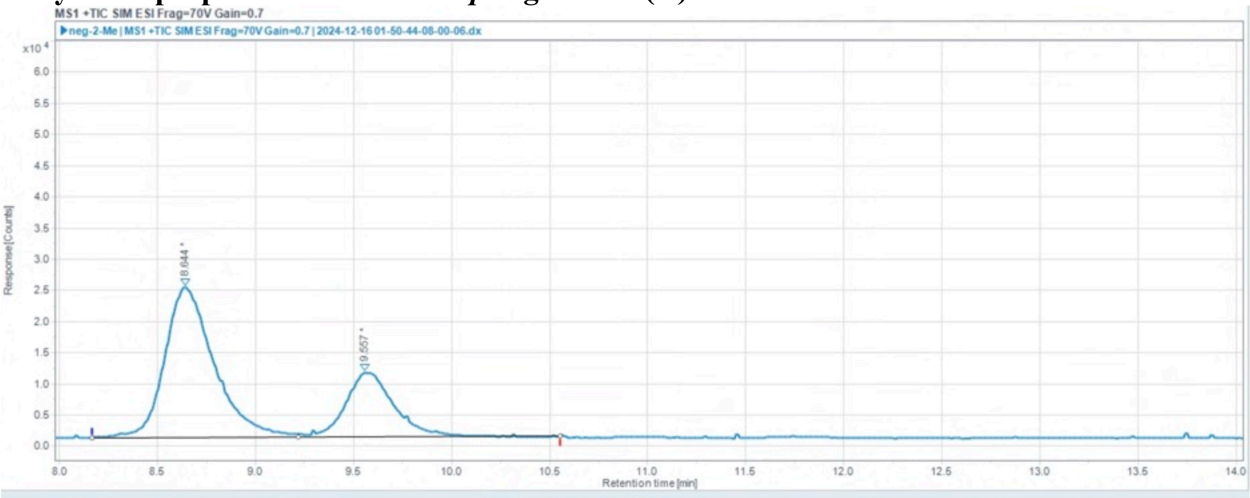

Injection Results

Peaks Summary

#	Name	Signal description	RT (min)	Area (Counts·s)	Area%	Height (Counts)	Height%	Amount	Concentration	Start time (min)	End time (min)
1		MS1 +TIC SIM ESI Frag=70V Gain=0.7	8.644	421176.780	70.914	24119.171	70.11			8.164	9.215
2		MS1 +TIC SIM ESI Frag=70V Gain=0.7	9.557	172751.682	29.086	10281.697	29.89			9.215	10.555

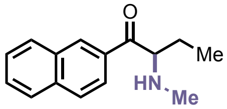

2-(Methylamino)-1-(naphthalen-2-yl)butan-1-one (3l)
Chiral polar protic HPLC conditions: Angilent Poroshell 120 Chiral-V, 2.7 µm, 2.1 × 150 mm, 95:5 EtOH:water (20 mM ammonium formate, pH 4.0), 0.2 mL/min, 25 ºC, 254 nm

Racemic 3l:

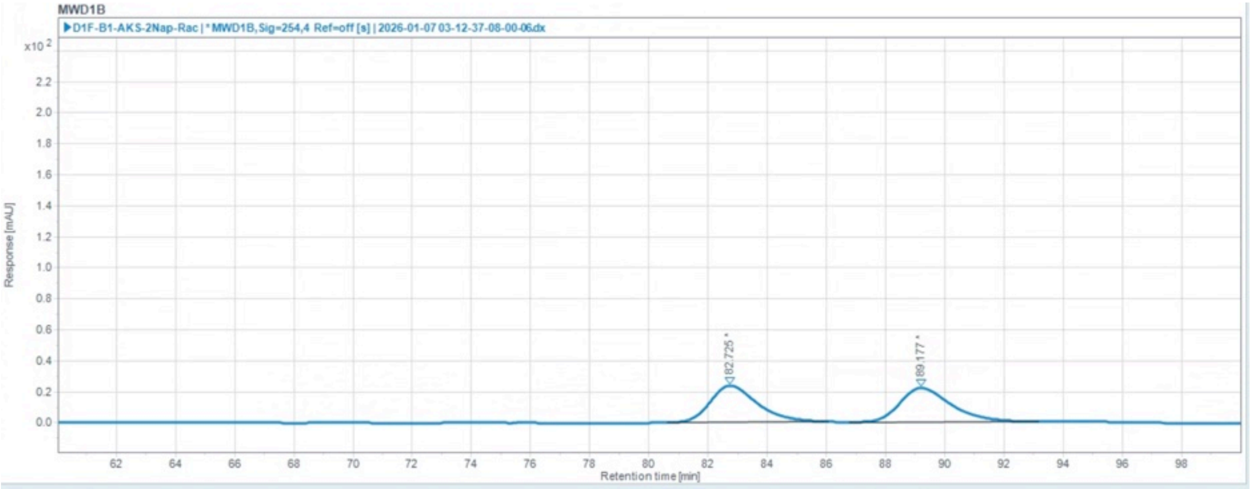

Injection Results

Peaks | Summary

#	Name	Signal description	RT (min)	Area (mAU·s)	Area%	Height (mAU)	Height%	Amou	Concentrati	Start time (	End t
1		* MWD1B,Sig=254,4 Ref=off [s]	82.725	2635.033	49.956	23.694	51.98			80.610	6.083
2		* MWD1B,Sig=254,4 Ref=off [s]	89.177	2639.702	50.044	21.888	48.02			86.770	3.152

Enzymatic preparation of 3l with *pa*Pgb-AKS-(S)-G3-5332: 82% *ee*

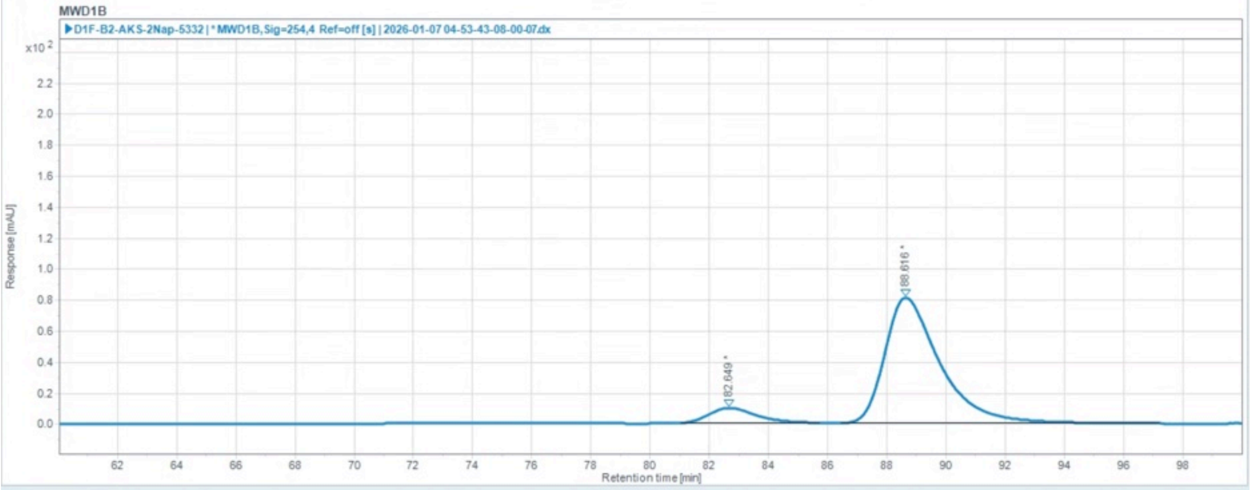

Injection Results

Peaks | Summary

#	Name	Signal description	RT (min)	Area (mAU·s)	Area%	Height (mAU)	Height%	Amou	Concentrati	Start time (	End t
1		* MWD1B,Sig=254,4 Ref=off [s]	82.649	1055.189	9.148	9.598	10.60			81.062	5.719
2		* MWD1B,Sig=254,4 Ref=off [s]	88.616	10479.786	90.852	80.931	89.40			86.466	7.186

Enzymatic preparation of 3l with *pa*Pgb-AKS-(R)-G3-5335: –82% *ee*

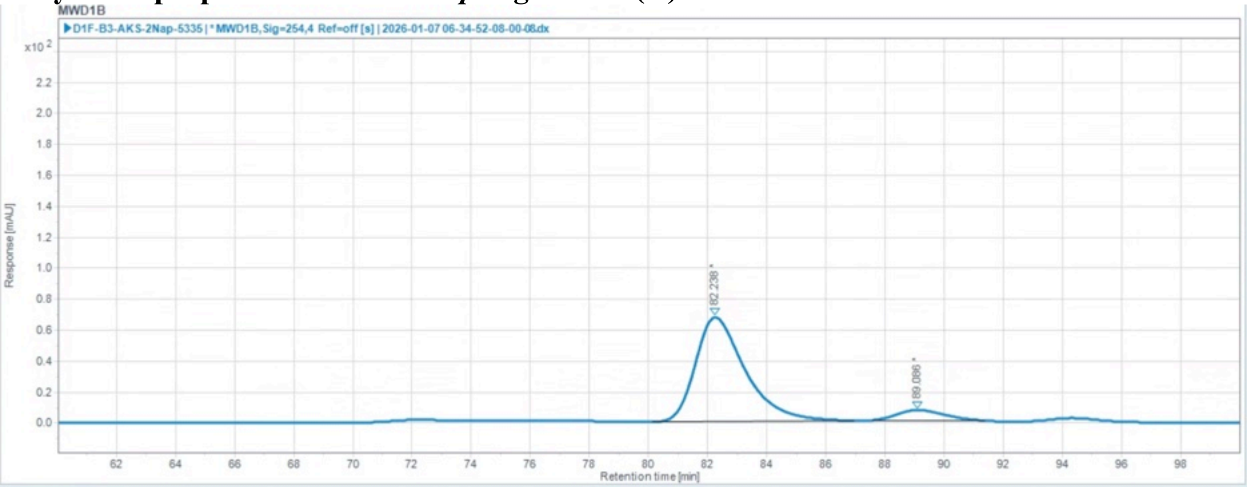

Injection Results

Peaks | Summary

#	Name	Signal description	RT (min)	Area (mAU·s)	Area%	Height (mAU)	Height%	Amou	Concentrati	Start time (	End t
1		* MWD1B,Sig=254,4 Ref=off [s]	82.238	7871.387	91.493	67.294	91.02			80.144	6.947
2		* MWD1B,Sig=254,4 Ref=off [s]	89.086	731.907	8.507	6.643	8.98			87.568	1.379

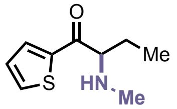

2-(Methylamino)-1-(thiophen-2-yl)butan-1-one (3m)
Chiral polar protic HPLC conditions: Angilent Poroshell 120 Chiral-V, 2.7 µm, 2.1 × 150 mm, 95:5 EtOH:water (20 mM ammonium formate, pH 4.0), 0.5 mL/min, 25 ºC, 254 nm

Racemic 3m:

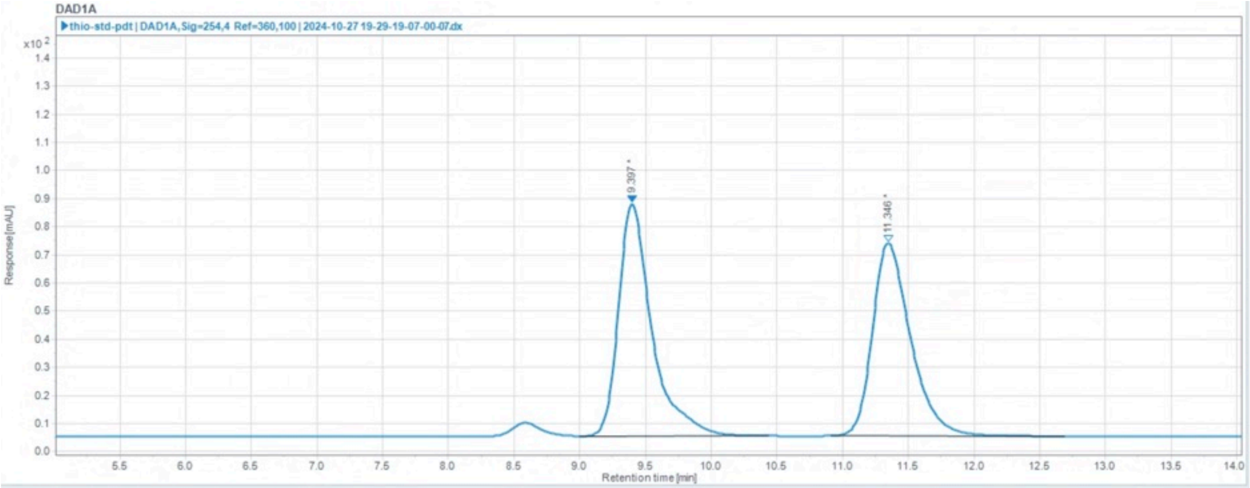

Injection Results

Peaks | Summary

#	Name	Signal description	RT (min)	Area (mAU·s)	Area%	Height (mAU)	Height%	Amount	Concentration	Start time (min)	End time (min)
1		DAD1A,Sig=254,4 Ref=360,100	9.397	1462.100	51.341	82.602	54.64			8.999	10.438
2		DAD1A,Sig=254,4 Ref=360,100	11.346	1385.740	48.659	68.572	45.36			10.914	12.691

Enzymatic preparation of 3m with *pa*Pgb-AKS-(S)-G3-5332: 94% *ee*

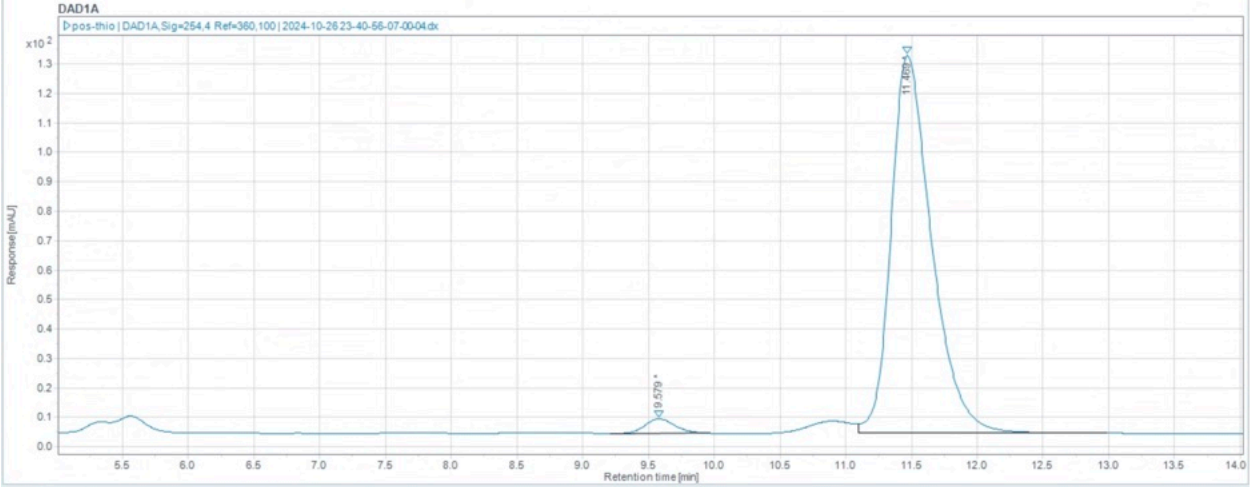

Injection Results

Peaks | Summary

#	Name	Signal description	RT (min)	Area (mAU·s)	Area%	Height (mAU)	Height%	Amount	Concentration	Start time (min)	End time (min)
1		DAD1A,Sig=254,4 Ref=360,100	9.579	78.601	2.782	4.950	3.72			9.212	9.967
2		DAD1A,Sig=254,4 Ref=360,100	11.469	2746.963	97.218	128.248	96.28			11.097	12.984

Enzymatic preparation of 3m with *pa*Pgb-AKS-(R)-G3-5335: –90% *ee*

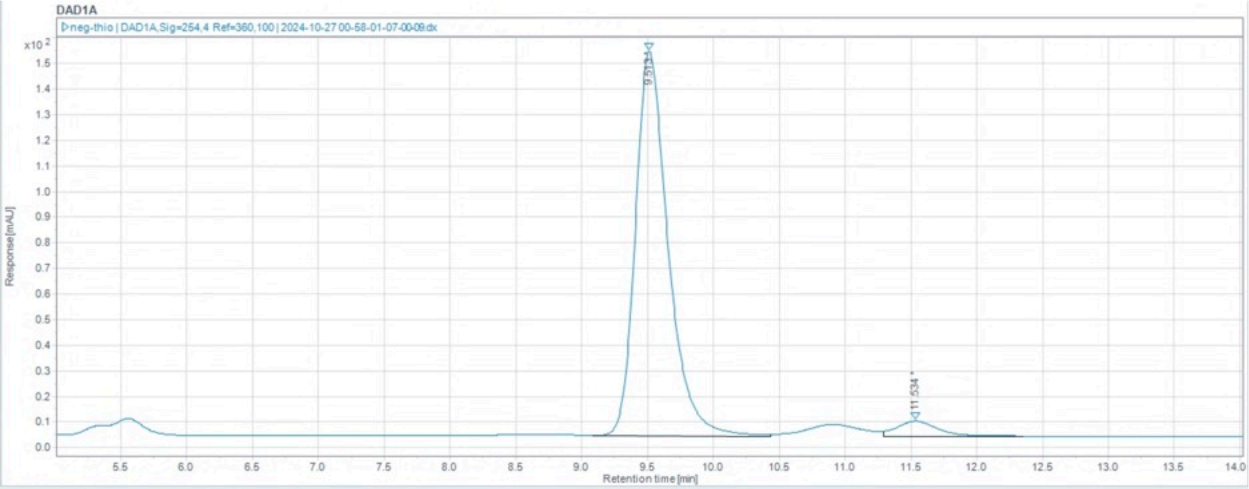

Injection Results

Peaks | Summary

#	Name	Signal description	RT (min)	Area (mAU·s)	Area%	Height (mAU)	Height%	Amount	Concentration	Start time (min)	End time (min)
1		DAD1A,Sig=254,4 Ref=360,100	9.513	2596.954	95.201	150.044	96.12			9.080	10.437
2		DAD1A,Sig=254,4 Ref=360,100	11.534	130.905	4.799	6.050	3.88			11.296	12.350

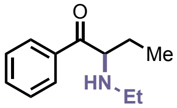

2-(Ethylamino)-1-phenylbutan-1-one (3q)
Chiral polar protic HPLC conditions: Angilent Poroshell 120 Chiral-V, 2.7 µm, 2.1 × 150 mm, 95:5 EtOH:water (20 mM ammonium formate, pH 4.0), 0.5 mL/min, 25 ºC, 254 nm

Racemic 3q:

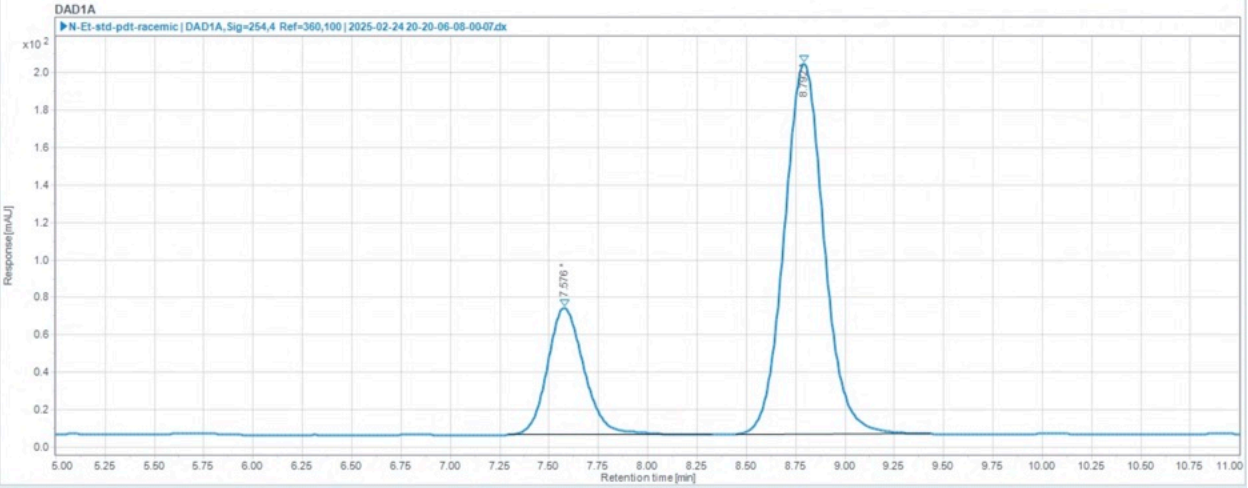

Injection Results

Peaks | Summary

#	Name	Signal description	RT (min)	Area (mAU·s)	Area%	Height (mAU)	Height%	Amount	Concentration	Start time (min)	End time (min)
1		DAD1A,Sig=254,4 Ref=360,100	7.576	875.586	24.069	67.857	25.55			7.291	8.327
2		DAD1A,Sig=254,4 Ref=360,100	8.792	2762.246	75.931	197.720	74.45			8.448	9.431

Enzymatic preparation of 3q with *pa*Pgb-AKS-(S)-G3-5332: 92% *ee*

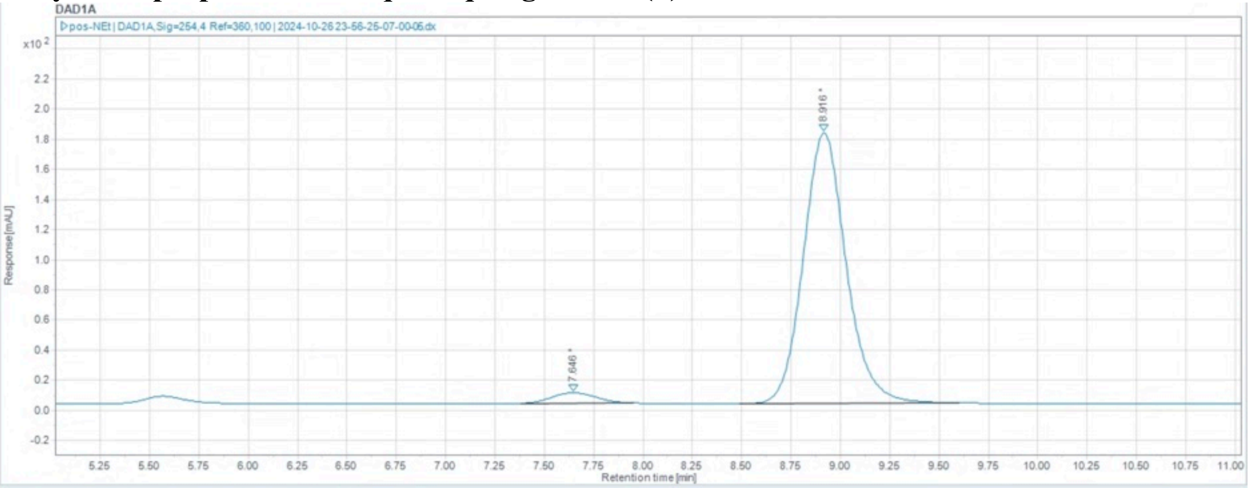

Injection Results

Peaks | Summary

#	Name	Signal description	RT (min)	Area (mAU·s)	Area%	Height (mAU)	Height%	Amount	Concentration	Start time (min)	End time (min)
1		DAD1A,Sig=254,4 Ref=360,100	7.646	107.646	3.807	6.923	3.71			7.379	7.952
2		DAD1A,Sig=254,4 Ref=360,100	8.916	2719.783	96.193	179.624	96.29			8.491	9.603

Enzymatic preparation of 3q with *pa*Pgb-AKS-(R)-G3-5335: –59% *ee*

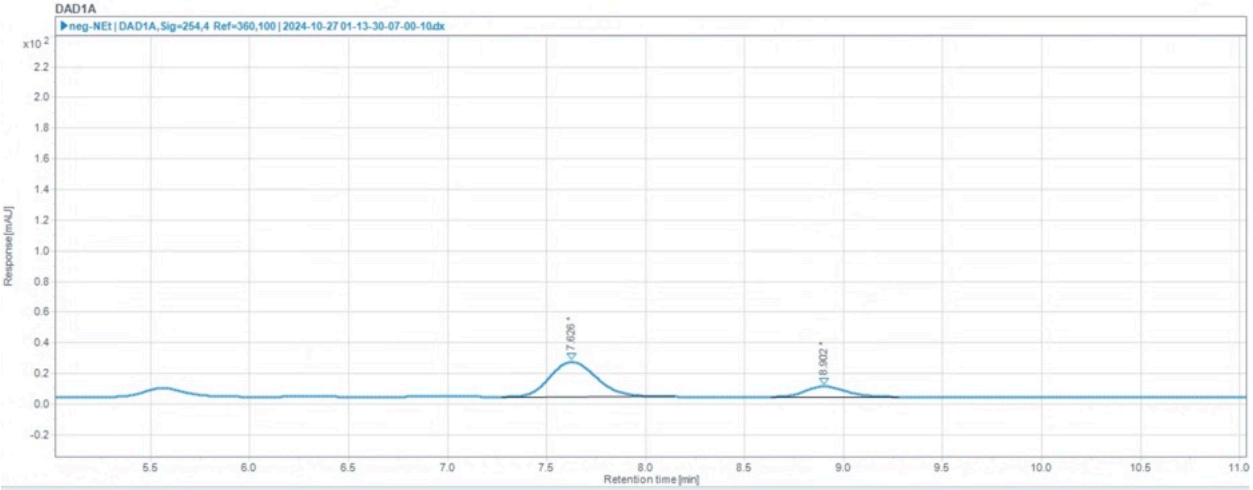

Injection Results

Peaks Summary

#	Name	Signal description	RT (min)	Area (mAU·s)	Area%	Height (mAU)	Height%	Amount	Concentration	Start time (min)	End time (min)
1		DAD1A,Sig=254,4 Ref=360,100	7.626	376.379	79.445	22.708	76.87			7.274	8.147
2		DAD1A,Sig=254,4 Ref=360,100	8.902	97.380	20.555	6.833	23.13			8.634	9.279

IX. Sequence Information

Mutations present in *pa*Pgb variants described in this study:

***pa*Pgb variant**	**Mutations relative to wild-type *pa*Pgb**
*pa*Pgb-AKS-5329	Y57D, W59L, V60Q
***pa*Pgb-AKS-(*S*)-5330**	**D51N**, Y57D, W59L, **V60A**, **V85G**, **I133V**
***pa*Pgb-AKS-(*S*)-5331**	D51N, **L55P**, Y57D, W59L, V60A, V85G, **I112Q**, I133V, **I152N**, **H158Y**
***pa*Pgb-AKS-(*S*)-5332**	D51N, L55P, Y57D, W59L, V60A, V85G, **M109T**, I112Q, I133V, I152N, **G157R**, H158Y
***pa*Pgb-AKS-(*R*)-5333**	**Y57E**, W59L, V60Q, **R86S**, **P151Q**
***pa*Pgb-AKS-(*R*)-5334**	**L55S**, Y57E, W59L, V60Q, R86S, P151Q, **S156W**
***pa*Pgb-AKS-(*R*)-5335**	L55S, Y57E, W59L, V60Q, R86S, P151Q, S156W, **P160L**, **A186E**

Nucleotide and amino acid sequences of AKS-G0 (*pa*Pgb-AKS-5329):

```
ATGGCGGTTCCCGGCTACGATTTTGGCAAAGTCCCGGATGCCCCAATCTCAGACGCGGATTTTGAGAGTT
TAAAAAAAACCGTGATGTGGGGTGAGGAAGATGAGAAATATCGCAAAATGGCTTGCGAAGCCTTAAAGGG
TCAAGTAGAAGATATTTTAGATTTGTGGGATGGCCTGCAGGGAAGCAATCAACACCTTATCTACTACTTC
GGTGATAAGAGTGGTCGTCCAATTCCGCAATACCTGGAAGCGGTCCGCAAGCGTTTCGGGTTGTGGATCA
TTGATACATTGTGTAAGCCACTGGACCGCCAGTGGTTGAATTACATGTACGAAATTGGCCTTCGCCATCA
CCGTACCAAGAAAGGGAAGACAGATGGCGTAGATACTGTTGAACATATCCCATTACGCTACATGATTGCT
TTCATCGCTCCCATCGGTCTGACTATTAAGCCGATCTTGGAAAAATCGGGACATCCGCCAGAGGCCGTGG
AGCGTATGTGGGCAGCATGGGTTAAGTTGGTGGTGTTACAGGTAGCTATCTGGTCGTACCCCTATGCAAA
GACGGGCGAATGGCTCGAGCACCACCACCACCACCAC
```

```
MAVPGYDFGKVPDAPISDADFESLKKTVMWGEEDEKYRKMACEALKGQVEDILDLWDGLQGSNQHLIYYF
GDKSGRPIPQYLEAVRKRFGLWIIDTLCKPLDRQWLNYMYEIGLRHHRTKKGKTDGVDTVEHIPLRYMIA
FIAPIGLTIKPILEKSGHPPEAVERMWAAWVKLVVLQVAIWSYPYAKTGEWLEHHHHHH
```

Nucleotide and amino acid sequences of AKS-(R)-G1 (*pa*Pgb-AKS-(R)-5333):

```
ATGGCGGTTCCCGGCTACGATTTTGGCAAAGTCCCGGATGCCCCAATCTCAGACGCGGATTTTGAGAGTT
TAAAAAAAACCGTGATGTGGGGTGAGGAAGATGAGAAATATCGCAAAATGGCTTGCGAAGCCTTAAAGGG
TCAAGTAGAAGATATTTTAGATTTGTGGGAGGGCCTGCAGGGAAGCAATCAACACCTTATCTACTACTTC
GGTGATAAGAGTGGTCGTCCAATTCCGCAATACCTGGAAGCGGTCAGCAAGCGTTTCGGGTTGTGGATCA
TTGATACATTGTGTAAGCCACTGGACCGCCAGTGGTTGAATTACATGTACGAAATTGGCCTTCGCCATCA
```

CCGTACCAAGAAAGGGAAGACAGATGGCGTAGATACTGTTGAACATATCCCATTACGCTACATGATTGCT
TTCATCGCTCCCATCGGTCTGACTATTAAGCAGATCTTGGAAAAATCGGGACATCCGCCAGAGGCCGTGG
AGCGTATGTGGGCAGCATGGGTTAAGTTGGTGGTGTTACAGGTAGCTATCTGGTCGTACCCCTATGCAAA
GACGGGCGAATGGCTCGAGCACCACCACCACCACCAC

MAVPGYDFGKVPDAPISDADFESLKKTVMWGEEDEKYRKMACEALKGQVEDILDLWEGLQGSNQHLIYYF
GDKSGRPIPQYLEAVSKRFGLWIIDTLCKPLDRQWLNYMYEIGLRHHRTKKGKTDGVDTVEHIPLRYMIA
FIAPIGLTIKQILEKSGHPPEAVERMWAAWVKLVVLQVAIWSYPYAKTGEWLEHHHHHH

Nucleotide and amino acid sequences of AKS-(R)-G2 (*pa*Pgb-AKS-(R)-5334):

ATGGCGGTTCCCGGCTACGATTTTGGCAAAGTCCCGGATGCCCCAATCTCAGACGCGGATTTTGAGAGTT
TAAAAAAAACCGTGATGTGGGGTGAGGAAGATGAGAAATATCGCAAAATGGCTTGCGAAGCCTTAAAGGG
TCAAGTAGAAGATATTTTAGATTCGTGGGAGGGCCTGCAGGGAAGCAATCAACACCTTATCTACTACTTC
GGTGATAAGAGTGGTCGTCCAATTCCGCAATACCTGGAAGCGGTCAGCAAGCGTTTCGGGTTGTGGATCA
TTGATACATTGTGTAAGCCACTGGACCGCCAGTGGTTGAATTACATGTACGAAATTGGCCTTCGCCATCA
CCGTACCAAGAAAGGGAAGACAGATGGCGTAGATACTGTTGAACATATCCCATTACGCTACATGATTGCT
TTCATCGCTCCCATCGGTCTGACTATCAAGCAGATCTTGGAAAAATGGGGACATCCGCCAGAGGCCGTGG
AGCGTATGTGGGCAGCATGGGTTAAGTTGGTGGTGTTACAGGTAGCTATCTGGTCGTACCCCTATGCAAA
GACGGGCGAATGGCTCGAGCACCACCACCACCACCAC

MAVPGYDFGKVPDAPISDADFESLKKTVMWGEEDEKYRKMACEALKGQVEDILDSWEGLQGSNQHLIYYF
GDKSGRPIPQYLEAVSKRFGLWIIDTLCKPLDRQWLNYMYEIGLRHHRTKKGKTDGVDTVEHIPLRYMIA
FIAPIGLTIKQILEKWGHPPEAVERMWAAWVKLVVLQVAIWSYPYAKTGEWLEHHHHHH

Nucleotide and amino acid sequences of AKS-(R)-G3 (*pa*Pgb-AKS-(R)-5333):

ATGGCGGTTCCCGGCTACGATTTTGGCAAAGTCCCGGATGCCCCAATCTCAGACGCGGATTTTGAGAGTT
TAAAAAAAACCGTGATGTGGGGTGAGGAAGATGAGAAATATCGCAAAATGGCTTGCGAAGCCTTAAAGGG
TCAAGTAGAAGATATTTTAGATTCGTGGGAGGGCCTGCAGGGAAGCAATCAACACCTTATCTACTACTTC
GGTGATAAGAGTGGTCGTCCAATTCCGCAATACCTGGAAGCGGTCAGCAAGCGTTTCGGGTTGTGGATCA
TTGATACATTGTGTAAGCCACTGGACCGCCAGTGGTTGAATTACATGTACGAAATTGGCCTTCGCCATCA
CCGTACCAAGAAAGGGAAGACAGATGGCGTAGATACTGTTGAACATATCCCATTACGCTACATGATTGCT
TTCATCGCTCCCATCGGTCTGACTATCAAGCAGATCTTGGAAAAATGGGGACATCCGCTAGAGGCCGTGG
AGCGTATGTGGGCAGCATGGGTTAAGTTGGTGGTGTTACAGGTAGCTATCTGGTCGTACCCCTATGAAAA
GACGGGCGAATGGCTCGAGCACCACCACCACCACCAC

MAVPGYDFGKVPDAPISDADFESLKKTVMWGEEDEKYRKMACEALKGQVEDILDSWEGLQGSNQHLIYYF
GDKSGRPIPQYLEAVSKRFGLWIIDTLCKPLDRQWLNYMYEIGLRHHRTKKGKTDGVDTVEHIPLRYMIA
FIAPIGLTIKQILEKWGHPLEAVERMWAAWVKLVVLQVAIWSYPYEKTGEWLEHHHHHH

Nucleotide and amino acid sequences of AKS-(S)-G1 (*pa*Pgb-AKS-(S)-5330):

ATGGCGGTTCCCGGCTACGATTTTGGCAAAGTCCCGGATGCCCCAATCTCAGACGCGGATTTTGAGAGTT
TAAAAAAAACCGTGATGTGGGGTGAGGAAGATGAGAAATATCGCAAAATGGCTTGCGAAGCCTTAAAGGG
TCAAGTAGAAAATATTTTAGATTTGTGGGATGGCCTGGCGGGAAGCAATCAACACCTTATCTACTACTTC
GGTGATAAGAGTGGTCGTCCAATTCCGCAATACCTGGAAGCGGGTCGCAAGCGTTTCGGGTTGTGGATCA
TTGATACATTGTGTAAGCCACTGGACCGCCAGTGGTTGAATTACATGTACGAAATTGGCCTTCGCCATCA
CCGTACCAAGAAAGGGAAGACAGATGGCGTAGATACTGTTGAACATGTCCCATTACGCTACATGATTGCT
TTCATCGCTCCCATCGGTCTGACTATTAAGCCGATCTTGGAAAAATCGGGACATCCGCCAGAGGCCGTGG
AGCGTATGTGGGCAGCATGGGTTAAGTTGGTGGTGTTACAGGTAGCTATCTGGTCGTACCCCTATGCAAA
GACGGGCGAATGGCTCGAGCACCACCACCACCACCAC

MAVPGYDFGKVPDAPISDADFESLKKTVMWGEEDEKYRKMACEALKGQVENILDLWDGLAGSNQHLIYYF
GDKSGRPIPQYLEAGRKRFGLWIIDTLCKPLDRQWLNYMYEIGLRHHRTKKGKTDGVDTVEHVPLRYMIA
FIAPIGLTIKPILEKSGHPPEAVERMWAAWVKLVVLQVAIWSYPYAKTGEWLEHHHHHH

Nucleotide and amino acid sequences of AKS-(S)-G2 (*pa*Pgb-AKS-(S)-5331):

ATGGCGGTTCCCGGCTACGATTTTGGCAAAGTCCCGGATGCCCCAATCTCAGACGCGGATTTTGAGAGTT
TAAAAAAAACCGTGATGTGGGGTGAGGAAGATGAGAAATATCGCAAAATGGCTTGCGAAGCCTTAAAGGG
TCAAGTAGAAAATATTTTAGATCCGTGGGATGGCCTGGCGGGAAGCAATCAACACCTTATCTACTACTTC
GGTGATAAGAGTGGTCGTCCAATTCCGCAATACCTGGAAGCGGGTCGCAAGCGTTTCGGGTTGTGGATCA
TTGATACATTGTGTAAGCCACTGGACCGCCAGTGGTTGAATTACATGTACGAACAGGGCCTTCGCCATCA
CCGTACCAAGAAAGGGAAGACAGATGGCGTAGATACTGTTGAACATGTCCCATTACGCTACATGATTGCT
TTCATCGCTCCCATCGGTCTGACTATTAAGCCGAACTTGGAAAAATCGGGATATCCGCCAGAGGCCGTGG
AGCGTATGTGGGCAGCATGGGTTAAGTTGGTGGTGTTACAGGTAGCTATCTGGTCGTACCCCTATGCAAA
GACGGGCGAATGGCTCGAGCACCACCACCACCACCAC

MAVPGYDFGKVPDAPISDADFESLKKTVMWGEEDEKYRKMACEALKGQVENILDPWDGLAGSNQHLIYYF
GDKSGRPIPQYLEAGRKRFGLWIIDTLCKPLDRQWLNYMYEQGLRHHRTKKGKTDGVDTVEHVPLRYMIA
FIAPIGLTIKPNLEKSGYPPEAVERMWAAWVKLVVLQVAIWSYPYAKTGEWLEHHHHHH

Nucleotide and amino acid sequences of AKS-(S)-G3 (*pa*Pgb-AKS-(S)-5332):

ATGGCGGTTCCCGGCTACGATTTTGGCAAAGTCCCGGATGCCCCAATCTCAGACGCGGATTTTGAGAGTT
TAAAAAAAACCGTGATGTGGGGTGAGGAAGATGAGAAATATCGCAAAATGGCTTGCGAAGCCTTAAAGGG
TCAAGTAGAAAATATTTTAGATCCGTGGGATGGCCTGGCGGGAAGCAATCAACACCTTATCTACTACTTC
GGTGATAAGAGTGGTCGTCCAATTCCGCAATACCTGGAAGCGGGTCGCAAGCGTTTCGGGTTGTGGATCA
TTGATACATTGTGTAAGCCACTGGACCGCCAGTGGTTGAATTACACGTACGAACAGGGCCTTCGCCATCA

CCGTACCAAGAAAGGGAAGACAGATGGCGTAGATACTGTTGAACATGTCCCATTACGCTACATGATTGCT
TTCATCGCTCCCATCGGTCTGACTATTAAGCCGAACTTGGAAAAATCGAGATATCCGCCAGAGGCCGTGG
AGCGTATGTGGGCAGCATGGGTTAAGTTGGTGGTGTTACAGGTAGCTATCTGGTCGTACCCCTATGCAAA
GACGGGCGAATGGCTCGAGCACCACCACCACCACCAC

MAVPGYDFGKVPDAPISDADFESLKKTVMWGEEDEKYRKMACEALKGQVENILDPWDGLAGSNQHLIYYF
GDKSGRPIPQYLEAGRKRFGLWIIDTLCKPLDRQWLNYTYEQGLRHHRTKKGKTDGVDTVEHVPLRYMIA
FIAPIGLTIKPNLEKSRYPPEAVERMWAAWVKLVVLQVAIWSYPYAKTGEWLEHHHHHH

X. Computational Studies

(A) Computational Method

All DFT calculations were performed using the Gaussian16 software[8] on the Hoffman2 Cluster at UCLA. A truncated computational model has been used, which includes the porphyrin pyrrole core, the Fe(II) center and a 4-methyl imidazole molecule to mimic the axial histidine. This model is similar to those used in previous computational mechanistic studies of heme-dependent enzymatic reactions.[9–11] Geometry optimizations were performed using dispersion corrected (U)B3LYP-D3(BJ)[12–15] density functional in combination with the SDD basis set for Fe and 6-31G(d) for other atoms. All the optimized transition states were confirmed by the intrinsic reaction coordinate (IRC) calculations, which connect the corresponding reactants and intermediates (or intermediates and products). Single-point energy calculations were carried out at Def2TZVP level, using the CPCM solvation model[16,17] with diethyl ether solvent to simulate the dielectric permittivity in the enzyme active site.[18–20] The stable=opt keyword was used in all oss, triplet and quintet single point calculations to confirm the stability of the wave function. Therefore, all energies are reported at the (U)B3LYP-D3(BJ) / Def2TZVP / CPCM (diethyl ether) // (U)B3LYP-D3(BJ) / 631G(d)+SDD(Fe) / CPCM (diethyl ether) level of theory. This methodology is similar to that previously used to investigate the mechanism of iron porphyrin-catalyzed nitrene, carbene and oxo transfer reactions.[10,21–26]

Hirshfield spin population analysis[27] was performed with Multiwfn software package[28,29], and the visualization of the Hirshfeld spin population analysis results are presented with VMD visualization software[30]. The 3D diagrams of molecules were generated using CYLview,[31] and certain fragments of the catalyst are simplified for clarity.

(B) Reaction energy profile and spin-state analysis

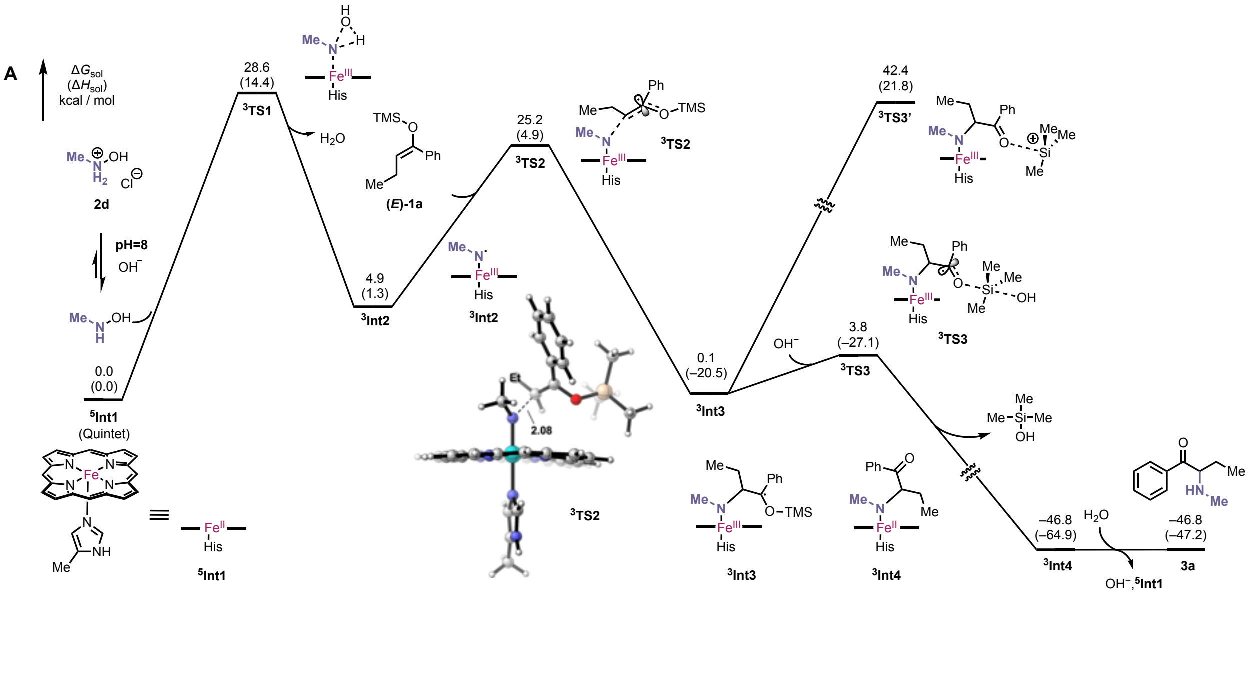

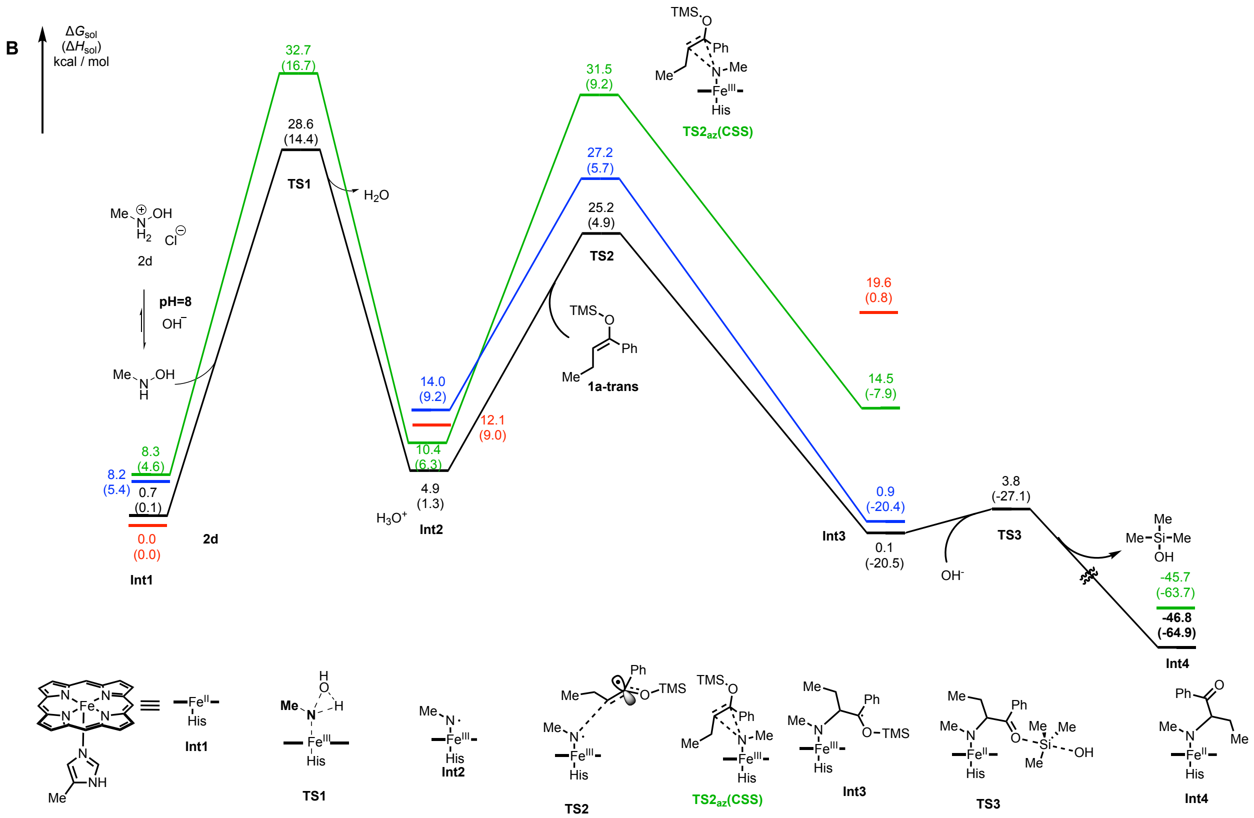

Figure S3. DFT calculations were carried out at (U)B3LYP-D3(BJ) / DEF2TZVP / CPCM(Et_2O) // (U)B3LYP-D3(BJ) / 6-31G*-SDD(Fe) / CPCM(Et_2O) level of theory. Energies are given in kcal·mol^{-1}. (A) DFT computed free energy profile for the iron(II)‒porphyrin catalyzed N-alkylated nitrene-induced amination using a truncated computational model and substrate 1a-trans. Key distance is given in Å. (B) Calculated energy profiles in the singlet closed-shell (CSS), singlet open-shell (OSS), triplet, and quintet electronic states in green, blue, black, and red, respectively.

The activation of hydroxylamine could proceed through the current model, although with a relatively high energy barrier (ΔG‡ = 28.6 kcal/mol, Figure S3A). In the enzymatic environment, residues surrounding the active site may further assist in stabilizing the transition state. The putative iron‒nitrenoid active species was identified as a triplet ground state, consistent with previous reported iron‒nitrenoid species.[32] Three mechanistic scenarios were initially considered based on the substrate architecture. Single-electron transfer (Figure S5)[33] and aziridination (Figure S6) were excluded due to prohibitively high barriers. Radical clock experiments afforded the ring-retaining product **3b** as the sole product with 24% yield (Figure S2), consistent with the computational results. Instead, the preferred pathway proceeds *via* direct C–N coupling (TS2 ΔG‡ = 25.2 kcal/mol, in the triplet state), forming an α-silyl ether radical intermediate **Int3**, which subsequently undergoes deprotection and intramolecular electron transfer to afford the final *N*-alkylated intermediate **Int4**. Protonation of **Int4** then readily regenerates **Int1** and releases the α-aminoketone product **3a** (Figure S3A).

To better understand the C–N bond forming process, we performed Hirshfeld spin population analysis (Figure 4) with Multiwfn.[27–29] The population on C1 and C2 indicates that TS1 is a radical

addition transition state, instead of a nitrene electrophilic addition to the enol. In comparison with previously reported examples, the presence of the *N*-alkyl substituent markedly increases the spin density localized on the nitrene N atom, rendering it higher than that on Fe (Figure 4). For instance, in Int2 the spin density on N is 0.99 (NMe) versus 0.84 (NH), while that on Fe is 0.85 (NMe) versus 1.11 (NH). A similar trend is observed in TS2 (0.84 vs 0.75 on N; 0.63 vs 0.91 on Fe). This redistribution indicates that the substituent stabilizes the unpaired electron on N, thereby enhancing its radical character. In summary, these computational analyses identify direct C–N coupling as the operative pathway and demonstrate that *N*-alkyl substitution redistributes spin density from Fe to the nitrene N atom, thereby stabilizing the radical center. These insights highlight substitution as a powerful lever for tuning spin distribution and reactivity, offering guidance for the future design of nitrene-transfer reactions.

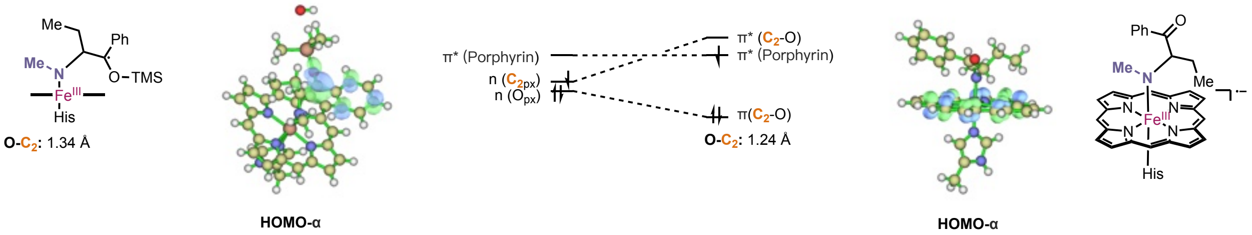

Figure S4. Atomic spin populations along reaction coordinate from TS3 to Int4 and conceptual molecular orbital diagram.

To understand the intramolecular electron-transfer accompanying TMS dissociation from the heme–substrate complex, we compared the electronic structures of Int3 and Int4 (Figure S4). The α-HOMO of Int3 is primarily contributed by the p orbital of C2 that is perpendicular to the Ph–C–O plane, together with the π system of the conjugated phenyl ring. In contrast, the α-HOMO of Int4 is mainly distributed over the porphyrin framework, indicating transfer of the unpaired electron from C2 to the porphyrin π system, accompanied by deprotection and formation of the C2–O double bond. This electronic redistribution can be rationalized by changes in orbital interactions. Upon dissociation of the TMS group, the stabilization provided by Si to the oxygen p orbital is lost. Consequently, the oxygen lone pair seeks alternative stabilizing interactions, leading to enhanced overlap with the p orbital on C2 that initially hosts the unpaired electron. This interaction converts the original nonbonding orbital into an antibonding orbital, raising its energy and promoting electron transfer to the porphyrin π* orbitals. This process may be further facilitated by π–π stacking interactions between the phenyl ring of the substrate and the porphyrin macrocycle.[34]

(C) Single electron oxidation

Although **Int2** exhibits electrophilicity and the silyl enol ether substrates appear nucleophilic, our calculations reveal that, with the hydrophobic environment of the enzyme active site, charge separation is highly disfavored due to an extremely high energy penalty.

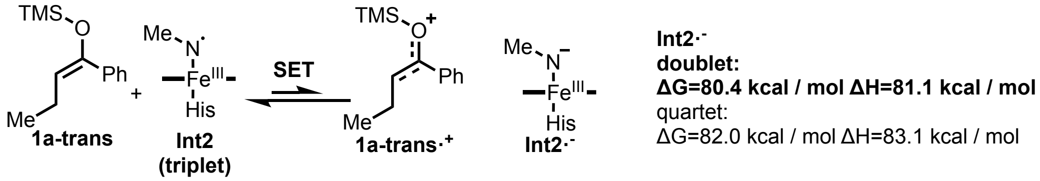

Figure S5. Free energy for single electron transfer.

(D) Aziridination

We investigated the aziridination mechanism with a comprehensive analysis of different spin states, including singlet closed-shell (CSS), singlet open-shell (OSS), triplet, and quintet electronic configurations. For each case, intrinsic reaction coordinate (IRC) calculations were performed to validate the transition states. Among them, only the CSS aziridination transition state (**TS1$_{az}$**) could be successfully located and confirmed via IRC. In contrast, both OSS and triplet states were found to converge instead to a transition state corresponding to C–N single bond formation (**TS1**), rather than aziridination. Despite extensive efforts, a quintet-state transition state could not be located for either **TS1** or **TS1$_{az}$**. The observed reactivity of the CSS state is consistent with the generally accepted behavior of singlet nitrenes in aziridination reactions.

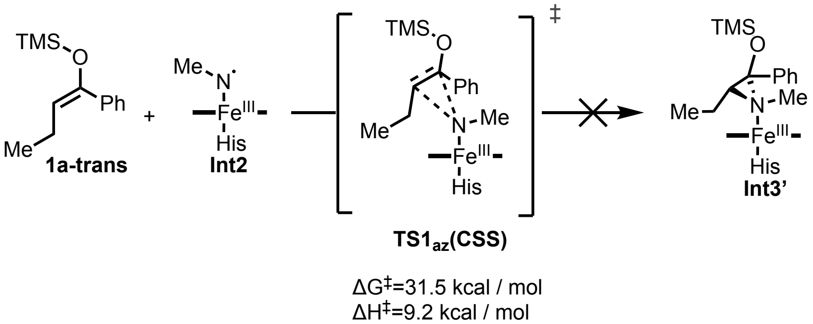

Figure S6. Free energy for aziridination transition state.

(E) Supplementary table

Table S5. Energies for **Figure S3** and **Figure S5**. Thermal correction to Gibbs Free Energy (TCG), Thermal correction to Enthalpy (TCE) were calculated with geometry optimization at the (U)B3LYP-D3(BJ) / DEF2TZVP / CPCM(Et_2O) level of theory, and the single point energy (E) was calculated at the // (U)B3LYP-D3(BJ) / 6-31G*-SDD / CPCM(Et_2O) level of theory. Gibbs free energies (G) in Hartree of the structures were calculated by adding up E and TCG, and enthalpies (H) in Hartree of the structures were calculated by adding up E and TCE. Spin states are listed for clarification: css: close-shell singlet; oss: open-shell singlet; t: triplet; q: quintet.

Structure	TCE	TCG	SPE	G	H	charge	spin multiplicity
Int1	0.402252	0.325288	-2518.367789	-2518.042501	-2517.965537	0	css

	0.401835	0.323442	-2518.366086	-2518.042644	-2517.964251	0	css
	0.401667	0.319665	-2518.374348	-2518.054683	-2517.972681	0	t
	0.400378	0.317467	-2518.373191	-2518.055724	-2517.972813	0	q
Int2	0.447039	0.361797	-2613.004408	-2612.642611	-2612.557369	0	oss
	0.447431	0.363197	-2613.000125	-2612.636928	-2612.552694	0	css
	0.446973	0.360699	-2613.012194	-2612.651495	-2612.565221	0	t
	0.444271	0.357330	-2612.997246	-2612.639916	-2612.552975	0	q
TS1	0.765327	0.641101	-3485.546653	-3484.905552	-3484.781326	0	oss
	0.765118	0.638993	-3485.547712	-3484.908719	-3484.782594	0	t
TS1$_{az}$	0.765650	0.642669	-3485.541332	-3484.898663	-3484.775682	0	css
Int3	0.768365	0.642772	-3485.591472	-3484.948700	-3484.823107	0	t
	0.768217	0.645518	-3485.571255	-3484.925737	-3484.803038	0	css
	0.768305	0.643766	-3485.591203	-3484.947437	-3484.822898	0	oss
	0.763947	0.635362	-3485.553040	-3484.917678	-3484.789093	0	q
TS2	0.780639	0.651914	-3561.519463	-3560.867549	-3560.738824	0	t
Int4	0.643944	0.536663	-3076.291999	-3075.755336	-3075.648055	-1	t
	0.644151	0.536866	-3076.290438	-3075.753572	-3075.646287	-1	css
Int2$^{-\cdot}$	0.443344	0.356686	-2613.09933	-2612.74264	-2612.65598	-1	doublet
	0.442726	0.355578	-2613.09561	-2612.74004	-2612.65289	-1	quartet
NH_2MeOH^+	0.089211	0.058751	-171.52956	-171.4708124	-171.44035	1	css
NHMeOH	0.07381	0.043406	-171.11258	-171.0691745	-171.03877	0	css
1a-trans	0.316700	0.250109	-872.539755	-872.289646	-872.223055	0	css
1a-trans$^{+\cdot}$	0.316981	0.249569	-872.319966	-872.070397	-872.002985	1	doublet
OH^-	0.011331	-0.008256	-75.91649	-75.92474575	-75.905159	-1	css
H_2O	0.024915	0.003465	-76.469139	-76.46567369	-76.444224	0	css
H_3O^+	0.038582	0.016656	-76.834243	-76.81758727	-76.795661	1	css
TMSOH	0.134969	0.092951	-485.285845	-485.1928946	-485.15088	0	css

(G) Cartesian coordinates of the structures

Int1-css

0 1

C	2.63807500	-1.23322500	-1.04326600
C	3.31745000	-2.50779500	-1.11496800
C	2.36142100	-3.46935200	-0.98390200
C	1.09758800	-2.78245300	-0.83377600
H	4.38451100	-2.63029800	-1.25149700
H	2.48053900	-4.54545700	-0.99098600
C	3.27243100	0.00006200	-1.11776100
H	4.35074300	0.00008300	-1.24552500
C	2.63802800	1.23332400	-1.04325800
C	3.31735400	2.50792000	-1.11494900
C	2.36128700	3.46944000	-0.98388800
H	4.38441100	2.63046500	-1.25147100
C	1.09748000	2.78249200	-0.83377200
H	2.48036400	4.54555000	-0.99096800
C	-0.12611800	3.41902200	-0.67510200
H	-0.12402400	4.50481100	-0.65754100
C	-1.35466800	2.78268900	-0.55813100
C	-2.62019900	3.46962800	-0.42475200
C	-3.58181700	2.50824800	-0.33976400
H	-2.73791300	4.54577200	-0.40654500
C	-2.90368500	1.23359200	-0.41981700
H	-4.65262600	2.63113500	-0.23697800
N	1.28319100	1.41834300	-0.87626500
N	1.28324700	-1.41829700	-0.87626500
N	-1.54530000	1.41863800	-0.55795000
Fe	-0.11633400	-0.00000300	-0.59361800
C	-3.53854000	-0.00007000	-0.35062600
H	-4.61835300	-0.00009200	-0.23603400
C	-2.90363500	-1.23370800	-0.41980200
C	-3.58171600	-2.50839000	-0.33973700
C	-2.62006000	-3.46973200	-0.42471900
H	-4.65251900	-2.63131800	-0.23694800
C	-1.35455700	-2.78274400	-0.55811400
H	-2.73773100	-4.54588100	-0.40650100
N	-1.54524300	-1.41870100	-0.55794100
C	-0.12598400	-3.41903000	-0.67509500
H	-0.12384800	-4.50481900	-0.65753100
C	1.28366400	-0.00002100	2.04012900
C	-0.88694900	0.00003500	2.22453700
C	1.01837800	0.00002400	3.38667200
H	2.23381900	-0.00004500	1.53025900
H	-1.94123600	0.00005500	1.99902400
N	0.09315800	0.00000700	1.33467500
N	-0.36493200	0.00000500	3.47778600
H	-0.90055700	0.00000300	4.33434100
C	1.91279100	0.00004400	4.58024900
H	1.75253600	0.88551700	5.20732700
H	1.75257000	-0.88543700	5.20732500
H	2.95704100	0.00006400	4.25771200

Int1-t

0 3

C	2.60439300	-1.23298100	-1.17722200
C	3.27777300	-2.50803000	-1.26681800
C	2.32247900	-3.46763000	-1.10817400
C	1.06475800	-2.78035400	-0.92267400
H	4.34022600	-2.63365700	-1.43191200
H	2.44056200	-4.54382400	-1.11606100
C	3.23728500	-0.00009400	-1.27279400
H	4.31090700	-0.00012200	-1.43344900
C	2.60445300	1.23282500	-1.17724100
C	3.27789500	2.50784000	-1.26685500
C	2.32264700	3.46748900	-1.10823500
H	4.34035500	2.63341300	-1.43194300
C	1.06489200	2.78027700	-0.92272900
H	2.44078200	4.54367700	-1.11614100
C	-0.15597900	3.41577500	-0.73091500
H	-0.15366000	4.50141900	-0.71649900
C	-1.38225900	2.78076300	-0.57506800
C	-2.64203800	3.46797600	-0.40510600
C	-3.60394500	2.50837500	-0.29155100
H	-2.75793900	4.54419900	-0.38187200
C	-2.93215500	1.23326000	-0.39079700
H	-4.67062300	2.63426500	-0.15648100
N	1.25551100	1.41838700	-0.96931100
N	1.25544300	-1.41847400	-0.96928100
N	-1.57826000	1.41875300	-0.56486100
Fe	-0.14389000	-0.00000700	-0.65009300
C	-3.56649900	0.00007900	-0.30595300
H	-4.64264100	0.00010600	-0.16293500
C	-2.93221300	-1.23313500	-0.39076400
C	-3.60406200	-2.50821500	-0.29147900
C	-2.64220100	-3.46786500	-0.40501300
H	-4.67074500	-2.63405200	-0.15640000
C	-1.38239100	-2.78071500	-0.57500100
H	-2.75815300	-4.54408100	-0.38175000
N	-1.57832800	-1.41869600	-0.56483000
C	-0.15614200	-3.41579000	-0.73084000
H	-0.15387500	-4.50143300	-0.71640000
C	1.38024700	-0.00007500	2.24401700
C	-0.77142000	0.00005600	2.48412300
C	1.15824200	0.00004700	3.59992600
H	2.31910600	-0.00014800	1.70962400
H	-1.83379000	0.00009400	2.29024600
N	0.17693300	0.00001100	1.56585300
N	-0.22334500	0.00002100	3.72887000
H	-0.73459300	0.00003400	4.60078300
C	2.08401300	0.00010500	4.76974100
H	1.94175500	0.88535900	5.40176600
H	1.94173400	-0.88507900	5.40185700
H	3.11945600	0.00007400	4.41942600

Int1-q

0 5

C	2.64389300	1.30093800	-1.13468100
C	4.03695900	0.96155500	-1.37205000
C	4.11313200	-0.40040700	-1.41094100

C	2.76718300	-0.90585300	-1.19803200
H	4.83720200	1.67911400	-1.50370200
H	4.98794500	-1.01562800	-1.58069600
C	2.11833700	2.59363200	-1.05631800
H	2.82705500	3.41145300	-1.15249000
C	0.77468300	2.95555200	-0.88623400
C	0.27574000	4.31092400	-0.80625500
C	-1.07775900	4.22972000	-0.62163600
H	0.88561100	5.20243500	-0.88237000
C	-1.41727200	2.82400200	-0.58772200
H	-1.78618800	5.04212300	-0.51825700
C	-2.70570100	2.30456000	-0.39975800
H	-3.50487800	3.03232500	-0.28995400
C	-3.07857000	0.95808700	-0.35745700
C	-4.43446500	0.45386100	-0.21988600
C	-4.35802600	-0.90836700	-0.25970600
H	-5.31942300	1.07016800	-0.12146300
C	-2.95479200	-1.24920400	-0.42188100
H	-5.16821100	-1.62441900	-0.20016200
N	-0.27583400	2.08006100	-0.76080500
N	1.91020900	0.14899800	-1.01938100
N	-2.21082500	-0.09828100	-0.46130200
Fe	-0.11701300	0.02002300	-0.51058500
C	-2.43450700	-2.54111700	-0.54106300
H	-3.14773200	-3.35821500	-0.47706200
C	-1.09681200	-2.90229300	-0.75413600
C	-0.60258000	-4.25700400	-0.86859100
C	0.75110600	-4.17575200	-1.05197200
H	-1.21556100	-5.14806800	-0.81552600
C	1.09525500	-2.77072200	-1.05100800
H	1.45652300	-4.98762700	-1.17758800
N	-0.04611800	-2.02701500	-0.87881800
C	2.38944300	-2.25149300	-1.19537700
H	3.18472500	-2.97814600	-1.33621000
C	1.36869600	-0.04841400	2.31822200
C	-0.79000100	-0.10819000	2.53798600
C	1.13174100	-0.11714600	3.66862200
H	2.31056400	-0.00276900	1.79227600
H	-1.84916900	-0.12388100	2.33083100
N	0.16932300	-0.04365600	1.62948500
N	-0.25091800	-0.15373800	3.78203300
H	-0.77036600	-0.20642600	4.64770500
C	2.04559900	-0.15240600	4.84688300
H	1.87757900	0.69977100	5.51650200
H	1.91408700	-1.06880600	5.43487300
H	3.08399200	-0.11536400	4.50795500

Int2-oss

0 1

C	2.59489400	-1.23418200	-1.15067300
C	3.26391600	-2.51070400	-1.29188300
C	2.33222300	-3.47142100	-1.03858700
C	1.09306500	-2.78139100	-0.74653200
H	4.30865300	-2.63324600	-1.54792200
H	2.45142500	-4.54739700	-1.04572800
C	3.21310300	-0.00002500	-1.31360100
H	4.26579800	-0.00009500	-1.57862100
C	2.59523600	1.23420400	-1.14998200
C	3.26464500	2.51061500	-1.29029100
C	2.33317700	3.47146500	-1.03665800
H	4.30946000	2.63302800	-1.54607400
C	1.09375600	2.78164300	-0.74528200
H	2.45272300	4.54740500	-1.04317800
C	-0.10684700	3.41406400	-0.44663300
H	-0.10535900	4.49949700	-0.42552100
C	-1.31250100	2.77730700	-0.17893400
C	-2.54589800	3.46789000	0.12765600
C	-3.49467900	2.51103300	0.33061700
H	-2.65123700	4.54419500	0.17659000
C	-2.84232100	1.23303100	0.15185100
H	-4.53967800	2.63834100	0.58302000
N	1.27993400	1.42583500	-0.81560200
N	1.27964200	-1.42560100	-0.81606600
N	-1.51689000	1.41920000	-0.15776500
Fe	-0.15570300	0.00024200	-0.58687800
C	-3.46786800	0.00040500	0.29462300
H	-4.52418400	0.00054100	0.54409300
C	-2.84270000	-1.23236200	0.15134300
C	-3.49526600	-2.51029400	0.32973500
C	-2.54666500	-3.46725800	0.12639000
H	-4.54027800	-2.63754500	0.58210900
C	-1.31316000	-2.77680800	-0.18003300
H	-2.65224800	-4.54355500	0.17497800
N	-1.51732000	-1.41867000	-0.15842200
C	-0.10764000	-3.41368400	-0.44804900
H	-0.10635400	-4.49912600	-0.42741300
C	1.61524400	-0.00014100	2.04149300
C	-0.50462300	-0.00149300	2.49900600
C	1.52916700	-0.00117800	3.41129900
H	2.49539800	0.00077700	1.41814000
H	-1.57998500	-0.00189500	2.41535500
N	0.34768200	-0.00034800	1.48927200
N	0.16765300	-0.00206700	3.67894900
H	-0.25541200	-0.00295400	4.59672700
C	2.57021300	-0.00140700	4.47961100
H	2.49301600	0.88342800	5.12332400
H	2.49383700	-0.88715700	5.12216300
H	3.56400600	-0.00064800	4.02444900
N	-0.55259800	0.00068800	-2.26172700
C	-1.85190400	0.00115800	-2.85399000
H	-1.78295300	0.00091300	-3.95125800
H	-2.43274300	-0.88187400	-2.54073800
H	-2.43171500	0.88502300	-2.54113800

Int2-css

0 1

C	2.58039100	-1.30006900	-1.07879100
C	3.21852500	-2.59418900	-1.20226100
C	2.25389600	-3.52972800	-0.98038000
C	1.02586600	-2.80701000	-0.72163700
H	4.26671600	-2.74442200	-1.42740900
H	2.34383200	-4.60852400	-0.98746400

C 3.23242900 -0.08290900 -1.23550500
H 4.29153200 -0.11017000 -1.47189400
C 2.63724300 1.16661400 -1.10895500
C 3.33621500 2.42623000 -1.25765800
C 2.41753900 3.41067400 -1.05297700
H 4.39020300 2.52199200 -1.48559100
C 1.15690200 2.75210900 -0.78009700
H 2.55888700 4.48363100 -1.07972500
C -0.03633000 3.41480900 -0.51936500
H -0.01203700 4.50017600 -0.52083800
C -1.25720700 2.80973000 -0.24712300
C -2.47246100 3.53304500 0.05642400
C -3.43900600 2.60100100 0.28872500
H -2.55383500 4.61210500 0.08661400
C -2.81647500 1.30620000 0.12644000
H -4.47804700 2.75565900 0.55019800
N 1.31857900 1.39189900 -0.81425300
N 1.25289400 -1.45695200 -0.77884700
N -1.49162000 1.45688200 -0.20291000
Fe -0.15340700 0.00351100 -0.60380300
C -3.46938200 0.08981300 0.29279100
H -4.52221800 0.11780500 0.55500000
C -2.87641300 -1.15864200 0.14757100
C -3.55981100 -2.41930000 0.33142200
C -2.63887800 -3.40017400 0.11509700
H -4.60493000 -2.51991000 0.59521200
C -1.39048000 -2.74060200 -0.19904100
H -2.77137900 -4.47346900 0.16395100
N -1.56033000 -1.37747700 -0.17815100
C -0.19870700 -3.40744800 -0.45572700
H -0.22592400 -4.49262600 -0.43848000
C 1.58149900 -0.00995200 2.06645300
C -0.54432200 0.03985500 2.48825900
C 1.47326300 0.00210500 3.43460600
H 2.47209700 -0.03431900 1.45866900
H -1.61791900 0.06235100 2.38782500
N 0.32375900 0.01361700 1.49203600
N 0.10781700 0.03399700 3.67955600
H -0.33024100 0.05026100 4.59012400
C 2.49593500 -0.01340300 4.52040700
H 2.42802900 0.87772400 5.15648900
H 2.38886600 -0.89233700 5.16795600
H 3.49703600 -0.03839200 4.08223300
N -0.41385600 -0.02592200 -2.28173300
C -1.72651800 -0.08563500 -2.85328300
H -1.74984000 0.50058900 -3.78499300
H -1.88285500 -1.13690800 -3.15427800
H -2.56236900 0.20645000 -2.20695400

In2-t

0 3
C 2.58624100 -1.25389200 -1.155278
C 3.24954300 -2.53420100 -1.2874460
C 2.31794400 -3.48884300 -1.0119970
C 1.08403400 -2.79221800 -0.7154650
H 4.29099000 -2.66325600 -1.5534960
H 2.43428700 -4.56513300 -1.0062070
C 3.20956600 -0.02427200 -1.3327920
H 4.26003000 -0.03284100 -1.6064180
C 2.60223800 1.21579600 -1.1717590
C 3.28071000 2.48503700 -1.3260890
C 2.35974500 3.45579000 -1.0701920
H 4.32369800 2.59706600 -1.5938580
C 1.11729000 2.77983600 -0.7638410
H 2.48868200 4.53055500 -1.0855730
C -0.07598600 3.42630100 -0.4671860
H -0.06459800 4.51179300 -0.4542430
C -1.28766700 2.79999800 -0.2015870
C -2.52136100 3.49708700 0.0904540
C -3.47384300 2.54393900 0.2933530
H -2.62423500 4.57398900 0.1308390
C -2.82259800 1.26256100 0.1289530
H -4.52052800 2.67568000 0.5364770
N 1.29059900 1.42054600 -0.8268530
N 1.27253300 -1.43669100 -0.8058060
N -1.49562300 1.44381800 -0.1724220
Fe -0.13778800 0.00225400 -0.5677040
C -3.45130000 0.03207000 0.2834410
H -4.50940700 0.03965900 0.5256030
C -2.83249900 -1.20711900 0.1665390
C -3.49774100 -2.47658200 0.3590990
C -2.55742700 -3.44555500 0.1732720
H -4.54563800 -2.59083600 0.6058660
C -1.31628600 -2.77013700 -0.1354960
H -2.67364000 -4.52007500 0.2349400
N -1.50723200 -1.41112700 -0.1302270
C -0.11491000 -3.41751700 -0.3985830
H -0.11772600 -4.50259900 -0.3648360
C 1.60252000 -0.01941300 2.0423490
C -0.51635900 0.07384900 2.5005720
C 1.51723500 0.01191400 3.4119190
H 2.48108400 -0.06629700 1.4185680
H -1.59086300 0.11365800 2.4168810
N 0.33487700 0.01958200 1.4915550
N 0.15748300 0.07094500 3.6796980
H -0.26439000 0.10558800 4.5973400
C 2.55886400 -0.00732700 4.4794380
H 2.51825400 0.89369000 5.1037020
H 2.44736900 -0.87516900 5.1409270
H 3.55125700 -0.05666000 4.0239400
N -0.64877600 -0.02137400 -2.2506750
C -1.90860800 -0.17531500 -2.8912560
H -1.80451500 -0.07167800 -3.9818530
H -2.34704600 -1.16575900 -2.6836760
H -2.63366800 0.57941900 -2.5440210

Int2-q

0 5
C 2.61122900 -1.25410600 -1.22776200
C 3.26890100 -2.53905300 -1.38060800
C 2.33425000 -3.49865400 -1.11430400
C 1.09797100 -2.80749600 -0.79663600

H	4.30783300	-2.67617900	-1.65300900
H	2.45895300	-4.57410400	-1.12598400
C	3.21309900	-0.00125300	-1.39601900
H	4.26229700	-0.00148300	-1.67695100
C	2.61128600	1.25189900	-1.22967600
C	3.26917100	2.53653600	-1.38403800
C	2.33458600	3.49662400	-1.11920400
H	4.30820300	2.67315800	-1.65631200
C	1.09811600	2.80606800	-0.80104600
H	2.45945700	4.57203900	-1.13221400
C	-0.11371100	3.42186500	-0.46527700
H	-0.11187400	4.50809200	-0.45326500
C	-1.32840900	2.80609800	-0.14406500
C	-2.55638800	3.49655000	0.20063100
C	-3.49851000	2.53949500	0.45201000
H	-2.66980100	4.57238400	0.24462900
C	-2.85667700	1.25272000	0.26426800
H	-4.53212600	2.68019800	0.74208700
N	1.30636500	1.45643200	-0.87691500
N	1.30640600	-1.45799800	-0.87423600
N	-1.55025200	1.45488800	-0.09071100
Fe	-0.19436500	-0.00058400	-0.68369400
C	-3.46009000	0.00046500	0.43029700
H	-4.50752300	0.00069200	0.71700600
C	-2.85663900	-1.25204200	0.26648500
C	-3.49858400	-2.53846300	0.45612100
C	-2.55655900	-3.49599500	0.20616900
H	-4.53222500	-2.67862900	0.74637000
C	-1.32849800	-2.80619400	-0.13949700
H	-2.67008600	-4.57175000	0.25175900
N	-1.55018500	-1.45488000	-0.08808700
C	-0.11390400	-3.42262800	-0.45987300
H	-0.11227800	-4.50883600	-0.44638000
C	1.71234900	0.00001000	2.06360800
C	-0.38659100	0.00550600	2.59255800
C	1.67602400	0.00286500	3.43695000
H	2.57117900	-0.00308200	1.40992500
H	-1.46486300	0.00765100	2.54714100
N	0.42805800	0.00171200	1.55414100
N	0.32495900	0.00633700	3.75215500
H	-0.06514700	0.00894300	4.68441600
C	2.75352200	0.00273300	4.46880200
H	2.70178100	0.88962300	5.11240300
H	2.69730400	-0.88065500	5.11682200
H	3.73121200	-0.00095600	3.97977700
N	-0.81008500	-0.00250100	-2.35780600
C	-2.15775500	-0.00543700	-2.81930900
H	-2.13019100	-0.00387700	-3.92181400
H	-2.71998000	-0.89865500	-2.50298800
H	-2.72463300	0.88401000	-2.50068900

TS1-oss

0 1

C	-0.053842	-2.288171	-1.015881
C	-0.302511	-3.668251	-0.660287
C	0.145631	-3.832397	0.616779
C	0.690230	-2.558501	1.032060
H	-0.759147	-4.402057	-1.311349
H	0.135240	-4.726460	1.227154
C	-0.399556	-1.718554	-2.234049
H	-0.916932	-2.349257	-2.949146
C	-0.151519	-0.406533	-2.611069
C	-0.543851	0.163529	-3.881359
C	-0.064727	1.439469	-3.906192
H	-1.105218	-0.361935	-4.643532
C	0.629527	1.647917	-2.654630
H	-0.148249	2.177470	-4.693869
C	1.342166	2.796829	-2.331532
H	1.344073	3.605731	-3.055330
C	2.060541	2.983070	-1.156510
C	2.807892	4.176866	-0.826189
C	3.339313	3.975469	0.4125220
H	2.901102	5.043175	-1.468755
C	2.916700	2.658602	0.8407760
H	3.958944	4.643026	0.997856
N	0.540462	0.521327	-1.872148
N	0.552233	-1.634593	0.025110
N	2.147830	2.072505	-0.132637
Fe	1.233704	0.269227	-0.006459
C	3.245430	2.094621	2.0679950
H	3.856655	2.692017	2.737726
C	2.860768	0.835608	2.5142190
C	3.222589	0.280556	3.8030210
C	2.675036	-0.965574	3.859932
H	3.817589	0.791780	4.549219
C	1.981005	-1.170961	2.606273
H	2.727041	-1.690931	4.661983
N	2.105926	-0.064617	1.808640
C	1.317203	-2.338291	2.252785
H	1.321664	-3.155424	2.967489
C	3.697836	-1.670490	-0.462855
C	3.634380	-0.058971	-1.913581
C	4.775329	-1.869371	-1.289326
H	3.377609	-2.255078	0.384852
H	3.349144	0.819856	-2.469718
N	2.999967	-0.545197	-0.861005
N	4.713463	-0.829301	-2.206120
H	5.360894	-0.669561	-2.965556
C	5.840529	-2.913403	-1.311582
H	5.841557	-3.473550	-2.254715
H	6.839398	-2.478836	-1.182427
H	5.675299	-3.624496	-0.498017
N	-0.186060	1.039209	0.822384
C	-0.788612	2.231270	0.279411
H	-1.621322	2.576106	0.903765
H	-0.034387	3.028159	0.271516
H	-1.163389	2.139031	-0.752288
C	-3.721206	1.135583	0.393098
C	-4.001171	1.461164	-0.952581
C	-4.873894	2.495509	-1.276150
C	-5.506977	3.227633	-0.267234
C	-5.253681	2.907585	1.068955

C	-4.373715	1.877978	1.398057
H	-3.510352	0.895217	-1.736215
H	-5.061183	2.732676	-2.320135
H	-6.192985	4.031347	-0.518723
H	-5.752918	3.456051	1.863238
H	-4.218534	1.631699	2.440207
C	-2.787771	0.035990	0.666886
C	-1.821098	-0.029871	1.658507
H	-1.263007	-0.958587	1.676617
C	-1.888493	0.715312	2.973063
H	-2.005428	1.790616	2.796736
H	-2.797293	0.398599	3.507094
C	-0.679968	0.471925	3.876500
H	0.231429	0.814291	3.386426
H	-0.797445	1.002849	4.828479
H	-0.561957	-0.594890	4.096924
O	-2.767979	-0.934063	-0.322651
Si	-3.923264	-2.173877	-0.292659
C	-3.752790	-3.066133	-1.936295
H	-2.816429	-3.625308	-2.004909
H	-4.580576	-3.773077	-2.069649
H	-3.783672	-2.351363	-2.766670
C	-5.663953	-1.468043	-0.143090
H	-5.792796	-0.894224	0.780582
H	-5.900998	-0.802828	-0.979970
H	-6.394396	-2.287122	-0.137949
C	-3.578724	-3.295049	1.176911
H	-4.308556	-4.112281	1.228500
H	-2.575408	-3.727103	1.112448
H	-3.642853	-2.727291	2.112778

TS1-t

0 3

C	0.328221	-2.651818	0.113871
C	0.328985	-3.822678	0.965725
C	0.952239	-3.464599	2.124634
C	1.351328	-2.081683	1.971669
H	-0.092462	-4.782651	0.694976
H	1.149349	-4.070456	3.000096
C	-0.219862	-2.61000	-1.161197
H	-0.667349	-3.52478	-1.53549
C	-0.273385	-1.49172	-1.983657
C	-0.924347	-1.45303	-3.277127
C	-0.769142	-0.18741	-3.757229
H	-1.434675	-2.29004	-3.73501
C	0.000875	0.540157	-2.768138
H	-1.114522	0.22647	-4.696271
C	0.480012	1.834366	-2.940847
H	0.197932	2.352036	-3.852725
C	1.331849	2.501532	-2.065606
C	1.871276	3.824465	-2.292192
C	2.644435	4.122828	-1.209008
H	1.676050	4.428706	-3.169240
C	2.571041	2.984124	-0.318965
H	3.212540	5.023518	-1.012978
N	0.272935	-0.270306	-1.694631
N	0.940056	-1.602404	0.752969
N	1.776689	2.008943	-0.865209
Fe	1.266223	0.241731	-0.00958
C	3.180239	2.919188	0.9299460
H	3.769352	3.777350	1.239605
C	3.090766	1.856303	1.8208360
C	3.735085	1.820417	3.1175850
C	3.434202	0.612545	3.6714290
H	4.338641	2.620240	3.528002
C	2.605761	-0.090054	2.714050
H	3.736523	0.215751	4.632430
N	2.403693	0.688110	1.6017080
C	2.112072	-1.375918	2.897084
H	2.364822	-1.880285	3.824797
C	3.930738	-1.363001	-0.330268
C	3.339950	-0.386412	-2.175242
C	4.892119	-1.694295	-1.252772
H	3.857838	-1.649982	0.706793
H	2.809240	0.194925	-2.912291
N	2.975941	-0.552697	-0.916212
N	4.494110	-1.060191	-2.420563
H	4.979078	-1.091559	-3.306482
C	6.124428	-2.529694	-1.157903
H	6.093208	-3.381054	-1.849004
H	7.027822	-1.950496	-1.385458
H	6.224397	-2.923724	-0.143196
N	-0.254762	1.01802	0.672275
C	-0.758622	2.332103	0.38684
H	-1.608210	2.59683	1.024908
H	0.025104	3.084835	0.537516
H	-1.101895	2.41325	-0.656641
C	-3.792974	1.144900	0.40543
C	-4.115982	1.476233	-0.929571
C	-5.012478	2.499474	-1.21857
C	-5.628664	3.213038	-0.18498
C	-5.336463	2.883865	1.14036
C	-4.432671	1.864003	1.43559
H	-3.636404	0.92502	-1.730864
H	-5.231987	2.74421	-2.254425
H	-6.332618	4.00883	-0.410455
H	-5.824324	3.41531	1.952962
H	-4.254288	1.60210	2.470481
C	-2.840462	0.055391	0.64357
C	-1.819085	0.007223	1.59189
H	-1.260509	-0.92197	1.594708
C	-1.867659	0.731920	2.92105
H	-2.129498	1.78550	2.778620
H	-2.691024	0.30115	3.510589
C	-0.575156	0.636470	3.73033
H	0.249945	1.109032	3.194548
H	-0.695527	1.12596	4.703711
H	-0.295617	-0.40732	3.904440
O	-2.830906	-0.90763	-0.34782
Si	-3.913017	-2.209160	-0.234033
C	-3.800387	-3.12933	-1.866379
H	-2.854238	-3.66650	-1.97400

H	-4.614670	-3.86043	-1.93927
H	-3.892853	-2.43491	-2.70865
C	-5.668797	-1.57286	0.011903
H	-5.764244	-0.99401	0.936623
H	-5.977294	-0.92514	-0.81576
H	-6.368423	-2.41640	0.066323
C	-3.416171	-3.28440	1.226009
H	-4.063348	-4.16655	1.303955
H	-2.378953	-3.62188	1.133114
H	-3.499731	-2.72255	2.163770

TS1$_{az}$-css

0 1

C	-0.067455	-2.27109	-1.139333
C	-0.288306	-3.67211	-0.849964
C	0.149612	-3.884406	0.423478
C	0.664749	-2.618944	0.901540
H	-0.723341	-4.38519	-1.53818
H	0.151697	-4.805475	0.992704
C	-0.435586	-1.64897	-2.325001
H	-0.934847	-2.25798	-3.07147
C	-0.234851	-0.30959	-2.631261
C	-0.649963	0.318685	-3.86857
C	-0.192695	1.602305	-3.82995
H	-1.205613	-0.17670	-4.65496
C	0.512293	1.754418	-2.574053
H	-0.292115	2.37764	-4.579204
C	1.237727	2.883217	-2.208212
H	1.229954	3.726434	-2.892170
C	1.992506	3.007557	-1.046395
C	2.761758	4.178965	-0.684102
C	3.321386	3.919466	0.5313720
H	2.848830	5.071504	-1.290928
C	2.892845	2.589898	0.9143430
H	3.962039	4.555701	1.129032
N	0.439461	0.591009	-1.846536
N	0.517371	-1.653279	-0.064032
N	2.097193	2.052727	-0.065963
Fe	1.174223	0.249664	-0.00352
C	3.227370	1.977328	2.1177300
H	3.854705	2.543392	2.800112
C	2.828929	0.709373	2.5271260
C	3.195828	0.102584	3.7928870
C	2.637124	-1.139686	3.806183
H	3.803274	0.578128	4.552749
C	1.933228	-1.290325	2.547921
H	2.690870	-1.896605	4.578631
N	2.059699	-0.155389	1.795345
C	1.275855	-2.444434	2.138778
H	1.283041	-3.293578	2.815532
C	3.676506	-1.589126	-0.518712
C	3.503770	-0.002888	-1.986806
C	4.728491	-1.764913	-1.383022
H	3.407184	-2.171937	0.347746
H	3.169962	0.859155	-2.542054
N	2.926051	-0.493668	-0.904536
N	4.597760	-0.741248	-2.310407
H	5.210304	-0.570993	-3.095774
C	5.826587	-2.773531	-1.431603
H	5.808768	-3.350768	-2.364334
H	6.814957	-2.304384	-1.350897
H	5.718648	-3.474941	-0.600122
N	-0.170552	0.99642	0.938042
C	-0.744829	2.245997	0.51270
H	-1.587974	2.55000	1.141972
H	0.040880	3.001443	0.642094
H	-1.060415	2.29489	-0.540844
C	-3.579530	1.129847	0.41740
C	-3.741815	1.521933	-0.92823
C	-4.575079	2.584434	-1.26681
C	-5.279797	3.271289	-0.27429
C	-5.140435	2.882968	1.06078
C	-4.298694	1.827244	1.40610
H	-3.194574	0.98754	-1.696941
H	-4.675656	2.87761	-2.308273
H	-5.935449	4.09593	-0.538526
H	-5.698061	3.39822	1.837931
H	-4.226556	1.52377	2.442806
C	-2.680183	0.004466	0.70771
C	-1.786549	-0.10930	1.754661
H	-1.231420	-1.04048	1.767916
C	-1.885546	0.608252	3.08007
H	-2.030649	1.68304	2.926904
H	-2.791167	0.25532	3.597137
C	-0.675697	0.375388	3.98551
H	0.230492	0.747692	3.507037
H	-0.810906	0.88465	4.946706
H	-0.533310	-0.69248	4.185016
O	-2.664107	-0.95243	-0.28072
Si	-3.860825	-2.158107	-0.314404
C	-3.728156	-2.96940	-2.001172
H	-2.819237	-3.56758	-2.09894
H	-4.588923	-3.62825	-2.16804
H	-3.724545	-2.21198	-2.793134
C	-5.572911	-1.396737	-0.120345
H	-5.685965	-0.87911	0.837810
H	-5.782164	-0.67182	-0.91408
H	-6.332597	-2.18716	-0.16936
C	-3.538231	-3.35657	1.096407
H	-4.289661	-4.15535	1.115595
H	-2.547491	-3.81139	1.000060
H	-3.578759	-2.83352	2.059340

Int3-t

0 3

C	-0.071906	-2.70576	0.809706
C	-0.119177	-4.119303	0.503502
C	-0.982330	-4.26557	-0.541802
C	-1.470045	-2.94272	-0.869376
H	0.441954	-4.882235	1.028073
H	-1.276868	-5.17420	-1.05150
C	0.714671	-2.138133	1.807033

H	1.323163	-2.807466	2.407051
C	0.799873	-0.777936	2.077956
C	1.635508	-0.192909	3.105045
C	1.434433	1.152721	3.0540490
H	2.288865	-0.754825	3.760198
C	0.468280	1.390868	2.0022350
H	1.888129	1.927645	3.658424
C	-0.032577	2.641462	1.67127
H	0.363358	3.492526	2.216085
C	-0.996648	2.903375	0.70509
C	-1.516303	4.221025	0.41090
C	-2.450552	4.066488	-0.57014
H	-1.195119	5.13167	0.900261
C	-2.499356	2.653639	-0.87845
H	-3.058060	4.82337	-1.050034
N	0.107498	0.203658	1.415916
N	-0.898827	-2.01127	-0.039762
N	-1.601270	1.96891	-0.095329
Fe	-1.186149	-0.00944	-0.12658
C	-3.338047	2.077002	-1.82725
H	-4.003266	2.73749	-2.374821
C	-3.370367	0.724033	-2.14681
C	-4.213063	0.138949	-3.16637
C	-3.932732	-1.19497	-3.188695
H	-4.917752	0.69120	-3.775254
C	-2.924555	-1.42668	-2.176161
H	-4.361279	-1.96434	-3.81851
N	-2.596907	-0.24655	-1.55824
C	-2.397520	-2.67625	-1.868551
H	-2.758076	-3.52132	-2.44718
C	-2.631196	-0.980057	2.465420
C	-3.902805	0.267377	1.22454
C	-3.829538	-0.89138	3.127583
H	-1.737373	-1.50946	2.752390
H	-4.279249	0.89158	0.430368
N	-2.690033	-0.25712	1.286948
N	-4.622569	-0.09142	2.317379
H	-5.576871	0.18176	2.506548
C	-4.303592	-1.46796	4.418980
H	-4.594093	-0.68583	5.131055
H	-5.169373	-2.12638	4.277138
H	-3.503480	-2.05733	4.874207
N	0.143990	0.214289	-1.403682
C	-0.147250	0.856489	-2.67242
H	-0.840811	1.68422	-2.520383
H	0.749939	1.267611	-3.147937
H	-0.616330	0.16929	-3.392893
C	2.739111	1.782061	-0.4066700
C	3.876668	2.260462	0.3134660
C	4.132776	3.616640	0.4464340
C	3.274775	4.569767	-0.121388
C	2.143597	4.123798	-0.816428
C	1.874038	2.769717	-0.956862
H	4.543109	1.538903	0.769821
H	5.011138	3.940168	1.0002360
H	3.477947	5.631421	-0.014650
H	1.444785	4.843633	-1.235693
H	0.961627	2.467471	-1.440744
C	2.515938	0.381201	-0.512681
C	1.451908	-0.417424	-1.258912
H	1.303177	-1.282244	-0.610197
C	2.016863	-1.004839	-2.580030
H	2.111382	-0.225339	-3.342083
H	3.036586	-1.353361	-2.391033
C	1.178281	-2.174616	-3.101471
H	0.145460	-1.874003	-3.295742
H	1.600141	-2.579015	-4.029074
H	1.141605	-2.982155	-2.361722
O	3.347078	-0.398352	0.234506
Si	4.452028	-1.649986	0.0856690
C	5.194579	-1.762633	1.806947
H	4.419020	-1.996908	2.545277
H	5.958717	-2.547343	1.857992
H	5.660331	-0.814580	2.099033
C	5.753498	-1.173991	-1.189096
H	5.316426	-1.036149	-2.184505
H	6.245527	-0.235843	-0.907577
H	6.523753	-1.951118	-1.266666
C	3.644995	-3.293562	-0.351363
H	4.357093	-4.108382	-0.168171
H	2.760817	-3.470993	0.268727
H	3.334207	-3.352421	-1.398541

Int3-css

0 1

C	-0.093376	-2.79307	0.427011
C	-0.140949	-4.14659	-0.090302
C	-0.953521	-4.11623	-1.185557
C	-1.409659	-2.74831	-1.327631
H	0.383973	-4.991840	0.337498
H	-1.234104	-4.93277	-1.83930
C	0.649862	-2.391728	1.536826
H	1.214686	-3.158169	2.059801
C	0.724741	-1.095079	2.032326
C	1.489159	-0.702127	3.199592
C	1.284213	0.633486	3.3696410
H	2.092561	-1.371757	3.798568
C	0.386069	1.056038	2.3136800
H	1.683890	1.284967	4.136882
C	-0.119781	2.344046	2.189588
H	0.218761	3.079821	2.913290
C	-1.027200	2.773222	1.22665
C	-1.544205	4.123755	1.13005
C	-2.396684	4.141547	0.06626
H	-1.280704	4.93502	1.797321
C	-2.399259	2.801125	-0.48604
H	-2.980164	4.96937	-0.317399
N	0.075053	-0.007311	1.500782
N	-0.869639	-1.96323	-0.34073
N	-1.547147	1.99169	0.226847
Fe	-1.166592	0.02192	-0.083839
C	-3.172789	2.392937	-1.56869

H	-3.802953	3.14131	-2.040660
C	-3.197414	1.107627	-2.10458
C	-3.985350	0.713079	-3.25583
C	-3.722099	-0.60579	-3.474526
H	-4.643471	1.37128	-3.809619
C	-2.779925	-1.01684	-2.451672
H	-4.121433	-1.25540	-4.24364
N	-2.473116	0.041611	-1.63485
C	-2.284568	-2.30973	-2.316040
H	-2.624303	-3.04724	-3.03789
C	-2.747685	-1.35481	2.184370
C	-3.920299	0.145840	1.14665
C	-3.978404	-1.35676	2.792830
H	-1.881544	-1.95711	2.406613
H	-4.238546	0.92153	0.468921
N	-2.724688	-0.41872	1.165392
N	-4.708964	-0.39284	2.115110
H	-5.666489	-0.13317	2.304808
C	-4.533508	-2.15438	3.924752
H	-4.844187	-1.51523	4.760594
H	-5.405250	-2.74583	3.618232
H	-3.772263	-2.84582	4.295666
N	0.329284	0.430737	-1.364047
C	0.065482	1.258052	-2.527067
H	-0.648538	2.04123	-2.269534
H	0.964017	1.753736	-2.919542
H	-0.376327	0.68330	-3.359522
C	2.749015	1.834873	-0.267425
C	4.054751	2.262893	0.0865680
C	4.359709	3.613997	0.1805510
C	3.371628	4.579389	-0.050526
C	2.071937	4.171649	-0.358637
C	1.754818	2.819576	-0.443020
H	4.829067	1.522554	0.247737
H	5.371059	3.919195	0.433809
H	3.609780	5.635606	0.036892
H	1.281672	4.906312	-0.484628
H	0.735936	2.499981	-0.575573
C	2.546167	0.411394	-0.4566250
C	1.566649	-0.287188	-1.380854
H	1.383477	-1.232099	-0.858874
C	2.193042	-0.664826	-2.742677
H	2.315758	0.229085	-3.361713
H	3.207025	-1.045788	-2.569351
C	1.363983	-1.721497	-3.478368
H	0.348376	-1.365856	-3.670238
H	1.824168	-1.983570	-4.438065
H	1.275957	-2.634006	-2.878212
O	3.277185	-0.339759	0.352216
Si	4.249008	-1.755311	0.2375640
C	4.599291	-2.180681	2.025383
H	3.720269	-2.615871	2.509714
H	5.414673	-2.911770	2.082247
H	4.896029	-1.292168	2.592344
C	5.794826	-1.186118	-0.665207
H	5.548711	-0.765982	-1.647251
H	6.333640	-0.421250	-0.095130
H	6.475371	-2.031273	-0.823826
C	3.463619	-3.205496	-0.653907
H	4.069209	-4.094681	-0.433918
H	2.444027	-3.402625	-0.316195
H	3.446789	-3.077626	-1.739445

Int3-oss

0 1

C	-0.024610	-2.68981	0.820623
C	-0.061927	-4.10497	0.520461
C	-0.944671	-4.26462	-0.506504
C	-1.453573	-2.94848	-0.828588
H	0.517888	-4.859778	1.036390
H	-1.239412	-5.17840	-1.00682
C	0.772806	-2.109136	1.802259
H	1.400712	-2.768895	2.392803
C	0.844628	-0.747789	2.068751
C	1.688435	-0.150776	3.081952
C	1.469814	1.192107	3.0320770
H	2.358993	-0.703543	3.727416
C	0.485579	1.416746	1.9941340
H	1.923134	1.973669	3.628135
C	-0.031985	2.661572	1.66684
H	0.363451	3.518065	2.203462
C	-1.010122	2.910561	0.71197
C	-1.545794	4.222209	0.41836
C	-2.493414	4.053975	-0.54717
H	-1.225624	5.13832	0.898103
C	-2.534427	2.638649	-0.84676
H	-3.115300	4.80262	-1.021547
N	0.129850	0.224393	1.415474
N	-0.876347	-2.00797	-0.01368
N	-1.617674	1.96635	-0.074601
Fe	-1.184709	-0.00897	-0.10213
C	-3.385045	2.049405	-1.77682
H	-4.065532	2.70101	-2.316204
C	-3.412097	0.694031	-2.08749
C	-4.268792	0.096048	-3.08792
C	-3.977519	-1.23553	-3.107981
H	-4.989334	0.63885	-3.686665
C	-2.948428	-1.45269	-2.113356
H	-4.410996	-2.01211	-3.725478
N	-2.619785	-0.26623	-1.50802
C	-2.403581	-2.69556	-1.810118
H	-2.766869	-3.54674	-2.37803
C	-2.581274	-0.99684	2.508450
C	-3.876481	0.257154	1.29900
C	-3.766841	-0.91242	3.193736
H	-1.681795	-1.52707	2.775636
H	-4.267930	0.88574	0.515596
N	-2.662646	-0.26727	1.335426
N	-4.575348	-0.10823	2.403244
H	-5.526057	0.16318	2.611937
C	-4.216033	-1.49638	4.490740
H	-4.493754	-0.71834	5.212338

H	-5.083764	-2.15487	4.361679
H	-3.406981	-2.08742	4.927640
N	0.119946	0.214011	-1.407977
C	-0.203384	0.852996	-2.67082
H	-0.891329	1.68261	-2.503890
H	0.681680	1.260127	-3.171574
H	-0.692426	0.16405	-3.376141
C	2.715260	1.793094	-0.450504
C	3.851680	2.275424	0.2685430
C	4.104251	3.632723	0.4003130
C	3.244061	4.582752	-0.168340
C	2.113714	4.132732	-0.8628470
C	1.846834	2.778118	-1.0000450
H	4.520377	1.556259	0.725136
H	4.981682	3.958904	0.954048
H	3.444010	5.645108	-0.062469
H	1.412894	4.850365	-1.282580
H	0.933685	2.473812	-1.481255
C	2.503569	0.390397	-0.563424
C	1.434979	-0.412778	-1.296421
H	1.301314	-1.280035	-0.647393
C	1.977797	-0.994125	-2.628032
H	2.055605	-0.212549	-3.389987
H	3.002567	-1.338079	-2.457691
C	1.135290	-2.166510	-3.137484
H	0.097062	-1.870797	-3.309444
H	1.540082	-2.564969	-4.075186
H	1.117925	-2.977192	-2.400378
O	3.340238	-0.383513	0.184997
Si	4.455538	-1.623724	0.0143370
C	5.219358	-1.740611	1.726027
H	4.454764	-1.986217	2.472098
H	5.990836	-2.518888	1.763212
H	5.680292	-0.790310	2.018475
C	5.735933	-1.125670	-1.273195
H	5.283802	-0.982485	-2.261122
H	6.223860	-0.185911	-0.989946
H	6.511440	-1.895530	-1.368542
C	3.659586	-3.272143	-0.424955
H	4.381312	-4.081382	-0.255220
H	2.783881	-3.462338	0.203276
H	3.338033	-3.326760	-1.469122

Int3-q

0 5

C	-0.304493	-2.69939	0.866099
C	-0.419110	-4.075028	0.531069
C	-1.175410	-4.13463	-0.634613
C	-1.518920	-2.79471	-0.960698
H	0.020359	-4.892604	1.088340
H	-1.485680	-5.01148	-1.188629
C	0.455331	-2.173267	1.945996
H	0.931241	-2.893341	2.605306
C	0.714361	-0.841220	2.173011
C	1.623300	-0.331602	3.184419
C	1.679272	1.015184	3.0255260
H	2.159205	-0.950184	3.892767
C	0.770746	1.352299	1.9446670
H	2.263553	1.737877	3.580157
C	0.449001	2.638264	1.5611670
H	0.995048	3.449053	2.032776
C	-0.589512	3.001802	0.67151
C	-1.037217	4.330909	0.43215
C	-2.150645	4.232475	-0.38698
H	-0.581148	5.22307	0.841986
C	-2.346572	2.846759	-0.64230
H	-2.764635	5.03103	-0.784002
N	0.206964	0.207596	1.427340
N	-0.972125	-1.92357	-0.04745
N	-1.385285	2.09821	0.007694
Fe	-1.126196	0.10296	-0.076075
C	-3.332239	2.304379	-1.49733
H	-4.031956	3.00705	-1.940882
C	-3.428646	0.982248	-1.89411
C	-4.320983	0.484508	-2.92278
C	-4.008787	-0.82686	-3.114005
H	-5.067829	1.08062	-3.431723
C	-2.948838	-1.14797	-2.176613
H	-4.454238	-1.53192	-3.80435
N	-2.613004	-0.03090	-1.44986
C	-2.407585	-2.40977	-1.994299
H	-2.765459	-3.20007	-2.64784
C	-3.559121	-1.08085	1.430774
C	-2.771341	0.704826	2.38595
C	-4.347133	-0.86607	2.533765
H	-3.617706	-1.86629	0.694395
H	-2.178733	1.57552	2.616637
N	-2.588699	-0.09988	1.353764
N	-3.825476	0.27519	3.125197
H	-4.170689	0.71675	3.965897
C	-5.519277	-1.60626	3.084189
H	-5.327329	-1.97295	4.099908
H	-6.416382	-0.97635	3.121846
H	-5.739810	-2.46917	2.450600
N	0.150839	0.271886	-1.423121
C	-0.194064	0.981880	-2.63985
H	-0.668800	1.93734	-2.398286
H	0.673278	1.190834	-3.272026
H	-0.912842	0.41638	-3.249897
C	2.924260	1.560794	-0.447512
C	3.981409	1.936091	0.4359620
C	4.384861	3.256519	0.5592140
C	3.765643	4.270439	-0.186904
C	2.729700	3.923105	-1.063509
C	2.314319	2.605370	-1.196384
H	4.453359	1.166810	1.034437
H	5.190452	3.505220	1.246251
H	4.083298	5.304203	-0.086810
H	2.230673	4.694231	-1.645346
H	1.499770	2.381279	-1.864643
C	2.517617	0.201509	-0.522192
C	1.420437	-0.452207	-1.355994

H	1.199652	-1.366223	-0.797213
C	1.980711	-0.927038	-2.720247
H	2.199499	-0.074302	-3.371488
H	2.950452	-1.398763	-2.527992
C	1.069292	-1.936355	-3.423131
H	0.094986	-1.503810	-3.665491
H	1.525253	-2.294674	-4.353339
H	0.884314	-2.800822	-2.775476
O	3.154678	-0.656588	0.322047
Si	4.129473	-2.018379	0.1738370
C	4.670028	-2.350771	1.940152
H	3.803015	-2.583277	2.568433
H	5.361429	-3.200396	1.987862
H	5.172528	-1.477294	2.370615
C	5.589958	-1.584523	-0.931792
H	5.258376	-1.298685	-1.936783
H	6.155811	-0.742994	-0.516000
H	6.272387	-2.436933	-1.034676
C	3.202266	-3.512196	-0.495476
H	3.809370	-4.413768	-0.341681
H	2.248200	-3.654150	0.022514
H	2.990582	-3.435230	-1.566422

TS2-t

-1 3

C	0.099018	-2.566483	1.115720
C	0.289048	-3.992266	0.954041
C	-0.471057	-4.37172	-0.112324
C	-1.141360	-3.18142	-0.592068
H	0.932937	-4.601451	1.575490
H	-0.585933	-5.35910	-0.54161
C	0.743244	-1.787875	2.070388
H	1.409099	-2.295506	2.761153
C	0.660009	-0.404246	2.165174
C	1.411615	0.401783	3.1048370
C	1.091247	1.700117	2.8511010
H	2.105692	0.006477	3.835253
C	0.128061	1.688513	1.7701330
H	1.461602	2.595130	3.334330
C	-0.510794	2.816691	1.27668
H	-0.208826	3.77697	1.682097
C	-1.535580	2.816240	0.33611
C	-2.263061	3.994438	-0.08100
C	-3.226415	3.574969	-0.95120
H	-2.052943	5.00006	0.260759
C	-3.078251	2.141220	-1.07610
H	-3.970551	4.16559	-1.470861
N	-0.107042	0.39884	1.362845
N	-0.770470	-2.09658	0.162105
N	-2.041002	1.70545	-0.287853
Fe	-1.340733	-0.18001	-0.12548
C	-3.854575	1.333391	-1.90060
H	-4.644831	1.81526	-2.468691
C	-3.661479	-0.02817	-2.104182
C	-4.404328	-0.83013	-3.050951
C	-3.873783	-2.08607	-3.003841
H	-5.215896	-0.46323	-3.66697
C	-2.817484	-2.05522	-2.015383
H	-4.163656	-2.96303	-3.56914
N	-2.704815	-0.79488	-1.48545
C	-2.073143	-3.16413	-1.622783
H	-2.271955	-4.10303	-2.13067
C	-3.088223	0.324343	2.40252
C	-3.570856	-1.55422	1.428106
C	-4.084152	-0.24376	3.157706
H	-2.571341	1.25943	2.550336
H	-3.582487	-2.39154	0.748612
N	-2.781999	-0.49902	1.335805
N	-4.375257	-1.43954	2.517288
H	-5.065974	-2.11579	2.810886
C	-4.780775	0.207596	4.39715
H	-5.859744	0.32633	4.238201
H	-4.642736	-0.50127	5.222932
H	-4.378988	1.17387	4.713038
N	-0.062196	0.09707	-1.438495
C	-0.466029	0.537392	-2.76062
H	-1.255508	1.28711	-2.675842
H	0.357660	0.987371	-3.324390
H	-0.869027	-0.28689	-3.36815
C	2.306331	2.113171	-0.6701290
C	3.354098	2.825652	-0.008401
C	3.446476	4.207053	-0.074159
C	2.503992	4.959249	-0.792715
C	1.458009	4.283192	-1.434345
C	1.353412	2.900130	-1.377146
H	4.081375	2.259100	0.559985
H	4.262406	4.710442	0.440128
H	2.578919	6.041978	-0.841866
H	0.697557	4.845584	-1.971793
H	0.502999	2.423470	-1.833641
C	2.253847	0.694922	-0.560187
C	1.328729	-0.313189	-1.242247
H	1.302369	-1.133126	-0.522278
C	2.001653	-0.909183	-2.505385
H	2.005817	-0.182601	-3.324501
H	3.053087	-1.090246	-2.267451
C	1.360380	-2.226700	-2.949511
H	0.302202	-2.102396	-3.194440
H	1.872044	-2.635613	-3.828965
H	1.418304	-2.969362	-2.145822
O	3.122796	0.133651	0.303715
Si	4.479321	-0.919525	0.2790270
C	5.169064	-0.696707	2.006764
H	4.425222	-0.949501	2.772776
H	6.020986	-1.389470	2.043747
H	5.512400	0.329696	2.187219
C	5.622947	-0.339592	-1.092004
H	5.164402	-0.389260	-2.086749
H	5.965382	0.689249	-0.924405
H	6.470664	-1.037760	-1.015256
C	3.888787	-2.689550	0.039096
H	4.762315	-3.321183	0.225303

H	3.090671	-2.942219	0.745304
H	3.518304	-2.890060	-0.971483
O	7.273741	-2.454486	0.491945
H	7.529991	-1.534696	0.675249

Int4-t

-1 3

C	-1.268092	2.279381	-1.84253
C	-2.042916	3.501696	-1.95227
C	-2.717405	3.652643	-0.78081
C	-2.362646	2.524074	0.06513
H	-2.054022	4.14410	-2.824986
H	-3.395332	4.44666	-0.490355
C	-0.443039	1.787614	-2.83083
H	-0.372070	2.35860	-3.752758
C	0.330704	0.614024	-2.745521
C	1.181571	0.114361	-3.7744810
C	1.732709	-1.062316	-3.298515
H	1.341242	0.592125	-4.733919
C	1.205140	-1.262184	-1.985653
H	2.422172	-1.732775	-3.797854
C	1.448191	-2.397322	-1.185134
H	2.122796	-3.148475	-1.585715
C	0.896737	-2.636083	0.057878
C	1.155441	-3.824847	0.852984
C	0.450588	-3.694143	2.009726
H	1.798635	-4.640142	0.543453
C	-0.253860	-2.42760	1.938527
H	0.390881	-4.383246	2.844032
N	0.364403	-0.224642	-1.649752
N	-1.478402	1.70727	-0.600587
N	0.048485	-1.795434	0.744835
Fe	-0.624977	0.01727	0.121127
C	-1.102007	-1.94954	2.913522
H	-1.235654	-2.56017	3.802771
C	-1.803818	-0.72927	2.865380
C	-2.660799	-0.23552	3.891521
C	-3.143756	0.986299	3.45311
H	-2.873598	-0.74796	4.822579
C	-2.571632	1.214670	2.16390
H	-3.830333	1.65800	3.955091
N	-1.750024	0.16732	1.817048
C	-2.860544	2.322163	1.33683
H	-3.547945	3.06619	1.729935
C	-2.170422	-2.11043	-1.565052
C	-3.502316	-0.71300	-0.572216
C	-3.441522	-2.49144	-1.917229
H	-1.223762	-2.54523	-1.84529
H	-3.885797	0.10158	0.022001
N	-2.226019	-1.00917	-0.73274
N	-4.274082	-1.58598	-1.27387
H	-5.283118	-1.57498	-1.316288
C	-3.950915	-3.59745	-2.779121
H	-4.599545	-4.28513	-2.22180
H	-4.526332	-3.21894	-3.63343
H	-3.108931	-4.17412	-3.17131
N	0.787499	0.942028	0.892521
C	1.071707	0.846291	2.3105790
H	0.827396	-0.154165	2.670416
H	2.134639	1.022414	2.528660
H	0.486915	1.561016	2.912672
C	3.775989	0.477133	0.0564160
C	5.153896	0.605077	-0.200766
C	6.053036	-0.358897	0.242763
C	5.585887	-1.484169	0.933614
C	4.219927	-1.630276	1.174357
C	3.318871	-0.654015	0.745428
H	5.497035	1.474913	-0.750797
H	7.115959	-0.240186	0.049527
H	6.286010	-2.242735	1.274329
H	3.840118	-2.511169	1.6844230
H	2.261327	-0.777237	0.910294
C	2.883079	1.561612	-0.463566
C	1.519104	1.951167	0.1553410
H	0.928827	2.212166	-0.721969
C	1.742681	3.287168	0.9178000
H	2.317655	3.109305	1.834676
H	2.360786	3.928437	0.279746
C	0.430308	3.998936	1.2466030
H	-0.237383	3.35922	1.828130
H	0.617831	4.918659	1.815336
H	-0.104986	4.26486	0.329193
O	3.289462	2.258106	-1.393567

Int4-css

-1 1

C	-1.320079	2.335473	-1.75262
C	-2.075523	3.570261	-1.83032
C	-2.712930	3.713099	-0.63248
C	-2.343515	2.565993	0.17248
H	-2.102243	4.22634	-2.692176
H	-3.370436	4.51102	-0.308388
C	-0.535300	1.824620	-2.78422
H	-0.482210	2.40536	-3.701322
C	0.194992	0.641924	-2.738819
C	1.014247	0.141153	-3.8264290
C	1.554905	-1.033505	-3.398334
H	1.147615	0.638745	-4.779461
C	1.063038	-1.251456	-2.050641
H	2.222331	-1.702702	-3.927982
C	1.344386	-2.378560	-1.286947
H	2.004603	-3.122446	-1.724565
C	0.850798	-2.638804	-0.011592
C	1.149629	-3.829801	0.758662
C	0.481492	-3.707065	1.942241
H	1.785869	-4.640043	0.423214
C	-0.222963	-2.44067	1.890446
H	0.455703	-4.396843	2.777439
N	0.247328	-0.215455	-1.668426
N	-1.492071	1.74757	-0.525103
N	0.025115	-1.806470	0.699620
Fe	-0.684548	0.00252	0.109037

C	-1.041003	-1.94812	2.903909
H	-1.150364	-2.56063	3.795210
C	-1.721635	-0.73450	2.880839
C	-2.555374	-0.24517	3.962000
C	-3.039749	0.965427	3.56464
H	-2.735656	-0.77263	4.891196
C	-2.502888	1.212343	2.24007
H	-3.701069	1.63701	4.099188
N	-1.697749	0.17112	1.849822
C	-2.804115	2.328617	1.464892
H	-3.470337	3.06974	1.898854
C	-2.327489	-2.08962	-1.502188
C	-3.593958	-0.63625	-0.513086
C	-3.615660	-2.43591	-1.829752
H	-1.401476	-2.56045	-1.79353
H	-3.944963	0.19764	0.074162
N	-2.329006	-0.97328	-0.68640
N	-4.408219	-1.49543	-1.18822
H	-5.416836	-1.45296	-1.21543
C	-4.174056	-3.53682	-2.667765
H	-4.835383	-4.19616	-2.091211
H	-4.751193	-3.15223	-3.518386
H	-3.357683	-4.14592	-3.06530
N	0.957811	0.902749	0.8728350
C	1.206678	0.900176	2.2874220
H	0.874208	-0.050499	2.717822
H	2.283081	0.988099	2.540013
H	0.697274	1.698594	2.876333
C	3.877765	0.402747	-0.002222
C	5.256237	0.510597	-0.261220
C	6.145918	-0.458132	0.195951
C	5.667478	-1.567368	0.903205
C	4.298850	-1.691749	1.150050
C	3.407026	-0.714558	0.704218
H	5.610601	1.367607	-0.825086
H	7.210114	-0.355280	-0.001586
H	6.359290	-2.330710	1.251317
H	3.912638	-2.560066	1.677906
H	2.340973	-0.787335	0.863719
C	2.991486	1.493340	-0.532615
C	1.651151	1.913337	0.1265320
H	1.052157	2.165656	-0.753320
C	1.911317	3.280647	0.8269300
H	2.483082	3.125752	1.751047
H	2.541562	3.890291	0.166765
C	0.617872	4.035204	1.1377360
H	-0.038817	3.44357	1.778993
H	0.826169	4.990266	1.639046
H	0.061551	4.246090	0.217858
O	3.371432	2.102841	-1.537746

1a-trans

0 1

C	-1.436311	-0.236323	-0.278162
C	-1.510403	-1.44343	0.436396
C	-2.740851	-2.03873	0.703900
C	-3.922086	-1.44792	0.247452
C	-3.860101	-0.26166	-0.485425
C	-2.628067	0.336212	-0.75036
H	-0.595763	-1.91038	0.785197
H	-2.778874	-2.96699	1.267163
H	-4.881313	-1.91468	0.452225
H	-4.770793	0.19377	-0.864097
H	-2.585872	1.23800	-1.351644
C	-0.117406	0.384514	-0.544183
C	0.157950	1.701397	-0.529658
H	1.170495	1.990732	-0.805840
C	-0.735404	2.831718	-0.09829
H	-1.062635	3.41642	-0.971608
H	-1.645216	2.44641	0.371905
C	-0.011553	3.770910	0.879135
H	0.897670	4.185246	0.427409
H	-0.655243	4.60841	1.170310
H	0.282687	3.233736	1.788099
O	0.858910	-0.548483	-0.832432
Si	2.338736	-0.709258	-0.020191
C	2.858489	-2.475927	-0.376680
H	2.958405	-2.647158	-1.454597
H	3.825663	-2.698334	0.089492
H	2.123401	-3.189674	0.011797
C	2.060368	-0.416122	1.815260
H	1.639287	0.578980	1.994077
H	1.369825	-1.154724	2.237744
H	3.006889	-0.486568	2.364145
C	3.619255	0.492271	-0.696226
H	4.612575	0.245738	-0.300773
H	3.672174	0.438460	-1.789732
H	3.399772	1.528756	-0.417991

1a-trans+·

1 2

C	1.561835	-0.252937	0.129854
C	1.805165	-1.503910	-0.482242
C	3.103441	-1.968802	-0.620520
C	4.173833	-1.212229	-0.125914
C	3.942719	0.011959	0.510812
C	2.648114	0.496325	0.635598
H	0.968525	-2.078948	-0.860854
H	3.289468	-2.918140	-1.111334
H	5.189101	-1.581344	-0.231466
H	4.772777	0.581437	0.914673
H	2.475743	1.423994	1.166733
C	0.190926	0.201605	0.270523
C	-0.202673	1.562637	0.41081
H	-1.218978	1.72313	0.760122
C	0.561011	2.780440	0.049563
H	0.867912	3.284351	0.981957
H	1.471404	2.535924	-0.500889
C	-0.313762	3.761756	-0.75982
H	-1.212690	4.04298	-0.202269
H	0.257511	4.669435	-0.971455
H	-0.619006	3.31651	-1.711314

O -0.721329 -0.73096 0.25592
Si -2.477390 -0.758156 0.036287
C -2.764682 -2.56508 -0.30920
H -2.425614 -3.18418 0.52803
H -3.833365 -2.75586 -0.45969
H -2.233910 -2.88431 -1.21238
C -2.849947 0.348594 -1.42446
H -2.698590 1.41106 -1.209294
H -2.233828 0.07874 -2.289041
H -3.900142 0.21716 -1.711313
C -3.230750 -0.18922 1.64945
H -4.313559 -0.35952 1.61650
H -2.828460 -0.76253 2.49143
H -3.071585 0.87523 1.849701

Int2-· doublet

-1 2
C 2.873144 -0.274076 -1.133175
C 3.908508 -1.253456 -1.297736
C 3.315145 -2.485725 -1.130631
C 1.930199 -2.240870 -0.863003
H 4.946968 -1.027847 -1.50926
H 3.773742 -3.466460 -1.17778
C 3.063207 1.115371 -1.199855
H 4.068367 1.470641 -1.409655
C 2.079431 2.064701 -1.003049
C 2.311927 3.4985070-1.063338
C 1.116699 4.1036500-0.836062
H 3.275034 3.955108 -1.258521
C 0.139658 3.049617 -0.633461
H 0.890433 5.163019 -0.805373
C -1.198374 3.273979 -0.387427
H -1.531207 4.307806 -0.34462
C -2.167668 2.282552 -0.173676
C -3.545510 2.530944 0.120432
C -4.141773 1.300403 0.297666
H -3.997398 3.513658 0.187003
C -3.115602 0.317294 0.107499
H -5.174298 1.079902 0.541283
N 0.752366 1.813999 -0.729806
N 1.673002 -0.888546 -0.87032
N -1.919122 0.927777 -0.18536
Fe -0.139829 0.017380 -0.59685
C -3.287173 -1.069610 0.250217
H -4.281640 -1.425905 0.50415
C -2.291717 -2.013246 0.095199
C -2.500166 -3.441639 0.251710
C -1.307596 -4.046861 0.008013
H -3.447751 -3.896121 0.51559
C -0.353032 -2.999622 -0.29681
H -1.069448 -5.103805 0.02972
N -0.975662 -1.763200 -0.23944
C 0.972939 -3.229445 -0.594336
H 1.309030 -4.262941 -0.60839
C 1.532491 -0.001476 2.115905
C -0.592600 0.215394 2.480509

C 1.395229 0.072745 3.480475
H 2.428778 -0.110093 1.525163
H -1.658896 0.305360 2.345527
N 0.293734 0.088410 1.510964
N 0.030658 0.209475 3.690996
H -0.425467 0.290775 4.588675
C 2.390984 0.031724 4.590750
H 2.375009 0.951437 5.189294
H 2.210124 -0.809199 5.272275
H 3.396815 -0.082022 4.177582
N -0.506911 0.003667 -2.37831
C -1.469614 -0.897497 -2.94170
H -1.594075 -0.725581 -4.02536
H -1.185203 -1.955695 -2.80360

Int2-· quartet

-1 4
C 2.613745 -1.274294 -1.111457
C 3.263767 -2.541691 -1.241203
C 2.299109 -3.508187 -1.020767
C 1.080430 -2.817124 -0.757091
H 4.314031 -2.678459 -1.470518
H 2.410891 -4.586097 -1.032897
C 3.250439 -0.023645 -1.255633
H 4.310097 -0.031229 -1.494886
C 2.633972 1.205422 -1.119802
C 3.314200 2.481286 -1.276083
C 2.388363 3.455343 -1.068691
H 4.365378 2.589237 -1.516343
C 1.131153 2.792223-0.7803890
H 2.520895 4.530449 -1.101797
C -0.050293 3.446356 -0.515244
H -0.032771 4.53311 -0.522557
C -1.284141 2.832174 -0.229659
C -2.491502 3.524006 0.073940
C -3.454914 2.559210 0.316111
H -2.596342 4.60218 0.105212
C -2.817265 1.291600 0.152768
H -4.495959 2.69908 0.582566
N 1.310255 1.420345 -0.812714
N 1.284048 -1.451366 -0.814249
N -1.494199 1.46676 -0.180234
Fe -0.136740 0.00732 -0.595634
C -3.446250 0.039635 0.326748
H -4.499373 0.04631 0.592992
C -2.832095 -1.189224 0.176639
C -3.502494 -2.465800 0.355612
C -2.579297 -3.438385 0.124686
H -4.546509 -2.57628 0.624127
C -1.331012 -2.775448 -0.193302
H -2.707941 -4.51366 0.165439
N -1.513147 -1.40327 -0.153750
C -0.151372 -3.430109 -0.469954
H -0.170565 -4.51685 -0.458987
C 1.583600 -0.037893 2.059073
C -0.539852 0.060311 2.489962

C	1.482019	-0.015040	3.428208
H	2.466095	-0.084568	1.439633
H	-1.613193	0.10361	2.385829
N	0.324048	0.009227	1.494065
N	0.118309	0.047412	3.680708
H	-0.314734	0.07910	4.592762
C	2.510269	-0.045277	4.508716
H	2.467651	0.851544	5.139760
H	2.389747	-0.917009	5.164330
H	3.507893	-0.095457	4.064253
N	-0.627422	0.00016	-2.286536
C	-1.928492	-0.161754	-2.848153
H	-1.903335	-0.02355	-3.941358
H	-2.332841	-1.16792	-2.644074
H	-2.649647	0.55895	-2.428254

(H) Molecular Docking Studies

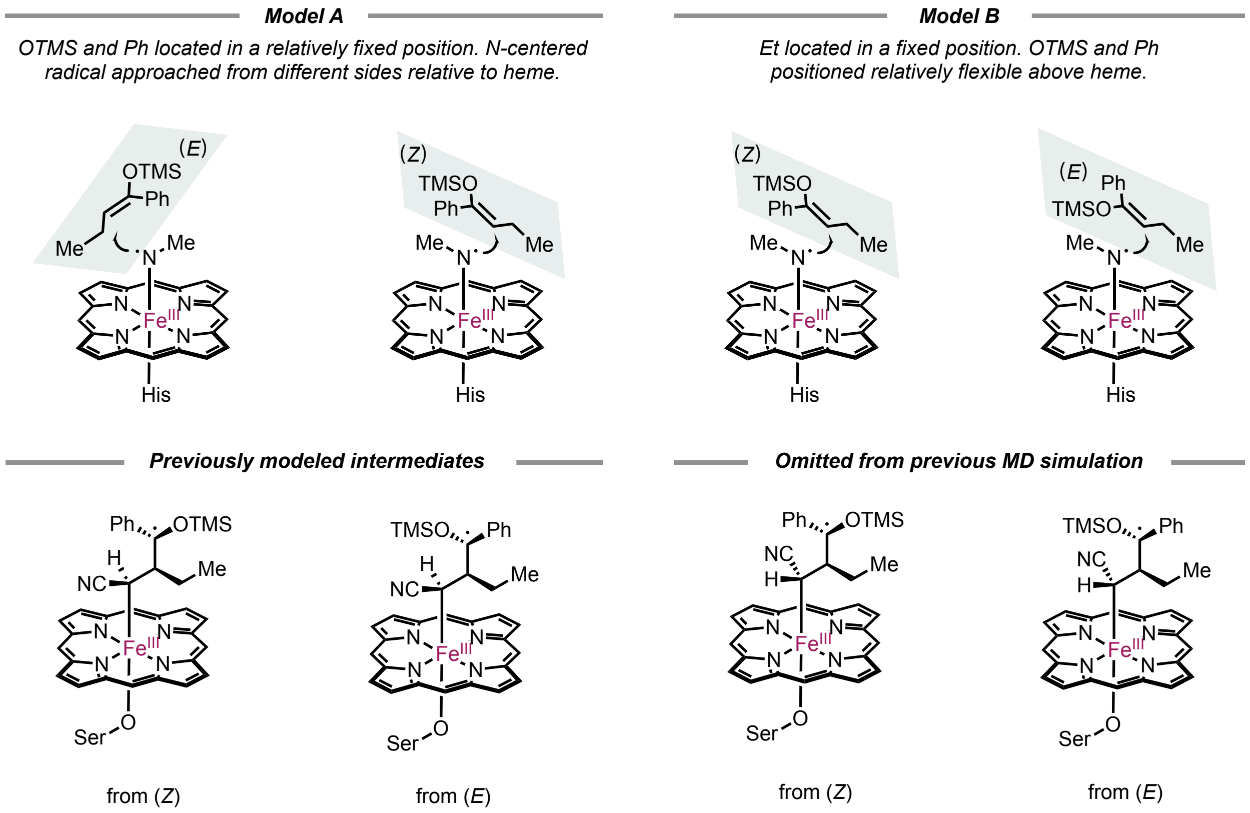

Figure S7. Two possible mechanistic models that explain the stereoconvergency of the transformation, relative to previous literature reports. Model A: The *N*-centered radical approaches to the silyl enol ether from different directions; Model B: The –Et group is held at a fixed position inside the active site.

The docking studies were performed using the Glide implemented in Maestro software version 14.6 from Schrodinger Inc[35,36]. Protein structures were predicted using Chai-1[37], and ligand structures were obtained from the DFT-optimized structures described above.

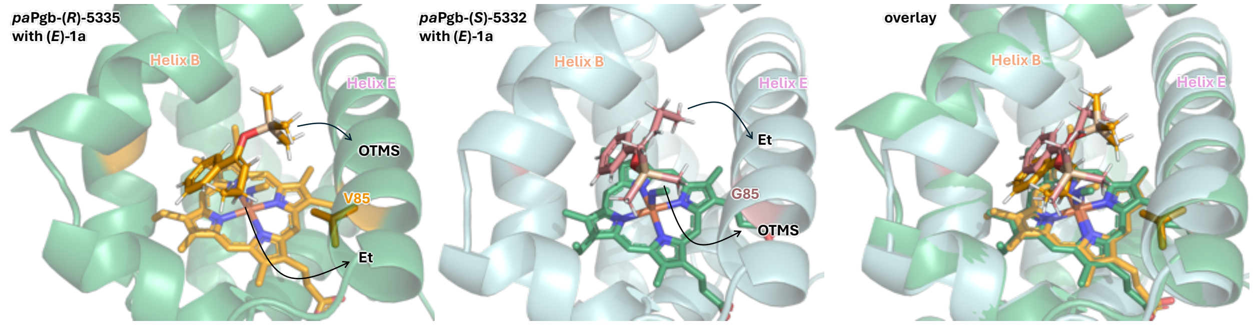

Figure S8. Molecular docking results for ***pa*Pgb-AKS-(*S*)-5332** and ***pa*Pgb-AKS-(*R*)-5335** with (*E*)-**1a**.

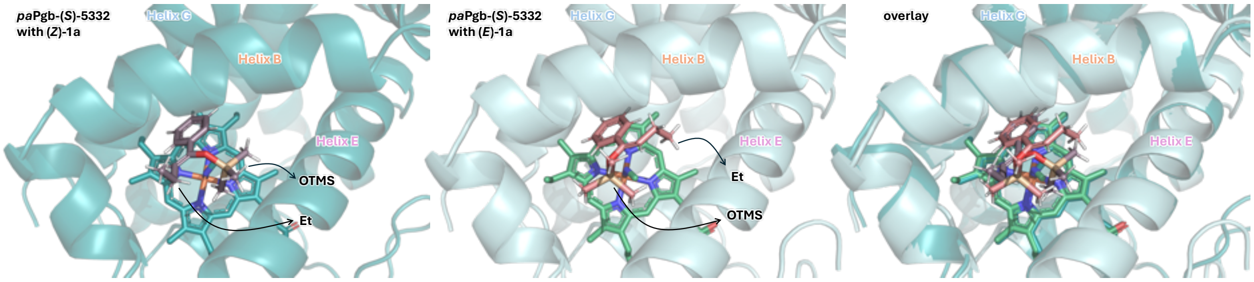

Figure S9. Molecular docking results for ***pa*Pgb-AKS-(*S*)-5332** with (*Z*)-**1a** and (*E*)-**1a**.

Molecular docking studies suggest different binding poses for the *E* and *Z* isomers in the active site of the engineered protoglobins. As shown in the figures above (based on a structure of ***pa*Pgb-AKS-(*S*)-G3-5332** predicted by Chai), both *E* and *Z* isomers anchor the phenyl group between B/G helixes while positioning the –OTMS group at the hydrophobic substrate tunnel; the C=C geometry flips but exposes the same *Si* face to the heme-nitrenoid species, thus affording the single (*S*)-**3a** product.

XI. NMR Spectra

NMR spectrums for standard products:

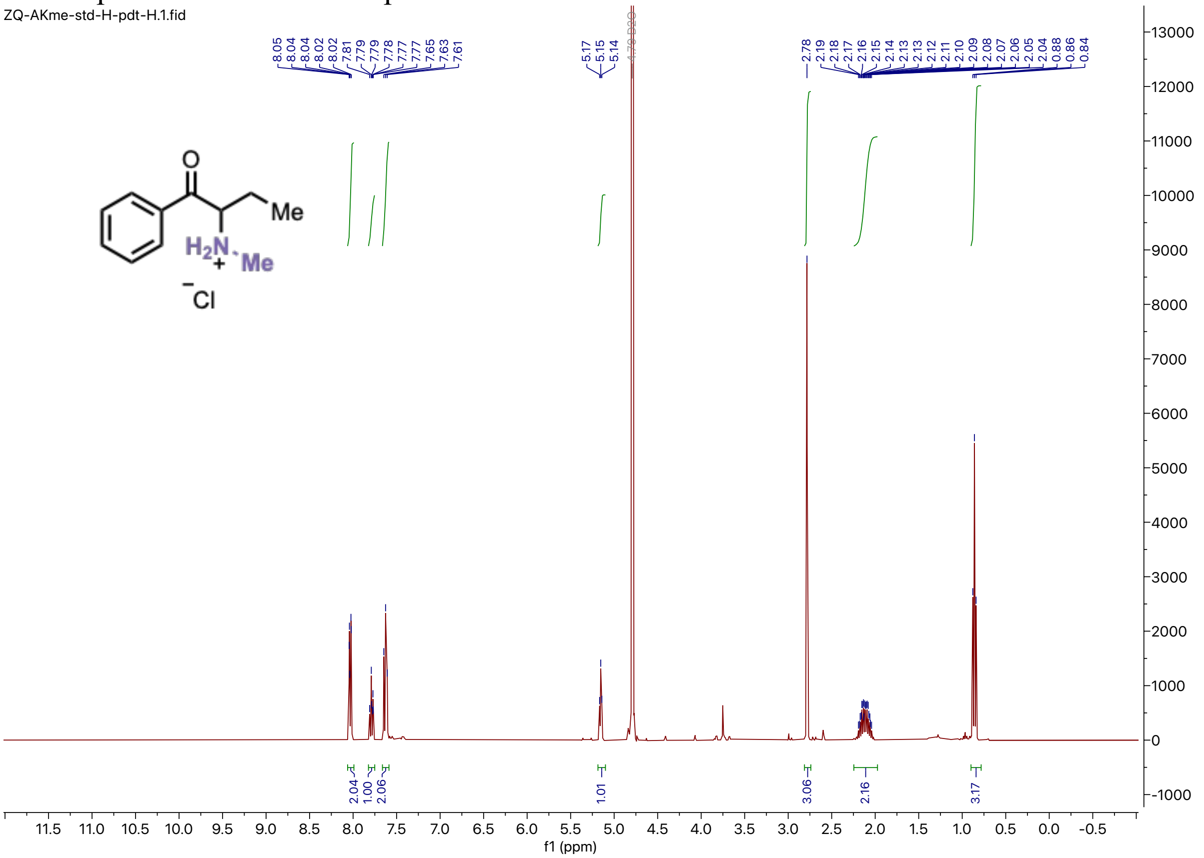

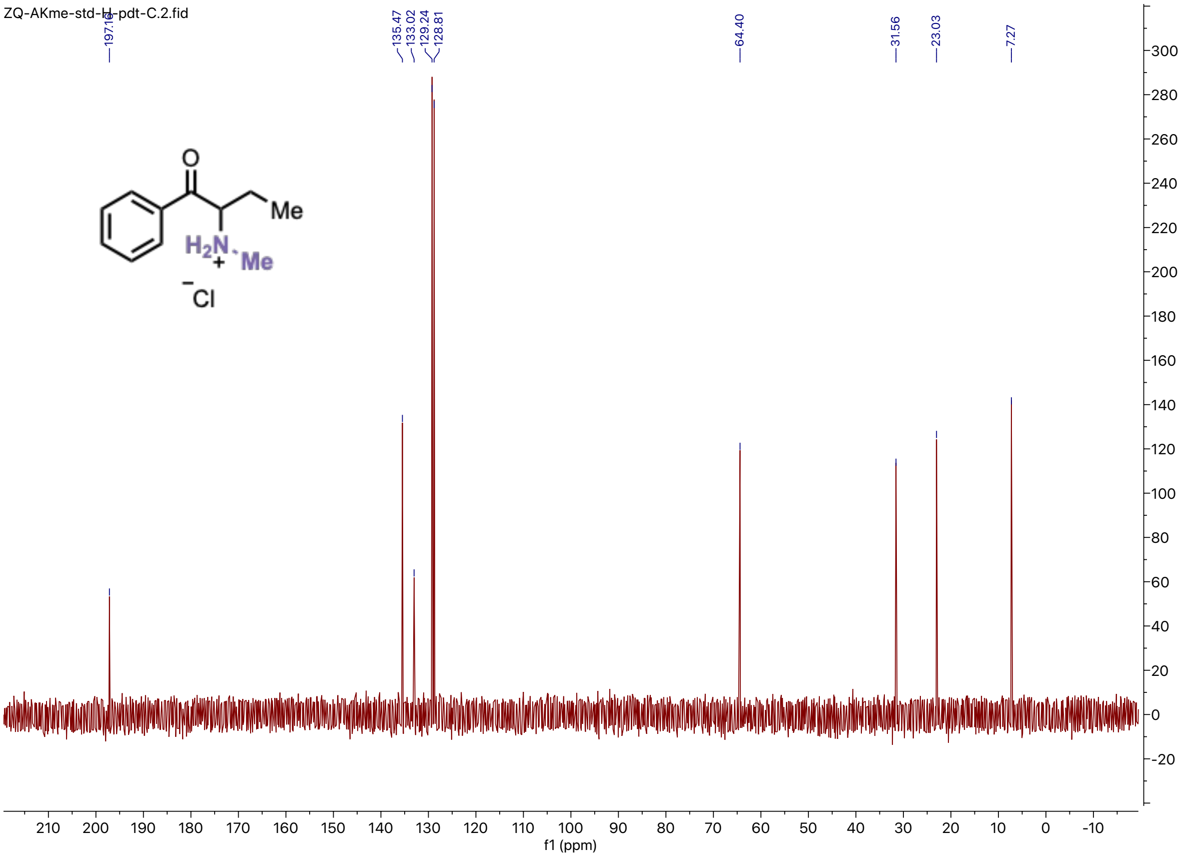

ZQ-AKme-std-H-pdt-C.2.fid
197.16
135.47
133.02
129.24
128.81
64.40
31.56
23.03
7.27
O
Me
H2N
+
Me
Cl
f1 (ppm)

ZQ-AKme-radical-clock-H-w-IS.1.fid

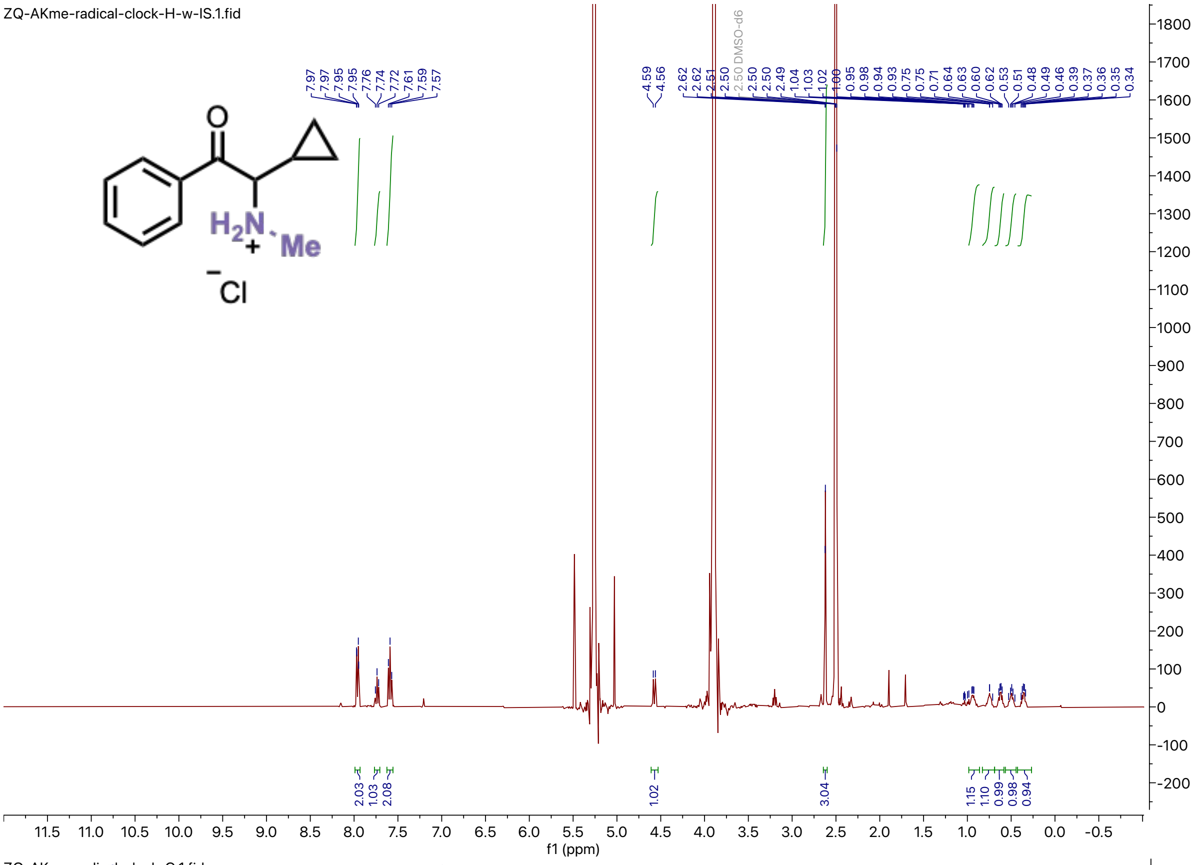

ZQ-AKme-radical-clock-C.1.fid

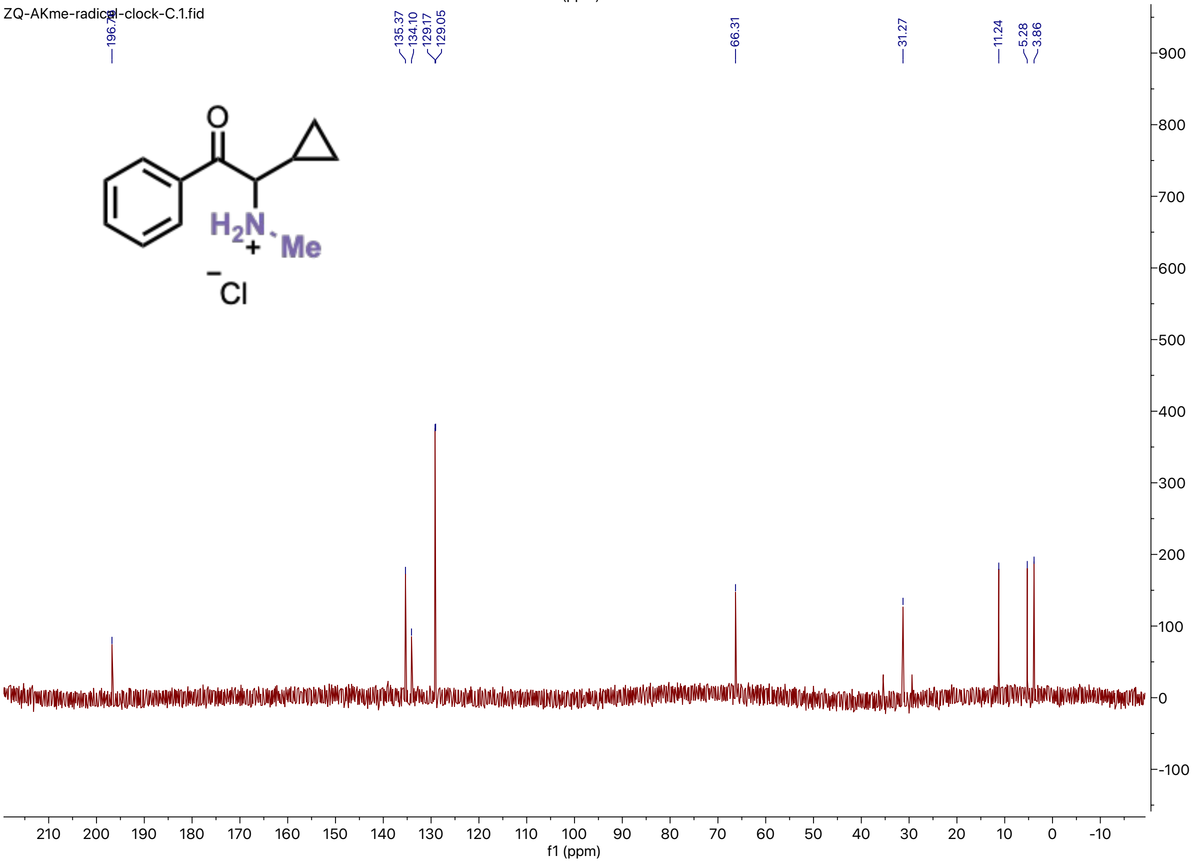

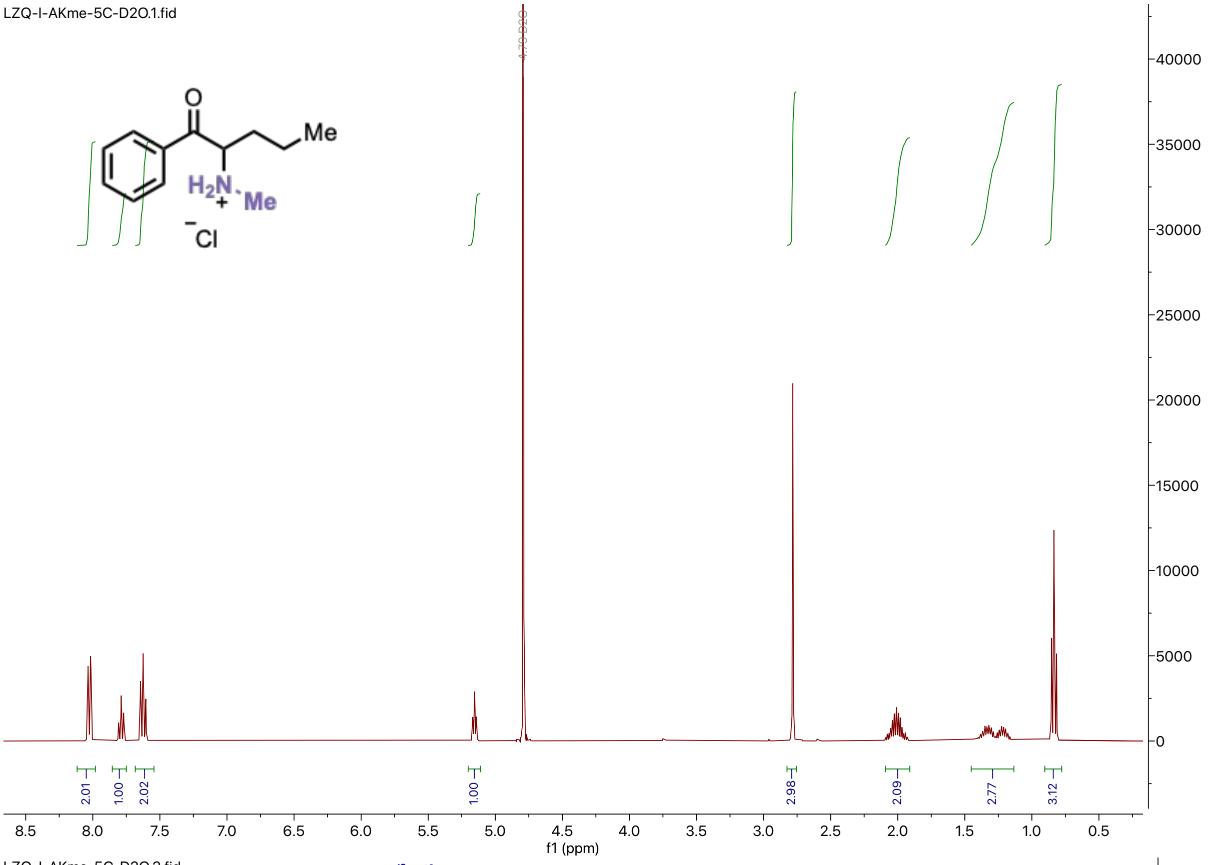
LZQ-I-AKme-5C-D2O.1.fid
4.79 D2O
H2N Me
Cl
2.01
1.00
2.02
1.00
2.98
2.09
2.77
3.12
f1 (ppm)

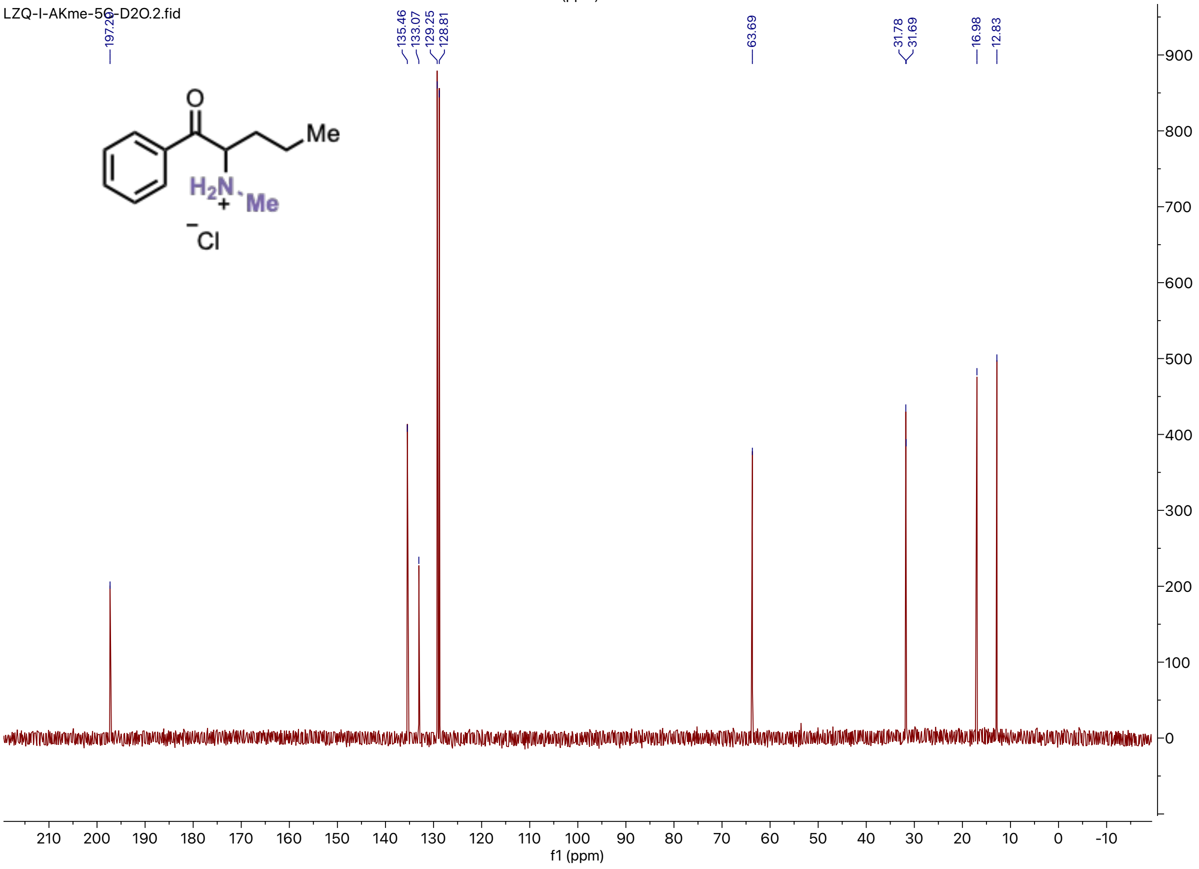
LZQ-I-AKme-5C-D2O.2.fid
197.29
135.46
133.07
129.25
128.81
63.69
31.78
31.69
16.98
12.83
H2N Me
Cl
f1 (ppm)

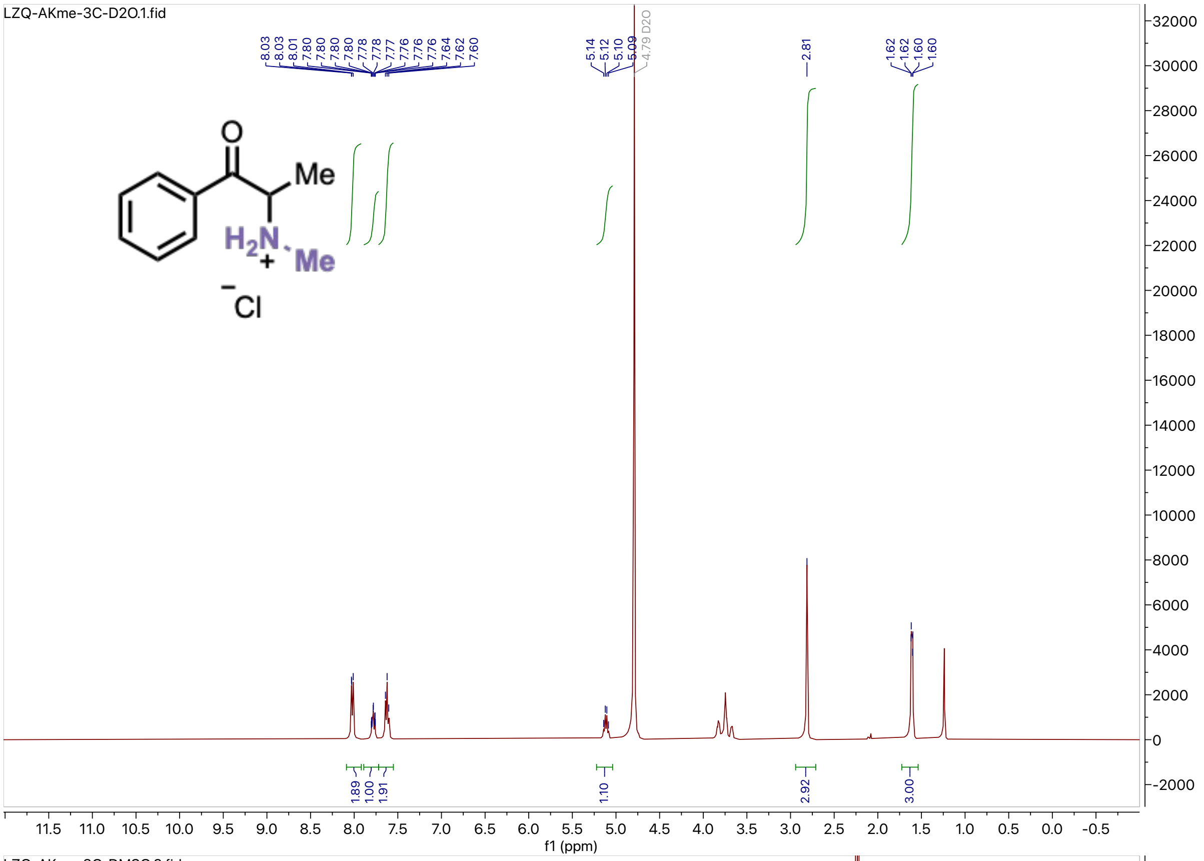
LZQ-AKme-3C-D2O.1.fid
8.03
8.03
8.01
7.80
7.80
7.80
7.80
7.78
7.78
7.77
7.76
7.76
7.76
7.64
7.62
7.60
5.14
5.12
5.10
5.09
4.79 D2O
2.81
1.62
1.62
1.60
1.60
O
Me
H_2N
Me
Cl
1.89
1.00
1.91
1.10
2.92
3.00
f1 (ppm)

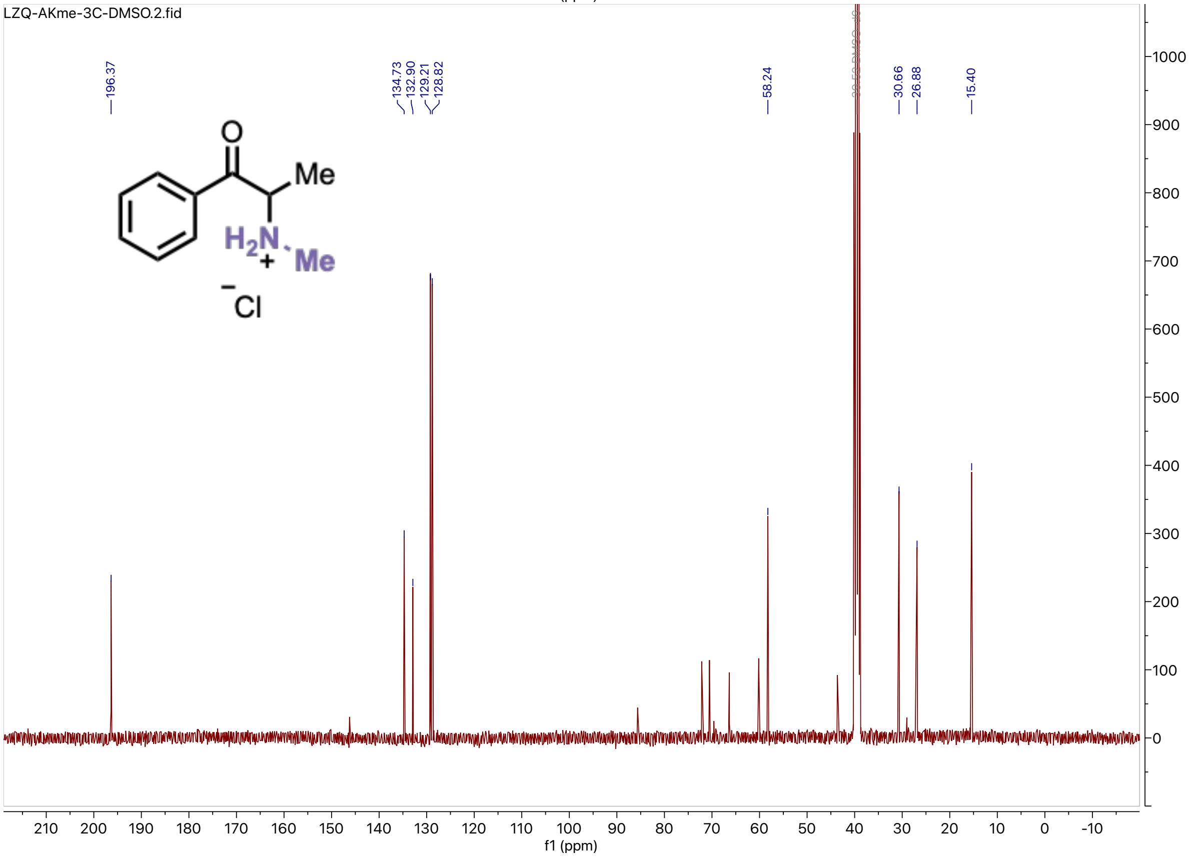
LZQ-AKme-3C-DMSO.2.fid
196.37
134.73
132.90
129.21
128.82
58.24
30.66
26.88
15.40
O
Me
H_2N
Me
Cl
f1 (ppm)

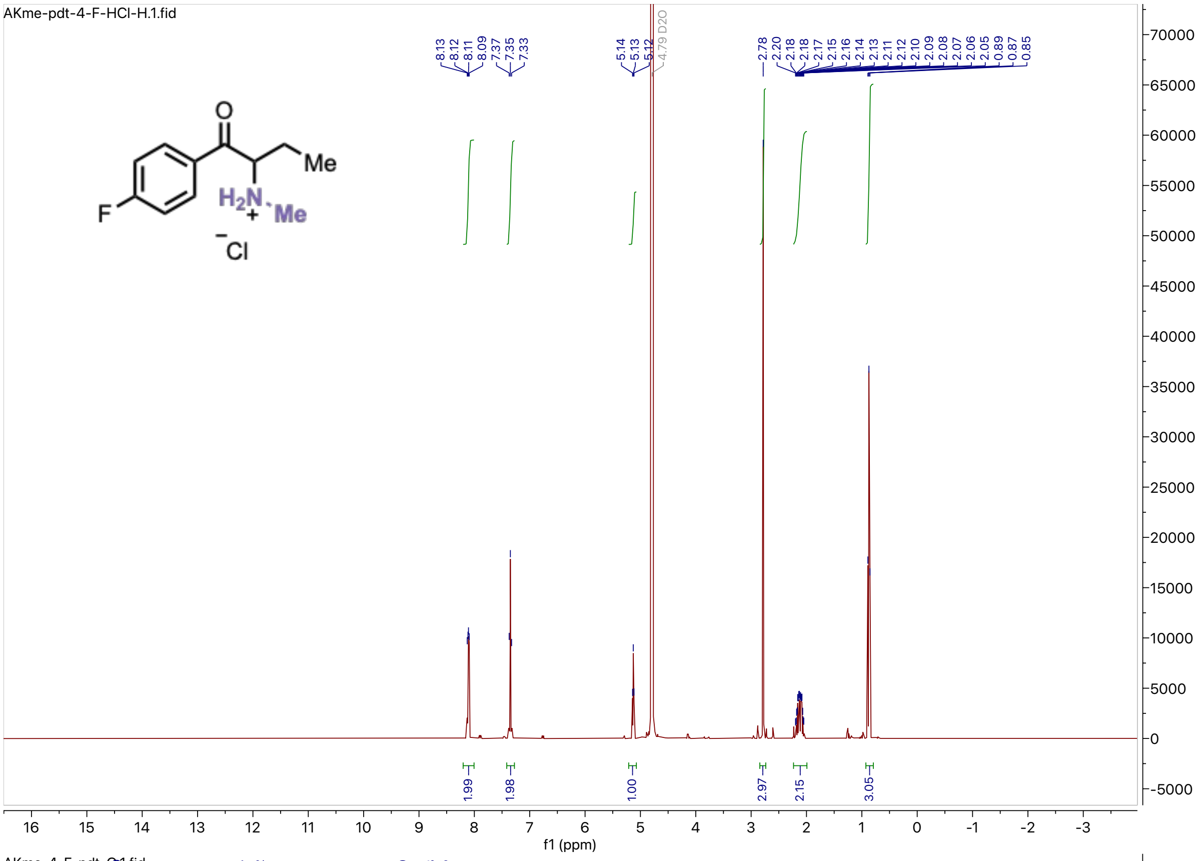

AKme-pdt-4-F-HCl-H.1.fid
8.13
8.12
8.11
8.09
7.37
7.35
7.33
5.14
5.13
5.12
4.79 D2O
2.78
2.20
2.18
2.18
2.17
2.15
2.16
2.14
2.13
2.11
2.12
2.10
2.09
2.08
2.07
2.06
2.05
0.89
0.87
0.85
O
Me
F
H_2N^+
Me
Cl^-
1.99
1.98
1.00
2.97
2.15
3.05
f1 (ppm)

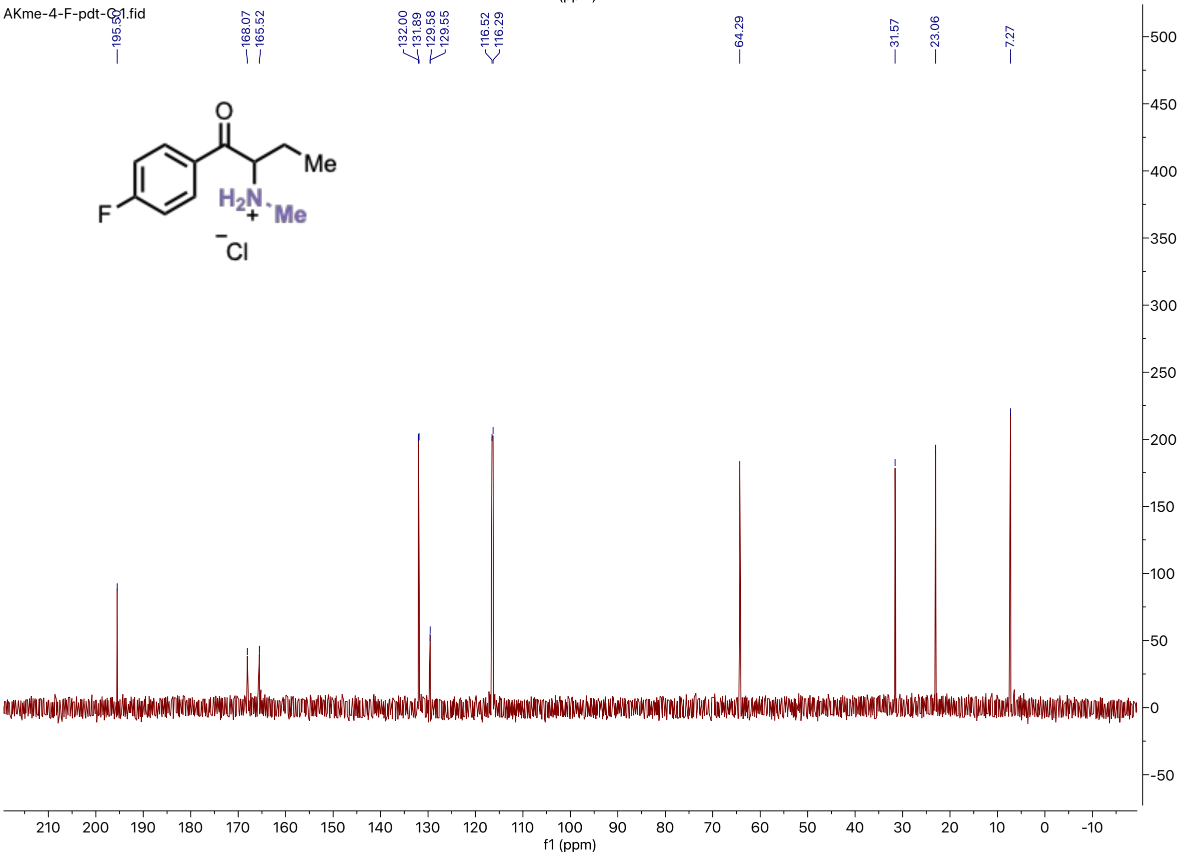

AKme-4-F-pdt-C.1.fid
195.53
168.07
165.52
132.00
131.89
129.58
129.55
116.52
116.29
64.29
31.57
23.06
7.27
O
Me
F
H_2N^+
Me
Cl^-
f1 (ppm)

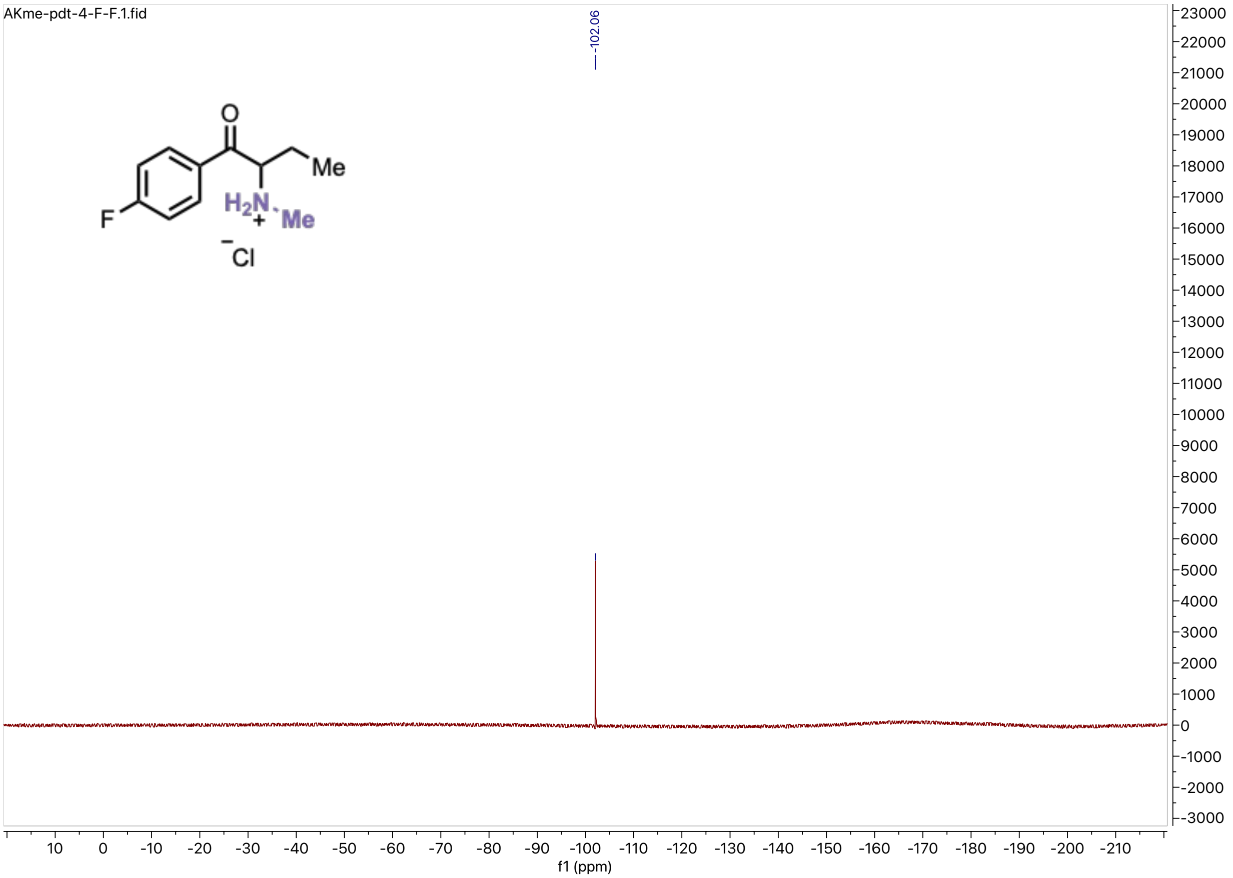

AKme-pdt-4-F-F.1.fid
-102.06
O
Me
F
H2N
Me
Cl
23000
22000
21000
20000
19000
18000
17000
16000
15000
14000
13000
12000
11000
10000
9000
8000
7000
6000
5000
4000
3000
2000
1000
0
-1000
-2000
-3000
10 0 -10 -20 -30 -40 -50 -60 -70 -80 -90 -100 -110 -120 -130 -140 -150 -160 -170 -180 -190 -200 -210
f1 (ppm)

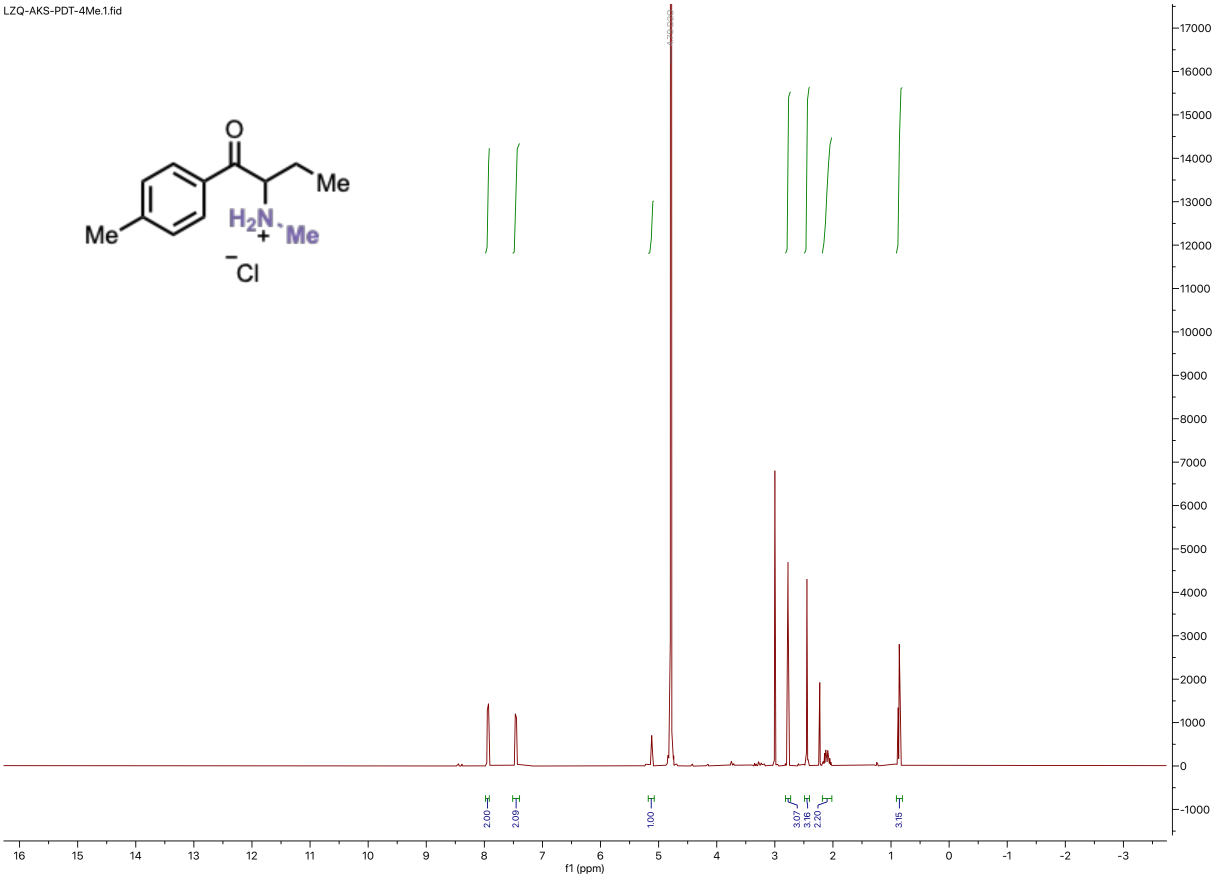
LZQ-AKS-PDT-4Me.1.fid
f1 (ppm)
2.00
2.09
1.00
3.07
3.16
2.20
3.15

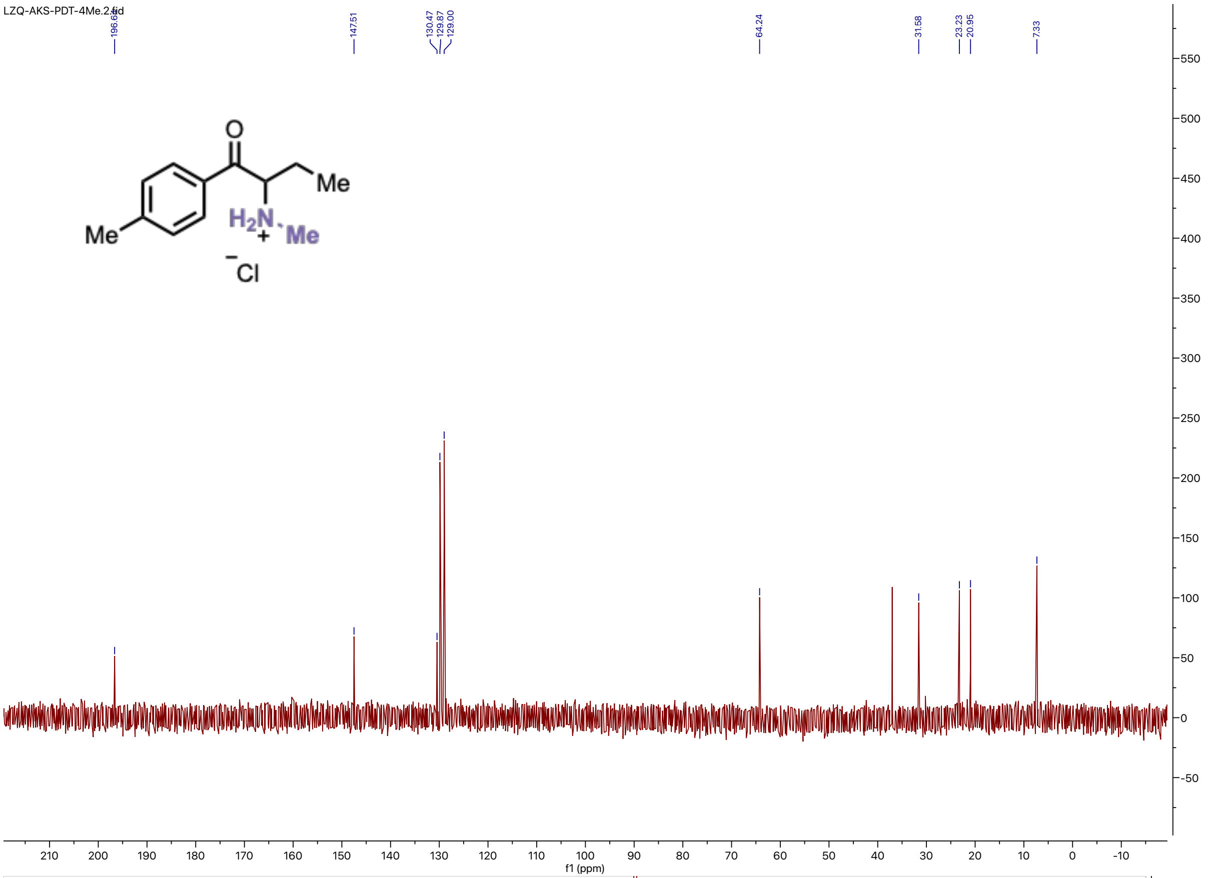
LZQ-AKS-PDT-4Me.2.fid
f1 (ppm)

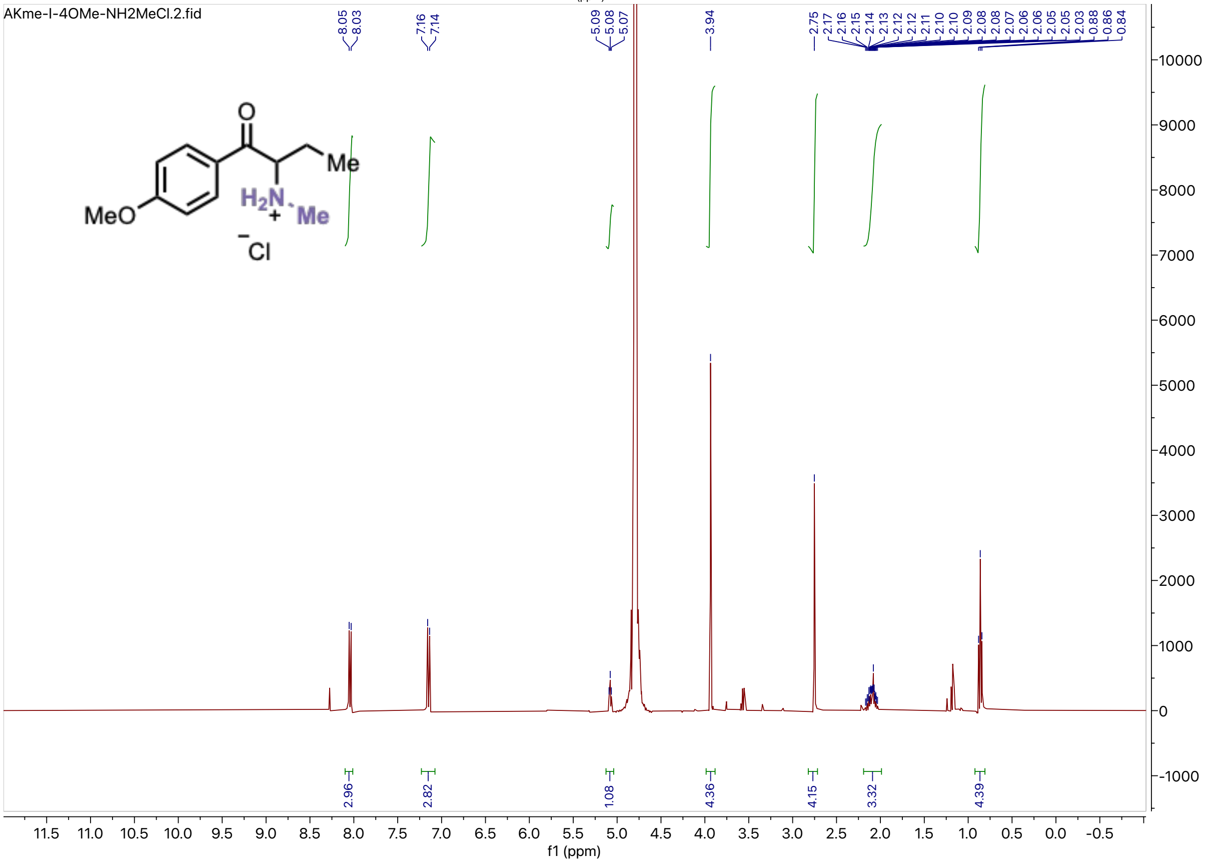
AKme-I-4OMe-NH2MeCl.2.fid
f1 (ppm)

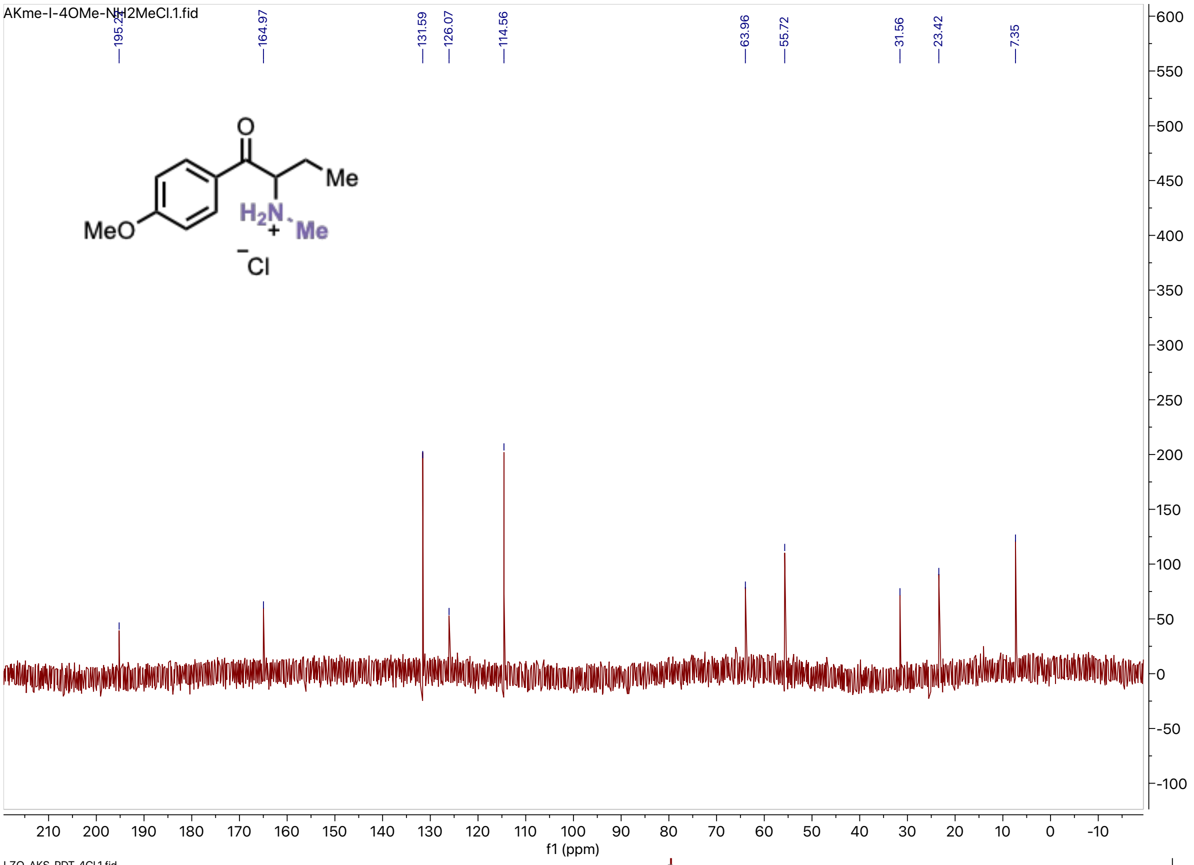

AKme-I-4OMe-NH2MeCl.1.fid
195.27
164.97
131.59
126.07
114.56
63.96
55.72
31.56
23.42
7.35
MeO
O
Me
H2N
Me
Cl
600
550
500
450
400
350
300
250
200
150
100
50
0
-50
-100
210
200
190
180
170
160
150
140
130
120
110
100
90
80
70
60
50
40
30
20
10
0
-10
f1 (ppm)

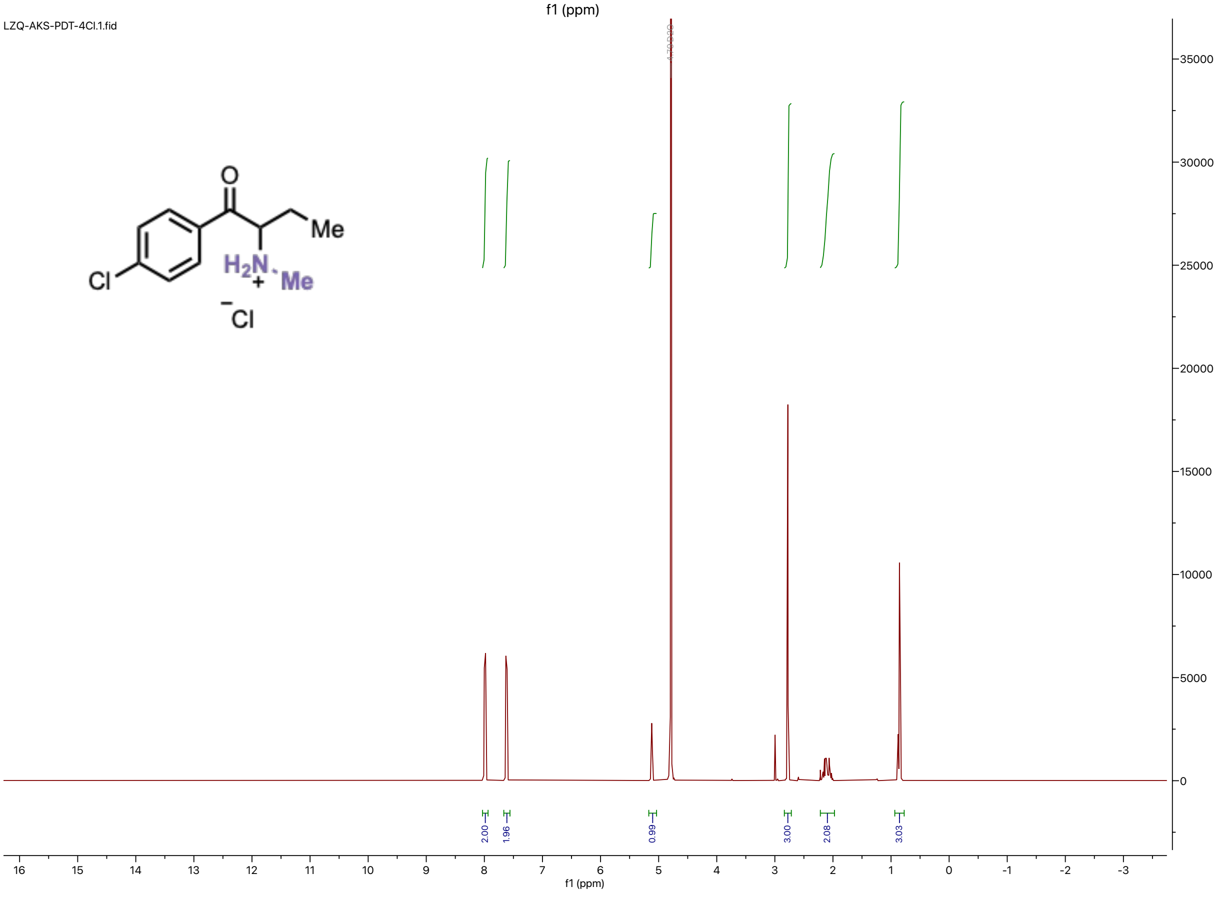

LZQ-AKS-PDT-4Cl.1.fid
4.79 D2O
Cl
O
Me
H2N
Me
Cl
35000
30000
25000
20000
15000
10000
5000
0
2.00
1.96
0.99
3.00
2.08
3.03
16
15
14
13
12
11
10
9
8
7
6
5
4
3
2
1
0
-1
-2
-3
f1 (ppm)

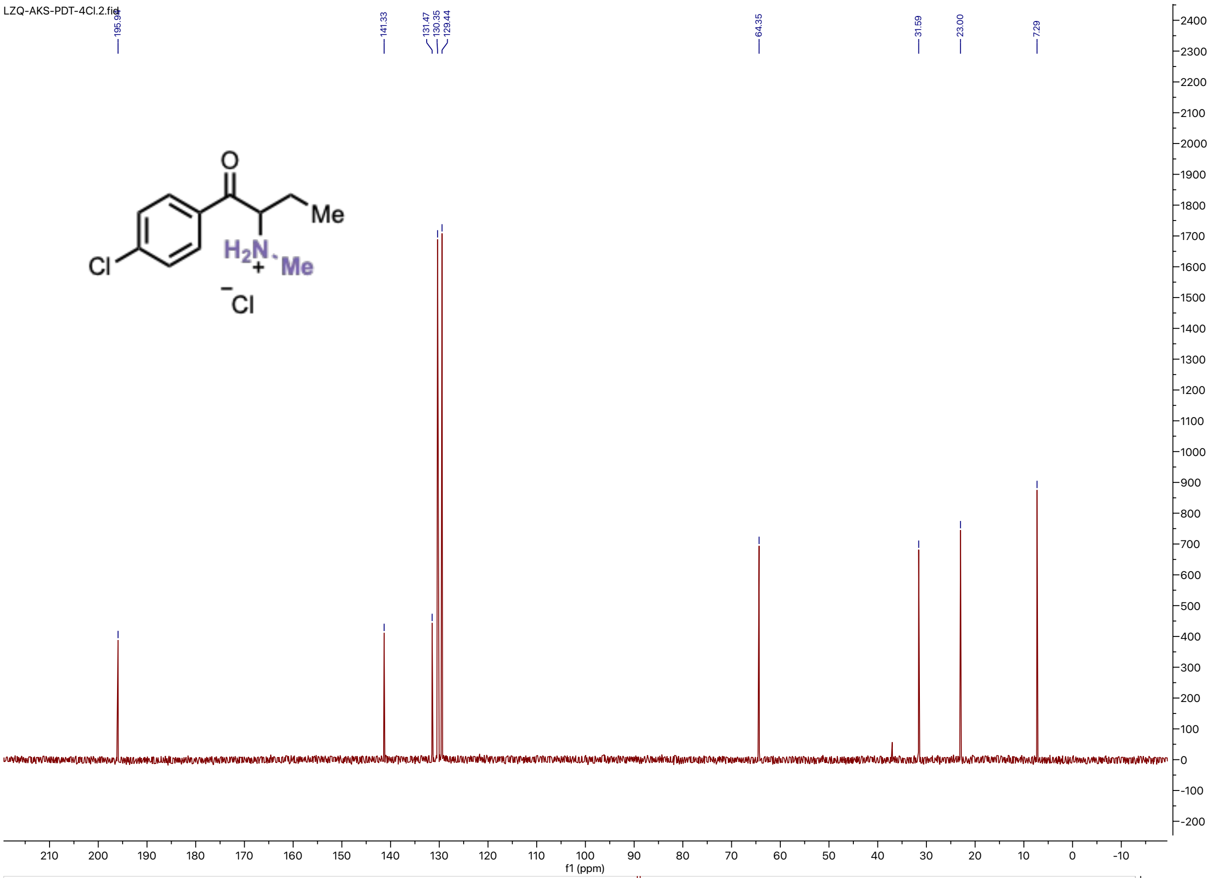
LZQ-AKS-PDT-4Cl.2.fid
f1 (ppm)

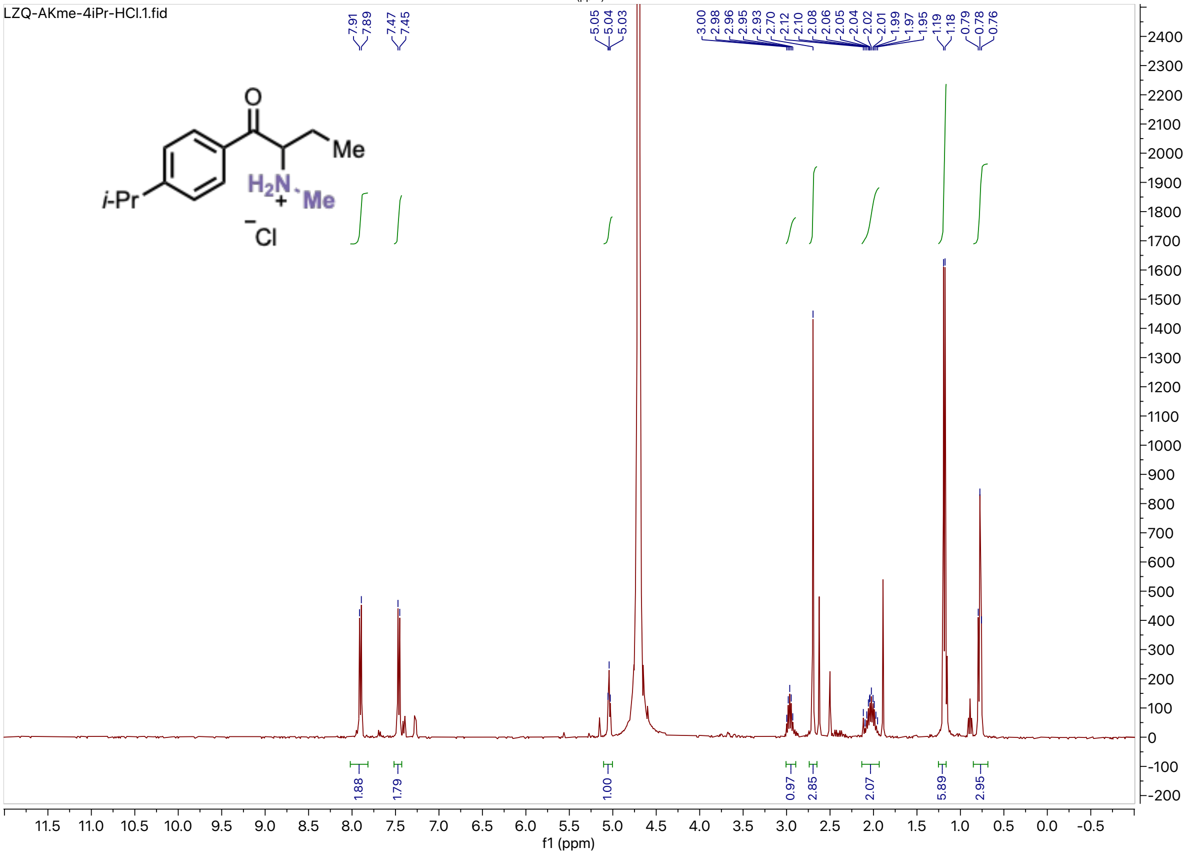
LZQ-AKme-4iPr-HCl.1.fid
f1 (ppm)

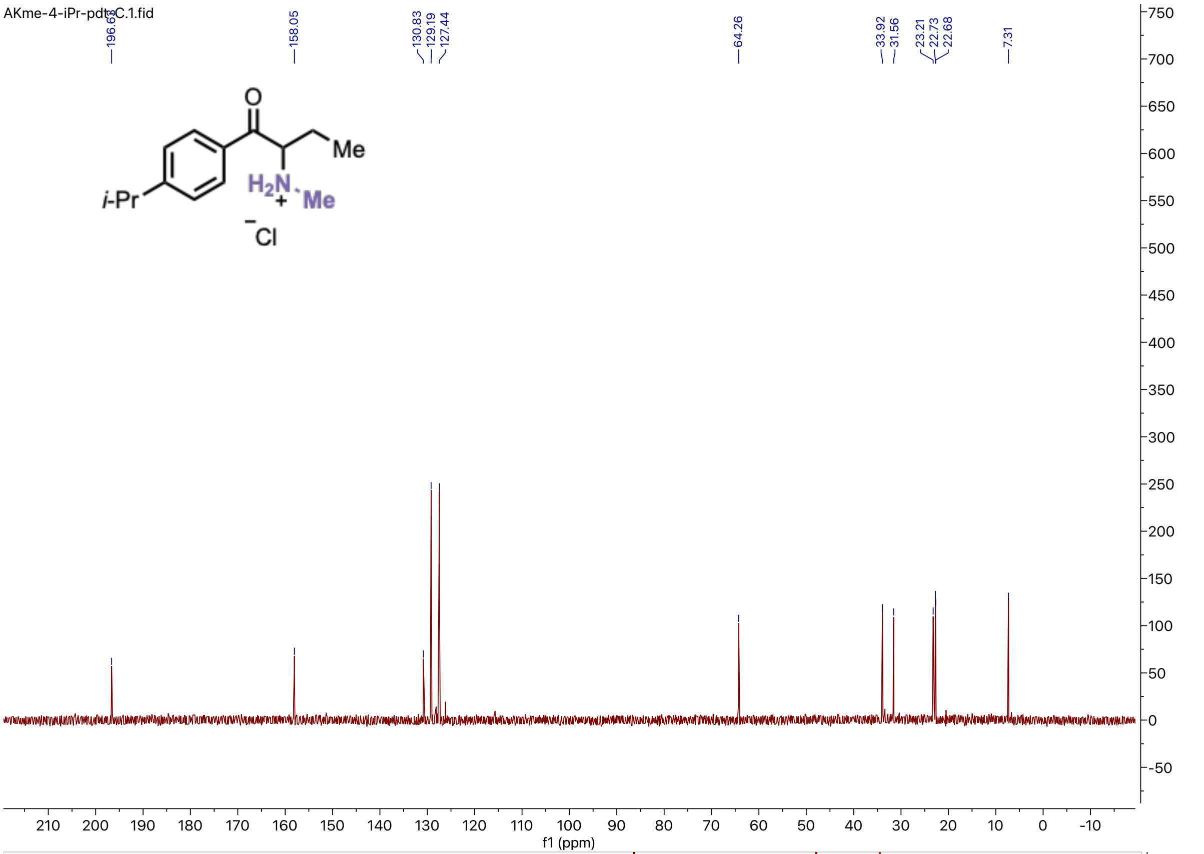
AKme-4-iPr-pdt-C.1.fid
196.63
158.05
130.83
129.19
127.44
64.26
33.92
31.56
23.21
22.73
22.68
7.31
i-Pr
Me
O
Me
Cl
f1 (ppm)

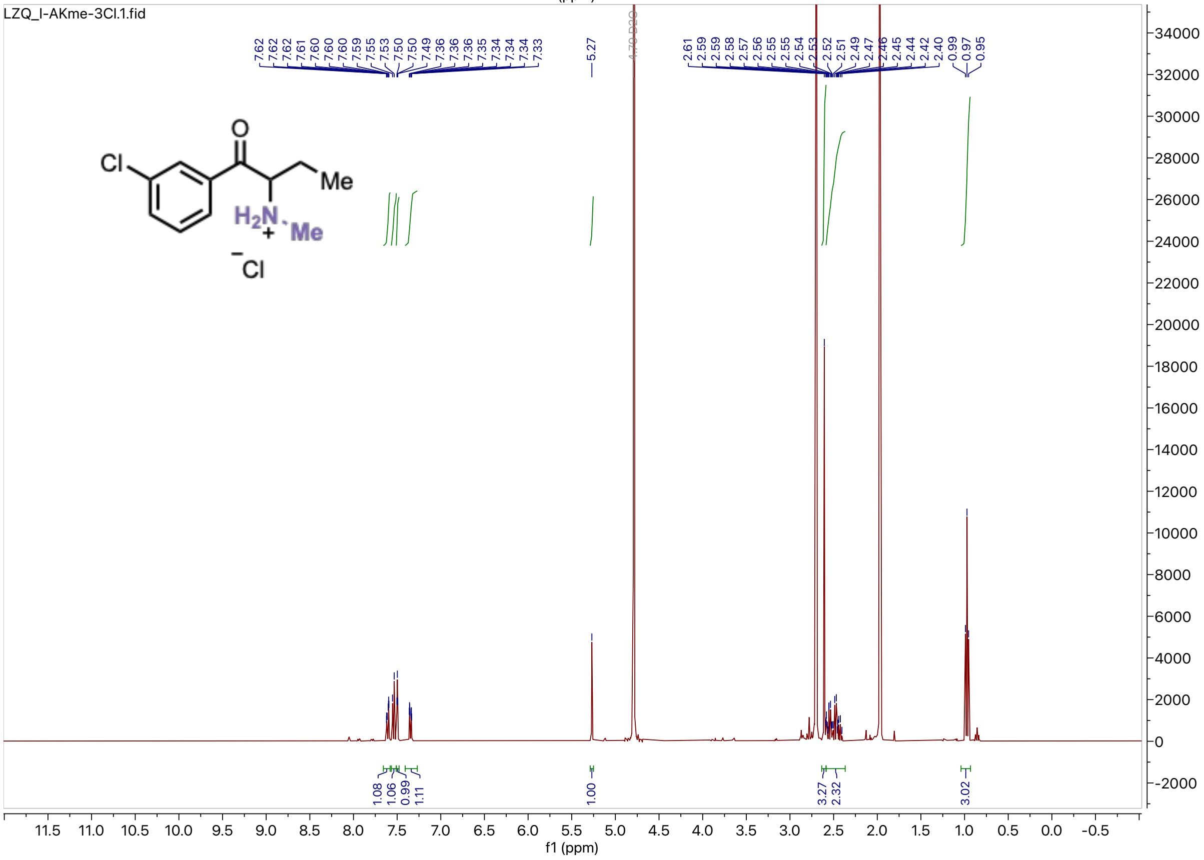
LZQ_I-AKme-3Cl.1.fid
7.62
7.62
7.62
7.61
7.60
7.60
7.60
7.59
7.55
7.53
7.50
7.50
7.49
7.36
7.36
7.36
7.35
7.34
7.34
7.33
5.27
2.61
2.59
2.59
2.58
2.57
2.56
2.55
2.55
2.54
2.53
2.52
2.51
2.49
2.47
2.46
2.45
2.44
2.42
2.40
0.99
0.97
0.95
Cl
O
Me
Me
Cl
1.08
1.06
0.99
1.11
1.00
3.27
2.32
3.02
f1 (ppm)

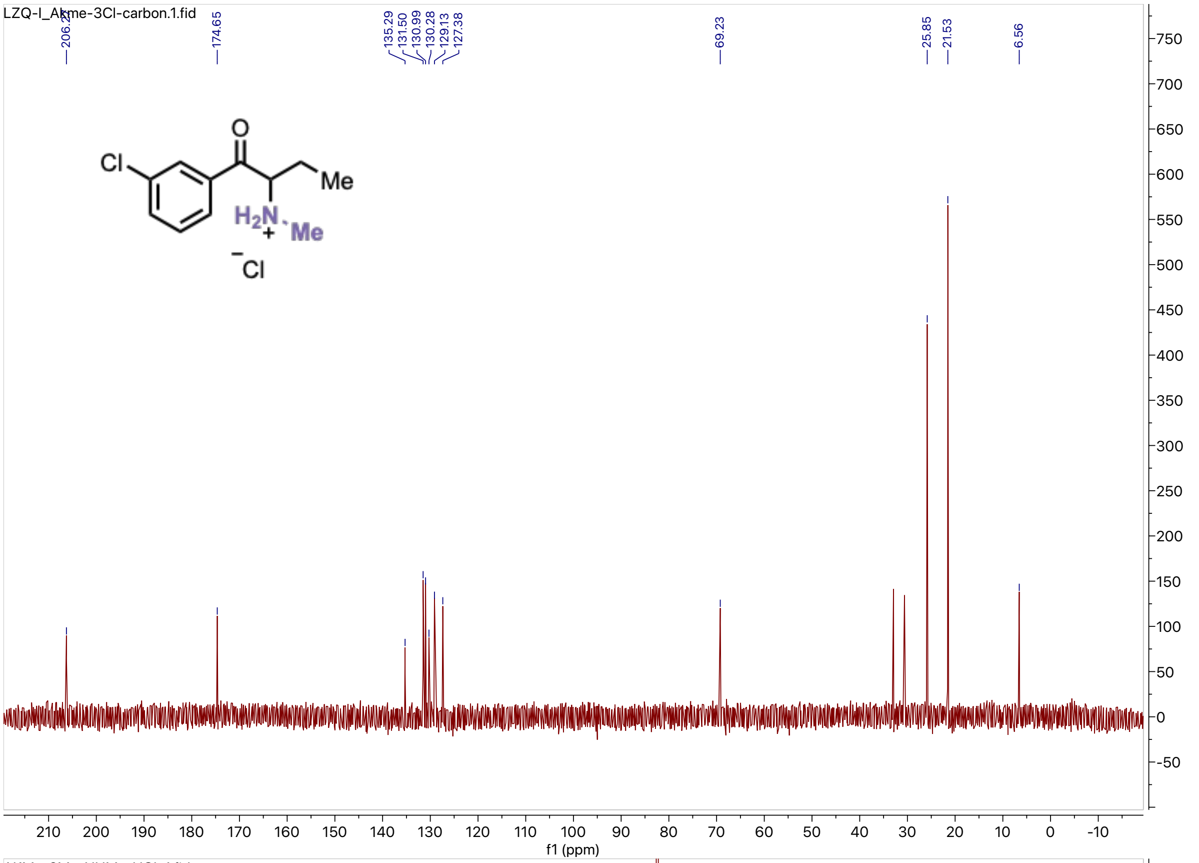
LZQ-I_Akme-3Cl-carbon.1.fid
206.2
174.65
135.29
131.50
130.99
130.28
129.13
127.38
69.23
25.85
21.53
6.56
f1 (ppm)

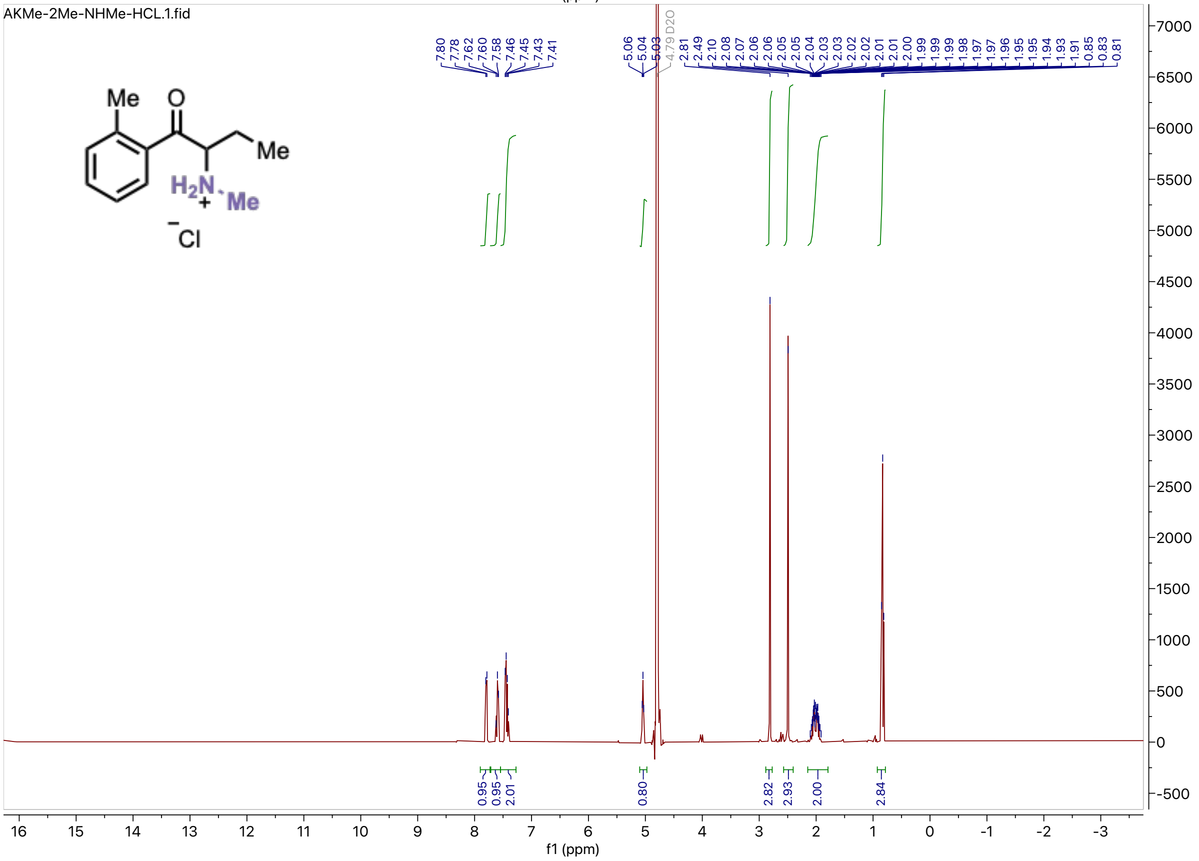
AKMe-2Me-NHMe-HCL.1.fid
4.79 D2O
0.95
0.95
2.01
0.80
2.82
2.93
2.00
2.84
f1 (ppm)

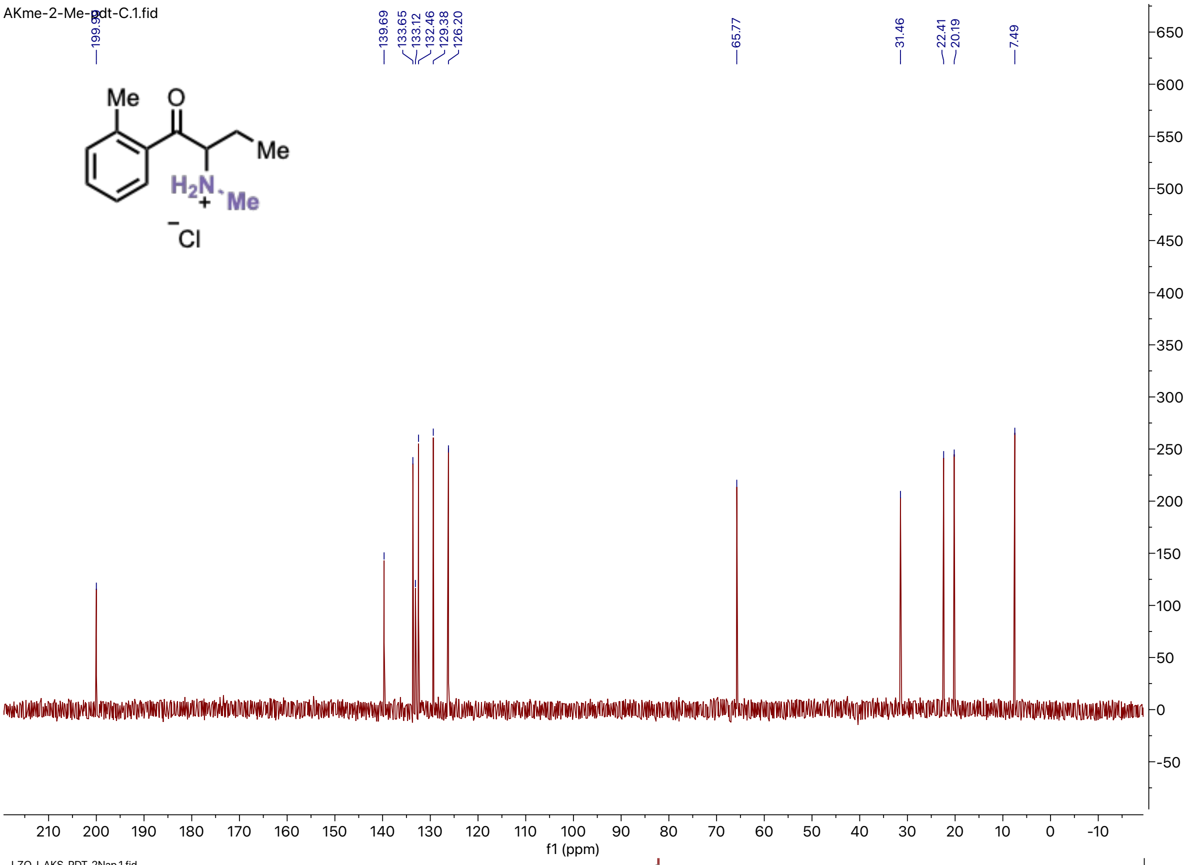
AKme-2-Me-pdt-C.1.fid
199.9
139.69
133.65
133.12
132.46
129.38
126.20
65.77
31.46
22.41
20.19
7.49
Me
O
Me
H2N
+
Me
Cl
f1 (ppm)

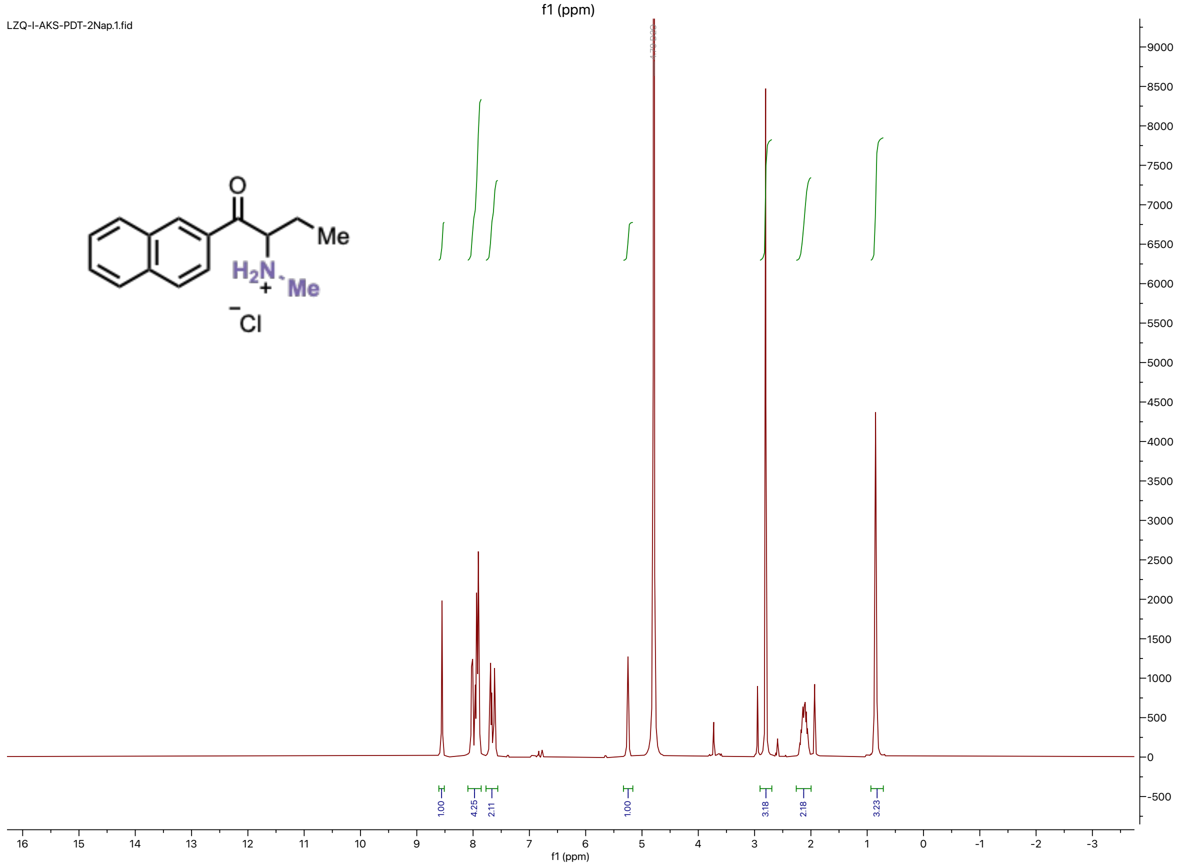
LZQ-I-AKS-PDT-2Nap.1.fid
O
Me
H2N
+
Me
Cl
1.00
4.25
2.11
1.00
3.18
2.18
3.23
f1 (ppm)

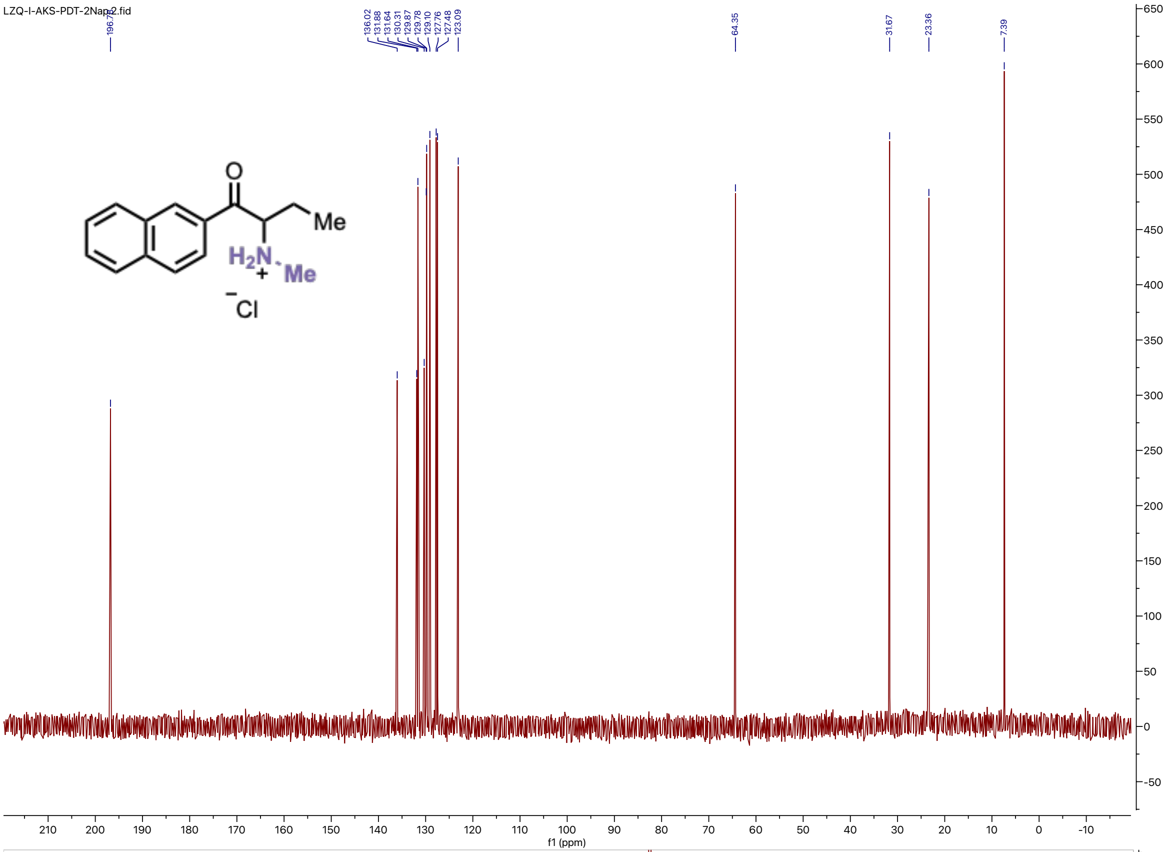
LZQ-I-AKS-PDT-2Nap2.fid
196.74
136.02
131.88
131.64
130.31
129.87
129.78
129.10
127.76
127.48
123.09
64.35
31.67
23.36
7.39
Me
H2N
Me
Cl
f1 (ppm)

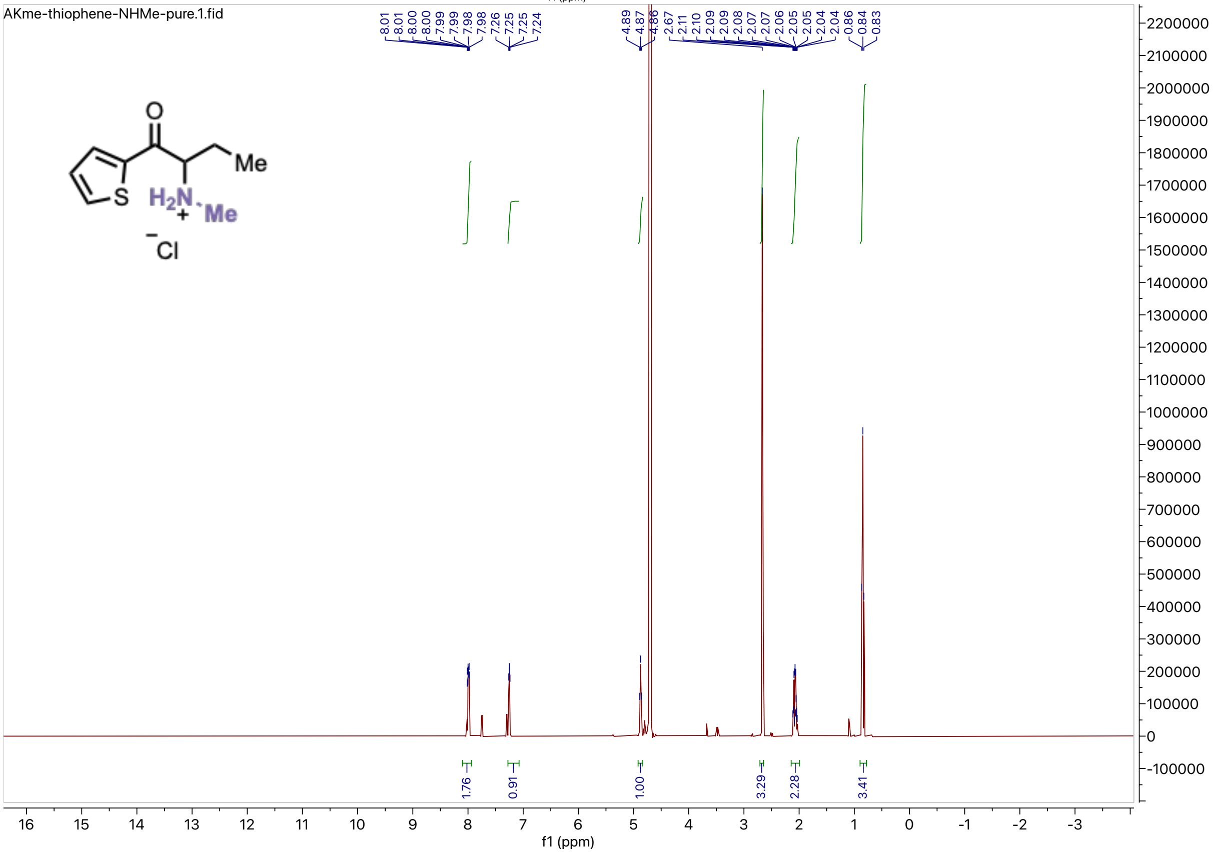
AKme-thiophene-NHMe-pure.1.fid
8.01
8.01
8.00
8.00
7.99
7.99
7.98
7.98
7.26
7.25
7.25
7.24
4.89
4.87
4.86
2.67
2.11
2.10
2.09
2.09
2.08
2.07
2.07
2.06
2.05
2.05
2.04
2.04
0.86
0.84
0.83
S
Me
H2N
Me
Cl
1.76
0.91
1.00
3.29
2.28
3.41
f1 (ppm)

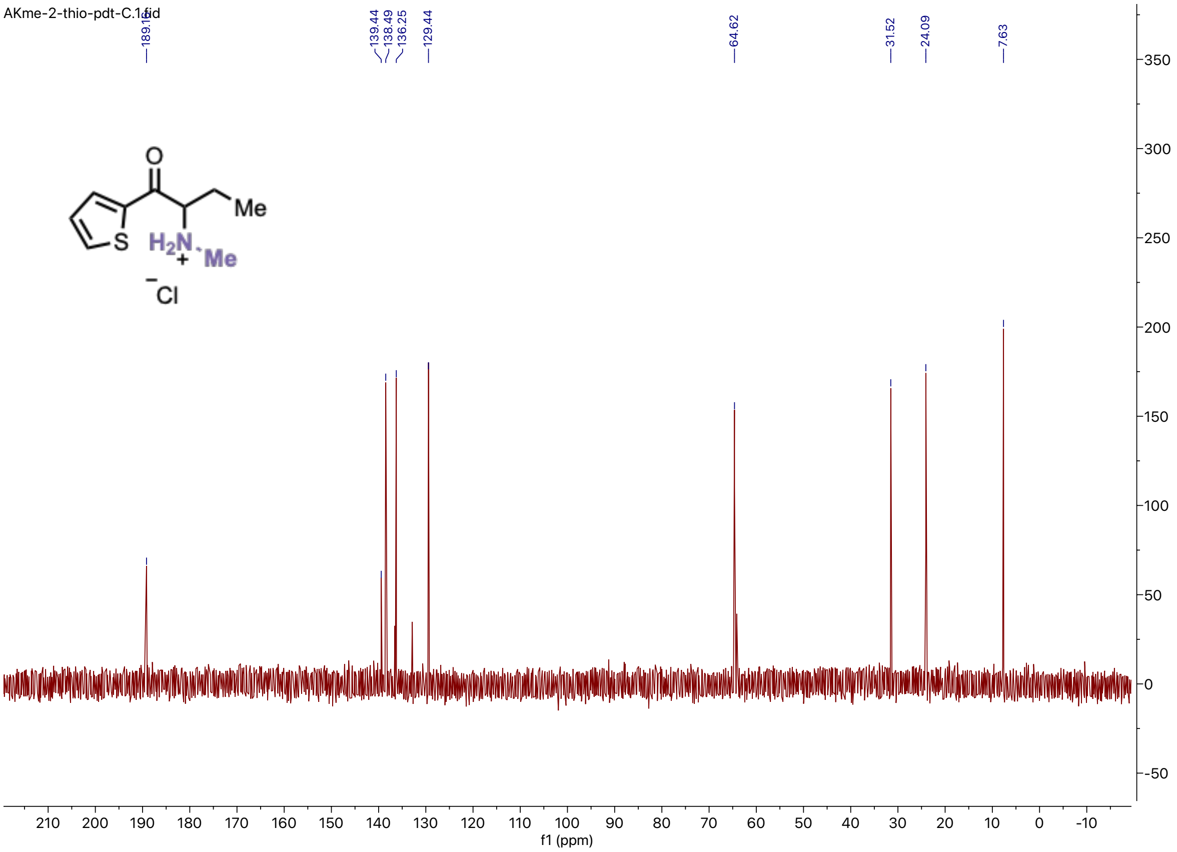

AKme-2-thio-pdt-C.1.fid
189.16
139.44
138.49
136.25
129.44
64.62
31.52
24.09
7.63
O
Me
S
H2N
Me
Cl
f1 (ppm)

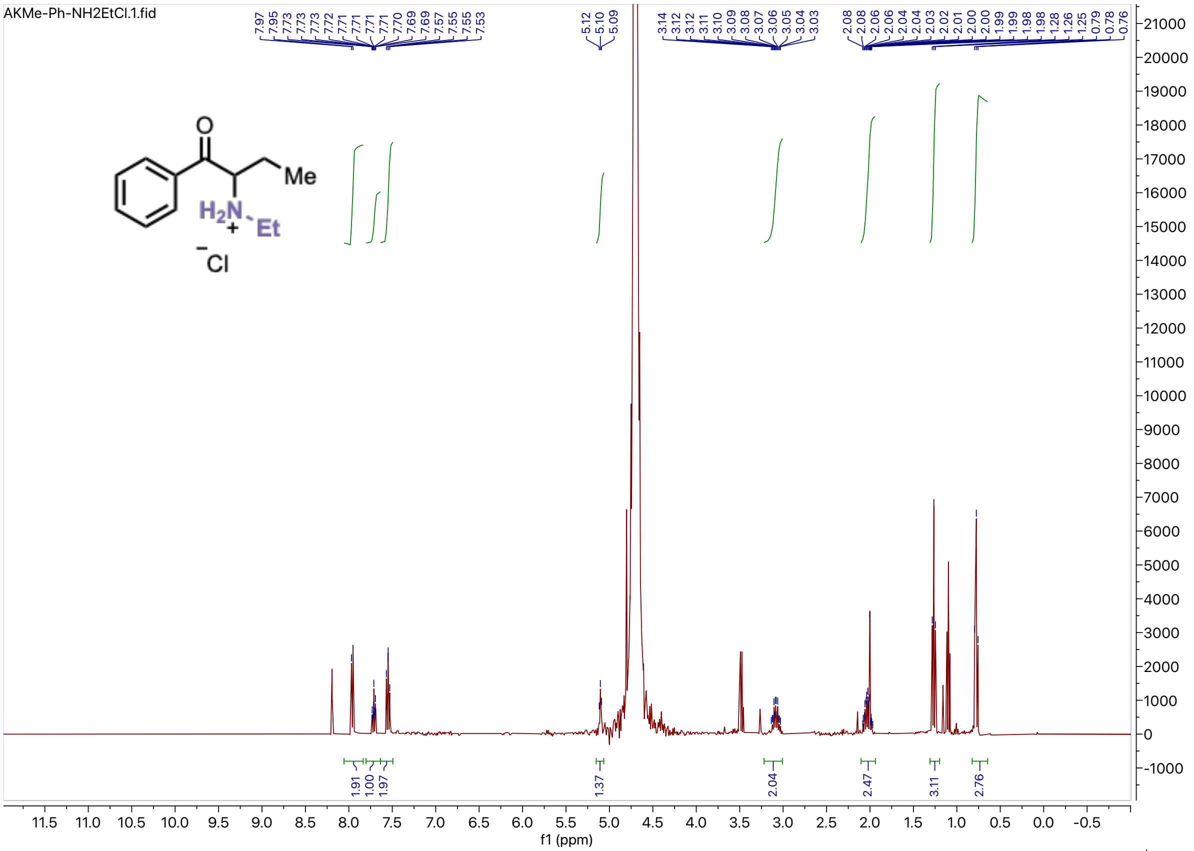
AKMe-Ph-NH2EtCl.1.fid
Me
H_2N
Et
Cl
f1 (ppm)

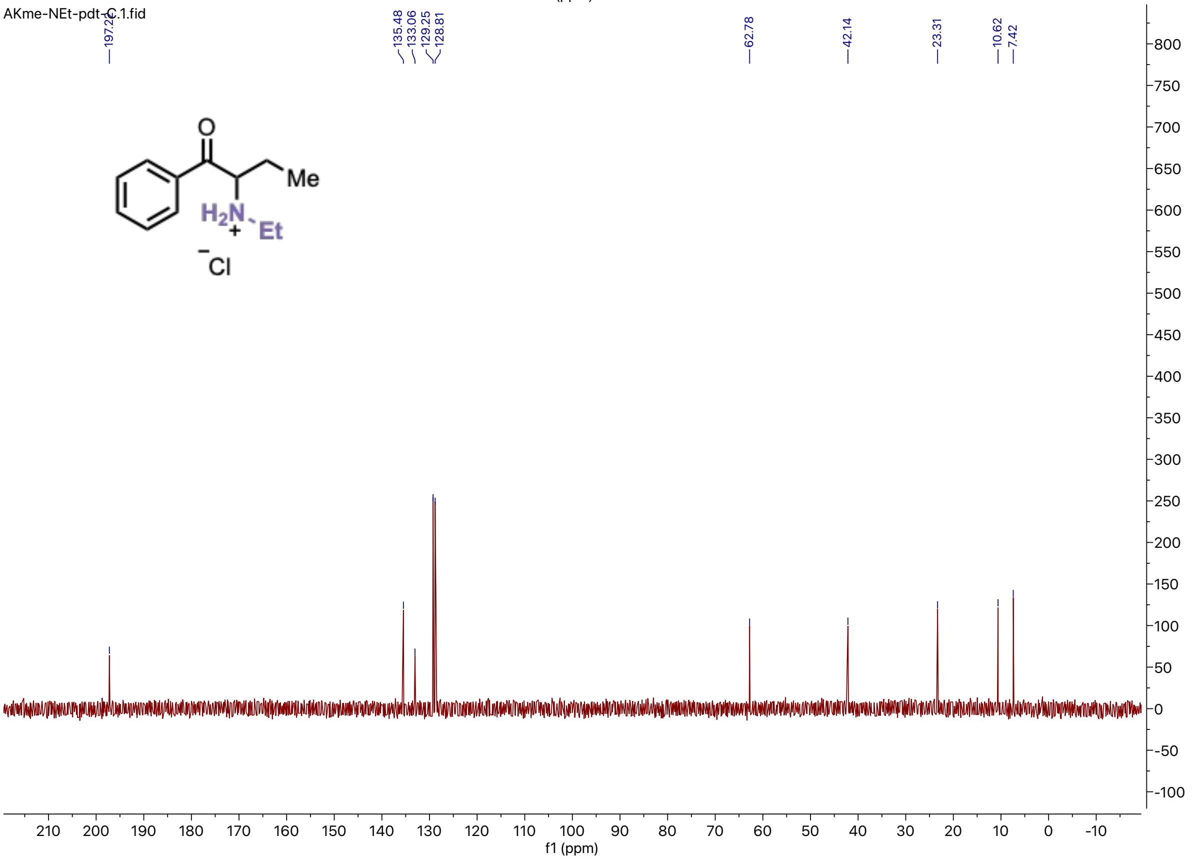
AKme-NEt-pdt-C.1.fid
197.2
135.48
133.06
129.25
128.81
62.78
42.14
23.31
10.62
7.42
Me
H_2N
Et
Cl
f1 (ppm)

XII. References

(1). Gibson, D. G.; Young, L.; Chuang, R.-Y.; Venter, J. C.; Hutchinson III, C. A.; Smith, H. O. Enzymatic assembly of DNA molecules up to several hundred kilobases. *Nat. Methods* **2009**, *6*, 343–345.

(2). Kille, S.; Acevedo-Rocha, C. G.; Parra, L. P.; Zhang, Z.-G.; Opperman, D. J.; Reetz, M. T.; Acevedo J. P. Reducing Codon Redundancy and Screening Effort of Combinatorial Protein Libraries Created by Saturation Mutagenesis. *ACS Synth. Biol.* **2013**, *2*, 83–92.

(3). Boville, C. E.; Scheele, R. A.; Koch, P.; Brinkmann-Chen, S.; Buller, A. R.; Arnold, F. H. Engineered Biosynthesis of β-Alkyl Tryptophan Analogues. *Angew. Chem. Int. Ed.* **2018**, *57*, 14764–14768.

(4). Zhao, H.; Giver, L.; Shao, Z.; Affholter, J. A.; Arnold, F. H. Molecular Evolution by Staggered Extension Process (StEP) in Vitro Recombination. *Nat. Biotechnol.* **1998**, *16*, 258–261.

(5). Spinnato, D.; Schweitzer-Chaput, B.; Goti, G.; Ošeka, M.; Melchiorre, P. A Photochemical Organocatalytic Strategy for the α-Alkylation of Ketones by Using Radicals. *Angew. Chem. Int. Ed.* **2020**, *59*, 9485–9490.

(6). Mao, R.; Taylor, D. M.; Wackelin, D. J.; Rogge, T.; Wu, S. J.; Sicinski, K. M.; Houk, K. N.; Arnold, F. H. Biocatalytic, Stereoconvergent Alkylation of (*Z*/*E*)-Trisubstituted Silyl Enol Ethers. *Nat. Synth.* **2023**, *3*, 256–264.

(7). Zhang, H.; Xu, P.; Wang, T.; Wang, S.; Li, W.; Mao, J.; Wang, J.; Zhang, F.; Cheng, M. Design, Synthesis and Biological Evaluation of Highly Potent and Selective CYP1B1 Inhibitors. *New J. Chem.* **2023**, *47*, 5680–5690.

(8). Frisch, M. J.; Trucks, G. W.; Schlegel, H. B.; Scuseria, G. E.; Robb, M. A.; Cheeseman, J. R.; Scalmani, G.; Barone, V.; Petersson, G. A.; Nakatsuji, H.; Li, X.; Caricato, M.; Marenich, A. V.; Bloino, J.; Janesko, B. G.; Gomperts, R.; Mennucci, B.; Hratchian, H. P.; Ortiz, J. V.; Izmaylov, A. F.; Sonnenberg, J. L.; Williams-Young, D.; Ding, F.; Lipparini, F.; Egidi, F.; Goings, J.; Peng, B.; Petrone, A.; Henderson, T.; Ranasinghe, D.; Zakrzewski, V. G.; Gao, J.; Rega, N.; Zheng, G.; Liang, W.; Hada, M.; Ehara, M.; Toyota, K.; Fukuda, R.; Hasegawa, J.; Ishida, M.; Nakajima, T.; Honda, Y.; Kitao, O.; Nakai, H.; Vreven, T.; Throssell, K.; Jr, J. A. M.; Peralta, J. E.; Ogliaro, F.; Bearpark, M. J.; Heyd, J. J.; Brothers, E. N.; Kudin, K. N.; Staroverov, V. N.; Keith, T. A.; Kobayashi, R.; Normand, J.; Raghavachari, K.; Rendell, A. P.; Burant, J. C.; Iyengar, S. S.; Tomasi, J.; Cossi, M.; Millam, J. M.; Klene, M.; Adamo, C.; Cammi, R.; Ochterski, J. W.; Martin, R. L.; Morokuma, K.; Farkas, O.; Foresman, J. B.; Fox, D. J. *Gaussian 16 Revision A.03*; Gaussian, Inc.: Wallingford CT, 2016.

(9). Rogge, T.; Zhou, Q.; Porter, N. J.; Arnold, F. H.; Houk, K. N. Iron Heme Enzyme-Catalyzed Cyclopropanations with Diazirines as Carbene Precursors: Computational

Explorations of Diazirine Activation and Cyclopropanation Mechanism. *J. Am. Chem. Soc.* **2024**, *146*, 2959–2966.

(10). Caddell Haatveit, K.; Garcia-Borràs, M.; Houk, K. N. Computational Protocol to Understand P450 Mechanisms and Design of Efficient and Selective Biocatalysts. *Frontiers in Chemistry* **2019**, *Volume 6-2018*.

(11). Yang, Y.; Cho, I.; Qi, X.; Liu, P.; Arnold, F. H. An Enzymatic Platform for the Asymmetric Amination of Primary, Secondary and Tertiary C(sp^3)–H Bonds. *Nature Chemistry* **2019**, *11*, 987–993.

(12). Becke, A. D. Density-Functional Thermochemistry. III. The Role of Exact Exchange. *The Journal of Chemical Physics* **1993**, *98*, 5648–5652.

(13). Lee, C.; Yang, W.; Parr, R. G. Development of the Colle-Salvetti Correlation-Energy Formula into a Functional of the Electron Density. *Phys. Rev. B* **1988**, *37*, 785–789.

(14). Grimme, S.; Antony, J.; Ehrlich, S.; Krieg, H. A Consistent and Accurate *Ab Initio* Parametrization of Density Functional Dispersion Correction (DFT-D) for the 94 Elements H-Pu. *The Journal of Chemical Physics* **2010**, *132*, 154104.

(15). Grimme, S.; Ehrlich, S.; Goerigk, L. Effect of the Damping Function in Dispersion Corrected Density Functional Theory. *Journal of Computational Chemistry* **2011**, *32*, 1456–1465.

(16). Barone, V.; Cossi, M. Quantum Calculation of Molecular Energies and Energy Gradients in Solution by a Conductor Solvent Model. *J. Phys. Chem. A* **1998**, *102*, 1995–2001.

(17). Cossi, M.; Rega, N.; Scalmani, G.; Barone, V. Energies, Structures, and Electronic Properties of Molecules in Solution with the C-PCM Solvation Model. *Journal of Computational Chemistry* **2003**, *24*, 669–681.

(18). Li, C.; Wu, W.; Cho, K.-B.; Shaik, S. Oxidation of Tertiary Amines by Cytochrome P450—Kinetic Isotope Effect as a Spin-State Reactivity Probe. *Chemistry – A European Journal* **2009**, *15*, 8492–8503.

(19). Schöneboom, J. C.; Lin, H.; Reuter, N.; Thiel, W.; Cohen, S.; Ogliaro, F.; Shaik, S. The Elusive Oxidant Species of Cytochrome P450 Enzymes: Characterization by Combined Quantum Mechanical/Molecular Mechanical (QM/MM) Calculations. *J. Am. Chem. Soc.* **2002**, *124*, 8142–8151.

(20). Usharani, D.; Janardanan, D.; Shaik, S. Does the TauD Enzyme Always Hydroxylate Alkanes, While an Analogous Synthetic Non-Heme Reagent Always Desaturates Them? *J. Am. Chem. Soc.* **2011**, *133*, 176–179.

(21). Altun, A.; Breidung, J.; Neese, F.; Thiel, W. Correlated Ab Initio and Density Functional Studies on H2 Activation by FeO+. *J. Chem. Theory Comput.* **2014**, *10*, 3807–3820.

(22). Lewis, R. D.; Garcia-Borràs, M.; Chalkley, M. J.; Buller, A. R.; Houk, K. N.; Kan, S. B. J.; Arnold, F. H. Catalytic Iron-Carbene Intermediate Revealed in a Cytochrome *c* Carbene Transferase. *Proceedings of the National Academy of Sciences* **2018**, *115*, 7308–7313.

(23). Sharon, D. A.; Mallick, D.; Wang, B.; Shaik, S. Computation Sheds Insight into Iron Porphyrin Carbenes' Electronic Structure, Formation, and N–H Insertion Reactivity. *J. Am. Chem. Soc.* **2016**, *138*, 9597–9610.

(24). Shaik, S.; Cohen, S.; Wang, Y.; Chen, H.; Kumar, D.; Thiel, W. P450 Enzymes: Their Structure, Reactivity, and Selectivity—Modeled by QM/MM Calculations. *Chem. Rev.* **2010**, *110*, 949–1017.

(25). Qin, Z.-Y.; Gao, S.; Zou, Y.; Liu, Z.; Wang, J. B.; Houk, K. N.; Arnold, F. H. Biocatalytic Construction of Chiral Pyrrolidines and Indolines via Intramolecular C(Sp^3)–H Amination. *ACS Cent. Sci.* **2023**, *9*, 2333–2338.

(26). Liu, Z.; Qin, Z.-Y.; Zhu, L.; Athavale, S. V.; Sengupta, A.; Jia, Z.-J.; Garcia-Borràs, M.; Houk, K. N.; Arnold, F. H. An Enzymatic Platform for Primary Amination of 1-Aryl-2-Alkyl Alkynes. *J. Am. Chem. Soc.* **2022**, *144*, 80–85.

(27). Hirshfeld, F. L. Bonded-Atom Fragments for Describing Molecular Charge Densities. *Theoretica chimica acta* **1977**, *44*, 129–138.

(28). Lu, T.; Chen, F. Multiwfn: A Multifunctional Wavefunction Analyzer. *J Comput Chem* **2012**, *33*, 580–592.

(29). Lu, T. A Comprehensive Electron Wavefunction Analysis Toolbox for Chemists, Multiwfn. *The Journal of Chemical Physics* **2024**, *161*, 082503.

(30). Humphrey, W.; Dalke, A.; Schulten, K. VMD: Visual Molecular Dynamics. *Journal of Molecular Graphics* **1996**, *14*, 33–38.

(31). Legault, C. Y. CYLview, Version 1.0b, 2009. http://www.cylview.org.

(32). Liu, Z.; Qin, Z.-Y.; Zhu, L.; Athavale, S. V.; Sengupta, A.; Jia, Z.-J.; Garcia-Borràs, M.; Houk, K. N.; Arnold, F. H. An Enzymatic Platform for Primary Amination of 1-Aryl-2-Alkyl Alkynes. *J. Am. Chem. Soc.* **2022**, *144*, 80–85.

(33). Ohmatsu, K.; Nakashima, T.; Sato, M.; Ooi, T. Direct Allylic C–H Alkylation of Enol Silyl Ethers Enabled by Photoredox-Brønsted Base Hybrid Catalysis. *Nat. Commun.* **2019**, *10*, 2706.

(34). Mei, J.-H.; Zeng, Y.-R.; Gong, Y.-N.; Shi, W.-J.; Zhong, D.-C.; Lu, T.-B. π–π Stacking as Electron-Transfer Channels in Hydrogen-Bonded Organic Frameworks for Boosting Photocatalysis. *Angew. Chem. Int. Ed.* **2025**, *64* (29), e202507332.

(35). Friesner, R. A.; Banks, J. L.; Murphy, R. B.; Halgren, T. A.; Klicic, J. J.; Mainz, D. T.; Repasky, M. P.; Knoll, E. H.; Shelley, M.; Perry, J. K.; Shaw, D. E.; Francis, P.; Shenkin, P. S. Glide: A New Approach for Rapid, Accurate Docking and Scoring. 1. Method and Assessment of Docking Accuracy. *J. Med. Chem.* **2004**, *47*, 1739–1749.

(36). Halgren, T. A.; Murphy, R. B.; Friesner, R. A.; Beard, H. S.; Frye, L. L.; Pollard, W. T.; Banks, J. L. Glide: A New Approach for Rapid, Accurate Docking and Scoring. 2. Enrichment Factors in Database Screening. *J. Med. Chem.* **2004**, *47*, 1750–1759.

(37). Chai Discovery team; Boitreaud, J.; Dent, J.; McPartlon, M.; Meier, J.; Reis, V.; Rogozhonikov, A.; Wu, K. Chai-1: Decoding the Molecular Interactions of Life. *bioRxiv* **2024**, 2024.10.10.615955.
